# Mechanism and cellular actions of the potent AMPK inhibitor BAY-3827

**DOI:** 10.1126/sciadv.adx2434

**Published:** 2025-08-22

**Authors:** Conchita Fraguas Bringas, Mohd Syed Ahangar, Joyceline Cuenco, Hongling Liu, Alex B. Addinsall, Maria Lindahl, Ashley J. Ovens, Mark A. Febbraio, Marc Foretz, Olga Göransson, John W. Scott, Elton Zeqiraj, Kei Sakamoto

**Affiliations:** ^1^ Novo Nordisk Foundation Center for Basic Metabolic Research, Faculty of Health and Medical Sciences, University of Copenhagen, Copenhagen 2200, Denmark.; ^2^ Astbury Centre for Structural Molecular Biology, School of Molecular and Cellular Biology, Faculty of Biological Sciences, University of Leeds, Leeds LS2 9JT, UK.; ^3^ Department of Experimental Medical Science, Lund University, Lund 22184, Sweden.; ^4^ St.Vincent's Institute of Medical Research, Fitzroy, Victoria 3065, Australia.; ^5^ Drug Discovery Biology, Monash Institute of Pharmaceutical Sciences, Parkville, Victoria 3052, Australia.; ^6^ Université Paris Cité, CNRS, Inserm, Institut Cochin, Paris 75014, France.

## Abstract

Inhibition of adenosine 5′-monophosphate (AMP)–activated protein kinase (AMPK) is under increasing investigation for its therapeutic potential in many diseases. Existing AMPK inhibitors are however limited, with poor selectivity and substantial off-target effects. Here, we provide mechanistic insights and describe the cellular selectivity of the recently identified AMPK inhibitor BAY-3827. A 2.5-Å cocrystal structure of the AMPK kinase domain with BAY-3827 revealed distinct features including a disulfide bridge between the αD helix Cys^106^ and the activation loop residue Cys^174^. This bridge appears to stabilize the activation loop such that Asn^162^ repositions the Asp-Phe-Gly (DFG) motif Phe^158^ toward the C-terminal lobe, displacing His^137^ and disrupting the regulatory spine, promoting an inactive kinase state. In hepatocytes, BAY-3827 blocked AMPK activator (MK-8722)–mediated phosphorylation of ACC1 and corresponding inhibition of lipogenesis. Transcriptome analysis revealed that BAY-3827 down-regulated ~30% of MK-8722–stimulated AMPK-dependent genes. We establish the molecular and cellular basis of BAY-3827’s selectivity and utility for delineating AMPK functions while highlighting its limitations.

## Teaser

The mechanism and unique molecular/cellular features of the potent and selective AMPK inhibitor BAY-3827 are unraveled.

## INTRODUCTION

Adenosine 5′-monophosphate (AMP)–activated protein kinase (AMPK) governs cellular energy metabolism and coordinates a myriad of cellular functions to maintain organismal homeostasis (*1*–*3*). AMPK is ubiquitously expressed in eukaryotic cells as heterotrimeric complexes comprising a catalytic α and regulatory β and γ subunits. In mammals, the three subunits are encoded by multiple genes, giving rise to seven subunit isoforms (α1, α2; β1, β2; and γ1, γ2, and γ3) that can generate up to 12 distinct heterotrimeric complexes (*4*, *5*). AMPK is activated through phosphorylation of Thr^172^ within the activation loop of the α subunit kinase domain (KD). The upstream kinases phosphorylating Thr^172^ are liver kinase B1 (LKB1) and Ca^2+^-calmodulin–dependent protein kinase kinase-2 (CaMKK2) (*1*, *2*). Thr^172^ is located in the activation loop of the catalytic α subunit, and the negative charge added upon residue phosphorylation leads to a conformational change that prepares AMPK for substrate phosphorylation, acquiring an active kinase state. The Thr^172^-containing activation loop is stabilized via charge interactions with positively charged αC helix Lys^60^ and Arg^138^ and with Asn^162^, swinging outward to direct the γ-phosphate of adenosine 5′-triphosphate (ATP) toward the peptide substrate. The conserved Asp-Phe-Gly (DFG) kinase motif will point away from the ATP-binding pocket, interacting with the conserved Lys^45^ residue and with ATP-Mg^2+^ interacting with Asp^157^ (*6*, *7*), allowing for phosphoryl transfer to take place. The γ subunits contain four tandem cystathionine β-synthase (CBS) repeats, creating three functional nucleotide-binding sites that provide AMPK with its energy-sensing capabilities (*5*). The binding of adenosine 5′-diphosphate (ADP) and/or AMP to CBS motifs causes conformational changes that increase net Thr^172^ phosphorylation. In addition, the binding of AMP further increases AMPK activity through allosteric stimulation. Although ADP has been shown to act as a modest allosteric activator of AMPK under certain conditions, AMP is still considered the more physiologically relevant activator in vivo (*8*). AMPK activity is therefore controlled by multiple regulatory mechanisms, often involving a combination of posttranslational modifications and allosteric regulation.

AMPK has long held promise as a therapeutic target for metabolic syndrome, as its physiological and pharmacological activation in multiple metabolic tissues has resulted in the amelioration of insulin resistance and reversal of hyperglycemia in preclinical studies (*9*). For example, active AMPK inhibits fatty acid and cholesterol biosynthesis in the liver through phosphorylation and inactivation of acetyl–coenzyme A (CoA) carboxylase-1 (ACC1) and 3-hydroxy-3-methylglutaryl (HMG)–CoA reductase, respectively (*10*, *11*). The activation of AMPK leads to increased fatty acid oxidation through phosphorylation of ACC2 and also promotion of plasma membrane localization of GLUT4 partly via TBC1D1 phosphorylation in an insulin-independent manner, promoting glucose uptake in skeletal muscle (*3*, *12*–*14*). These beneficial effects of AMPK activation prompted the pharmaceutical development of small-molecule activators of AMPK, resulting in the identification of several potent and specific allosteric activators, including A-769662 (*15*, *16*), 991 (*17*), MK-8722 (*17*, *18*), and PF-739 (*19*). These compounds bind into a pocket termed the allosteric drug and metabolite (ADaM) site (*7*) located between the α and β subunits (*9*, *17*). To date, some ADaM site binding compounds have demonstrated promising efficacy in reversing hyperglycemia in preclinical proof-of-concept studies (*18*, *19*). Although current allosteric pan-AMPK activators have the potential risk of causing cardiac hypertrophy, isoform-selective activators are being developed to mitigate safety issues (*9*). Moreover, these highly potent and selective allosteric activators serve as valuable research tools and have profoundly contributed to delineating AMPK functions.

In cancer, AMPK has a dual role, as a tumor promoter or suppressor, depending on, for instance, subcellular context, trimeric complex formation, and upstream regulators (*20*, *21*). Previously, AMPK was viewed as a tumor suppressor, based on its ability to inhibit anabolic processes and mediate the tumor suppressive effects of LKB1, such as promoting cell growth through the inactivation of mTORC1 (*22*–*24*). Conversely, more recent studies have supported the notion that increased AMPK activity may help to promote tumor growth and survival, alongside conferring drug resistance and resilience under tumor hypoxia and nutrient deprivation (*20*, *25*). In such conditions in established tumors, AMPK inhibitors might be efficacious tools for cancer treatment. Specifically, they may be particularly effective in cases where the *PRKAA1* or *PRKAB2* genes are amplified, causing aberrantly high expression of AMPK. (*21*, *26*).

In contrast to activators, the availability of selective AMPK inhibitors is limited. The first and most commonly used AMPK inhibitor, the pyrazolopyrimidine derivative compound C, was identified in an in vitro high-throughput screen, and although its selectivity was poorly characterized, it was used to probe for AMPK-dependent metabolic actions of the antidiabetes drug metformin in hepatocytes (*27*). Compound C was “rediscovered” in an in vivo compound screen in zebrafish embryos as an inhibitor of the bone morphogenetic protein (BMP) pathway (e.g., BMP type 1 receptors ALK2, 3, and 6) and was renamed “dorsomorphin” as it induced dorsalization in embryos (*28*). Cocrystal structure studies have established that compound C/dorsomorphin binds to the highly conserved ATP-binding pocket in the KD of AMPKα (*29*) and ALK2 (*30*). However, multiple in vitro kinase activity screens studying inhibitor selectivity have verified that compound C is not a selective inhibitor of AMPK nor BMP receptor kinases (*31*–*33*), as, for example, it has been reported to inhibit the activities of 34 of 119 kinases more potently than AMPK at 1 μM (*32*). In line with this, numerous cellular studies to date have shown that compound C affects a diverse range of biological processes via AMPK-independent mechanisms (*34*).

SBI-0206965, a 2-aminopyrimidine derivative, was originally identified through a cell-based screen and described as a selective inhibitor of the autophagy initiator kinase ULK1, with the ability to inhibit ULK signaling and ULK1-mediated survival of cancer cells (*35*). Recent studies have reported that SBI-0206965 inhibits AMPK and ULK1/2 with a similar potency (*36*) and in a cell-free assay, SBI-0206965 inhibited AMPK with a median inhibitory concentration (IC_50_) of 0.16 μM at 20 μM ATP, compared to a value of 1.9 μM with compound C (*33*). Although SBI-0206965 demonstrated much higher potency and selectivity toward AMPK in cell-free assays than compound C, several other kinases including NUAK1, FAK, MLK1/3, and MARK3/4, all of which contain a methionine in their gatekeeper position in the KD, were also potently inhibited (*36*). In cellular studies, SBI-0206965 dose-dependently blocked 991-induced phosphorylation of ACC1 and inhibition of lipogenesis in mouse primary hepatocytes. Notably, SBI-0206965 also inhibited glucose and nucleoside transport systems in adipocytes and myotubes (*36*).

More recently, a potent and selective AMPK inhibitor termed BAY-3827 was identified in a high- throughput compound screen and underwent chemical optimization. In cell-free assays, BAY-3827 inhibited AMPK with IC_50_ values of 1.4 nM at low (10 μM) ATP and 15 nM at high (2 mM) ATP concentrations (with 2 μM AMP under both conditions), although it inhibited 90-kDa ribosomal S6 kinase (RSK) isoforms with a similar potency (*37*). In cellular assays, BAY-3827 inhibited phosphorylation of ACC1 and showed antiproliferative effects in a subset of tumor models, namely androgen-dependent prostate cancer and myeloma cell lines (*37*). However, it is unknown whether this antiproliferative effect of BAY-3827 is mediated through inhibition of AMPK. To date, there is limited information about the molecular mechanisms of AMPK inhibition by BAY-3827. Recent biochemical analysis has characterized BAY-3827 as a mixed-type inhibitor (*38*); however, the structural mechanisms underlying its inhibitory action and the basis for its kinase specificity remain largely undefined. Here, we describe the molecular basis for BAY-3827’s inhibitory effect on AMPK through the cocrystallization of the AMPKα2 KD (α2KD) with BAY-3827. In addition, we provide cellular potency and selectivity data of BAY-3827 with a specific emphasis on AMPK-dependent transcriptional and metabolic processes (fatty acid synthesis) in AMPKα1α2^−/−^ double knockout (DKO) and AMPKα1α2^+/+^ (control) primary mouse hepatocytes.

## RESULTS

### Kinase inhibition selectivity profile of BAY-3827 and its structurally related BAY-974

**Fig. 1.**
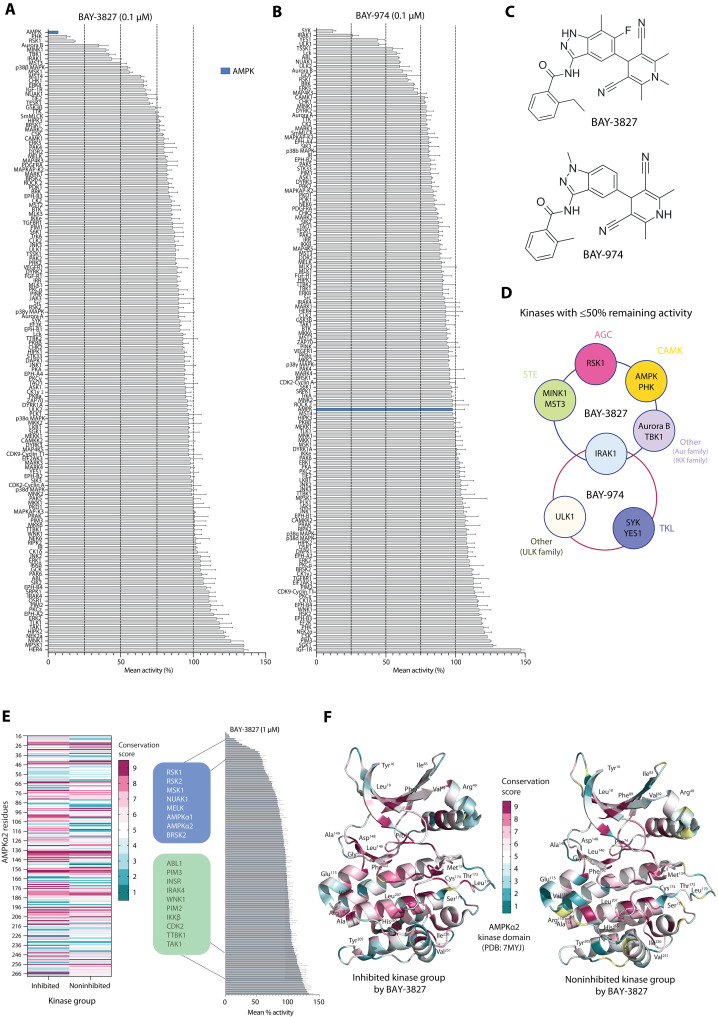
BAY-3827 but not BAY-974 selectively inhibits AMPK. Kinase activity assays were conducted across a panel of 140 human kinases, and compound selectivity of (**A**) BAY-3827 (0.1 μM) and (**B**) BAY-974 (0.1 μM) was assessed, reporting remaining kinase activity (%) from *n* = 2 in means ± SEM. (**C**) Chemical structures of BAY-3827 (N-[5-(3,5-dicyano-1,2,6-trimethyl-4H-pyridin-4-yl)-6-fluoro-7-methyl-1H-indazol-3-yl]-2-ethylbenzamide) and BAY-974 (N-[5-(3,5-dicyano-2,6-dimethyl-1,4-dihydropyridin-4-yl)-1-methylindazol-3-yl]-2-methylbenzamide) drawn in Marvin (24.3.1), 2024, ChemAxon (http://chemaxon.com). (**D**) Schematic showing the inhibitory overlap between BAY-3827 and BAY-974 of kinases with ≤50% remaining activity as well as the corresponding kinase groups they belong to. (**E**) In silico residue conservation analysis based on inhibited (≤50% remaining activity) and noninhibited (≥99% remaining activity) kinases by BAY-3827 at 1 μM across a 140-human kinase panel cross-referenced with available data (*37*). (**F**) Ribbon representation of α2KD [Protein Data Bank (PDB): 7MYJ, chain A] showing computed ConSurf conservation scale (1 to 9) colors (*40*, *41*) between BAY-3827–inhibited kinase group (left) and noninhibited kinase group (right) displaying residues with a three-point or higher scale difference between groups.

We determined the selectivity of BAY-3827 alongside its inactive control compound BAY-974, which shares a similar chemical scaffold with BAY-3827, by performing an in vitro kinase activity assay across a panel of 140 human protein kinases (Fig. 1, A and B). Both compounds were tested at 0.1 μM in the assay and consistent with previous data (*37*), AMPK was the most inhibited (>90%) by BAY-3827, followed by PHK and RSK1 (~80 to 85%). Aurora B, MINK1, TBK1, and IRAK1 were also inhibited (~60%) by BAY-3827 (Fig. 1A and table S1). The inhibitory profile of BAY-3827 in the tested panel displayed a preference for the human kinome CAMK group (which includes AMPK and PHK) and the AGC group containing RSK family and MSK1. Furthermore, Aurora kinase family Aurora B and STE group kinases MINK1 and MST3 were also highly inhibited alongside IRAK1, which belongs to the TKL group (*39*). As anticipated, BAY-974 had almost no effect on AMPK; however, it inhibited (~55 to 90%) other kinases mainly from the TK and TKL groups, including SYK, YES1, IRAK1, and ULK1 from the ULK family (Fig. 1B and table S1). These results demonstrate that BAY-3827, but not its structurally related BAY-974, selectively inhibits AMPK. This can be attributed to specific chemical alterations; BAY-974 has a missing fluoride group in the indazole ring compared to BAY-3827, a methyl group in the benzamide ring instead of BAY-3827’s ethyl group and finally a dimethyl dihydropyridine ring compared to a trimethyl H-pyridine ring present in BAY-3827 (Fig. 1C). The inhibitory profiles between BAY-3827 and BAY-974 across the panel of kinases with ≤50% remaining activity revealed a minimal overlap (Fig. 1D), making BAY-974 a suitable control compound to help dissect BAY-3827-specific effects.

Achieving a specific kinase inhibitor often involves targeting residues that are unique to the kinase of interest or poorly conserved across the kinome. On the basis of the selectivity profile of BAY-3827, we attempted to identify the pattern of this compound’s preference by studying residue conservation in the KD of highly inhibited kinases by BAY-3827 compared to those that were not inhibited. To do this comprehensively, we screened a higher concentration of BAY-3827 (1 μM) across the same kinase panel, and these data were cross-referenced with available kinase screen data at the same concentration (Fig. 1E) (*37*). We defined a BAY-3827–inhibited kinase group containing kinases that had 50% or less activity remaining after BAY-3827 treatment, and a noninhibited kinase group representing those with 99% or more remaining activity after BAY-3827 treatment. AMPK, BRSK2, MELK, NUAK1, MSK1, RSK1, and RSK2 composed the BAY-3827–inhibited group (Fig. 1E, shown in blue) and kinases ABL1, PIM3, INSR, IRAK4, WNK1, PIM2, IKKβ, CDK2, TTBK1, and TAK1 were selected as representatives of the noninhibited group (Fig. 1E, shown in green). A sequence alignment of human KDs was conducted in each group, and their conservation was studied using AMPKα1 as a reference sequence, with the inhibited group displaying 30% or higher percentage sequence identity up to 47.8% in BRSK2, whereas the noninhibited group ranged from 29.7 to 24.1% identity to AMPK (table S2). On the basis of residue conservation, a score was computed using the ConSurf server (Fig. 1, E and F, and table S2) (*40*, *41*), revealing notably different residue conservation patterns between the two kinase groups (fig. S1, A and B). We therefore hypothesized that highly conserved residues in the BAY-3827–inhibited, but not in the noninhibited kinase group, would be important for the inhibitory mechanism of BAY-3827. Residues with at least a three-point difference in the conservation scale (1 to 9) between kinase groups were displayed in the structure of AMPKα2. These were localized in four clusters: (i) in the N-terminal lobe such as Tyr^16^ and Arg^49^; (ii) near the ATP-binding pocket, including residues such as Gly^99^, Leu^146^, and Asp^148^; (iii) in the C-terminal lobe helices such as His^265^ and Val^251^; and (iv) in the activation loop, where Thr^172^ had a four-point scale decrease in conservation in the noninhibited group, and residues Cys^174^ and Leu^170^ had a notable six-point decrease in conservation compared to the BAY-3827–inhibited kinase group (Fig. 1E, fig. S1C, and table S2). Collectively, these analyses suggest that there is a conservation basis for the mechanistic inhibitory action of BAY-3827. Residues surrounding the ATP-binding pocket and the activation loop were identified as key contributors to BAY-3827’s inhibitory action, showing high conservation among BAY-3827–sensitive kinases but with low or variable conservation in BAY-3827–insensitive kinases.

### AMPKα2 binds BAY-3827 in a DFG-in inactive kinase conformation

**Fig. 2.**
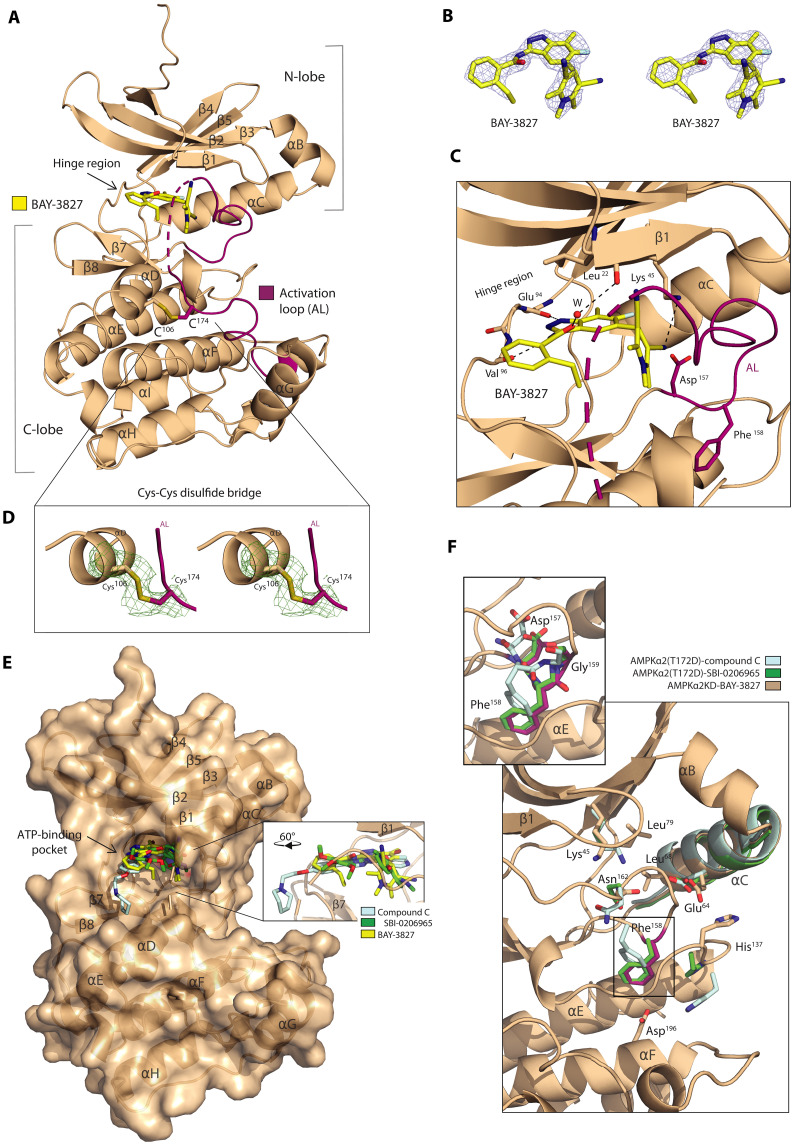
Structure of α2KD bound to BAY-3827. (**A**) Cocrystal structure of α2KD (shown as ribbons) with BAY-3827 (shown as yellow sticks). The activation loop (AL) segment is colored magenta. (**B**) Stereo-image of the 2Fo-Fc omit map of BAY-3827 shown at 1σ. (**C**) Magnified view of the BAY-3827 binding site with interactions indicated as gray dash lines. The side chains of Asp^157^ and Phe^158^ of the DFG motif are shown as sticks with Phe^158^ in an “FG down” conformation. A water molecule (W) is shown as a red sphere. A disulfide bond (Cys^106^-Cys^174^) is shown as sticks. (**D**) Stereo image of the Fo-Fc polder map (Phenix) of the indicated disulfide bond between Cys^106^ from αD and Cys^174^ from activation loop (AL). (**E**) Surface view of α2KD bound to BAY-3827 with superimposed compound C (light cyan, PDB:3AQV) and SBI-0206965 green, PDB:6BX6) inhibitors shown with sticks (*29*, *33*, *80*). (**F**) Zoomed-in view of the DFG motif superimposed with compound C (light cyan, PDB: 3AQV) and SBI-0206965 (green, PDB:6BX6), displaying the backbone (as ribbons) of α2KD bound to BAY-3827 showing key regulatory spine residues.

To understand the precise molecular mechanism behind the inhibition of AMPK by BAY-3827, the cocrystal structure of BAY-3827 with wild-type (WT) α2KD was obtained at a resolution of 2.5 Å (Table 1 and Fig. 2A). BAY-3827 binds into the conserved ATP-binding pocket between the N- and C-terminal lobes, as revealed by an unambiguous compound density map (Fig. 2B), binding with a dissociation constant (*K*_d_) value of 1.23 μM as measured by a spectral shift binding assay (fig. S2A). In this structure, the activation loop (highlighted in magenta) instead of swinging out in an active conformation (fig. S2B) (*42*), it hovers over the kinase core below the β1 sheet and interacts with the αD helix in the C-lobe through Cys^174^, constituting an inactive kinase conformation (Fig. 2, C and D). The indazole ring in BAY-3827 hydrogen bonds with hinge region residues, with a single bond formed with Glu^94^ and two with Val^96^ alongside two additional bonds made with N-lobe residues Leu^22^ and Lys^45^ (Fig. 2C and fig. S2C). Leu^22^ bonding to BAY-3827’s benzamide moiety is water-mediated and the conserved Lys^45^ in the β3 sheet interacts with the pyridine ring of BAY-3827 (Fig. 2C). Since BAY-3827 interacts directly with Lys^45^, the typical salt bridge with Glu^64^ in the αC helix is no longer possible, having a Lys-Glu distance of 11.7 Å (Table 2), and an αC helix out conformation (*43*). Residues that accommodate and engage in hydrophobic contacts with BAY-3827 include hinge region residues Tyr^95^ and Ser^97^, as well as N-lobe residues Gly^99^, Val^30^, and Asp^166^. In the C-lobe, residues Glu^100^, Gly^23^, Ile^77^, and Ala^43^ and the gatekeeper Met^93^ also contribute to ligand hydrophobic contacts, as well as Leu^146^, which faces the benzamine moiety of BAY-3827. Moreover, activation loop residues Asn^144^ and Ala^156^, alongside Asn^162^, Met^164^, and Ser^165^ face toward the pyridine ring of BAY-3827 and engage in hydrophobic contacts (fig. S2C), with BAY-3827 occupying a space in the ATP-binding pocket that does not extend further past gatekeeper Met^93^. Focusing on the DFG motif, Asp^157^ presents with an out conformation and the DFG motif Phe^158^ residue has shifted down toward the C-lobe compared to the kinase active state in AMPK (Fig. 2C and fig. S2B) (*42*). In the C-lobe, a key interaction occurs between αD helix Cys^106^ and activation loop residue Cys^174^, which forms a disulfide bridge (Fig. 2D). To confirm the relevance of this interaction to BAY-3827’s mechanism, we performed binding analyses in the presence or absence of dithiothreitol (DTT) alongside the phosphomimetic AMPKα2 (T172D)–α2KD mutant protein to evaluate the impact of this mutation on BAY-3827 binding affinity (fig. S2, D to G). The T172D mutation modestly reduced binding affinity compared to WT AMPKα2-KD (fig. S2F). Notably, treatment with 3 mM DTT markedly diminished BAY-3827 binding in the WT protein but had no substantial effect on the T172D- α2KD mutant (fig. S2, E to G), highlighting the role of disulfide bridge formation as well as Thr^172^ residue in stabilizing BAY-3827 binding. Superimposition of α2KD–BAY-3827 with available compound C and SBI-0206965 structures (*29*, *33*), showed a partial overlap with compound C’s binding site in the ATP-binding pocket. However, the binding space of BAY-3827 and SBI-0206965 on AMPK was shared, whereas compound C extended outside of this pocket (Fig. 2E and fig. S2J).

**Table 1.** Crystallography data collection and refinement statistics. Crystallography data collection for α2KD with BAY-3827. Statistics for the highest-resolution shell are shown in parentheses.

	α2 KD-BAY-3827
Wavelength	0.6199 Å
Resolution range	58.89 to 2.5 (2.56 to 2.50)
Space group	P 21 21 2
Unit cell	59.176 117.778 38.327 90 90 90
Total reflections	65,446 (6013)
Unique reflections	10,021 (890)
Multiplicity	6.5 (6.8)
Completeness (%)	99.9 (100.00)
Mean I/sigma(I)	9.07 (0.81)
Wilson B-factor	39.04
R-merge	0.074 (0.418)
R-meas	0.081 (0.454)
R-pim	0.0332 (0.174)
CC1/2	0.997 (0.944)
CC*	0.999 (0.973)
Reflections used in refinement	9799 (2397)
Reflections used for R-free	497 (119)
R-work	0.1975 (2433)
R-free	0.2350 (0.3053)
Number of nonhydrogen atoms	2256
Macromolecules	2142
Ligands	43
Solvent	71
Protein residues	267
RMS (bonds)	0.002
RMS (angles)	0.52
Ramachandran favored (%)	95.44
Ramachandran allowed (%)	3.04
Ramachandran outliers (%)	1.52
Rotamer outliers (%)	6.06
Clash score	5.52
Average B-factor	53.50
Macromolecules	53.90
Ligands	48.62
Solvent	44.43

**Table 2.** Atomic distance measurements to define kinase conformation. Distance measurements performed as previously described (*43*) in PyMOL (*80*).

Structures	D1: Leu^68^.CA-Phe^158^.A CZ	D2: Lys^45^.CA-Phe^158^.CZ	Lys^45^.CB-Glu^64^.CB
α2KD–BAY-3827	12.4 Å	18.4 Å	11.7 Å
AMPKα2 (T172D)–KD-compound C (PDB: 3AQV)	11.4 Å	16.6 Å	11.3 Å
AMPKα2 (T172D)–KD-SBI-0206965 (PDB: 6BX6)	13.1 Å	18.2 Å	11.8 Å

A recent classification of kinase structural states based on DFG motif conformation used the positions of the αC helix and the DFG Phe^158^ ring relative to Lys^45^ (D1 and D2 distances) (*43*). According to these measurements (Table 2), α2KD–BAY-3827 and AMPKα2(T172D) inhibitor structures of compound C and SBI-0206965 clustered together with other reported “DFG-in” structures (*43*). To further classify the DFG conformation of the α2KD–BAY-3827 structure, the Phi/Psi (Φ/Ψ) angles of the x-DFG-x motif residues were also measured (Table 3). However, we could not place the structure in an established cluster, suggesting that this structure belongs to a specific DFG-in conformation with an inactive kinase state that has not yet been defined (*43*). The SBI-0206965–bound structure (*33*) had nearly identical Φ/Ψ angle measurements compared to α2KD–BAY-3827 (Table 3) and a “Phe^158^-down” orientation toward the C-lobe compared to the active AMPK conformation (Fig. 2F and fig. S2B). In AMPKα2(T172D)–compound C (*29*), Phe^158^ also pointed down toward the C-lobe but adopted a different conformation than in BAY-3827/SBI-0206965 structures (Fig. 2F and Table 3).

**Table 3.** DFG motif Phe^158^ Phi, Psi (Φ, ψ) angle measurements. Measurements were conducted in PyMOL (*80*) retrieving available compound C (*29*) and SBI-0206965–bound (*33*) AMPKα2 (T172D) KD structures.

Structures	Ala^156^ (Φ, ψ)	Asp^157^ (Φ, ψ)	Phe^158^ (Φ, ψ)	Gly^159^ (Φ, ψ)	Leu^160^ (Φ, ψ)
α2KD–BAY-3827	−77.8°, 133.2°	−64.3°, 162.0°	−90.6°, 42.6°	−91.5°, 174.0°	−69.5°, −13.6°
AMPKα2 (T172D)–KD compound C (PDB: 3AQV)	−77.6°, 137.5°	−58.4°, 143.4°	−127.8°, 21.4°	−78.7°, −145.7°	−61.1°, −34.9°
AMPKα2 (T172D)–KD-SBI-0206965 (PDB: 6BX6)	−83.3°, 141.7°	−56.9°, 150.6°	−90.9°, 68.7°	−125.6°, 179.3°	−68.2°, −25.5°

In α2KD–BAY-3827, activation-loop residue Asn^162^ occupies a position below Lys^45^ in the N-lobe that is stabilized by hydrogen bonds with DFG residues Asp^157^ and Gly^159^ (Fig. 2F). This differs from its proximity to His^137^ in the C-lobe observed in active AMPK (fig. S2B) (*42*). As a result, Phe^158^ is displaced downward toward the αE helix, and His^137^ is pushed outward, away from its typical stack on Asp^196^, resulting in a broken hydrophobic spine characteristic of an inactive kinase (Fig. 2F and fig. S2K) (*44*). Notably, in BAY-3827–bound α2KD, His^137^ points toward the αC helix in the N-lobe, whereas in SBI-0206965– and compound C-bound structures, it faces the C-lobe (Fig. 2F and fig. S2K), highlighting two different ways of breaking the regulatory spine. Superimposing these three inhibitors also revealed a preserved αC helix conformation (Fig. 2F and fig. S2J). Overall, given their overlapping binding sites and DFG conformations (Fig. 2, E and F), the “FG-down” arrangement may represent a pivotal DFG-in transitional state in AMPK structural dynamics that is exploited by small molecule inhibitors.

### BAY-3827 inhibits different AMPK trimeric complexes with similar potency

**Fig. 3.**
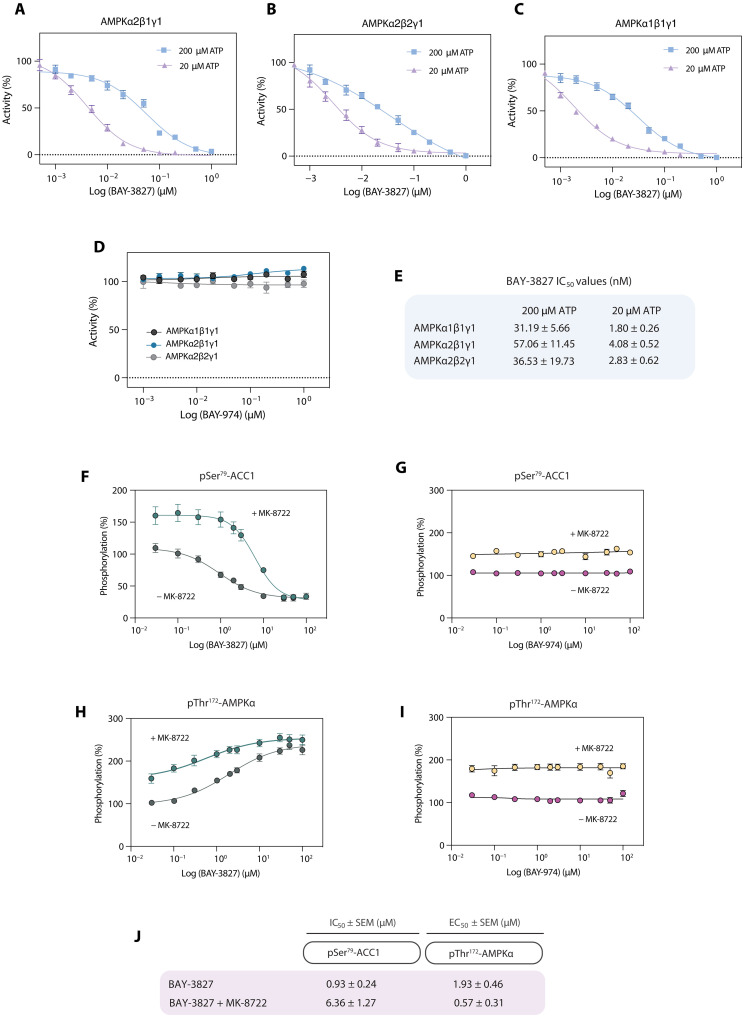
BAY-3827 and BAY-974 kinetics. (**A** to **C**) Inhibition of AMPK complexes in cell-free assays by BAY-3827 in high (200 μM) and low (20 μM) ATP concentrations and by (**D**) BAY-974 at 200 μM ATP. Data are *n* = 3 represented as means ± SEM. (**E**) IC_50_ values (nM) of BAY-3827 inhibition of AMPK complexes in different ATP conditions. (**F** and **G**) HTRF assays in U2OS cells measuring phosphorylation (%) of Ser^79^-ACC1 following BAY-3827 or BAY-974 treatment and phosphorylation (%) of (**H** and **I**) Thr^**172**^-AMPKα in the presence or absence of AMPK activator MK-8722 (10 μM). (**J**) Estimated IC_50_ and EC_50_ values (μM) shown, respectively, from (F) to (I). Data are means ± SEM with *n* = 3 technical replicates from three independent experiments.

Given the diverse assemblies that AMPK complexes can take depending on their subunit isoforms, the inhibitory action of BAY-3827 alongside BAY-974 compound control was studied using cell-free assays with different AMPK heterotrimeric complexes, namely α2β1γ1, α1β1γ1, and α2β2γ1 (Fig. 3, A to E). The α1β1γ1 and α2β2γ1 complexes showed similar IC_50_ values in response to BAY-3827 treatment when tested at high (200 μM) ATP concentrations, with IC_50_ values of 31.2 and 36.5 nM, respectively, in line with previous cell-free assay data with these two complexes (*38*), whereas the α2β1γ1 complex had a moderately higher IC_50_ of 57 nM. At lower ATP (20 μM), BAY-3827–treated α1β1γ1 and α2β2γ1 had values of 1.8 and 2.8 nM, with the α2β1γ1 complex having an IC_50_ value of 4.1 nM (Fig. 3E). Despite this modest difference, BAY-3827 had an inhibitory effect in the same order of magnitude and kinetics across all three tested AMPK complexes (Fig. 3, A to C), whereas BAY-974 showed no effect (Fig. 3, D and E). To examine the effect of different AMPK complexes on BAY-3827’s binding affinity, we also performed binding analyses on nonphosphorylated AMPKα2β2γ1 and AMPKα2β2γ3 complexes, which were obtained at a comparable purity level (fig. S2D). AMPKα2β2γ1 showed a slightly lower *K*_d_ value compared to the AMPKα2β2γ3 complex; however, both had a binding affinity found between an ~10 and 30 μM range (fig. S2, H and I). In addition, we also tested the effects of increasing concentrations of BAY-3827 and BAY-974 on selected kinases; ULK1 [known to be potently inhibited by SBI-0206965 (*36*)] and CaMKK2 (upstream kinase of AMPK), and confirmed no inhibitory effect (fig. S3, A and B).

### Cellular kinetics of BAY-3827 inhibitory action on AMPK

Before using BAY-3827 and the control compound BAY-974 in cellular functional studies, their dose-dependent effect on AMPK activity was investigated in U2OS cells, an osteosarcoma cell line. Homogeneous time-resolved fluorescence (HTRF) is a high-throughput fluorescence resonance energy transfer–based technique previously benchmarked as a sensitive and quantitative assay for detection of ACC1 Ser^79^ phosphorylation (*36*, *45*) and is an established surrogate marker for cellular AMPK activation. HTRF assays were performed following treatment with BAY-3827 and BAY-974 in the presence and absence of allosteric AMPK activator MK-8722 (*18*). Vehicle compared to MK-8722 control treatment showed an expected significant increase in both pSer^79^-ACC1 and pThr^172^-AMPK (fig. S3, C and D). ACC1 phosphorylation dose-dependently decreased upon BAY-3827 treatment, with an IC_50_ of 0.93 μM, which increased to a value of 6.36 μM in cells which were also treated with MK-8722. No inhibition was observed with BAY-974 even at the highest tested concentration of 100 μM (Fig. 3, F, G, and J). AMPK phosphorylation dose-dependently increased with BAY-3827, but not BAY-974, with a median effective concentration (EC_50_) of 1.93 and 0.57 μM in the presence of MK-8722 (Fig. 3, H to J). This observed increase in pAMPK is consistent with the reported effects of BAY-3827 on AMPK phosphorylation levels, possibly due to its protective effect against Thr^172^ dephosphorylation (*38*).

### BAY-3827 inhibits AMPK and blocks its suppressive effect on lipogenesis in hepatocytes

**Fig. 4.**
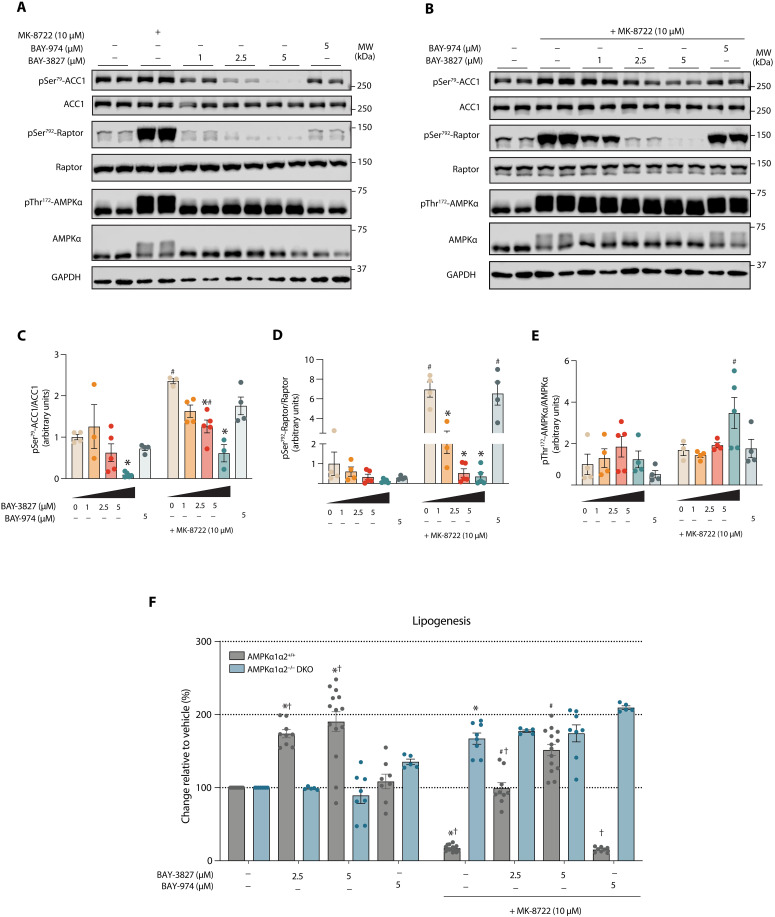
BAY-3827 but not BAY-974 dose-dependently reverses AMPK-inhibited lipogenesis in primary hepatocytes. (**A**) Representative blots of AMPKα1α2^+/+^ (control) hepatocytes treated with BAY-3827 and BAY-974 in basal conditions and (**B**) treated with 10 μM MK-8722 after compound treatment for 15 min, with selected antibodies describing AMPK signaling. (**C** to **E**) Calculated phosphorylation ratios following band intensity quantification of data from (A) and (B) showing means ± SEM from four separate experiments with *n* = 2 to 3 biological replicates subjected to a two-way analysis of variance (ANOVA) test with *P < 0.05 (vehicle versus treatments) and ^#^*P* < 0.05 (basal versus MK-8722). (**F**) Lipogenesis assay of control and AMPKα1α2^−/−^ DKO primary hepatocytes treated with BAY-3827 and inactive BAY-974, where *n* = 3 to 5 biological replicates from five separate experiments. A two-way ANOVA test was conducted, where *P < 0.05 (vehicle versus indicated treatment), #*P* < 0.05 (vehicle + MK-8722 versus in indicated treatment) and †*P* < 0.05 (control versus DKO treatments in basal and + MK-8722 conditions).

We next sought to establish whether BAY-3827 inhibited an AMPK-dependent process in a more physiologically relevant cell system. Mouse primary hepatocytes were treated with increasing doses of BAY-3827 with or without MK-8722 (10 μM). Immunoblot analysis revealed a dose-dependent reduction in phosphorylation of ACC1 and Raptor, with full inhibition in the presence of MK-8722 achieved at 5 and 2.5 μM. (Fig. 4, A to D). Increased pThr^172^AMPK levels were only significant under MK-8722–treated conditions at 5 μM (Fig. 4E). BAY-974 (5 μM) showed no effect on levels of pACC1, pRaptor, or pAMPK with or without MK-8722 (Fig. 4, C to E).

One of the best-characterized physiological consequences of AMPK activation in hepatocytes is the suppression of fatty acid synthesis through phosphorylation of Ser^79^ on ACC1 (*46*, *47*). Mouse primary hepatocytes derived from AMPKα1α2^+/+^ control and liver-specific AMPKα1α2^−/−^ DKO mice were treated with BAY-3827 (2.5 and 5 μM) with or without MK-8722 (10 μM), and the [^14^C]-acetate incorporation into fatty acids was assessed. In control hepatocytes, BAY-3827 alone significantly stimulated (~70 to 90%) lipogenesis, while MK-8722 alone robustly suppressed it (>80%) (Fig. 4F). BAY-3827 dose-dependently rescued the MK-8722–mediated inhibition of lipogenesis. In AMPKα1α2^−/−^ DKO cells, basal lipogenesis was significantly higher than vehicle-treated, but similar to BAY-3827–treated control hepatocytes. In AMPKα1α2^−/−^ DKO hepatocytes, BAY-3827 and MK-8722 treatment had no effect on lipogenesis. In addition, BAY-974 treatment (5 μM) had no effect on lipogenesis in both vehicle- and MK-8722–treated conditions (Fig. 4F). We also tested whether compound C was able to rescue MK-8722–mediated inhibition of hepatic lipogenesis and observed that it only negligibly (10%) restored it at 25 μM, confirming its low potency in cells (fig. S3E). These results establish BAY-3827 as a potent and selective compound to probe the AMPK-dependent regulation of lipogenesis in response to pharmacological and genetic modulations in hepatocytes.

We have previously reported that SBI-0206965 not only inhibits AMPK-related kinases (NUAK1 and MARK3/4) equally or more potently than AMPK or ULK1 but also inhibits critical cellular functions such as basal and insulin-stimulated glucose uptake in adipocytes and skeletal muscle (*36*). We treated mouse primary adipocytes and isolated mouse skeletal muscle ex vivo with varying doses of BAY-3827 with or without MK-8722 or insulin (fig. S3, F to K). We observed a dose-dependent reduction in Raptor phosphorylation in response to BAY-3827 irrespective of MK-8722’s presence (fig. S3, F and G). Both basal and insulin-stimulated phosphorylation of Akt and TBC1D4, as well as glucose uptake were not significantly affected in adipocytes (fig. S3, H to J). In ex vivo skeletal muscle, BAY-3827 dose-dependently inhibited MK-8722–stimulated glucose uptake and showed a full inhibition at 5 μM (fig. S3K). BAY-3827 attenuated insulin-stimulated (AMPK-independent) glucose uptake in ex vivo skeletal muscle, indicating a potential off-target effect.

### BAY-3827 diminishes drug-stimulated AMPK-dependent gene expression

**Fig. 5.**
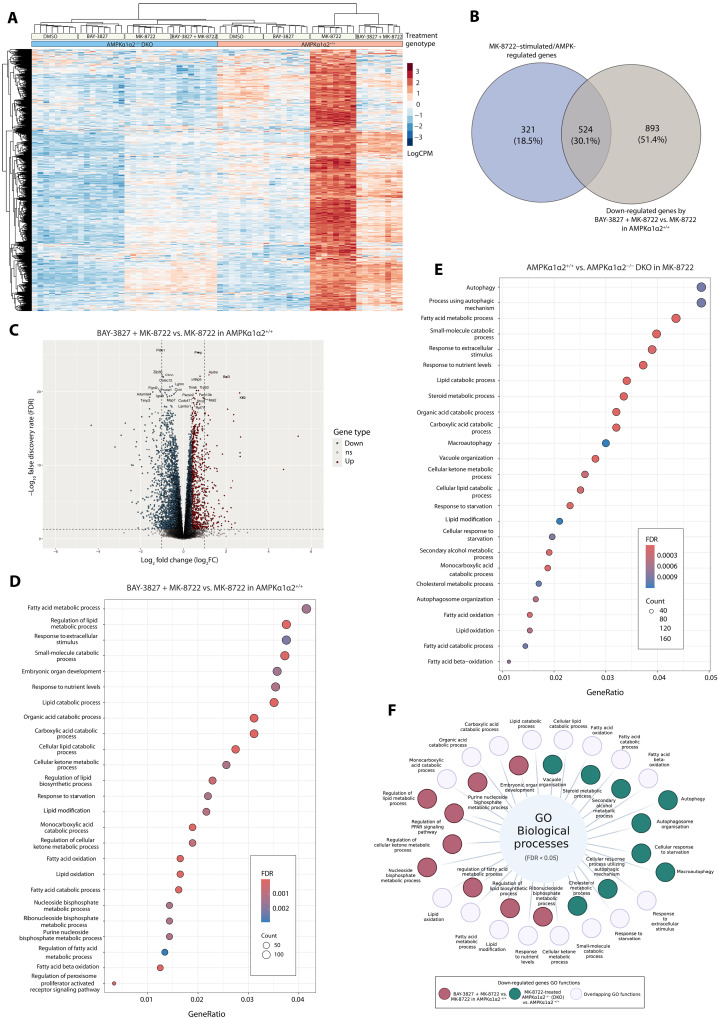
Unbiased transcriptome analysis of BAY-3827 ± MK-8722–treated AMPKα1α2^+/+^ (control) and AMPKα1α2^−/−^ DKO primary hepatocytes. (**A**) Heatmap of gene expression of 845 significant genes that are stimulated by MK-8722 and are AMPK-dependent. (**B**) Venn diagram showing the overlap between genes with a significantly reduced expression when treated with BAY-3827 in MK-8722–stimulated control cells compared to MK-8722–stimulated/AMPK-regulated genes, with a 30.1% overlap. (**C**) Volcano plot showing top significant (FDR < 0.05) up-regulated (FC ≥ 1.3; red), down-regulated (FC < −1.3; blue), and nonsignificant (ns) regulated genes by BAY-3827 in MK-8722–stimulated control cells compared to MK-8722 treatment alone. (**D**) GO enrichment analysis of biological processes of down-regulated significant genes (FDR < 0.05) by BAY-3827 + MK-8722 treatment and (**E**) by the loss of AMPKα1α2 in MK-8722–treated cells. (**F**) Schematic of the down-regulated GO processes showing the functional overlap between MK-8722–stimulated AMPKα1α2^−/−^ DKO versus control cells and BAY-3827 + MK-8722 treated versus MK-8722 in control cells. Created in BioRender. C. Fraguas (2025) (https://BioRender.com/j60q096).

To further examine the effect of BAY-3827 on AMPK signaling and gene expression in an unbiased manner, we treated control and AMPKα1α2^−/−^ DKO mouse primary hepatocytes with 5 μM BAY-3827 in the presence or absence of MK-8722 and performed RNA sequencing (RNA-seq) analysis. We focused on 1841 genes that were significantly up-regulated in MK-8722 treatment compared to basal conditions (vehicle-treated) in control hepatocytes. To confidently define them as AMPK-dependent genes, we selected genes that were significantly down-regulated in MK-8722–treated AMPKα1α2^−/−^ DKO cells compared to control. This yielded 845 genes that were both MK-8722 stimulated and AMPK dependent, with their expression visualized in a heatmap where the MK-8722–stimulated gene expression in control cells was almost completely abolished in AMPKα1α2^−/−^ DKO cells (Fig. 5A). In hepatocytes treated with BAY-3827 and MK-8722, approximately 30% of MK-stimulated AMPK genes were down-regulated (524 genes of 845 predefined MK-8722-AMPK genes) (Fig. 5, A and B, and fig. S4A). In control hepatocytes treated with BAY-3827 and MK-8722, 2511 significant genes were differentially expressed compared to MK-8722, with 61.2% (1539 genes) down-regulated by BAY-3827 treatment in AMPK-activated conditions. Top significant [fold change (FC) < −1.3; false discovery rate (FDR) < 0.05] down-regulated genes included *PLCE1*, *ZFP36*, *FIGNL2*, *ADAMTSL4*, and *TIMP3* (Fig. 5C and table S3). In MK-8722–treated AMPKα1α2^−/−^ DKO cells compared to control, *ADAMTSL4* and *ZFP36* were also among the top down-regulated genes (fig. S4B).

Gene ontology (GO) enrichment analysis of biological processes was performed to query the gene functions of those down-regulated when AMPK is inactivated either via genetic (AMPKα1α2^−/−^ DKO) or pharmacological (BAY-3827) means, in the presence of MK-8722 (Fig. 5, D to F). The top 25 categories (FDR < 0.05) of down-regulated genes in MK-8722–stimulated AMPKα1α2^−/−^ DKO and BAY-3827–treated control cells revealed a large overlap in known AMPK-regulated functions. These functions, which BAY-3827 also inhibited, included lipid oxidation and modulation, organic acid catabolic processes, and response to nutrient levels and starvation (Fig. 5F). Specific functions of BAY-3827–down-regulated genes in MK-8722–stimulated control cells included nucleoside biphosphate and ketone metabolism, as well as the regulation of peroxisome proliferator–activated receptor signaling pathway (Fig. 5, D and F). Uniquely down-regulated functions in MK-8722–induced AMPKα1α2^−/−^ DKO compared to control cells included autophagy-related functions as well as steroid and secondary alcohol metabolic processes (fig. 5, E and F). Treatment of BAY-3827 in the presence of MK-8722 resulted in a significant decrease of AMPK-dependent genes involving lipid metabolic genes, which in MK-8722 alone conditions are normally up-regulated, and promote lipid oxidation and other catabolic processes (fig. S4C). AMPK-dependent genes that were down-regulated by BAY-3827 treatment in MK-8722–treated control cells included fatty acid metabolism genes *PLIN5*, *LPIN2*, *EHHADH*, *AVPR1A*, and *ADORA2B* as well as liver metabolism regulator *SDS* and histidine catabolism gene *HAL*, establishing the role of BAY-3827 in suppressing the expression of AMPK-regulated lipid oxidative genes (Table 4 and fig. S4C). Apart from lipid metabolic genes, BAY-3827 also reduced the expression of genes involved in transcription regulation, such as *SP1,* which encodes a transcription factor proposed to be AMPK regulated (*48*). Another example includes the *TBCD15* and *TBCD17* genes with roles in autophagy, with encoded Rab-GTPase activating protein TBCD17 recently described as an AMPK substrate (table S3) (*49*, *50*).

**Table 4.** Examples of AMPK-dependent genes down-regulated in BAY-3827 in MK-8722–stimulated control hepatocytes. The complete list of differentially expressed genes in BAY-3827 + MK-8722 versus MK-8722 in control hepatocytes is recorded in table S3.

Gene	Name	LogFC	FDR
*LIPE*	Lipase, hormone sensitive	−0.28159	0.004450324
*PFKFB2*	6-phosphofructo-2-kinase/fructose-2,6-biphosphatase 2	−0.54205	3.09 × 10^−08^
*HMGCR*	3-hydroxy-3-methylglutaryl–CoA reductase	−0.16208	0.00451032
*PLIN5*	Perilipin 5	−1.29957	5.66092 × 10^−18^
*LPIN2*	Lipin 2	−0.52774	1.10 × 10^−08^
*PLIN4*	Perilipin 4	−1.23494	4.01982 × 10^−14^
*SP1*	trans-acting transcription factor 1	−0.43973	7.4359 × 10^−13^
*HPGD*	Hydroxyprostaglandin dehydrogenase 15 (NAD)	−1.745	5.54633 × 10^−14^
*EHHADH*	Enoyl-CoA, hydratase/3-hydroxyacyl CoA dehydrogenase	−1.5955	2.79131 × 10^−17^
*AVPR1A*	Arginine vasopressin receptor 1A	−1.70797	7.31686 × 10^−13^
*ADORA2B*	Adenosine A2b receptor	−1.3062	7.77511 × 10^−11^
*TBC1D15*	TBC1 domain family, member 15	−0.48954	1.47094 × 10^−15^
*TBC1D17*	TBC1 domain family, member 17	−0.559835768	9.72654 × 10^−17^
*HAL*	Histidine ammonia lyase	−1.34842	1.52157 × 10^−09^

In addition, given AMPK’s established roles in mitochondrial biogenesis, to validate our dataset, we visualized the normalized counts of the *PPARGC1A* gene across sample treatments with vehicle [dimethyl sulfoxide (DMSO)], BAY-3827, MK-8722, or BAY-3827 + MK-8722 in control and AMPKα1α2^−/−^ DKO genotypes. We observed a significant count increase in the MK-8722 condition compared to vehicle in control cells but not in AMPKα1α2^−/−^ DKO cells, and, furthermore, BAY-3827 + MK-8722 significantly reduced gene counts compared to MK-8722 treatment alone in AMPKα1α2^+/+^ cells (fig. S4D). Moreover, *PPARGC1A* was among the top significant down-regulated genes by BAY-3827 + MK-8722 compared to MK-8722 treatment alone in control hepatocytes, with a *P*-adjusted value of 6.5083 × 10^−05^ (table S3). Together, these data indicate that BAY-3827 inhibits AMPK-induced increase in *PPARGC1A*, a key regulator of mitochondrial biogenesis.

Yang *et al.* (*51*) recently described the transcriptional profiles of RSK1- and RSK2 knockout glioma-based cell lines. We found proposed RSK-regulated genes that were down-regulated by BAY-3827 in MK-8722–stimulated control cells. These included *STAT5B* and *CALCOCO1* (RSK2) as well as *NEK2*, *DSN1*, *MASTL*, and *SPC25* (RSK1) among others (fig. S4E and table S3) (*51*).

### BAY-3827 inhibits RSK in cells

Although previous (*37*) and current in vitro (cell-free) data (Fig. 1A) have shown that BAY-3827 potently inhibits RSK isoforms, this has not been verified in a cellular context. We treated serum-starved human embryonic kidney–293 (HEK293) cells with varying concentrations of BAY-3827 or the RSK inhibitor BI-D1870 (*52*) with or without phorbol 12-myristate 13-acetate (PMA), an activator of extracellular signal–regulated kinase (ERK) 1/2 and RSK isoforms, and assessed phosphorylation of glycogen synthase kinase (GSK) 3α/β as a surrogate marker for cellular RSK activity (*52*). Since phosphorylation of GSK3α/β is also regulated by Akt, we included the Akt inhibitor MK-2206 to specifically assess RSK-mediated (i.e., PMA-stimulated) phosphorylation of GSK3α/β. As anticipated, BAY-3827 treatment resulted in a dose-dependent reduction of ACC1 phosphorylation, while BI-D1870 treatment had no effect (fig. S5, A and B). MK-2206 treatment reduced basal pGSK3αβ, and PMA robustly increased pERK1/2 and pGSK3αβ levels under MK-2206–treated conditions (fig. S5, C and D). PMA-stimulated pGSK3αβ (in the presence of MK-2206) was inhibited to the same extent by 10 μM BAY-3827 and BI-D1870 (fig. S5D), indicating that BAY-3827 has an inhibitory potency similar to BI-D1870 in suppressing RSK in HEK293 cells.

To explore the molecular basis for RSK inhibition by BAY-3827, we conducted an in silico superimposition of the BAY-3827–α2KD structure with available structures of RSK2 N-terminal (NT) KD in complex with BI-D1870 and SL0101 RSK inhibitors (*53*, *54*). Both compounds had overlapping binding sites with BAY-3827–α2KD as well as conserved interacting residues with AMPK (fig. S5, E and F). In addition, both inhibitors make hydrogen bond contacts with hinge region residues Glu^94^ (Asp^148^ in RSK2) and Val^96^ (Leu^150^ in RSK2) where the latter in SL0101 is water mediated (fig. S5, E and F). This suggests that the effect of BAY-3827 on RSK inhibition is likely derived from these conserved structural features as well as from the compound binding mode.

## DISCUSSION

Here, we provide the structure-based mechanism of action of BAY-3827 in the inhibition of AMPK, describing distinct and previously unidentified molecular features. We also have comprehensively evaluated the cellular potency and selectivity (i.e., on/off-target effects) of BAY-3827 alongside its inactive control BAY-974 in signaling, transcriptomic, and metabolic output levels by using control and AMPKα1α2^−/−^ DKO primary hepatocytes. Consistent with a previous study (*37*), we show that BAY-3827 potently inhibits the AGC group kinases RSK1 and RSK2 in cell-free assays, albeit to a lower extent than AMPK, as shown across a panel of 140 human protein kinases (Fig. 1A and table S1). Compound C and SBI-0206965, based on their reported selectivity profiles (*31*, *33*, *36*) did not affect the RSK family, although other compounds reported to inhibit AMPK have also shown a preference for this kinase family. For instance, the ATP-competitive PAK4 inhibitor PF-03758309 has been reported to potently inhibit AMPK with an IC_50_ of 1 nM in a cell-free assay. PF-03758309 is also known to potently inhibit RSK1 and RSK2 with an IC_50_ of 2 nM (*55*), where in silico docking to AMPK KD predicted three hydrogen bonding interactions in the hinge region with Glu^94^ and Val^96^ residues (*56*) in the same fashion as in the current α2KD–BAY-3827 structure. RSK inhibitors BI-D1870 and SL0101 both make contacts with these two hinge residues, which correspond to Asp^148^ and Leu^150^ in the reported RSK2 structures (fig. S5, E and F) (*53*, *54*), sharing some interaction residues with BAY-3827’s binding mode, pointing to a structural basis for RSK inhibition. In line with this notion, and with results from current and previous (*37*) cell-free assays, our cellular studies revealed that BAY-3827 treatment inhibited PMA-stimulated RSK activation to the same extent as BI-D1870 (fig. S5, A and D). Thus, RSK isoforms are validated off-targets of BAY-3827, and RSK inhibitors could be included as an additional control alongside BAY-974 (which does not inhibit RSKs) to mitigate the involvement of RSK isoforms.

The cocrystal structure of BAY-3827 with inactive WT α2KD reveals the molecular mechanism allowing compound inhibition, with past inhibitor structures (compound C, SBI-0206965) were instead obtained using a phosphomimetic KD; AMPKα2 (T172D). BAY-3827 binds the ATP-binding pocket at a site that overlaps with SBI-0206965, consistent with past biochemical data deeming both SBI-0206965 and BAY-3827 compounds as mixed-type inhibitors (*33**,* *38*). Superimposition of α2KD–BAY-3827 with SBI-0206965– and compound C-bound structures revealed an activation loop conformation characteristic of an inactive kinase, with a conserved αC helix-out state (Fig. 2F). However, in the BAY-3827–bound conformation, activation loop residue Asn^162^ is found in a position that disrupts the AMPK regulatory spine and where His^137^ is displaced (fig. S2K). Phe^158^ residue faces down toward the C-lobe compared to the active state, with a conserved conformation with the SBI-0206965–bound structure (Fig. 2F and fig. S2B), which is likely due to their shared binding sites.

The AMPKα2 (T172D)–SBI-0206965 structure (*33*) was first described as a type IIB structure in a noncanonical DFG-out conformation; however, distance and Φ/Ψ angle measurements following an updated kinase nomenclature (*43*) revealed that it clusters together with the BAY-3827–bound structure with other DFG-in states (Table 2). DFG-in structures are often associated with active kinases; however, they are also found in inactive states (*43*, *44*, *57*). In these structures, an inactive state is achieved either due to αC helix movement or activation loop conformation (*44*). In the case of BAY-3827 binding, we find that both factors contribute to this, mainly by disrupting the regulatory spine that is otherwise uninterrupted in an active kinase. Given the conserved orientation of the Phe^158^ ring toward the C-lobe, we propose that an FG-down conformation, known to be associated with DFG-in states (*57*), might be important in AMPK structural dynamics upon compound-based inhibition. We further observed a cysteine disulfide bridge unique to the α2KD–BAY-3827 structure between the αD Cys^106^ and activation loop Cys^174^, which we hypothesize helps to stabilize activation loop conformation in an unproductive kinase state upon BAY-3827 binding (Fig. 2D). This interaction has not been observed previously in available α2KD-inhibitor co-structures, and Cys^174^ alongside the close-by Phe^102^, which is displaced as a result of this interaction, were among the top residues predicted as highly conserved in BAY-3827–inhibited kinases but were lowly conserved in noninhibited kinases (Fig. 1, E and F, and table S2), suggesting that the A-loop stabilization is a contributing factor to BAY-3827 inhibition of AMPK. Although Cys^174^ is a highly conserved activation loop residue across CAMK and AGC Ser/Thr kinases (*58*), our data indicate that BAY-3827’s kinase selectivity is mediated by a cohort of residues (table S2 and fig. S1C), and, hence, selectivity is not solely imparted by the disulfide bridge, but rather, this interaction points to a stabilizing feature that we find contributes to BAY-3827’s binding affinity (fig. S2, E to G). Previous crystal structure studies with other kinases have shown intramolecular disulfides in the activation segment region (*59*); however, the Akt2 KD structure lacked solved residues between the two interacting cysteines (Cys^297^-Cys^311^), which was not the case in the AMPK α2KD–BAY-3827 structure, and Cys^297^ is not conserved in AMPK. Cells are known to maintain high reducing environments, raising the possibility that the observed disulfide Cys^106^-Cys^174^ bridge could be affected under such conditions. To investigate this, we studied BAY-3827–binding affinity in the presence or absence of 3 mM DTT in both WT and T172D AMPKα2-KD and found that a reducing environment lowered BAY-3827’s binding affinity in WT but was unaffected in T172D-α2KD compared to no-DTT (fig. S2, E to G).

We also report that BAY-3827 increases AMPK-Thr^172^ phosphorylation in U2OS cells, mouse primary hepatocytes, and mouse primary adipocytes (Fig. 4 and fig. S3). It was recently suggested that the binding of BAY-3827 (and also SBI-0206965) (*33*, *38*) induces a protective effect toward phosphatase-meditated dephosphorylation of Thr^172^ (*38*). Given the dose-dependent reduction in AMPK downstream target phosphorylation such as ACC1 and Raptor, we find that it does not reflect functional activation status of AMPK. Rather, BAY-3827 likely stabilizes an activation loop conformation that contributes to phosphatase access shielding as well as an inactive conformation for substrate engagement.

We have demonstrated that BAY-3827 is capable of fully reversing MK-8722–induced inhibition of fatty acid synthesis and down-regulating ~30% of MK-8722–stimulated AMPK-regulated genes (Fig. 4B) involved in known AMPK-governing functions such as fatty acid oxidation and lipid catabolism (Fig. 4, D to F). Lemos *et al.* (*37*) treated androgen-dependent prostate cancer cells (LNCaP) with BAY-3827 and reported several down-regulated genes involved in lipid metabolism that we also observed were down-regulated by BAY-3827 treatment in MK-8722–stimulated control primary hepatocytes, namely *LIPE*, *PFKFB2*, and *HMGCR*. Our analysis also uncovered other lipid metabolism–related genes with their expression dependent on the presence of functional AMPK, which were significantly down-regulated following the treatment with BAY-3827 when AMPK had been pharmacologically activated (fig. S4, A and C). The RSK family encompasses a conserved group of serine/threonine protein kinases formed by human isoforms RSK1, RSK2, RSK3, and RSK4 alongside RSK-like mitogen- and stress-activated kinases 1 (MSK1) and 2 (MSK2), with key functions in cellular growth and proliferation such as cell survival promotion, protein translation, and ribosome biogenesis (*60*). Because of their growth-promoting roles alongside ensuring cell survival, RSKs are also established targets in anticancer therapies and are associated with increased expression in certain types of cancer (*61*). We searched for RSK-regulated genes (*51*) in our dataset and found that BAY-3827 in MK-8722–stimulated control hepatocytes significantly down-regulated some RSK-proposed genes, with a larger number of previously defined RSK1 genes present in our dataset compared to RSK2 (fig. S4D). This is consistent with our data showing higher inhibition of RSK1 compared to RSK2 isoform (table S1) and warrants further investigations.

As emerging roles and therapeutic implications of AMPK arise, it is critical to perform robust proof-of-concept experiments with multifaceted approaches. Although there are well-established preclinical genetic models (*62*, *63*) and allosteric activators (*9*), availability of potent and selective inhibitors is largely limited. Here, we have delineated the molecular basis of BAY-3827’s inhibition of AMPK and robustly characterized AMPK-regulated transcriptomic and metabolic functions, alongside building on the knowledge of AMPK-specific structural features, which will facilitate future efforts in inhibitor discovery and rational drug design. We have also described the current limitations of BAY-3827 (e.g., potent inhibition of RSK) and have extensively characterized BAY-974 across different cell lines and concentration range (0.03 to 100 μM), deeming this compound as a good experimental control for BAY-3827 use. In addition, we propose the context-dependent use of RSK inhibitors. In summary, BAY-3827 offers an increased potency and selectivity compared to prevalently used inhibitors (e.g., compound C, SBI-0206965), making it the preferred choice for cell-based studies. Still, potent inhibition of RSK isoforms and poor pharmacokinetic properties limit its in vivo use, and these issues will need to be addressed in future efforts to identify AMPK inhibitors.

## MATERIALS AND METHODS

### Animals

Animal experiments were conducted in accordance with the European directive 2010/63/EU of the European Parliament and of the Council of the protection of animals used for scientific purposes. Ethical approval was given by the Danish Animal Experiments Inspectorate (license number #2021-15-0201-01059). For primary hepatocytes (signaling studies) and ex vivo skeletal muscle incubation experiments, WT C57BL/ 6N Tac male mice (10 to 16 weeks old) were obtained from Taconic Biosciences and housed in the animal facility at the Faculty of Health and Medical Sciences (University of Copenhagen). Generation of AMPKα1α2^lox/lox^ and liver-specific AMPKα1α2 DKO (C57BL/6 background) for lipogenesis and RNA-seq experiments were described previously (*46*). Animals were anesthetized with Avertin [stock of tribromoethanol (1 g/ml; #T48402, Merck MilliporeSigma)] in 2-methyl-2-butanol (#152463, Merck MilliporeSigma), diluted 1:20 in saline, and dosed at 10 μl/g body weight via intraperitoneal injection before the procedure.

For adipocyte experiments, animal experiments were approved by the Regional Ethical Committee on Animal Experiments in Malmö/Lund (approval number 5.8.18–19111/2023). WT C57BL6/J male mice (10 to 11 weeks old) were obtained from Taconic Biosciences and kept in the animal facility at the Biomedical Centre (Lund University, Sweden). All the animals were kept and maintained according to local regulations under a light/dark cycle of 12 hours, 22° ± 1°C, and had free access to water and standard chow diet.

### Mouse primary hepatocyte isolation and lipogenesis assay

Mouse hepatocytes were isolated by collagenase perfusion as previously described (*47*). Hepatocytes were seeded in medium Eagle-199 (MEM-199) (#41150, Thermo Fisher Scientific) containing penicillin G (100 U/ml), streptomycin (100 μg/ml), and 10% (v/v) fetal bovine serum (FBS). Hepatocytes were left for attachment (3 to 4 hours) and serum-starved overnight at 37°C with 5% CO_2_ in MEM-199 supplemented with penicillin G (100 U/ml), streptomycin (100 μg/ml), 10 nM insulin, and 100 nM dexamethasone. Isolated hepatocytes were seeded and after 16 to 18 hours incubated for 3 hours for signaling studies as previously described (*46*) or were incubated with MEM-199 media supplemented with 0.6 μCi/ml [^14^C]-acetate (PerkinElmer, #NEC084H001MC) in the presence of BAY-3827 (MedChemExpress, #HY-112083) or inactive BAY-974 (donated probe from the Structural Genomics Consortium; SGC-Frankfurt) cotreated with MK-8722 (MedChemExpress, HY-111363) or vehicle (0.1% DMSO), respectively. Cells were then harvested in 0.5 ml of phosphate-buffered saline (PBS), transferred into 1 ml of 40% KOH and 2 ml of methanol followed by 1-hour incubation at 80°C. Lipids were saponified by acidifying the samples in 37% HCl and extracted with petroleum ether. Extracts were allowed to evaporate to dryness and then dissolved in Ultima Gold scintillation fluid for determination of [^14^C]-acetate incorporation into lipids.

### Mouse primary adipocyte isolation, compound treatment, and glucose uptake

Epidydimal adipose tissue excised from mice was digested with collagenase (1 mg/ml) in Krebs-Ringer medium (120 mM NaCl, 4.7 mM KCl, 1.2 mM KH_2_PO_4_, and 1.2 mM MgSO_4_) containing 25 mM Hepes (pH 7.4), 200 nM adenosine, 2 mM glucose, and 3% (w/v) bovine serum albumin (BSA) (KRH buffer) at 37°C in a shaking incubator. Adipocytes were isolated by filtering and washing in KRH buffer. For preparation of protein extracts for Western blot, adipocyte suspensions [0.7 to 1 ml of 10% (v/v) cells] were preincubated with BAY-3827 or BAY-974 compounds or 0.1% DMSO for 1 hour at 37°C in a shaking (80 rpm) water bath followed by either 1 hour of incubation with 10 μM MK-8722 or 30 min with 0.1 nM insulin (Novo Nordisk) in KRH buffer, as indicated in the figure legends. Cells were then washed in KRH buffer without BSA and lysed in 50 mM tris-HCl (pH 7.5), 0.27 M sucrose, 1 mM EDTA, 1 mM EGTA, 5 mM sodium pyrophosphate, 1 mM sodium orthovanadate, 50 mM sodium fluoride, 1 mM dithiothreitol, 1% (w/v) NP-40, and complete protease inhibitor cocktail (one tablet/50 ml) included in lysis buffer. Lysates were centrifuged at 13,000*g* for 15 min (4°C), and supernatant protein concentration was determined by the Bradford assay. Freshly isolated mouse adipocytes were washed in glucose-free buffer containing 30 mM Hepes (pH 7.4), 120 mM NaCl, 4 mM KH_2_OPO_4_, 1 mM MgSO_4_, 0.75 mM CaCl_2_, 10 mM NaHCO_3_, 200 nM adenosine, and 3% (w/v) BSA (KRBH buffer). Adipocyte suspensions [400 μl of 3.75% (v/v) cells] were preincubated with BAY compounds or 0.1% DMSO for 1 hour at 37°C in a shaking (80 rpm) water bath, before being stimulated with 0.1 insulin (Novo Nordisk) or 10 μM cytochalasin B (Sigma-Aldrich) for 30 min. Subsequently, 100 μl KRBH buffer containing 0.25 μl (0.025 μCi) [^14^C]-glucose (275 mCi/mmol glucose; final glucose concentration = 0.18 μM) was added, and the cells were incubated for a further 30 min. Reactions were stopped by aliquoting 300 μl of the total 500-μl adipocyte suspension to Beckman microtubes containing 75 μl of dinonylphtalate. The adipocyte suspension was centrifuged at 6000*g* and frozen at −20°C before adipocytes were collected and subjected to scintillation counting. The assay was performed in triplicate for each condition.

### Ex vivo skeletal muscle incubation for signaling and glucose uptake assays

Extensor digitorum longus muscles were rapidly dissected and mounted in oxygenated (95% O_2_ and 5% CO_2_), and warmed (30°C) Krebs Ringer buffer (KRB) containing 117 mM NaCl, 4.7 mM KCl, 2.5 mM CaCl_2_*2H_2_O, 1.2 mM KH_2_PO_4_, 1.2 mM MgSO_4_*7H_2_O, 24.6 mM NaHCO_3_ (pH 7.5) supplemented with 2 mM pyruvate (Sigma-Aldrich, P-2256) in the presence of the BAY-3827 or vehicle (0.1% DMSO) for 30 min. The muscles were then coincubated for 50 min with BAY-3827 and ± 10 μM MK-8722 or 100 nM insulin (Novo Nordisk).The muscles were further incubated for 10 min in the presence of vehicle, compounds, or insulin in glucose uptake buffer composed of KRB buffer containing 1.5 μCi/ml [^3^H]-2-deoxy-d-glucose (NET549005MC lot #2644198 2DG H3), 1 mM 2-deoxy-d-glucose, 0.45 μCi/ml [^14^C]-mannitol (NEC852250UC lot #2741082 D-Mannitol C14), and 7 mM mannitol. At the end of incubation period, the muscles were frozen in liquid nitrogen and subsequently processed for glucose uptake [as described in (*64*)] and Western blot analysis.

### Kinase selectivity assays

BAY-3827 and BAY-974 were assayed against a 140-human kinase panel at 0.1 μM and at 1 μM (BAY-3827) concentration in a [γ-^33^P] ATP–based radioactive filter binding assay (*31*, *65*) setup in duplicates with reported mean remaining kinase percentage activity alongside SD (table S1). Assays were performed at either 5, 20, or 50 μM ATP to be at or below the Michaelis constant for ATP for each enzyme and conducted by the services offered by the International Centre for Kinase Profiling at MRC Protein Phosphorylation and Ubiquitylation Unit, University of Dundee (https://www.kinase-screen.mrc.ac.uk/services/premier-screen).

### Protein production and kinase assays

Heterotrimeric human AMPK FLAG- α1β1γ1, FLAG- α2β1γ1, and FLAG- α2β2γ1, as well as human FLAG-CaMKK2 and FLAG-ULK1, were produced in mammalian HEK293 cells as described (*66*, *67*). For AMPK expression, the cells were triply transfected at 60% confluency using FuGene HD (Roche Applied Science) and 1 μg of pcDNA3 plasmid expression constructs for AMPK Flag-α1, β1-Myc, β2-Myc, and HA-γ1. For CaMKK2 expression, the cells were transfected with 1 μg of pcDNA3 Flag-CaMKK2 plasmid. For ULK1 expression, the cells were transfected with 2 mg of pcDNA3 FLAG-ULK1 plasmid. After 48 hours, the transfected cells were harvested by rinsing with ice-cold PBS, followed by rapid lysis using 500 μl of lysis buffer. AMPK, CaMKK2, and ULK1 kinase activities were determined by phosphorylation of synthetic peptide substrates: SAMS (HMRSAMSGLHLVKRR), CaMKKtide (LSNLYHQGKFLQTFCGAPLYRRR), and S108tide. (KLPLTRSHNNFVARRR), respectively. Briefly, recombinant AMPK, CaMKK2, or ULK1 were immunoprecipitated from 10 μg of transfected cultured cell lysate using 10 μl of anti-FLAG M2 agarose beads [50% (v/v)] (Merck MilliporeSigma), washed, and then added to a 25-μl reaction containing assay buffer [50 mM Hepes-NaOH (pH 7.4), 1 mM DTT, and 0.02% (v/v) Brij-35], 200 μM synthetic peptide substrate (SAMS, CaMKKtide, or S108tide), 200 or 20 μM [γ-^32^P]-ATP (PerkinElmer), 5 mM MgCl_2_, in the presence of 0 to 1 μM BAY-3827 or BAY-974. Reactions were performed at 30°C and terminated after 10 min by spotting 15 μl onto phosphocellulose paper. Radioactivity was quantified by liquid scintillation counting. Data visualization and nonlinear regression fitting were performed in GraphPad Prism v10.

### Sequence conservation of BAY-3827 inhibited and noninhibited kinases

Human KD sequences of AMPKα1, AMPKα2, RSK1, RSK2, MSK1, NUAK1, MELK, BRSK2, ABL1, PIM3, INSR, IRAK4, WNK1, PIM2, IKKβ, CDK2, TTBK1, and TAK1 were retrieved from KinBase (http://kinase.com/web/current/kinbase) (*39*). For RSK1, RSK2, and MSK1 kinases, the two KDs were included in the analysis. A multiple sequence alignment of BAY-3827–inhibited and noninhibited kinase groups was performed in Jalview (*68*) containing both AMPKα1 and AMPKα2, aligned using Tcoffee with default settings. Pairwise alignment scores in reference to AMPKα1 were calculated (*68*) with respective kinases in each group (table S2). FASTA alignment files were inputted into ConSurf web server (*40*, *41*), and the template Protein Data Bank (PDB): 7MYJ (chain A) was selected, with sequence reported in AMPKα2 numbering. Running parameters included phylogenetic tree construction using Neighbor Joining with ML distance, conservation score calculation using the Bayesian method and with amino acid substitution model chosen by best fit. For the BAY-3827–inhibited kinase group, the average pairwise distance was 1.32, whereas for the noninhibited group, it was 1.82. The ConSurf color scale (9-conserved; 1-variable) was assigned on the basis of calculated conservation scores and computed confidence intervals. When confidence interval scores >3 or the MSA number <6, this was flagged as insufficient data with an asterisk (*) to signify a low confidence score. The difference in conservation between kinase groups was calculated, omitting low confidence scores and focusing on conservation score changes at 3 or above (table S2).

### HEK293 cell culture and RSK signaling experiments

HEK293 cells (Invitrogen, R75007) were seeded at a density of 500,000 cells per well in six-well cell culture plates (Corning Incorporated Costar #3516) in Dulbecco’s modified Eagle medium (DMEM) high-glucose + GlutaMAX Supplement, pyruvate (Thermo Fisher Scientific, catalog no. 31966-021) containing penicillin G (100 U/ml), streptomycin (100 μg/ml), and 10% (v/v) FBS. Once attached, the next day, the media were changed to serum-free and the cells were starved for 16 hours followed by compound treatment with BAY-3827, BI-D1870 (MedChemExpress, #HY-10510) ± 10 μM MK-2206 dihydrochloride (MedChemExpress, #HY-10358) with 30 min incubation at 37°C followed by coincubation with 0.1 PMA μM (MedChemExpress, #HY-18739) or vehicle for additional 30 min with final assay DMSO concentration of 0.13%. All cell lines used here were tested monthly for mycoplasma and tested negative.

### Western blotting

Following compound treatment, the cells were washed with room temperature PBS and lysed on ice with the addition of lysis buffer [50 mM tris-HCl (pH 7.5), 150 mM NaCl, 1 mM EDTA, 1 mM EGTA, 0.27 M sucrose, 1% w/v Triton X-100, 20 mM glycerol-2 phosphate disodium, 50 mM NaF, and 5 mM Na_4_P_2_O_7_.10 H_2_O] containing proteases and phosphatase inhibitors [0.5 mM phenylmethylsulfonyl fluoride (PMSF), 1 mM benzamidine HCl, leupeptin (1 μg/ml), pepstatin A (1 μg/ml), 1 μM microcystin-LR, 1 mM Na_3_VO_4_, and 1 mM DTT]. Lysates were centrifuged at 10,000*g*, 4°C for 10 min, and protein concentration of the supernatant was determined with the Pierce Bradford Protein Assay Kit (Thermo Fisher Scientific, #23200) and read on the Hidex Plate Reader (software version 0.5.64.0). Samples were prepared in Laemmli sample buffer (tris 200 mM, 8% SDS, EDTA 2 mM, and 40% glycerol) supplemented with 10% DTT and boiled for 5 min at 100°C. Ten micrograms of protein per sample was run on a Criterion tris-glycine polyacrylamide gel (Bio-Rad, #5678095) and transferred to a nitrocellulose membrane. For cell signaling experiments in HEK293 cells, the samples were run on a Criterion TGX stain-free precast gel (Bio-Rad, catalog no. 5678085) with transfer to a polyvinylidene difluoride membrane performed in Bio-Rad Criterion blotter (#1704071) using tris-glycine transfer buffer (Bio-Rad, #1610734) following the manufacturer’s instructions. The membranes were probed at 4°C overnight in a 4% BSA TBS-T solution containing 0.02% sodium azide with the following antibodies where appropriate: Phospho-ACC (Ser^79^) (1:1000, Cell Signaling Technology, #3661S), ACC (C83B10) (1:1000, Cell Signaling Technology, #3676S), phospho-Raptor (Ser^792^) (1:1000, Cell Signaling Technology, #2083S), Raptor (24C12) (1:1000, Cell Signaling Technology, #2280S), GSK3αβ (1:2000, Sigma-Aldrich, #04-903), phospho-GSK3αβ (Ser^21/9^) (D17D2) (1:1000, Cell Signaling Technology, #8566S), ERK1/2 (137F5) (1:1000, Cell Signaling Technology, #4695), phospho-ERK1/2 (Thr^202^/Tyr^204^) (D13.14.4E) (1:1000, Cell Signaling Technology, #4370), phospho-AMPKα (Thr^172^) (40H9) (1:1000, Cell Signaling Technology, #2535S), AMPKα (1:1000, Cell Signaling Technology, #2532S), anti–glyceraldehyde-3-phosphate dehydrogenase antibody (1:5000, Cell Signaling Technology, #G8795), and vinculin (1:1000, Cell Signaling Technology, #13901). Membranes were scanned using the Odyssey Fc LiCor system (exposure time, 30 s) with densitometry analysis performed using LiCor Image Studio Lite normalizing to control loading protein. Figures were made using GraphPad Prism v10.

For adipocyte experiments, cell lysates (25 μg protein) were heated at 95°C for 3 min in LDS sample buffer, subjected to polyacrylamide gel electrophoresis on precast Novex gradient gels (Thermo Fisher Scientific) and electrotransferred to nitrocellulose membranes. Membranes were blocked for 30 min in 50 mM tris-HCl (pH 7.6), 137 mM NaCl, and 0.1% (w/v) Tween 20 (TBS-T) containing 10% (w/v) skimmed milk and then probed with the indicated antibodies in TBS-T containing 5% (w/v) BSA for 16 hours at 4°C. The following antibodies were used: anti-AMPK (#2603, 1:1000), anti–AMPK-pT172 (#2535, 1:1000), anti-Raptor (#2280, 1:1000), anti–Raptor-pS792 (#2083, 1:1000), anti-ACC1 (#3662, 1:1000), anti–ACC1-pS79 (#3661, 1:1000), anti-PKB/Akt (#9272, 1:1000), anti–PKB/Akt-pT308 (#9275, 1:1000), and anti-GSK3α/β-pS21/9 (#9331, 1:1000), which were all purchased from Cell Signaling Technology (Danvers, USA). Anti-AS160 (#07-741, 1:1000) was from Merck MilliporeSigma, anti–AS160-pT649 (#44-1071G, 1:1000) from Thermo Fisher Scientific, and anti-HSP90 from BD Biosciences (San Jose, CA, United States). Detection of primary antibodies was performed using horseradish peroxidase–conjugated secondary antibodies, and SuperSignal West Pico or Femto Chemiluminescent Substrates (Thermo Fisher Scientific). Luminescence signals were visualized in a ChemiDoc XRS+ (Bio-Rad) and quantified by densitometry using the software Image LabTM 5.1 (Bio-Rad). Signals were normalized to the loading control HSP90.

### HTRF assays

Human osteosarcoma U2OS WT cells were obtained from John Rouse (MRC Protein Phosphorylation and Ubiquitylation Unit, University of Dundee) and were cultured in DMEM high glucose + GlutaMAX Supplement, pyruvate (Thermo Fisher Scientific, catalog no. 31966-021) containing penicillin G (100 U/ml), streptomycin (100 μg/ml), and 10% (v/v) FBS seeding with 50,000 cells per well in a 50-μl volume in culture 96-well cell culture plates (Corning Incorporated costar, #3599). The next day, the media were changed to 50 μl of serum-free media, and 50 μl of serum-free media containing BAY-3827 or BAY-974 was dispensed and left to incubate at 37°C for 30 min. The media were aspirated and replaced with 50 μl of serum-free media containing either MK-8722 (10 μM) or vehicle and incubated at 37°C for 1 hour, with a final DMSO assay concentration of 1.03%. HTRF assays using either phospho-Ser^79^-ACC1 kit (Revvity, #64ACCPEG) or phospho-Thr^172^-AMPK kit (Revvity, #64AMPKPEG) were performed in triplicate in HTRF 96-well low volume white plates (Revvity, #66PL96001) following manufacturer’s instructions. Sealed plates incubated overnight in darkness conditions were scanned at room temperature in Multimode Plate Reader EnVision (PerkinElmer) machine following HTRF setup recommendations for EnVision (Revvity), equipped with a top mirror LANCE/DELFIA single mirror (#412), a UV2 (TRF) 320 excitation filter (#111), emission filter APC 665 (#205), and second emission filter Europium 615 (#203), with a 2000-μs cycle and 60-μs delay, with the number of flashes set to 100 as well as for the 2nd detector. Results were viewed in EnVision Manager software (version 1.13.3009.1401) where 665/615 ratios were calculated, and data were normalized to control condition as %. All assay ratios between control lysate signal/nonspecific signal were greater than 2 as expected of a successful assay following the manufacturer’s instructions. Data were visualized in GraphPad Prism v10.

### Primary hepatocyte RNA isolation, sequencing, and bioinformatics analysis

Primary hepatocytes were pretreated for 15 min with BAY-3827 5 μM at 37°C in MEM-199 supplemented with penicillin G (100 U/ml), streptomycin (100 mg/ml), and 100 nM dexamethasone followed by addition of MK-8722 10 μM treatment. Six hours posttreatment, the cells were washed twice with room-temperature PBS. RNA was isolated using QIAwave RNAeasy kit (catalog no. 74104) with on-column deoxyribonuclease (DNAse) treatment using Qiagen RNase-free DNase set (catalog no. 79256) following the manufacturer’s protocol. Messenger RNA-seq was performed by the Single-Cell Omics platform at the Novo Nordisk Foundation Center for Basic Metabolic Research. Libraries were prepared using the Universal Plus mRNA-seq protocol (Tecan) as recommended by the manufacturer. Libraries were quantified with NuQuant using the CLARIOstar Plate Reader (BMG Labtech), quality checked using a TapeStation instrument (Agilent Technologies), and subjected to 52–base pair paired-end sequencing on a NovaSeq 6000 (Illumina). The sequencing analysis pipeline nf-core (*69*) was used, with alignment and quantification selection of STAR and Salmon, and mouse GRCm38 set as the reference genome. Unique molecular identifier (UMI)-based deduplication was performed using UMI tools, and sorting and index alignments were completed with SAMtools as part of the pipeline. Differential expression analysis was carried out using the R package edgeR (*70*) and a batch-effect removal was performed in limma (*71*) to account for the variability induced by two mice (291 + 295) from which cells with the same treatments had been pooled to obtain the required cell confluence. Venn diagrams were produced using the R package ggvenn (*72*). Volcano plots were produced using the R package ggplot2, and significant up-regulated or down-regulated genes were described as having an FC ≥1.3 and FDR < 0.05. Heatmaps of log_2_ counts per million normalized counts were produced using R package pheatmap (*73*). GO enrichment analysis was performed using the R package clusterProfiler version 4.6.2 (*74*) setting FDR < 0.05 with reference organism specified as mouse, pAdjustMethod was set to BH, and ontology category set to biological process, with a qvalueCutoff of 0.05. Visualization analyses were conducted in RStudio version 4.2.0 (*75*).

### Plasmid preparation for AMPK construction

The coding sequence for the human AMPK-α2 KD (α2KD; residues 6 to 280) and its phosphomimetic mutant T172D-α2KD were custom synthesized (GENEWIZ, Azenta Life Sciences) with codon-optimized for *Escherichia coli* expression and cloned into the pET-14b vector with an N-terminal cleavable 6 × His-AviTag. For expression of full-length AMPKα2β2γ1, α2β2γ2, and α2β2γ3 complexes, polycistronic constructs encoding the three subunits were assembled into the pET-30b vector with a TEV-cleavable 6 × His–glutathione *S*-transferase (GST) tag on the γ subunit. Constructs were transformed into *E. coli* BL21(DE3)-RIL cells (Agilent) and cultured in LB medium at 37°C to an optical density at 600 nm of 0.7. Protein expression was induced with 0.2 μM isopropyl-β-d-thiogalactopyranoside, followed by overnight incubation at 18°C with shaking at 200 rpm. The cells were harvested by centrifugation, resuspended in lysis buffer [25 mM tris-HCl (pH 8.0), 500 mM NaCl, 10% (v/v) glycerol, 20 mM imidazole, 2 mM TCEP, 5 mM MgCl_2_, 0.2 mM PMSF, 1 mM benzamidine, and lysozyme (0.3 mg/ml)], and lysed by sonication (10 min total, 20 s on/40 s off) on ice. Lysates were clarified by centrifugation at 30,000*g* for 30 min at 4°C and filtered through a 0.45-μm syringe filter before loading onto the His-Trap column.

### Purification of AMPKα2 and T172D-α2 KDs

Clarified lysates were loaded onto a 5-ml HisTrap HP column (Cytiva) preequilibrated with lysis buffer. The column was washed with 10 column volumes (CV) of wash buffer containing tris 25 mM, NaCl 500 mM, glycerol 10%, imidazole 20 mM, TCEP 2 mM, MgCl_2_ 5 mM (pH 8.0). Proteins were eluted with a linear imidazole gradient (20 to 300 mM) more than 35 CV using an ÄKTA system. For crystallization, α2KD pooled fractions were incubated with TEV protease at a 1:50 molar ratio and dialyzed overnight at 4°C in buffer containing 25 mM tris-HCl (pH 8.0), 200 mM NaCl, 10% glycerol, 2 mM β-mercaptoethanol, and 5 mM MgCl_2_. This step of dialysis and TEV-cleavage was skipped for protein preparation that was used for Dianthus assays. Eluted fractions were pooled and further purified by size-exclusion chromatography on a Superdex S-75 16/600 column (Cytiva) equilibrated in 25 mM tris-HCl (pH 8.0), 200 mM NaCl, 10% glycerol, and 1 mM TCEP. The pure fractions of the proteins were concentrated to 5 mg/ml (α2KD) and 2 mg/ml (T172D-α2KD), and flash frozen for storage for further use.

### Purification of full-length AMPK complexes

Clarified lysates containing AMPK complexes were incubated with 5 ml of Ni–nitrilotriacetic acid (NTA) agarose resin (Qiagen) for 2 hours at 4°C. The resin was washed ≥six times with wash buffer and eluted with 300 mM imidazole-containing buffer. Eluted proteins were incubated with 5 ml of GST Sepharose (Cytiva), washed, and eluted in buffer containing 20 mM reduced glutathione.

Final purification was performed by gel filtration chromatography using a Superdex-S200 16/600 column (Cytiva) equilibrated in 25 mM Hepes (pH 7.5), 200 mM NaCl, 5% glycerol, and 1 mM TCEP. Peak fractions were concentrated using 30-kDa MWCO centrifugal concentrators (Millipore) with final concentration obtained at <1 mg/ml and flash-frozen in liquid nitrogen for storage at −80°C.

### Crystallization and structure determination

The crystallization screening was set up with various commercially available crystal screens with a protein concentration of 5 mg/ml in a sitting-drop vapor diffusion setup. Four hundred–nanoliter drops were setup with 1:1 ratio of protein to reservoir solution in a 96-well two-drop MRC plates. For α2KD–BAY-3827 cocrystallization, the protein at 0.5 mg/ml was mixed with the BAY-3827 compound at a final concentration of 0.1 mM, which was then concentrated to 5 mg/ml. The α2KD–BAY-3827 cocrystals were grown in a condition containing 0.1 M ammonium sulphate, 0.3 M sodium formate, 0.1 M sodium cacodylate (pH 6.5), 3% (w/v) γ-PGA, 3% (w/v) polyethylene glycol, molecular weight 20,000. The crystals were harvested and flash frozen in liquid nitrogen. Diffraction data were collected at 100 K at I24 beamline, Diamond light source. The data were processed by autoPROC (*76*) in space group P21212. The structure of α2KD-BAY-3827 was solved by molecular replacement using the program PHASER (*77*) with a search model 2H6D (Apo-α2KD structure). The structure was built in coot (*78*) and further refined in phenix-refine (*79*). The figures were generated in PyMOL (*80*).

### Dianthus binding assays

The binding affinity of BAY-3827 to α2KD, AMPK (T172D)-α2KD, or AMPK complexes α2β2γ1 and α2β2γ3 was measured by Dianthus (Nano Temper Technologies). BAY-3827 was diluted from a 100 mM stock in DMSO to 0.5 mM working concentration in the assay buffer (PBS with 0.005% Tween-20) with a final DMSO concentration of 2.5%, which was kept constant throughout the concentration gradient of the compound. For additional experiments, assay buffer containing Hepes 25 mM, NaCl 250 mM, and 0.005% Tween-20 was used to optimize the assay, enhancing signal-to-noise ratios. His-tagged α2KD, T172D-α2KD, or His-tagged AMPK-α2β2γ1 or α2β2γ3 complexes were labeled with His-tag labeling dye (His-Tag Labeling Kit RED-tris-NTA 2nd Generation). The ratio of fluorescence intensities (670/650 nm) over BAY-3827 concentration gradient ranging from 250 μM down to 0.015 μM. The concentration gradient was prepared by 1:1 serial dilution over 16 steps, with a setup of two to three technical replicates for each concentration. The binding experiments were repeated three times, and the mean *K*_d_ values of the inhibitor are reported in the appropriate figure alongside ± SEM (micromolar) displaying a representative graph. A concentration of 50 nM of the corresponding protein target was used in each performed assay. The ratio of fluorescence intensities was measured over BAY-3827 concentration gradient in GraphPad Prism using nonlinear regression (one site-total binding). Data quality was assessed by monitoring XYZ scans for each well to detect air bubbles or aggregation where present following the manufacturer’s instructions.

### Statistical analyses

Data points are plotted as means ± SEM. Data were inputted, and all statistical tests were performed using GraphPad Prism, version 10.1.0. One- or two-way analysis of variance (ANOVA) statistical tests were conducted using Tukey’s correction for multiple comparisons; specific test will be stated in the figure legend. Significance was accepted as *P* < 0.05. *N* number and statistical details for each experiment are reported in each figure legend. For lipogenesis generated data, a one-way Welch and Brown-Forsythe ANOVA test was conducted to assess statistical significance, which does not assume that all the groups were sampled from populations with equal variances (skew in data), with Sunnett T3 multiple comparisons correction. To estimate IC_50_ values, nonlinear regression fitting was conducted using the model (Inhibitor) versus response—variable slope in GraphPad Prism (version 10.1.0) and estimating values reported in nanomolar. To estimate binding affinities (*K*_d_) from performed spectral shift binding assays, data were fitted using a one-site total model [Y = *B*_max_*X/(K*_d_ *+ X) + NS*X + Background] where *X* is the ligand concentration, *B*_max_ represents the maximum binding, and NS is the slope of the nonlinear regression of *Y*/*X*. Values are reported in means ± SEM in micromolar.

## Supplementary Materials

### This PDF file includes:

Figs. S1 to S5 Tables S1 to S3 References
