# Supplementary material for: Mechanism and cellular actions of the potent AMPK inhibitor BAY-3827
Source: Sci Adv. 2025 Aug 22;11(34):eadx2434. doi: 10.1126/sciadv.adx2434 (PMC12372887; doi:10.1126/sciadv.adx2434)
Supplement: Supplementary file 1 — Figs. S1 to S5 Tables S1 to S3 References [file sciadv.adx2434_sm.pdf]

Science Advances
AAAS

Supplementary Materials for

Mechanism and cellular actions of the potent AMPK inhibitor BAY-3827

Conchita Fraguas Bringas *et al.*

Corresponding author: Kei Sakamoto, kei.sakamoto@sund.ku.dk; Elton Zeqiraj, e.zeqiraj@leeds.ac.uk

Sci. Adv. **11**, eadx2434 (2025)
DOI: 10.1126/sciadv.adx2434

This PDF file includes:

Figs. S1 to S5
Tables S1 to S3
References

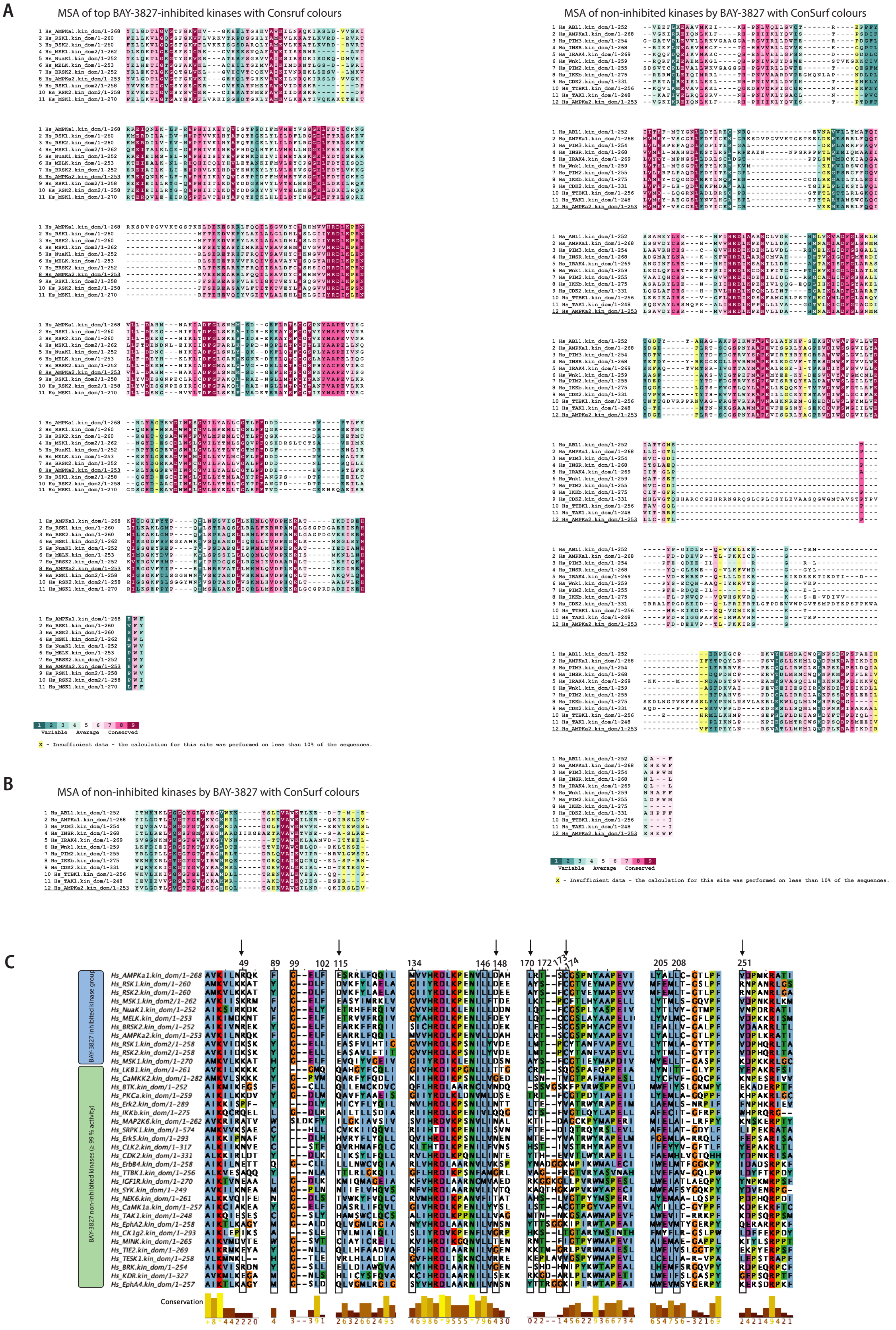
A
MSA of top BAY-3827-inhibited kinases with Consruf colours
MSA of non-inhibited kinases by BAY-3827 with ConSurf colours
Variable
Average
Conserved
X - Insufficient data - the calculation for this site was performed on less than 10% of the sequences.
B
MSA of non-inhibited kinases by BAY-3827 with ConSurf colours
C
BAY-3827 inhibited kinase group
BAY-3827 non-inhibited kinases (≥ 99% activity)
Conservation

Fig. S1. Sequence conservation of BAY-3827-inhibited and non-inhibited human kinases. (**A**) Multiple sequence alignment (MSA) of the kinase domains of BAY-3827-inhibited kinases (≤50% remaining activity) showing computed ConSurf *(40,41)* residue conservation (1-9) reported in AMPKα2 numbering and (**B**) MSA of kinase domains in the non-inhibited kinase group by BAY-3827 (≥99% remaining activity) with yellow colors representing insufficient data to calculate a conservation score. (**C**) MSA of the kinase domains of top inhibited kinases by BAY-3827 and non-inhibited kinases was conducted in Jalview *(68)*, with highlighted residues shown in AMPKα2 numbering with more than a 3-point conservation scale difference between kinase groups (Table S2). Arrows highlight residues with the highest difference in conservation across groups (≥5-point difference).

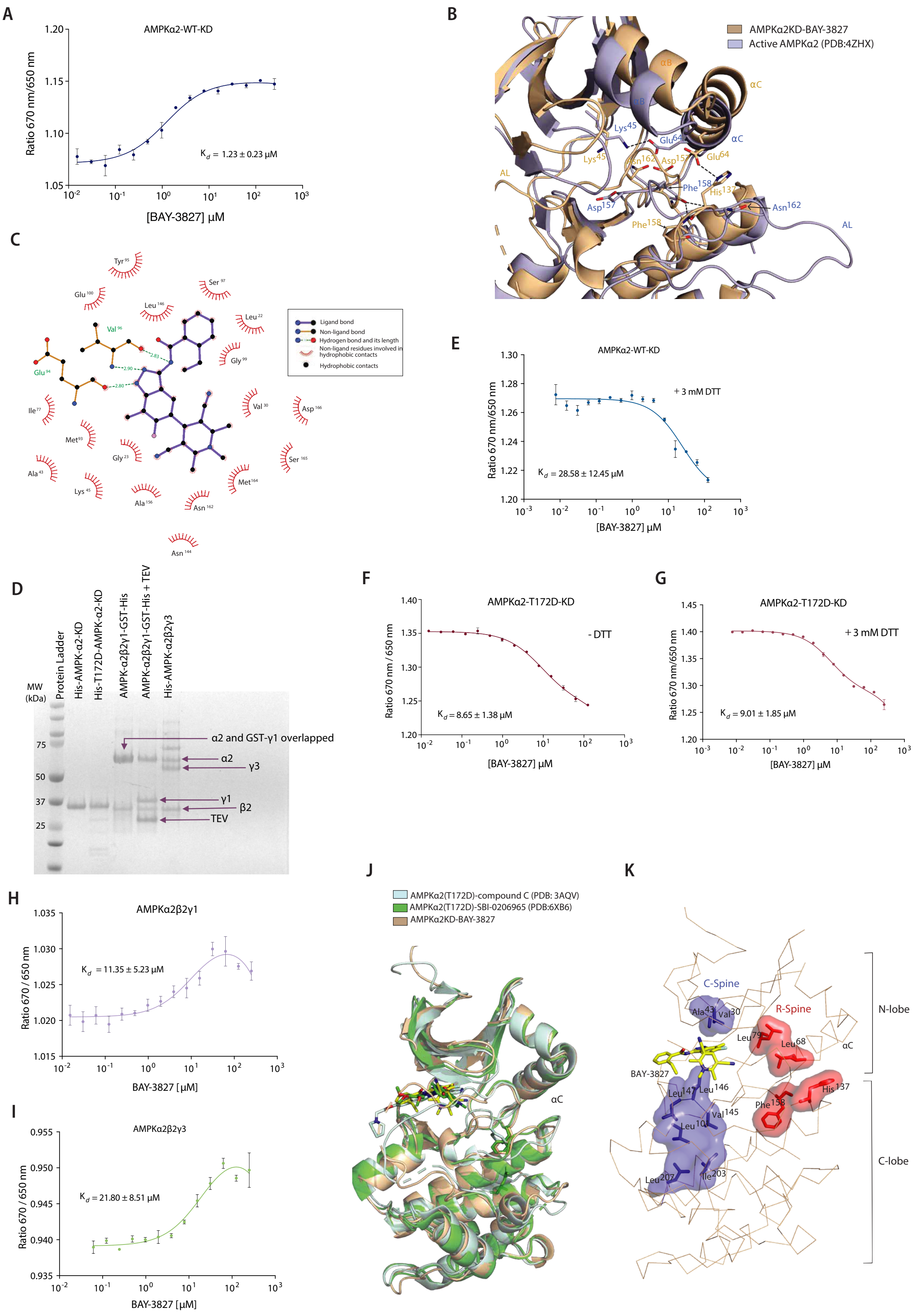

A
AMPKα2-WT-KD
Ratio 670 nm/650 nm
K_d = 1.23 ± 0.23 μM
[BAY-3827] μM
B
AMPKα2KD-BAY-3827
Active AMPKα2 (PDB:4ZHX)
αB
αC
Lys45
Glu64
Asp157
Asn162
Phe158
His137
AL
C
Tyr 95
Ser 97
Glu 100
Leu 146
Leu 22
Val 96
Glu 94
Gly 99
Ile 77
Val 30
Asp 166
Met 93
Gly 23
Ser 165
Ala 43
Met 164
Lys 45
Ala 156
Asn 162
Asn 144
2.83
2.90
2.80
Ligand bond
Non-ligand bond
Hydrogen bond and its length
Non-ligand residues involved in hydrophobic contacts
Hydrophobic contacts
D
MW (kDa)
Protein Ladder
His-AMPK-α2-KD
His-T172D-AMPK-α2-KD
AMPK-α2β2γ1-GST-His
AMPK-α2β2γ1-GST-His + TEV
His-AMPK-α2β2γ3
75
50
37
25
α2 and GST-γ1 overlapped
α2
γ3
γ1
β2
TEV
E
AMPKα2-WT-KD
+ 3 mM DTT
Ratio 670 nm/650 nm
K_d = 28.58 ± 12.45 μM
[BAY-3827] μM
F
AMPKα2-T172D-KD
- DTT
Ratio 670 nm / 650 nm
K_d = 8.65 ± 1.38 μM
[BAY-3827] μM
G
AMPKα2-T172D-KD
+ 3 mM DTT
Ratio 670 nm/650 nm
K_d = 9.01 ± 1.85 μM
[BAY-3827] μM
H
AMPKα2β2γ1
Ratio 670 / 650 nm
K_d = 11.35 ± 5.23 μM
BAY-3827 [μM]
I
AMPKα2β2γ3
Ratio 670 / 650 nm
K_d = 21.80 ± 8.51 μM
BAY-3827 [μM]
J
AMPKα2(T172D)-compound C (PDB: 3AQV)
AMPKα2(T172D)-SBI-0206965 (PDB:6XB6)
AMPKα2KD-BAY-3827
αC
K
C-Spine
R-Spine
Ala43
Val30
Leu79
Leu68
αC
BAY-3827
Leu147
Leu146
Val145
Leu10
Phe158
His137
Leu207
Ile203
N-lobe
C-lobe

Fig. S2. Structural features of AMPKα2KD bound to BAY-3827. (**A**) Spectral shift binding assay of BAY-3827 to AMPKα2 kinase domain (α2KD). Data is from three separate experiments with n=3 technical replicates shown as mean ± SEM showing a representative graph with K_d reported in µM. (**B**) The AMPKα2 kinase domain (α2KD) BAY-3827-bound structure (tan) was superimposed with active AMPKα2KD (purple, PDB: 4ZHX) with labelled displayed residues and features. (**C**) Ligand map of BAY-3827-interacting residues with AMPKα2KD showing hydrogen bonds and their lengths and ligand-protein hydrophobic contacts. (**D**) SDS-PAGE gel containing pure protein fractions of *E. coli*-purified inactive AMPK WT-α2KD (3µg), T172D-α2KD (3 µg) and AMPK complexes α2β2γ1 (both TEV protease cleaved and non-cleaved) and α2β2γ3 complex (6 µg). (**E**) Representative graphs of performed spectral shift binding assays of BAY-3827 binding to WT-α2KD in the presence of 3 mM DTT and (**F**)-(**G**) T172D-α2KD ± 3 mM DTT. Data are from three separate experiments with n=3 technical replicates reported as mean ± SEM (µM). (**H**) Spectral shift binding assay of BAY-3827 to inactive AMPKα2β2γ1 complex and (**I**) AMPKα2β2γ3 complex with data from four separate experiments with n=2-3 technical replicates. K_d values are reported as mean ± SEM (µM) and shown is a representative graph. (**J**) Superimposition of compound C (light cyan, PDB:3AQV) and SBI-0206965 (green, PDB:6BX6)-AMPKα2(T172D) kinase domain structures with AMPKα2KD bound to BAY-3827. (**K**) Catalytic (C) and regulatory (R) spines in AMPKα2KD-BAY-3827 structure shown in purple and red respectively, with the hydrophobic residues that compose them. Visualizations were conducted in PyMOL *(80)*.

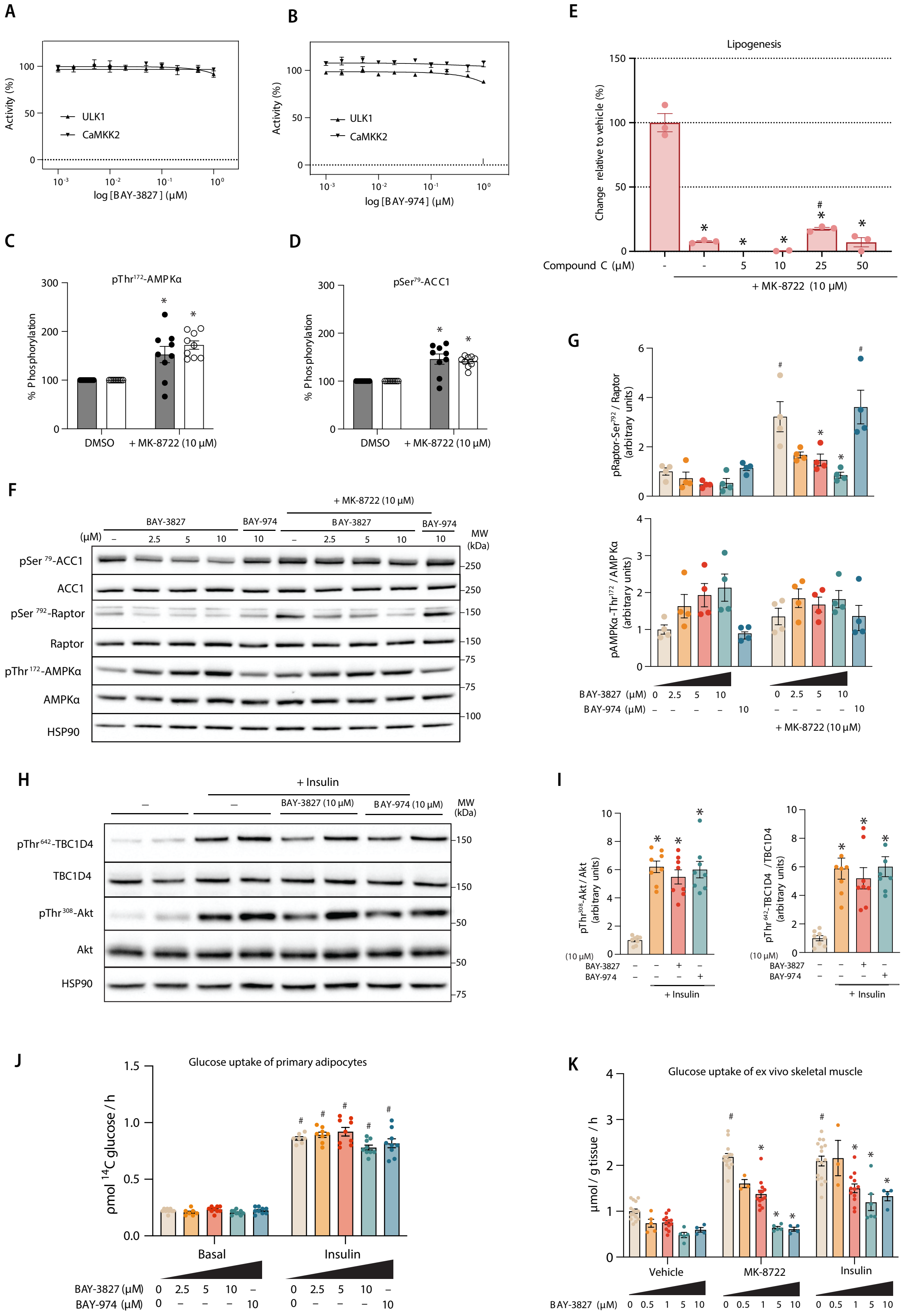
A
Activity (%)
ULK1
CaMKK2
log [BAY-3827] (µM)
B
Activity (%)
ULK1
CaMKK2
log [BAY-974] (µM)
C
pThr172-AMPKα
% Phosphorylation
DMSO
+ MK-8722 (10 µM)
D
pSer79-ACC1
% Phosphorylation
DMSO
+ MK-8722 (10 µM)
E
Lipogenesis
Change relative to vehicle (%)
Compound C (µM)
+ MK-8722 (10 µM)
F
+ MK-8722 (10 µM)
BAY-3827
BAY-974
(µM)
MW (kDa)
pSer79-ACC1
ACC1
pSer792-Raptor
Raptor
pThr172-AMPKα
AMPKα
HSP90
G
pRaptor-Ser792 / Raptor (arbitrary units)
pAMPKα-Thr172 / AMPKα (arbitrary units)
BAY-3827 (µM)
BAY-974 (µM)
+ MK-8722 (10 µM)
H
+ Insulin
BAY-3827 (10 µM)
BAY-974 (10 µM)
MW (kDa)
pThr642-TBC1D4
TBC1D4
pThr308-Akt
Akt
HSP90
I
pThr308-Akt / Akt (arbitrary units)
pThr642-TBC1D4 /TBC1D4 (arbitrary units)
(10 µM)
BAY-3827
BAY-974
+ Insulin
J
Glucose uptake of primary adipocytes
ρmol 14C glucose / h
Basal
Insulin
BAY-3827 (µM)
BAY-974 (µM)
K
Glucose uptake of ex vivo skeletal muscle
µmol / g tissue / h
Vehicle
MK-8722
Insulin
BAY-3827 (µM)

Fig. S3. BAY-3827 but not BAY-974 inhibit AMPK signaling in adipocytes and ex vivo skeletal muscle. (**A**) and (**B**) ULK1 and CaMKK2 kinase activity (%) assays in response to BAY-3827 or BAY-974 treatment from n=3. HTRF assay controls; vehicle (DMSO) and MK-8722 10 μM in (**C**) pThr^{172}-AMPK and (**D**) pSer^{79}-ACC kits. Data are n=3 from three independent experiments subjected to a two-way ANOVA test with $^{*}p < 0.05$. (**E**) Lipogenesis assay from n=6 mice seeded in n=3 technical triplicate wells. A one-way Brown-Forsynthe and Welch one-way ANOVA test was conducted due to unequal standard deviations in the data. Multiple comparisons were corrected with Dunnett T3 test. $^{*}p < 0.05$ vehicle vs + MK-8722 conditions; $^{\#}p < 0.05$ vehicle + MK-8722 vs compound C + MK-8722 conditions. (**F**) Representative western blot of AMPK signaling in adipocytes treated with BAY-3827 or BAY-974 in basal and MK-8722 (10 μM) conditions and (**G**) phospho/total ratios of pSer^{792}-Raptor and pThr^{172}-AMPK $^{\#}p < 0.05$ (basal vs + MK-8722). **(H)** Representative western blot of insulin signaling in primary adipocytes treated with BAY-3827 or BAY-974 ± insulin and corresponding (**I**) pThr^{308}-Akt and pThr^{642}-TBC1D4 phosho/total ratios where $^{*}p < 0.05$ (vehicle vs treatments). (**J**) Glucose uptake in primary adipocytes treated with increasing BAY-3827 doses in the presence of MK-8722 or insulin. Data is n=8-9 from three separate experiments and $^{\#}p < 0.05$ (basal vs insulin). (**K**) Ex-vivo glucose uptake of EDL muscle treated with BAY-3827 ± MK-8722 in the presence or absence of insulin. Data is n=3-5 from four separate experiments. $^{*}p < 0.05$ (veh vs treatment) and $^{\#}p < 0.05$ (veh vs veh + MK-8722; veh vs veh +insulin). (**J**) and (**K**) were subjected to a two-way ANOVA statistical test. All data points are shown as mean ± SEM.

A

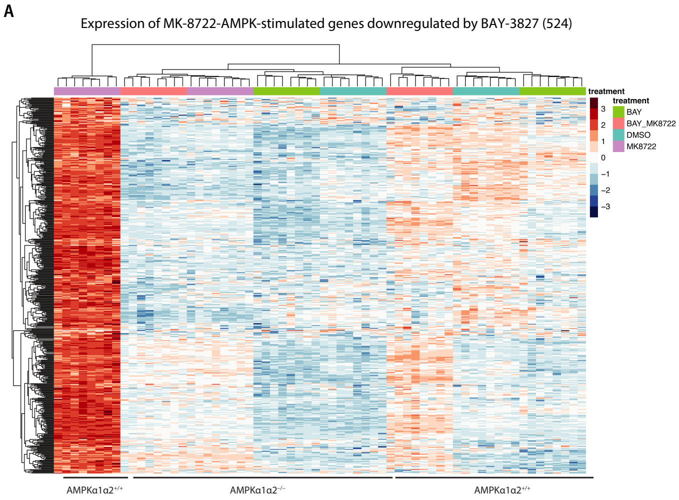

D

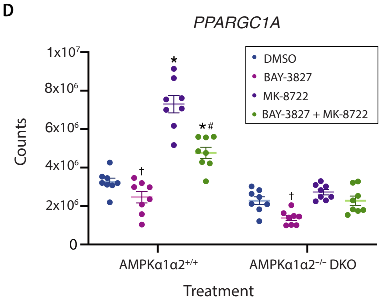

B

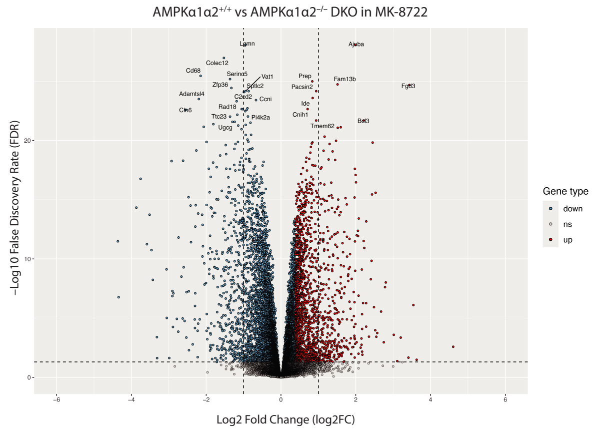

E

RSK1 genes

Gene	Name	logFC	FDR
FN1	fibronectin 1	-0.217763	0.0048283
MASTL	microtubule associated serine/threonine kinase - like	-0.8493677	2.2034E -06
SPC25	SPC25, NDC80 kinetochore complex component, homolog (S. cerevisiae)	-0.6826222	7.9197E -05
CENPW	centromere protein W	-0.473485	0.01371326
NEK2	NIMA (never in mitosis gene a) -related expressed kinase 2	-1.198511	3.7688E -08
CENPI	centromere protein I	-0.6534286	0.04589508
CENPE	centromere protein E	-1.2797475	0.00048978
DSN1	DSN1 homolog, MIS12 kinetochore complex component	-0.7890109	6.5304E -07

RSK2 genes

Gene	Name	logFC	FDR
STAT5B	signal transducer and activator of transcription 5B	-0.6006035	9.1572E -18
CALCOCO1	calcium binding and coiled coil domain 1	-0.7893176	1.932E -11

C

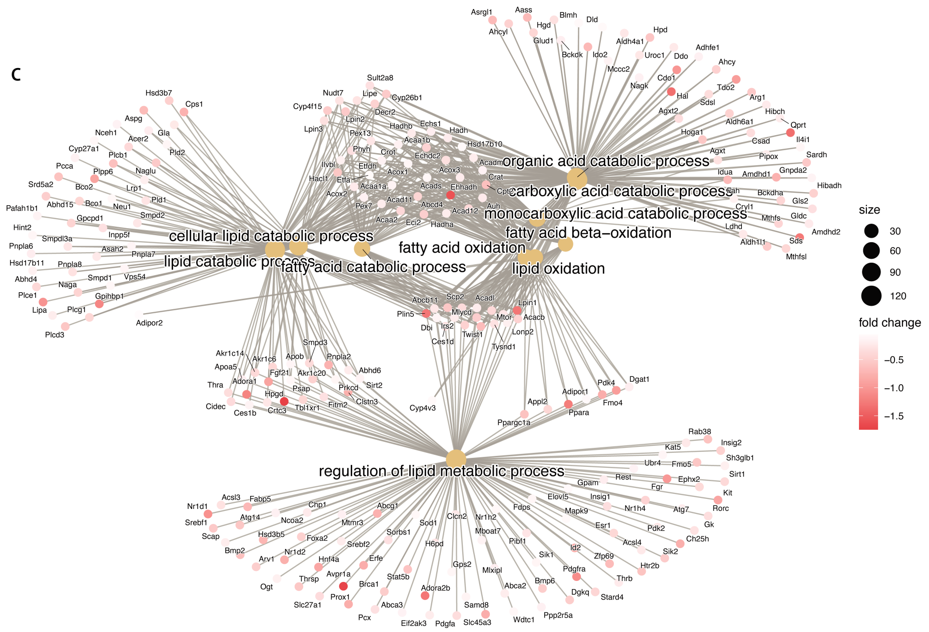

Fig. S4. Unbiased transcriptome sequencing of AMPKα1α2$^{-/-}$ double knockout (DKO) and AMPKα1α2$^{+/+}$ (control) primary hepatocytes treated with BAY-3827 ± MK-8722. (**A**) Heatmap representation of the gene expression of 524 significant MK-8722-stimulated genes downregulated by BAY-3827 with shown treatments. (**B**) Volcano plot showing top significant (abs (FC ≥ 1.3), FDR < 0.05) upregulated (red) and downregulated (blue) genes by loss of AMPKα1α2 vs control in MK-8722-treated cells. (**C**) Cnetplot of top gene ontology (GO) biological processes of downregulated genes by BAY-3827 + MK-8722 vs MK-8722 in control cells. (**D**) Plot of normalized counts of mitochondrial biogenesis gene *PPARGC1A* (ENSMUSG00000029167) in control and DKO cells across the depicted treatments. A two-way ANOVA test with Tukey's correction for multiple comparisons where *p < 0.05 (DMSO vs treatment); $^{\#}$p <0.05 (MK-8722 vs BAY-3827 + MK-8722) and †p <0.05 (MK-8722 vs BAY-3827). (**E**) Selected genes proposed as Ribosomal S6 kinase (RSK) 1 and 2 genes *(51)* downregulated by BAY-3827 in MK-8722-treated control hepatocytes.

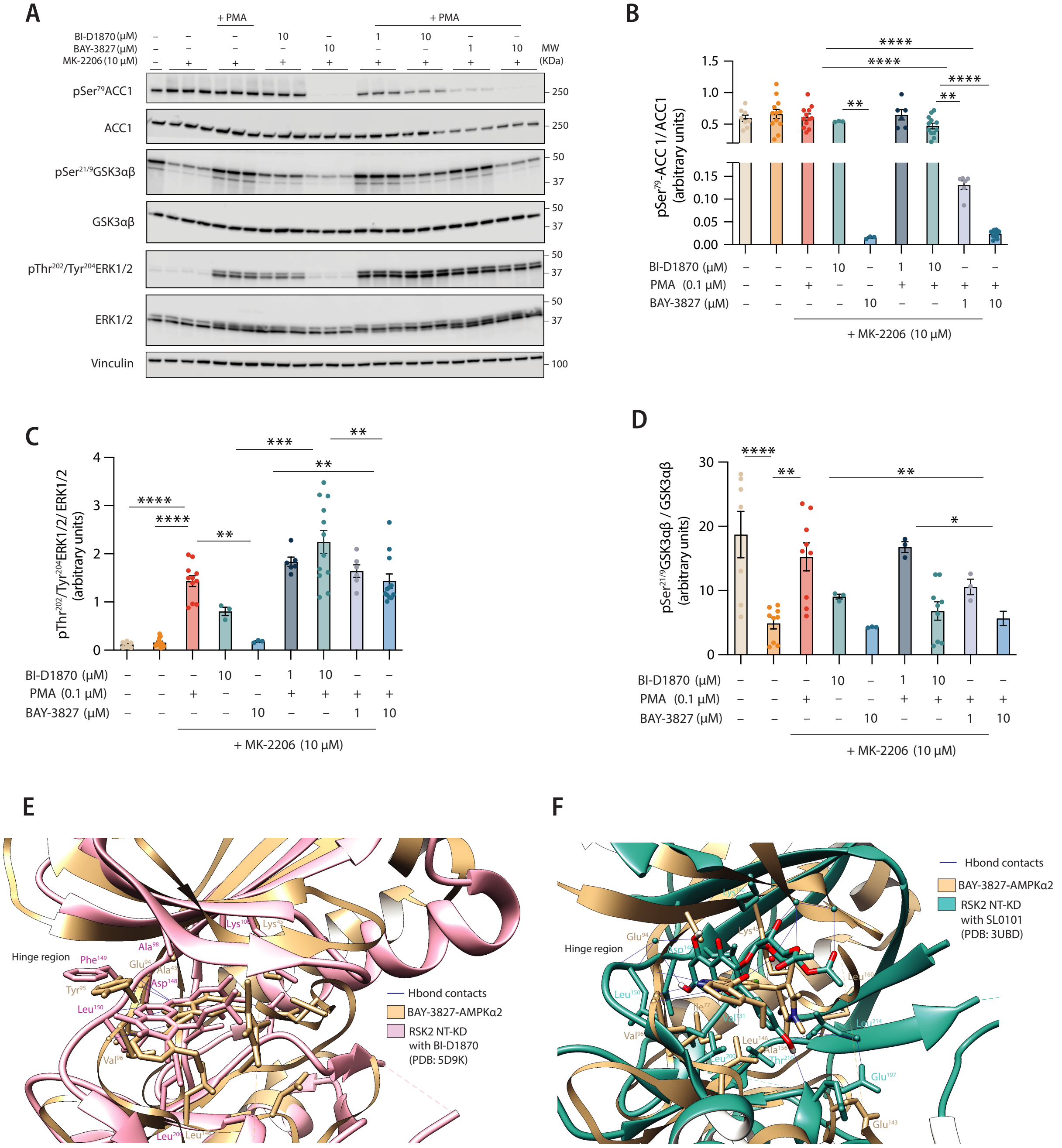
A
+ PMA
+ PMA
BI-D1870 (μM)
BAY-3827 (μM)
MK-2206 (10 μM)
MW (KDa)
pSer79ACC1
ACC1
pSer21/9GSK3αβ
GSK3αβ
pThr202/Tyr204ERK1/2
ERK1/2
Vinculin
250
250
50
37
50
37
50
37
50
37
100
B
pSer79-ACC 1/ACC1 (arbitrary units)
BI-D1870 (μM)
PMA (0.1 μM)
BAY-3827 (μM)
+ MK-2206 (10 μM)
C
pThr202/Tyr204ERK1/2/ ERK1/2 (arbitrary units)
BI-D1870 (μM)
PMA (0.1 μM)
BAY-3827 (μM)
+ MK-2206 (10 μM)
D
pSer21/9GSK3αβ / GSK3αβ (arbitrary units)
BI-D1870 (μM)
PMA (0.1 μM)
BAY-3827 (μM)
+ MK-2206 (10 μM)
E
Hinge region
Hbond contacts
BAY-3827-AMPKα2
RSK2 NT-KD with BI-D1870 (PDB: 5D9K)
F
Hinge region
Hbond contacts
BAY-3827-AMPKα2
RSK2 NT-KD with SL0101 (PDB: 3UBD)

Fig. S5. BAY-3827 and 90 kDa ribosomal S6 kinase (RSK) signaling. (**A**) Representative western blot of RSK signaling in HEK293 cells treated with BAY-3827 or RSK inhibitor BI-D1870 in the presence of Akt inhibitor MK-2206 in the presence or absence of phorbol 12-myristate 13-acetate (PMA) ERK1/2-RSK activator. (**B**) Calculated phospho/total ratios following (A) band quantification of pSer^{79}-ACC (**C**) pSer$^{21/9}$GSK3αβ and (**D**) pThr^{202}/Tyr^{204}ERK1/2. Data are n=3 from 3 independent experiments shown as mean ± SEM and subject to a one-way ANOVA analysis, where $*p < 0.05$, $**p < 0.002$ and $***p < 0.0002$ $****p < 0.0001$. (**E**) RSK2 NT-KD structure solved with BI-D1870 inhibitor **(**PDB: 5D9K) and (**F**) SL0101 inhibitor (PDB: 3UBD) *(53,54)* superimposed with BAY-3827-AMPKα2KD structure showing key interacting residues, with hydrogen bonds shown in blue. Visualizations were conducted in Chimera *(81)*.

	BAY-3827 0.1 µM	
**Kinases**	**Kinase activity remaining (%)**	**SD**
AMPK	7	0
PHK	13	3
RSK1	18	1
Aurora B	35	9
MINK1	40	2
TBK1	42	6
IRAK1	44	9
MST3	50	6
p38b MAPK	55	2
MSK1	56	3
MST4	64	3
ERK8	66	3
CHK1	66	3
IGF-1R	67	5
NUAK1	68	9
TIE2	69	9
TESK1	70	3
TTK	74	3
GSK3b	74	5
SmMLCK	76	5
MARK2	77	5
BRSK1	77	4
HIPK3	77	1
CSK	79	1
DDR2	80	8
PAK4	80	12
ERK5	80	10
CAMK1	80	4
MELK	81	2
BRSK2	82	5
MARK1	82	1
MAPKAP-K2	82	4
PDGFRA	82	10
MAP4K3	82	10
BRK	83	9
PDK1	83	5
ROCK 2	83	3
EPH-B3	84	2
IKKe	85	8

MLK3	85	1
BTK	85	9
MST2	85	8
CK2	85	3
PIM1	86	9
TGFBR1	86	10
CLK2	87	4
TrkA	87	9
S6K1	87	9
PRK2	88	1
PAK2	88	12
TSSK1	88	0
ULK1	88	3
JNK3	88	7
MLK1	89	1
IRR	89	3
FGF-R1	89	2
DYRK2	89	4
VEG-FR	89	10
Aurora A	90	6
p38g MAPK	90	15
RSK2	90	5
Src	90	11
JAK3	90	15
PINK	90	5
PKCa	90	9
EPH-B1	91	9
EF2K	91	1
SYK	91	10
CHK2	93	1
PKBb	93	5
TTBK2	93	14
Lck	93	2
TAO1	94	13
PKCγ	94	2
EPH-A4	94	8
PKA	94	1
JNK1	94	11
DAPK1	94	5
STK33	94	6
HIPK1	94	2

PKBa	95	5
CK1γ2	95	5
ASK1	95	7
ULK2	96	3
DYRK1A	96	4
ZAP70	96	6
CDK9-Cyclin T1	97	12
MAP4K5	97	5
DYRK3	97	3
CAMKKb	97	6
MEKK1	97	1
SGK1	97	10
LKB1	97	1
MKK2	97	12
p38a MAPK	97	4
PLK1	97	12
EPH-B2	98	4
YES1	98	8
MARK4	98	4
MARK3	98	3
EIF2AK3	98	12
MNK2	99	14
p38d MAPK	99	5
CDK2-Cyclin A	99	9
SIK3	99	1
PKD1	100	16
MKK1	100	10
PAK5	100	7
WNK1	101	7
TTBK1	101	7
MKK6	101	11
PIM3	101	11
PRAK	101	13
MAPKAP-K3	101	1
IR	102	1
RIPK2	102	4
NEK6	102	13
CK1δ	103	10
GCK	105	15
IKKb	105	10
ERK1	105	10

JNK2	105	9
PAK6	106	5
SIK2	107	11
ABL	107	12
EPH-B4	109	4
OSR1	111	7
IRAK4	111	6
SRPK1	111	6
PKCz	112	6
PIM2	112	12
EPH-A2	114	15
ERK2	116	6
TAK1	118	13
TLK1	118	12
HIPK2	121	2
NEK2a	122	3
MNK1	126	12
HER4	135	4
MPSK1	135	0
	**BAY-974 0.1 µM**	
**Kinases**	**Kinase activity remaining (%)**	**SD**
SYK	12	3
IRAK1	26	6
YES1	44	7
ULK1	45	7
TSSK1	55	10
Lck	58	3
ABL	59	1
NUAK1	60	1
ULK2	60	10
Aurora B	62	9
GCK	65	14
RSK1	66	2
BRK	70	1
ERK5	70	4
MAP4K3	73	9
CAMK1	78	3
CHK1	78	1
MINK1	79	0
DYRK2	79	7
Aurora A	79	5

TTK	79	4
CK2	79	1
MARK3	80	2
SmMLCK	80	7
MAPKAP-K3	81	9
EPH-A4	81	2
SIK3	81	10
p38b MAPK	81	1
IR	81	11
EPH-B2	82	4
PAK5	82	5
STK33	82	5
PIM1	82	8
ASK1	83	1
DYRK3	83	3
PRK2	83	7
MAPKAP-K2	84	3
PKD1	84	2
PDK1	85	2
NEK6	85	10
PDGFRA	86	1
CHK2	87	1
MARK2	87	4
SIK2	88	4
TAO1	88	11
TESK1	88	4
PAK2	88	4
IRR	88	2
IKKb	89	2
MAP4K5	89	6
MST3	89	1
DDR2	89	11
MELK	90	3
MLK3	90	15
MLK1	90	2
FGF-R1	91	9
HIPK1	91	1
TTBK2	91	13
TBK1	91	6
ERK8	91	6
Src	92	8

IRAK4	92	15
MARK1	93	14
HER4	93	17
CLK2	93	13
GSK3b	93	3
TAK1	93	9
BTK	94	2
MKK6	94	3
MST2	94	1
ZAP70	94	11
PINK	95	11
VEG-FR	95	4
PKBa	95	8
MKK2	95	1
p38g MAPK	95	0
PAK4	96	14
MARK4	96	0
BRSK1	96	15
CDK2-Cyclin A	96	6
S6K1	97	6
SRPK1	97	1
TrkA	97	2
MNK2	98	11
ROCK 2	98	16
AMPK (hum)	98	12
MST4	98	3
HIPK3	99	2
PKBb	99	6
MEKK1	99	8
TLK1	100	3
MNK1	100	5
MKK1	100	7
MSK1	100	7
DYRK1A	101	9
IKKe	101	3
PAK6	102	0
ERK1	103	3
PKA	103	5
PKCz	103	5
TIE2	103	5
LKB1	104	13

JNK2	104	7
JNK3	104	5
TTBK1	104	14
MPSK1	105	25
PLK1	105	3
JAK3	106	6
JNK1	106	3
EPH-B1	107	13
CAMKKb	107	2
PRAK	107	15
RIPK2	108	2
p38a MAPK	108	9
p38d MAPK	108	0
HIPK2	108	8
OSR1	109	7
DAPK1	109	5
EPH-A2	109	8
ERK2	110	0
PKCa	110	14
BRSK2	112	1
CK1γ2	112	7
TGFBR1	113	15
EIF2AK3	113	7
PIM2	113	8
CDK9-Cyclin T1	114	9
PKCγ	115	13
CK1δ	116	1
EPH-B4	116	10
WNK1	117	12
RSK2	117	2
EPH-B3	119	4
EF2K	119	1
PHK	120	5
NEK2a	121	7
CSK	123	0
PIM3	125	1
SGK1	127	3
IGF-1R	147	12

Table S1. BAY-3827 (A) and BAY-974 (B) kinase selectivity data at 0.1 µM across a panel of 140 human kinases. From duplicate conditions reported as mean kinase activity remaining (%) and standard deviation (SD) values.

**Percentage identity (PID) scores of kinase domains to AMPKα1 kinase domain (%)**	
**Non-inhibited kinases by BAY-3827 (≥99% activity)**	**BAY-3827-inhibited kinases (≤50% activity)**
ABL kinase domain: 24.63%	AMPKα2 kinase domain: 85.07%
PIM3 kinase domain: 29.71%	RSK1 kinase domain 1: 30.11%
INSR kinase domain: 25%	RSK1 kinase domain 2: 31.79%
IRAK kinase domain: 25.74%	MSK1 kinase domain 1: 28.32%
WNK1 kinase domain: 23.02%	MSK1 kinase domain 2: 33.1%
PIM2 kinase domain: 29.86 %	NUAK1 kinase domain: 45.52%
IKKβ kinase domain: 24.1%	RSK2 kinase domain 1: 30.94%
CDK2 kinase domain: 28.94%	RSK2 kinase domain 2: 32.86%
TTBK1 kinase domain: 23.47%	MELK kinase domain: 46.67%
TAK1 kinase domain: 28.21%	BRSK2 kinase domain: 47.76%

**ConSurf scores of non-inhibited kinases (A) and BAY-3827-inhibited kinases (B)**					
**AMPKα2 residue**	**Score (A)**	**Confidence interval**	**Score (B)**	**Confidence interval**	**Δ Change (B-A)**
**TYR:16:A**	4	-0.437, 0.877 6,3	7	-1.105, -0.141 8,5	3
VAL:17:A	4	-0.335, 1.157 6,3	3	0.219, 2.137 5,2	-1
**LEU:18:A**	3	-0.114, 1.520 5,2	6	-1.037, -0.141 8,5	3
GLY:19:A	2	0.461, 2.070 4,1	3	0.219, 2.137 5,2	1
ASP:20:A	4	-0.228, 1.157 6,3	5	-0.678, 0.530 7,4	1
THR:21:A	5	-0.711, 0.461 7,4	7	-1.105, -0.340 8,6	2
LEU:22:A	6	-1.110, -0.228 8,6	7	-1.172, -0.340 9,6	1
GLY:23:A	9	-2.015, -1.415 9,8	9	-1.610, -0.899 9,8	0
VAL:24:A	2	0.293, 2.070 4,1	3	0.089, 1.584 5,3	1
GLY:25:A	9	-2.015, -1.415 9,8	9	-1.610, -0.899 9,8	0
THR:26:A	4	-0.335, 1.157 6,3	5	-0.753, 0.219 7,5	1
PHE:27:A	8	-1.659, -1.034 9,7	5	-0.431, 0.718 6,4	-3
GLY:28:A	8	-1.753, -0.956 9,7	7	-1.105, -0.141 8,5	-1
LYS:29:A	4	-0.437, 0.877 6,3	5	-0.599, 0.718 7,4	1
VAL:30:A	9	-2.015, -1.574 9,9	9	-1.610, -1.037 9,8	0
LYS:31:A	6	-1.110, -0.114 8,5	7	-1.105, -0.141 8,5	1
ILE:32:A	7	-1.262, -0.437 8,6	4	-0.141, 1.584 5,3	-3
GLY:33:A	6	-1.034, 0.009 7,5	5	-0.431, 0.718 6,4	-1
GLU:34:A	1	0.652, 3.457 4,1	3	0.530, 2.137 4,2	2
HIS:35:A	3*	0.145, 2.070 5,1	6	-0.899, -0.031 8,5	
GLN:36:A	3	0.009, 1.520 5,2	2	0.942, 3.551 4,1	-1

LEU:37:A	2	0.461, 2.070 4,1	3	0.219, 2.137 5,2	1
THR:38:A	7	-1.493, -0.711 9,7	7	-0.968, -0.141 8,5	0
GLY:39:A	4	-0.335, 1.157 6,3	5	-0.753, 0.365 7,4	1
HIS:40:A	3*	0.009, 2.070 5,1	5	-0.431, 0.718 6,4	
LYS:41:A	3	0.009, 1.520 5,2	4	-0.141, 1.218 5,3	1
VAL:42:A	9	-2.015, -1.574 9,9	6	-1.037, -0.031 8,5	-3
ALA:43:A	9	-2.195, -1.753 9,9	9	-1.722, -1.307 9,9	0
VAL:44:A	6	-0.956, 0.009 7,5	7	-1.172, -0.431 9,6	1
LYS:45:A	9	-2.015, -1.415 9,8	9	-1.722, -1.240 9,9	0
ILE:46:A	4	-0.437, 0.877 6,3	6	-0.968, -0.031 8,5	2
LEU:47:A	4	-0.335, 0.877 6,3	6	-0.827, 0.219 8,5	2
ASN:48:A	3	0.009, 1.520 5,2	5	-0.516, 0.530 7,4	2
**ARG:49:A**	4	-0.437, 0.877 6,3	8	-1.448, -0.753 9,7	4
GLN:50:A	5	-0.531, 0.652 6,4	4	-0.031, 1.218 5,3	-1
LYS:51:A	6	-0.956, 0.293 7,4	6	-1.037, -0.141 8,5	0
ILE:52:A	4	-0.531, 0.877 6,3	4	-0.031, 1.584 5,3	0
ARG:53:A	4*	-0.335, 1.520 6,2	3	0.089, 2.137 5,2	
SER:54:A	4	-0.335, 1.157 6,3	3	-0.031, 1.584 5,3	-1
LEU:55:A	3*	0.009, 2.070 5,1	3	0.219, 2.137 5,2	
ASP:56:A	3	0.009, 1.520 5,2	3	-0.031, 1.584 5,3	0
VAL:57:A	3*	0.009, 2.070 5,1	4*	-0.431, 1.218 6,3	
VAL:58:A	4	-0.437, 0.877 6,3	4	-0.031, 1.584 5,3	0
GLY:59:A	4	-0.531, 0.877 6,3	3	0.219, 2.137 5,2	-1
LYS:60:A	5	-0.531, 0.652 6,4	4	-0.243, 0.942 6,4	-1
ILE:61:A	5	-0.711, 0.461 7,4	7	-1.105, -0.141 8,5	2
LYS:62:A	1	0.652, 3.457 4,1	3	0.219, 1.584 5,3	2
ARG:63:A	4	-0.228, 1.157 6,3	4	-0.340, 0.942 6,4	0
GLU:64:A	9	-2.195, -1.753 9,9	9	-1.722, -1.172 9,9	0
ILE:65:A	7	-1.187, -0.335 8,6	9	-1.525, -0.968 9,8	2
GLN:66:A	4	-0.437, 0.877 6,3	4	-0.340, 0.942 6,4	0
ASN:67:A	5	-0.876, 0.293 7,4	4	-0.340, 0.942 6,4	-1
LEU:68:A	8	-1.574, -0.876 9,7	8	-1.448, -0.753 9,7	0
LYS:69:A	7	-1.262, -0.531 8,6	4	-0.141, 1.218 5,3	-3

LEU:70:A	5	-0.531, 0.652 6,4	2	0.942, 3.551 4,1	-3
PHE:71:A	5	-0.622, 0.652 6,4	3	0.365, 2.137 4,2	-2
ARG:72:A	5	-0.622, 0.652 6,4	3	0.089, 1.584 5,3	-2
HIS:73:A	8	-1.574, -0.876 9,7	9	-1.525, -0.968 9,8	1
PRO:74:A	6	-1.034, 0.009 7,5	7	-1.240, -0.340 9,6	1
HIS:75:A	8	-1.659, -1.034 9,7	8	-1.240, -0.516 9,7	0
ILE:76:A	7	-1.262, -0.437 8,6	7	-1.172, -0.431 9,6	0
ILE:77:A	8	-1.753, -1.187 9,8	6	-0.827, 0.089 8,5	-2
LYS:78:A	6	-1.034, -0.114 7,5	5	-0.678, 0.530 7,4	-1
LEU:79:A	7	-1.415, -0.622 8,6	8	-1.448, -0.753 9,7	1
TYR:80:A	5	-0.622, 0.652 6,4	6	-0.968, -0.031 8,5	1
GLN:81:A	7	-1.574, -0.711 9,7	4	-0.141, 1.218 5,3	-3
**VAL:82:A**	6	-0.956, 0.145 7,5	9	-1.610, -1.037 9,8	3
**ILE:83:A**	2	0.293, 2.070 4,1	5	-0.678, 0.530 7,4	3
SER:84:A	4	-0.437, 0.877 6,3	5	-0.678, 0.365 7,4	1
THR:85:A	5	-0.795, 0.461 7,4	6	-0.968, -0.031 8,5	1
PRO:86:A	4*	-0.335, 1.520 6,2	3	-0.031, 1.584 5,3	
THR:87:A	3	-0.114, 1.520 5,2	2	0.942, 3.551 4,1	-1
ASP:88:A	2	0.461, 2.070 4,1	4	-0.340, 1.218 6,3	2
**PHE:89:A**	2	0.293, 2.070 4,1	5	-0.516, 0.718 7,4	3
PHE:90:A	2	0.293, 2.070 4,1	4	-0.340, 0.942 6,4	2
MET:91:A	7	-1.415, -0.622 8,6	5	-0.753, 0.219 7,5	-2
VAL:92:A	9	-1.861, -1.262 9,8	8	-1.377, -0.753 9,7	-1
MET:93:A	7	-1.338, -0.622 8,6	7	-0.968, -0.141 8,5	0
GLU:94:A	9	-1.861, -1.415 9,8	8	-1.448, -0.827 9,8	-1
TYR:95:A	5	-0.711, 0.461 7,4	5	-0.678, 0.530 7,4	0
VAL:96:A	6	-0.956, 0.009 7,5	4	-0.431, 0.942 6,4	-2
SER:97:A	4	-0.228, 1.157 6,3	5	-0.431, 0.718 6,4	1
GLY:98:A	5	-0.711, 0.461 7,4	7	-1.240, -0.340 9,6	2
**GLY:99:A**	6	-1.187, -0.228 8,6	9	-1.610, -0.899 9,8	3
GLU:100:A	7	-1.187, -0.335 8,6	8	-1.448, -0.753 9,7	1
LEU:101:A	9	-2.195, -1.574 9,9	9	-1.722, -1.105 9,8	0
**PHE:102:A**	2	0.293, 2.070 4,1	7	-1.240, -0.431 9,6	5

ASP:103:A	6	-0.956, 0.009 7,5	7	-1.172, -0.340 9,6	1
TYR:104:A	5	-0.795, 0.293 7,4	6	-0.827, 0.219 8,5	1
ILE:105:A	7	-1.493, -0.711 9,7	8	-1.377, -0.678 9,7	1
CYS:106:A	5	-0.711, 0.461 7,4	3	0.089, 1.584 5,3	-2
LYS:107:A	3	-0.114, 1.520 5,2	5	-0.678, 0.365 7,4	2
HIS:108:A	1	0.877, 3.457 3,1	3	0.089, 1.584 5,3	2
GLY:109:A	3	0.009, 1.520 5,2	3	0.089, 1.584 5,3	0
**ARG:110:A**	2	0.461, 2.070 4,1	5	-0.516, 0.718 7,4	3
**VAL:111:A**	3	0.009, 1.520 5,2	7	-1.172, -0.243 9,6	4
GLU:112:A	3*	0.009, 2.070 5,1	7	-1.037, -0.243 8,6	
GLU:113:A	4*	-0.228, 1.520 6,2	8	-1.448, -0.753 9,7	
MET:114:A	1	1.157, 3.457 3,1	3	0.365, 2.137 4,2	2
**GLU:115:A**	2	0.461, 3.457 4,1	8	-1.448, -0.753 9,7	6
**ALA:116:A**	2	0.293, 2.070 4,1	6	-0.827, 0.219 8,5	4
ARG:117:A	5	-0.711, 0.461 7,4	7	-1.037, -0.141 8,5	2
ARG:118:A	4	-0.437, 0.877 6,3	2	0.718, 3.551 4,1	-2
LEU:119:A	5	-0.531, 0.652 6,4	4	-0.243, 1.218 6,3	-1
PHE:120:A	6	-0.956, 0.145 7,5	6	-0.827, 0.219 8,5	0
GLN:121:A	2	0.293, 2.070 4,1	4	-0.243, 0.942 6,4	2
GLN:122:A	8	-1.753, -1.187 9,8	7	-1.105, -0.340 8,6	-1
ILE:123:A	7	-1.338, -0.531 8,6	8	-1.377, -0.678 9,7	1
LEU:124:A	4	-0.437, 1.157 6,3	4	-0.431, 0.942 6,4	0
**SER:125:A**	5	-0.622, 0.461 6,4	8	-1.307, -0.516 9,7	3
ALA:126:A	6	-0.876, 0.145 7,5	7	-1.105, -0.243 8,6	1
VAL:127:A	6	-0.956, 0.009 7,5	7	-1.307, -0.516 9,7	1
ASP:128:A	4	-0.228, 1.157 6,3	4	-0.141, 1.218 5,3	0
TYR:129:A	6	-1.034, -0.114 7,5	6	-0.968, -0.141 8,5	0
CYS:130:A	8	-1.659, -0.876 9,7	6	-0.899, 0.089 8,5	-2
HIS:131:A	8	-1.861, -1.262 9,8	9	-1.722, -1.307 9,9	1
ARG:132:A	6	-1.110, -0.114 8,5	4	-0.340, 1.584 6,3	-2
HIS:133:A	5	-0.622, 0.461 6,4	5	-0.516, 0.718 7,4	0
**MET:134:A**	3	0.009, 1.520 5,2	6	-0.968, 0.089 8,5	3
VAL:135:A	6	-1.187, -0.228 8,6	7	-1.105, -0.340 8,6	1

VAL:136:A	7	-1.493, -0.795 9,7	7	-1.172, -0.431 9,6	0
HIS:137:A	9	-2.195, -1.753 9,9	9	-1.525, -0.968 9,8	0
ARG:138:A	9	-2.195, -1.659 9,9	9	-1.722, -1.172 9,9	0
ASP:139:A	9	-2.195, -1.659 9,9	9	-1.722, -1.172 9,9	0
LEU:140:A	7	-1.493, -0.795 9,7	8	-1.448, -0.753 9,7	1
LYS:141:A	9	-2.015, -1.574 9,9	9	-1.722, -1.240 9,9	0
PRO:142:A	6	-1.110, -0.114 8,5	5*	-0.899, 0.365 8,4	
GLU:143:A	6	-1.034, -0.114 7,5	8	-1.448, -0.753 9,7	2
ASN:144:A	9	-2.195, -1.861 9,9	9	-1.826, -1.307 9,9	0
VAL:145:A	5	-0.795, 0.293 7,4	6	-0.899, 0.089 8,5	1
**LEU:146:A**	6	-1.187, -0.228 8,6	9	-1.722, -1.105 9,8	3
LEU:147:A	6	-0.876, 0.145 7,5	6	-0.827, 0.219 8,5	0
**ASP:148:A**	4	-0.437, 0.877 6,3	9	-1.722, -1.172 9,9	5
**ALA:149:A**	3	-0.114, 1.520 5,2	6	-0.899, 0.089 8,5	3
HIS:150:A	2	0.293, 2.070 4,1	4	-0.431, 0.942 6,4	2
MET:151:A	1	0.877, 3.457 3,1	3	0.089, 1.584 5,3	2
ASN:152:A	2*	0.145, 2.070 5,1	5	-0.599, 0.530 7,4	
ALA:153:A	4	-0.335, 1.157 6,3	5	-0.678, 0.530 7,4	1
LYS:154:A	9	-2.015, -1.415 9,8	6	-0.968, -0.031 8,5	-3
ILE:155:A	8	-1.493, -0.876 9,7	8	-1.377, -0.678 9,7	0
ALA:156:A	4	-0.335, 1.157 6,3	5	-0.516, 0.530 7,4	1
ASP:157:A	9	-2.195, -1.659 9,9	9	-1.722, -1.172 9,9	0
PHE:158:A	8	-1.659, -1.034 9,7	9	-1.722, -1.172 9,9	1
GLY:159:A	9	-2.015, -1.415 9,8	9	-1.610, -0.899 9,8	0
LEU:160:A	6	-1.110, -0.114 8,5	7	-1.172, -0.340 9,6	1
SER:161:A	8	-1.493, -0.876 9,7	7	-1.172, -0.431 9,6	-1
ASN:162:A	6	-1.110, -0.228 8,6	5	-0.678, 0.365 7,4	-1
MET:163:A	2*	0.145, 2.070 5,1	4	-0.340, 0.942 6,4	
MET:164:A	4	-0.335, 0.877 6,3	1	1.218, 3.551 3,1	-3
SER:165:A	2	0.461, 2.070 4,1	2	0.718, 2.137 4,2	0
ASP:166:A	4	-0.437, 0.877 6,3	3	0.530, 2.137 4,2	-1
GLY:167:A	3*	0.009, 2.070 5,1	5	-0.678, 0.530 7,4	
GLU:168:A	2	0.461, 3.457 4,1	2	0.530, 2.137 4,2	0

PHE:169:A	3*	0.145, 2.070 5,1	3	0.530, 2.137 4,2	
**LEU:170:A**	2	0.461, 2.070 4,1	8	-1.448, -0.678 9,7	6
ARG:171:A	7	-1.187, -0.335 8,6	5	-0.431, 0.718 6,4	-2
**TPO:172:A**	5	-0.795, 0.293 7,4	9	-1.610, -1.105 9,8	4
**SER:173:A**	2	0.461, 2.070 4,1	5	-0.678, 0.530 7,4	3
**CYS:174:A**	3	-0.114, 1.520 5,2	9	-1.722, -1.172 9,9	6
GLY:175:A	6	-1.110, 0.009 8,5	6	-0.968, 0.089 8,5	0
SER:176:A	8	-1.753, -1.110 9,8	9	-1.610, -1.037 9,8	1
PRO:177:A	4	-0.114, 1.520 5,2	4	-0.031, 1.584 5,3	0
ASN:178:A	4	-0.114, 1.157 5,3	4	-0.243, 1.218 6,3	0
TYR:179:A	7	-1.415, -0.622 8,6	8	-1.448, -0.678 9,7	1
ALA:180:A	7	-1.187, -0.437 8,6	8	-1.377, -0.678 9,7	1
ALA:181:A	9	-1.861, -1.338 9,8	8	-1.307, -0.678 9,7	-1
PRO:182:A	8	-1.861, -1.110 9,8	8	-1.610, -0.827 9,8	0
GLU:183:A	9	-2.015, -1.574 9,9	8	-1.448, -0.753 9,7	-1
VAL:184:A	5	-0.711, 0.461 7,4	5	-0.753, 0.219 7,5	0
ILE:185:A	6	-1.110, -0.114 8,5	7	-1.172, -0.340 9,6	1
SER:186:A	4	-0.335, 1.157 6,3	4	-0.431, 0.942 6,4	0
GLY:187:A	3*	0.145, 2.070 5,1	5	-0.678, 0.530 7,4	
ARG:188:A	4	-0.228, 1.157 6,3	3	0.219, 1.584 5,3	-1
LEU:189:A	6	-0.876, 0.145 7,5	4	-0.141, 1.218 5,3	-2
TYR:190:A	7	-1.415, -0.531 8,6	8	-1.448, -0.753 9,7	1
ALA:191:A	5*	-0.956, 0.461 7,4	2	0.530, 2.137 4,2	
GLY:192:A	5	-0.711, 0.461 7,4	8*	-1.448, -0.516 9,7	
PRO:193:A	2	0.652, 3.457 4,1	2	0.718, 3.551 4,1	0
GLU:194:A	4	-0.335, 1.157 6,3	4	-0.340, 0.942 6,4	0
VAL:195:A	7	-1.338, -0.437 8,6	6	-0.827, 0.089 8,5	-1
ASP:196:A	9	-2.015, -1.415 9,8	9	-1.722, -1.172 9,9	0
ILE:197:A	6	-0.956, -0.114 7,5	4	-0.243, 1.218 6,3	-2
TRP:198:A	7	-1.262, -0.335 8,6	8	-1.610, -0.899 9,8	1
SER:199:A	9	-2.015, -1.493 9,9	9	-1.610, -1.105 9,8	0
CYS:200:A	5	-0.795, 0.293 7,4	4	-0.243, 1.218 6,3	-1
GLY:201:A	8	-1.659, -0.795 9,7	9	-1.610, -0.899 9,8	1

VAL:202:A	6	-0.876, 0.145 7,5	8	-1.377, -0.678 9,7	2
ILE:203:A	5	-0.795, 0.293 7,4	7	-1.105, -0.141 8,5	2
LEU:204:A	7	-1.415, -0.622 8,6	8	-1.525, -0.899 9,8	1
**TYR:205:A**	4	-0.228, 1.157 6,3	7	-1.172, -0.243 9,6	3
ALA:206:A	9	-1.861, -1.415 9,8	6	-0.968, -0.031 8,5	-3
LEU:207:A	7	-1.187, -0.335 8,6	8	-1.377, -0.678 9,7	1
**LEU:208:A**	4	-0.437, 0.877 6,3	7	-1.172, -0.340 9,6	3
CYS:209:A	7	-1.338, -0.622 8,6	5	-0.431, 0.718 6,4	-2
GLY:210:A	7	-1.262, -0.335 8,6	9	-1.610, -0.899 9,8	2
THR:211:A	3*	0.145, 2.070 5,1	3	0.219, 2.137 5,2	
LEU:212:A	3	0.009, 1.520 5,2	4	-0.431, 0.942 6,4	1
PRO:213:A	8	-1.753, -0.956 9,7	8	-1.610, -0.827 9,8	0
PHE:214:A	7	-1.187, -0.228 8,6	9	-1.722, -1.172 9,9	2
ASP:215:A	4	-0.114, 1.157 5,3	5	-0.753, 0.365 7,4	1
ASP:216:A	5	-0.795, 0.461 7,4	4	-0.243, 1.218 6,3	-1
GLU:217:A	3	-0.114, 1.520 5,2	3	0.530, 2.137 4,2	0
HIS:218:A	3	0.009, 1.520 5,2	5	-0.431, 0.718 6,4	2
VAL:219:A	3	-0.114, 1.520 5,2	2	0.530, 3.551 4,1	-1
PRO:220:A	5	-0.795, 0.461 7,4	2	0.530, 3.551 4,1	-3
THR:221:A	7	-1.493, -0.711 9,7	5	-0.599, 0.530 7,4	-2
LEU:222:A	7*	-1.574, -0.335 9,6	7	-1.240, -0.340 9,6	
PHE:223:A	4	-0.114, 1.157 5,3	4	-0.031, 1.584 5,3	0
LYS:224:A	4	-0.335, 1.157 6,3	4	-0.141, 1.218 5,3	0
LYS:225:A	3*	0.009, 2.070 5,1	5	-0.516, 0.718 7,4	
**ILE:226:A**	6	-1.110, -0.228 8,6	9	-1.610, -1.105 9,8	3
ARG:227:A	5	-0.622, 0.652 6,4	2	0.718, 3.551 4,1	-3
GLY:228:A	3	0.145, 1.520 5,2	4	-0.141, 1.218 5,3	1
**GLY:229:A**	3	0.009, 1.520 5,2	7	-1.105, -0.141 8,5	4
VAL:230:A	3*	-0.114, 2.070 5,1	3	0.089, 2.137 5,2	
PHE:231:A	3*	-0.114, 2.070 5,1	5	-0.599, 0.530 7,4	
TYR:232:A	2	0.461, 2.070 4,1	2	0.530, 3.551 4,1	0
ILE:233:A	2	0.293, 2.070 4,1	2	0.530, 3.551 4,1	0
PRO:234:A	4	-0.114, 1.520 5,2	6	-0.899, 0.219 8,5	2

GLU:235:A	3	-0.114, 1.520 5,2	4	-0.340, 0.942 6,4	1
TYR:236:A	4	-0.437, 1.157 6,3	1	1.218, 3.551 3,1	-3
LEU:237:A	5	-0.876, 0.293 7,4	5	-0.678, 0.530 7,4	0
ASN:238:A	6	-0.876, 0.145 7,5	7	-1.172, -0.431 9,6	1
ARG:239:A	6	-1.110, -0.114 8,5	2	0.718, 3.551 4,1	-4
SER:240:A	4	-0.228, 1.157 6,3	3	0.089, 1.584 5,3	-1
VAL:241:A	5	-0.711, 0.461 7,4	7	-1.105, -0.340 8,6	2
ALA:242:A	1	0.877, 3.457 3,1	3	0.219, 2.137 5,2	2
THR:243:A	3	-0.114, 1.520 5,2	4	-0.243, 1.218 6,3	1
LEU:244:A	7	-1.187, -0.335 8,6	9	-1.722, -1.105 9,8	2
LEU:245:A	7	-1.187, -0.335 8,6	6	-0.827, 0.219 8,5	-1
MET:246:A	4	-0.228, 1.157 6,3	4	-0.141, 1.218 5,3	0
HIS:247:A	4	-0.114, 1.520 5,2	2	0.942, 3.551 4,1	-2
MET:248:A	7	-1.262, -0.228 8,6	8	-1.448, -0.827 9,8	1
LEU:249:A	6	-1.034, 0.009 7,5	7	-1.172, -0.243 9,6	1
GLN:250:A	5	-0.711, 0.293 7,4	4	-0.431, 0.718 6,4	-1
**VAL:251:A**	1	1.157, 3.457 3,1	8	-1.307, -0.599 9,7	7
ASP:252:A	6	-0.956, -0.114 7,5	7	-1.240, -0.431 9,6	1
PRO:253:A	7	-1.262, -0.228 8,6	7	-1.240, -0.340 9,6	0
LEU:254:A	4	-0.437, 0.877 6,3	2	0.718, 3.551 4,1	-2
LYS:255:A	4	-0.437, 0.652 6,4	5	-0.753, 0.365 7,4	1
ARG:256:A	9	-2.015, -1.493 9,9	9	-1.722, -1.172 9,9	0
ALA:257:A	6	-0.876, 0.293 7,4	5	-0.753, 0.530 7,4	-1
THR:258:A	8	-1.493, -0.795 9,7	6	-0.968, -0.031 8,5	-2
ILE:259:A	5	-0.876, 0.293 7,4	4	-0.243, 1.218 6,3	-1
LYS:260:A	6	-0.876, 0.145 7,5	3	0.089, 1.584 5,3	-3
ASP:261:A	6	-0.876, 0.145 7,5	3	0.089, 1.584 5,3	-3
ILE:262:A	7	-1.493, -0.711 9,7	7	-1.172, -0.431 9,6	0
ARG:263:A	3*	0.009, 2.070 5,1	4	-0.141, 1.218 5,3	
GLU:264:A	4	-0.335, 1.157 6,3	2	0.718, 3.551 4,1	-2
**HIS:265:A**	6	-1.110, 0.009 8,5	9	-1.525, -1.037 9,8	3
GLU:266:A	6	-1.110, 0.145 8,5	1	1.584, 3.551 3,1	-5
TRP:267:A	6	-1.338, 0.009 8,5	7	-1.307, -0.431 9,6	1

PHE:268:A	6	-0.876, 0.145 7,5	3	0.530, 2.137 4,2	-3

Table S2. Kinase residue conservation scores calculated based sequence similarity based on in vitro kinase activity data of BAY-3827-inhibited and non-inhibited kinases. In vitro kinase selectivity data at 1 μM was cross-referenced with past data *(38)* and BAY-3827-inhibited and non-inhibited kinase groups were selected with shown calculated percentage identity (PID) values (%) based on a sequence alignment of selected group kinases in Jalview *(69)* with AMPKα1 as the reference. Subsequent ConSurf *(41,42)* residue conservation scores (1-9) were calculated, where * represents low confidence scoring and the calculated score change (Δ) between groups.

ENSEMBL_ID	Gene Name	logFC	Average log2 CPM project wide	PValue	FDR
ENSMUSG00000024998	*PLCE1*	-1.0376636	6.1364141	1.5755E-30	2.3135E-26
ENSMUSG00000019849	*PREP*	0.72113849	6.46925708	6.8589E-30	5.0358E-26
ENSMUSG00000044786	*ZFP36*	-0.98901986	5.10259272	1.4218E-26	5.2196E-23
ENSMUSG00000022178	*AJUBA*	1.21427328	7.0971376	1.2957E-26	5.2196E-23
ENSMUSG00000035199	*ARL6IP5*	0.80166259	6.80604434	2.9168E-26	8.5661E-23
ENSMUSG00000021097	*CLMN*	-0.94812875	5.97694898	3.9232E-26	9.6014E-23
ENSMUSG00000053175	*BCL3*	2.01140047	6.07145752	4.9336E-26	1.0349E-22
ENSMUSG00000036103	*COLEC12*	-0.93499223	5.81950357	6.2547E-26	1.148E-22
ENSMUSG00000021190	*LGMN*	-0.5214791	6.43670262	1.1455E-24	1.869E-21
ENSMUSG00000015711	*PRUNE1*	-0.62966503	6.21764823	1.5833E-24	2.325E-21
ENSMUSG00000025034	*TRIM8*	0.63897579	7.22457462	5.6728E-24	6.9416E-21
ENSMUSG00000059552	*TRP53*	0.72757854	6.94964976	5.3791E-24	6.9416E-21
ENSMUSG00000095440	*FIGNL2*	-1.06689849	3.74565927	8.9343E-24	1.0092E-20
ENSMUSG00000015850	*ADAMTSL4*	-1.41932317	2.49986922	1.0352E-23	1.0858E-20
ENSMUSG00000038034	*IGSF8*	-0.84958208	5.91679914	1.4026E-23	1.3731E-20
ENSMUSG00000070392		2.66614355	3.02376431	1.5714E-23	1.4421E-20
ENSMUSG00000016664	*PACSIN2*	0.61737749	8.08515711	1.8915E-23	1.6338E-20
ENSMUSG00000063015	*CCNI*	-0.4505774	7.17590155	2.5117E-23	2.049E-20
ENSMUSG00000030254	*RAD18*	-0.78049198	4.0562406	3.2463E-23	2.5089E-20
ENSMUSG00000021036	*SPTLC2*	-0.54711166	6.87366188	4.5006E-23	3.3044E-20
ENSMUSG00000019996	*MAP7*	-0.64390205	5.58174531	5.4261E-23	3.7942E-20
ENSMUSG00000053774	*UBXN7*	-0.74394788	4.89021768	7.2614E-23	4.8467E-20
ENSMUSG00000020044	*TIMP3*	-1.5860914	2.18831585	8.7141E-23	5.5634E-20
ENSMUSG00000055148	*KLF2*	2.80823536	1.99385144	9.9621E-23	6.0952E-20
ENSMUSG00000078622	*CCDC47*	0.51110458	7.18106032	1.1067E-22	6.2989E-20
ENSMUSG00000036501	*FAM13B*	0.94342825	5.20532629	1.1153E-22	6.2989E-20
ENSMUSG00000024479	*MAL2*	0.99204662	6.3769454	1.5147E-22	8.2379E-20
ENSMUSG00000042699	*DHX9*	0.56158856	6.74358803	1.673E-22	8.7738E-20
ENSMUSG00000030842	*LAMTOR1*	0.41420023	6.25866033	5.726E-22	2.8993E-19

ENSMUSG00000063888	*RPL7L1*	0.50268668	6.54363455	6.1018E-22	2.9866E-19
ENSMUSG00000018932		0.65966332	6.90229429	6.3884E-22	3.0261E-19
ENSMUSG00000004446	*BID*	0.78172013	5.61413192	1.0039E-21	4.6068E-19
ENSMUSG00000059436	*MAX*	0.63995007	5.70026135	1.1079E-21	4.9296E-19
ENSMUSG00000045975	*C2CD2*	-0.58117452	5.84154337	1.6642E-21	7.1874E-19
ENSMUSG00000022884	*EIF4A2*	-0.87648095	7.77785005	2.1117E-21	8.8593E-19
ENSMUSG00000030655	*SMG1*	-0.85193766	6.89707996	2.4716E-21	1.0082E-18
ENSMUSG00000033416	*GUCD1*	-0.78128549	7.29972588	2.7274E-21	1.0539E-18
ENSMUSG00000020538	*SREBF1*	-0.53912806	6.62173403	2.7112E-21	1.0539E-18
ENSMUSG00000020307	*CDC34*	0.44581627	7.53714468	3.3149E-21	1.2481E-18
ENSMUSG00000042851	*ZC3H6*	-1.58988901	1.47881656	5.1438E-21	1.8883E-18
ENSMUSG00000015759	*CNIH1*	0.44826478	6.47944754	5.8319E-21	2.0887E-18
ENSMUSG00000036181	*H1F2*	2.06900436	6.79201038	6.1133E-21	2.1373E-18
ENSMUSG00000021638	*OCLN*	-0.70108409	5.0982124	9.6062E-21	3.2804E-18
ENSMUSG00000000555	*ITGA5*	-0.62763188	5.03591296	1.0542E-20	3.5183E-18
ENSMUSG00000011179	*ODC1*	0.50712809	8.39180856	1.1208E-20	3.6574E-18
ENSMUSG00000062937	*MTAP*	0.58552631	6.05146889	1.4417E-20	4.6023E-18
ENSMUSG00000011305	*PLIN5*	-1.29956942	3.81374286	1.8119E-20	5.6609E-18
ENSMUSG00000028179	*CTH*	0.69391708	6.41882074	1.858E-20	5.6838E-18
ENSMUSG00000030541	*IDH2*	-0.71675707	4.89175875	1.9483E-20	5.7217E-18
ENSMUSG00000063972	*NR6A1*	1.03710582	4.39121138	1.9175E-20	5.7217E-18
ENSMUSG00000042328	*HPS4*	-0.87650943	4.19471863	1.9946E-20	5.743E-18
ENSMUSG00000036078	*SIGMAR1*	0.43583938	7.33434621	2.04E-20	5.7606E-18
ENSMUSG00000001910	*NACC1*	0.44341222	7.15010269	3.0531E-20	8.4587E-18
ENSMUSG00000037321	*TAP1*	-0.74770704	5.74412352	3.3035E-20	8.983E-18
ENSMUSG00000090100	*TTBK2*	-1.16857568	3.52306033	3.3655E-20	8.9853E-18
ENSMUSG00000020919	*STAT5B*	-0.6006035	6.05375795	3.5546E-20	9.1572E-18
ENSMUSG00000021131	*ERH*	0.54723094	5.50554605	3.5281E-20	9.1572E-18
ENSMUSG00000056493	*FOXK1*	-0.5924171	6.12138984	4.1455E-20	1.0182E-17
ENSMUSG00000058355	*ABCE1*	0.40761857	7.59557518	4.1604E-20	1.0182E-17

ENSMUSG00000054484	*TMEM62*	0.60538119	4.92239444	4.1124E-20	1.0182E-17
ENSMUSG00000006920	*EZH1*	-0.47255417	5.61201344	4.272E-20	1.0284E-17
ENSMUSG00000022185	*ACIN1*	0.56955819	6.70632556	4.4065E-20	1.0436E-17
ENSMUSG00000026864	*HSPA5*	1.01298563	10.3698351	4.8195E-20	1.1168E-17
ENSMUSG00000055116	*BMAL1*	1.04204188	4.41504119	4.8675E-20	1.1168E-17
ENSMUSG00000030643	*RAB30*	-1.45315546	3.44214233	5.0728E-20	1.146E-17
ENSMUSG00000008855	*HDAC5*	-0.70369853	5.8150903	5.1514E-20	1.1461E-17
ENSMUSG00000028676	*SRSF10*	0.44790961	5.77306306	5.5112E-20	1.2079E-17
ENSMUSG00000001751	*NAGLU*	-0.53170956	5.69831054	6.1779E-20	1.3341E-17
ENSMUSG00000044646	*ZBTB7C*	2.39023463	2.75119167	6.9819E-20	1.4858E-17
ENSMUSG00000028069	*GPATCH4*	0.63557406	6.55114859	7.887E-20	1.6545E-17
ENSMUSG00000031657	*HEATR3*	0.62976992	5.56314034	8.5352E-20	1.7652E-17
ENSMUSG00000004535	*TAX1BP1*	-0.37962091	7.51390734	8.8768E-20	1.7856E-17
ENSMUSG00000012535	*TNPO3*	0.40353612	6.83620839	9.6467E-20	1.9142E-17
ENSMUSG00000022641	*BBX*	-0.94554588	5.54254662	1.0095E-19	1.9269E-17
ENSMUSG00000068587	*MGAM*	-0.79149252	4.88878755	1.0104E-19	1.9269E-17
ENSMUSG00000048878	*HEXIM1*	1.18762857	4.1961273	9.9431E-20	1.9269E-17
ENSMUSG00000029438	*BCL7A*	-0.92891951	3.61080076	1.0361E-19	1.9277E-17
ENSMUSG00000038722	*BUD31*	0.40108141	5.79367351	1.0371E-19	1.9277E-17
ENSMUSG00000035069	*OMA1*	-0.72799355	4.09994207	1.0536E-19	1.9338E-17
ENSMUSG00000008090	*FGFRL1*	-0.65690005	4.36731552	1.0827E-19	1.9627E-17
ENSMUSG00000038503	*MESD*	0.58938611	5.34269098	1.1524E-19	2.0636E-17
ENSMUSG00000007812	*ZFP655*	-0.50965545	5.85035622	1.2087E-19	2.1384E-17
ENSMUSG00000003269	*CYTH2*	-0.57265321	5.72478282	1.4307E-19	2.4949E-17
ENSMUSG00000038482	*TFDP1*	0.4929127	6.0630939	1.4442E-19	2.4949E-17
ENSMUSG00000025178	*PI4K2A*	-0.5221872	6.35946797	1.5706E-19	2.6818E-17
ENSMUSG00000022853	*EHHADH*	-1.59549669	6.67291873	1.6538E-19	2.7913E-17
ENSMUSG00000002831	*PLIN4*	-1.95351379	3.90987601	1.7081E-19	2.8502E-17
ENSMUSG00000041355	*SSR2*	0.43690848	6.68246248	1.8099E-19	2.9861E-17
ENSMUSG00000035757	*SELENOO*	-0.614501	5.00514082	2.3656E-19	3.8597E-17

ENSMUSG00000015994	*FNTA*	0.52496767	5.09238584	2.5829E-19	4.1225E-17
ENSMUSG00000040297	*SUCO*	-0.67210468	5.67124049	2.6365E-19	4.1628E-17
ENSMUSG00000030685	*KCTD13*	-0.7767568	4.00332542	2.7504E-19	4.2964E-17
ENSMUSG00000024014	*PIM1*	-1.08633823	5.50536623	3.4551E-19	5.3316E-17
ENSMUSG00000053819	*CAMK2D*	-0.80604639	5.3561782	3.4856E-19	5.3316E-17
ENSMUSG00000028741	*MRTO4*	0.48922847	5.70857273	3.5263E-19	5.3382E-17
ENSMUSG00000048826	*DACT2*	-0.66976769	4.60601508	3.7708E-19	5.65E-17
ENSMUSG00000032531	*AMOTL2*	0.77568511	5.98479136	3.8882E-19	5.7094E-17
ENSMUSG00000026036	*NIF3L1*	0.6483248	4.90870749	4.0859E-19	5.9404E-17
ENSMUSG00000020180	*SNRPD3*	0.41568268	5.84836068	4.6592E-19	6.7075E-17
ENSMUSG00000002658	*GTF2F1*	0.44657932	6.280007	4.8641E-19	6.8678E-17
ENSMUSG00000053841	*TXLNA*	0.4728115	6.08310426	4.8397E-19	6.8678E-17
ENSMUSG00000052397	*EZR*	1.26709808	4.80478033	5.4045E-19	7.5581E-17
ENSMUSG00000053411	*CBX7*	-0.7547906	4.44437809	5.5916E-19	7.7459E-17
ENSMUSG00000028382	*PTBP3*	-0.47111	6.88349803	6.5843E-19	9.0359E-17
ENSMUSG00000041688	*AMOT*	-1.09948453	3.57583179	6.8769E-19	9.3439E-17
ENSMUSG00000025355	*MMP19*	0.96379434	4.8683947	6.936E-19	9.3439E-17
ENSMUSG00000038520	*TBC1D17*	-0.55983577	5.72031897	7.2863E-19	9.7265E-17
ENSMUSG00000060373	*HNRNPC*	0.48199166	7.15870306	7.4651E-19	9.8754E-17
ENSMUSG00000039529	*ATP8B1*	-0.88111411	4.61297233	8.0768E-19	1.0589E-16
ENSMUSG00000047246	*H2BC6*	2.38808537	1.240064	8.4257E-19	1.0949E-16
ENSMUSG00000040550	*OTUD6B*	0.44653233	6.01921855	1.0332E-18	1.3309E-16
ENSMUSG00000030967	*ZRANB1*	-0.65150442	5.79945514	1.1257E-18	1.4373E-16
ENSMUSG00000007216	*ZFP775*	-0.98940109	2.73881376	1.2403E-18	1.57E-16
ENSMUSG00000021703	*SERINC5*	-0.6243496	6.48237455	1.2752E-18	1.6004E-16
ENSMUSG00000045193	*CIRBP*	-0.85232614	3.72583323	1.3504E-18	1.6805E-16
ENSMUSG00000047098	*RNF31*	-0.55405477	5.86836344	1.3994E-18	1.7268E-16
ENSMUSG00000025509	*PNPLA2*	-0.75291603	7.06493215	1.5403E-18	1.8848E-16
ENSMUSG00000031016	*WEE1*	-1.44184783	4.37454734	1.6217E-18	1.968E-16
ENSMUSG00000022032	*SCARA5*	-2.71454558	1.44295687	1.6817E-18	2.0241E-16

ENSMUSG00000004508	*GAB2*	-0.94622113	4.33735096	1.8068E-18	2.157E-16
ENSMUSG00000024640	*PSAT1*	0.78396563	7.25209673	2.0265E-18	2.3998E-16
ENSMUSG00000053931	*CNN3*	0.42800539	8.09858051	2.0595E-18	2.4193E-16
ENSMUSG00000078851	*H2AC25*	1.17306583	2.98851003	2.1026E-18	2.4504E-16
ENSMUSG00000046447	*CAMK2N1*	-0.59209752	5.66470288	2.167E-18	2.5055E-16
ENSMUSG00000040661	*RAD54L2*	-0.61062101	5.87058687	2.275E-18	2.6098E-16
ENSMUSG00000029571	*TMEM106B*	-0.49807935	7.52610431	2.3817E-18	2.7111E-16
ENSMUSG00000042599	*KDM7A*	-0.85286761	3.95632431	2.4009E-18	2.7119E-16
ENSMUSG00000024943	*SMC5*	-0.51301628	5.05777205	2.6011E-18	2.9156E-16
ENSMUSG00000032220	*MYO1E*	-0.76754179	6.03941167	2.7899E-18	3.1036E-16
ENSMUSG00000035834	*POLR3G*	0.61021235	4.69422185	2.857E-18	3.1543E-16
ENSMUSG00000019838	*SLC16A10*	-0.88311912	7.5495033	2.9757E-18	3.2368E-16
ENSMUSG00000031403	*DKC1*	0.76018953	5.72721529	2.9758E-18	3.2368E-16
ENSMUSG00000030555	*TTC23*	-0.72721684	4.86255263	3.2544E-18	3.5138E-16
ENSMUSG00000026656	*FCGR2B*	-1.79524009	1.79217696	3.295E-18	3.5317E-16
ENSMUSG00000034349	*SMC4*	-0.74380595	3.85311208	3.5375E-18	3.7641E-16
ENSMUSG00000032253	*PHIP*	-0.86888607	3.96220984	3.5897E-18	3.7922E-16
ENSMUSG00000106775		-4.323407	-0.29803059	3.8221E-18	4.0089E-16
ENSMUSG00000025134	*ALYREF*	0.55561058	4.80287958	3.9841E-18	4.1491E-16
ENSMUSG00000026435	*SLC45A3*	-0.80731562	3.79605383	4.6852E-18	4.8449E-16
ENSMUSG00000045441	*GPRIN3*	-1.04140715	3.56618226	4.7937E-18	4.9225E-16
ENSMUSG00000015305	*SASH1*	-1.28871276	5.13220447	4.8726E-18	4.9687E-16
ENSMUSG00000020448	*RNF185*	0.76627446	5.88169601	5.0079E-18	5.0715E-16
ENSMUSG00000021775	*NR1D2*	-0.77897075	5.7427279	5.6904E-18	5.7231E-16
ENSMUSG00000079104	*PRPS1L3*	0.59603911	5.37037196	5.8755E-18	5.8691E-16
ENSMUSG00000027405	*NOP56*	0.90467326	7.74064569	5.9869E-18	5.94E-16
ENSMUSG00000021271	*ZFP839*	-0.70619695	3.69092261	6.0505E-18	5.9628E-16
ENSMUSG00000029594	*RBM19*	0.54157	5.79726066	6.0947E-18	5.9663E-16
ENSMUSG00000038335	*TSR1*	0.49485734	6.87806085	6.3247E-18	6.1504E-16
ENSMUSG00000055013	*AGAP1*	0.50978989	6.05042754	6.5083E-18	6.2874E-16

ENSMUSG00000042111	*CCDC115*	0.70126955	4.5146054	6.9316E-18	6.6093E-16
ENSMUSG00000045211	*NUDT18*	-0.73769126	5.57228936	7.9741E-18	7.5543E-16
ENSMUSG00000026558	*UCK2*	0.53646432	5.97001823	8.7006E-18	8.1897E-16
ENSMUSG00000058152	*CHSY3*	-1.06395479	3.83001985	9.3835E-18	8.7763E-16
ENSMUSG00000024007	*PPIL1*	0.67967989	4.46271029	9.5061E-18	8.8347E-16
ENSMUSG00000028603	*SCP2*	-0.55406116	8.93093032	1.0382E-17	9.5879E-16
ENSMUSG00000039879	*HECA*	-0.76798066	5.55016992	1.0716E-17	9.8349E-16
ENSMUSG00000058013	*SEPTIN11*	0.52773634	6.4175161	1.0895E-17	9.9365E-16
ENSMUSG00000024193	*PHF1*	-0.64495467	4.25733167	1.1995E-17	1.0872E-15
ENSMUSG00000008373	*PRPF31*	0.49161613	5.85772641	1.276E-17	1.1495E-15
ENSMUSG00000005481	*DDX39A*	0.54927263	6.19052889	1.3559E-17	1.212E-15
ENSMUSG00000020142	*SLC1A4*	0.92564468	6.01065387	1.3619E-17	1.212E-15
ENSMUSG00000039220	*PPP1R10*	0.88921684	5.42428468	1.3995E-17	1.2379E-15
ENSMUSG00000074797	*ITPA*	0.67717163	4.91395825	1.5675E-17	1.3783E-15
ENSMUSG00000050423	*PPP1R3G*	-2.30664182	0.21313911	1.621E-17	1.4168E-15
ENSMUSG00000020130	*TBC1D15*	-0.48953674	6.18860808	1.6929E-17	1.4709E-15
ENSMUSG00000023073	*SLC10A2*	-1.39094097	4.95633538	1.7605E-17	1.5207E-15
ENSMUSG00000021127	*ZFP36L1*	-0.72984172	7.93611654	1.7947E-17	1.5412E-15
ENSMUSG00000003752	*ITPKC*	-0.77320808	5.91125903	1.8131E-17	1.5456E-15
ENSMUSG00000033589	*REEP4*	-0.72899308	4.82561581	1.8277E-17	1.5456E-15
ENSMUSG00000029833	*TRIM24*	-0.53906881	4.67004611	1.8314E-17	1.5456E-15
ENSMUSG00000021737	*PSMD6*	0.4172561	6.21510593	1.9464E-17	1.6332E-15
ENSMUSG00000018583	*G3BP1*	0.48268635	7.49063327	1.9949E-17	1.6644E-15
ENSMUSG00000034543	*MORC2A*	0.69024557	4.734025	2.2878E-17	1.8873E-15
ENSMUSG00000029446	*PSPH*	0.71963902	5.05328565	2.2784E-17	1.8873E-15
ENSMUSG00000037070	*RBMXL1*	0.56630521	5.11764985	2.4522E-17	2.0117E-15
ENSMUSG00000026456	*CYB5R1*	0.91280473	6.42975306	2.511E-17	2.0484E-15
ENSMUSG00000027875	*HMGCS2*	-1.81734037	6.33818772	2.82E-17	2.2752E-15
ENSMUSG00000021266	*WARS1*	0.53461705	6.17386458	2.8053E-17	2.2752E-15
ENSMUSG00000029610	*AIMP2*	0.41995898	5.82157137	3.1864E-17	2.5429E-15

ENSMUSG00000040620	*DHX33*	0.53662981	6.07524656	3.236E-17	2.5685E-15
ENSMUSG00000002409	*DYRK1B*	-1.36187267	3.65494098	3.3214E-17	2.6222E-15
ENSMUSG00000000826	*DNAJC5*	0.38991526	8.32545736	3.4311E-17	2.6943E-15
ENSMUSG00000009013	*DYNLL1*	0.53108178	6.28230324	3.6355E-17	2.8396E-15
ENSMUSG00000056612	*PPP1R14B*	0.39052169	6.73558161	4.0038E-17	3.0943E-15
ENSMUSG00000031826	*USP10*	0.4370136	6.93199492	3.988E-17	3.0943E-15
ENSMUSG00000015522	*ARNT*	-0.52609436	6.16187389	4.1774E-17	3.1949E-15
ENSMUSG00000074746	*PDZD8*	0.50267116	6.40086804	4.1656E-17	3.1949E-15
ENSMUSG00000025198	*ERLIN1*	0.70284666	5.76900183	4.2057E-17	3.1998E-15
ENSMUSG00000032000	*BIRC3*	0.55517155	4.66620629	4.23E-17	3.2017E-15
ENSMUSG00000022389	*TEF*	-0.66957513	5.44215732	4.2717E-17	3.2167E-15
ENSMUSG00000020964	*SEL1L*	0.42694709	6.93312558	4.4848E-17	3.3599E-15
ENSMUSG00000032252	*GLCE*	0.63625447	5.82917021	5.007E-17	3.7321E-15
ENSMUSG00000025795	*RASSF3*	0.54893587	6.68882532	5.1211E-17	3.7979E-15
ENSMUSG00000030528	*BLM*	-1.41249955	1.96395084	5.3185E-17	3.9245E-15
ENSMUSG00000030465	*PSD3*	-0.78433607	5.25691871	5.4629E-17	4.0109E-15
ENSMUSG00000041037	*IRGQ*	0.53496294	5.68849133	5.6861E-17	4.154E-15
ENSMUSG00000019944	*RHOBTB1*	-1.04933878	6.50323971	5.7899E-17	4.2089E-15
ENSMUSG00000022607	*PTK2*	0.38474706	5.99111358	5.8849E-17	4.2153E-15
ENSMUSG00000038506	*DCUN1D2*	0.6617975	4.47483687	5.8331E-17	4.2153E-15
ENSMUSG00000034889	*CACTIN*	0.71023328	5.54936104	5.8698E-17	4.2153E-15
ENSMUSG00000079111	*KDELR2*	0.39201365	6.56950207	6.3711E-17	4.5414E-15
ENSMUSG00000039356	*EXOSC2*	0.5534107	5.06334834	7.1537E-17	5.0746E-15
ENSMUSG00000024772	*EHD1*	0.46406572	7.36735477	7.5092E-17	5.3012E-15
ENSMUSG00000029669	*TSPAN12*	-0.50053614	6.41203722	7.593E-17	5.304E-15
ENSMUSG00000068856	*SF3B4*	0.49706592	6.56973166	7.6216E-17	5.304E-15
ENSMUSG00000053907	*MAT2A*	0.5154912	7.26886337	7.5805E-17	5.304E-15
ENSMUSG00000037300	*TTC13*	0.48732433	6.02475921	7.8701E-17	5.4512E-15
ENSMUSG00000048039	*ISG20L2*	0.57548278	4.98959634	8.2911E-17	5.7158E-15
ENSMUSG00000019432	*DDX39B*	0.55050729	7.1406641	8.4906E-17	5.826E-15

ENSMUSG00000020653	*KLF11*	-0.94891208	4.3414445	8.5768E-17	5.8578E-15
ENSMUSG00000042487	*LEO1*	0.55505115	5.28304998	8.9903E-17	6.0836E-15
ENSMUSG00000018848	*RARS*	0.5113642	7.13055994	9.1134E-17	6.1386E-15
ENSMUSG00000028410	*DNAJA1*	0.49215371	7.65280815	1.0103E-16	6.7431E-15
ENSMUSG00000039157	*EEIG1*	-1.05656423	5.59698312	1.0766E-16	7.1535E-15
ENSMUSG00000004319	*CLCN3*	0.52627678	6.1231034	1.1024E-16	7.2917E-15
ENSMUSG00000021810	*ECD*	0.39732525	5.87226693	1.2437E-16	8.1893E-15
ENSMUSG00000040146	*RGL3*	-0.6262741	4.08962436	1.2821E-16	8.3675E-15
ENSMUSG00000027995	*TLR2*	1.15129153	3.53209002	1.2965E-16	8.4241E-15
ENSMUSG00000028607	*CPT2*	-0.62062098	5.86696144	1.313E-16	8.4936E-15
ENSMUSG00000026475	*RGS16*	-2.90724574	2.33950791	1.34E-16	8.63E-15
ENSMUSG00000030313	*DENND5B*	-0.54211164	5.79253958	1.3485E-16	8.647E-15
ENSMUSG00000022809	*NR1I2*	-1.06634449	4.54417369	1.3875E-16	8.8003E-15
ENSMUSG00000029338	*ANTXR2*	0.60164595	5.84042973	1.3904E-16	8.8003E-15
ENSMUSG00000074063	*OSGIN1*	1.13965969	9.14040848	1.3881E-16	8.8003E-15
ENSMUSG00000024773	*ATG2A*	-0.87177576	7.10344993	1.4362E-16	9.0513E-15
ENSMUSG00000032245	*CLN6*	-1.07732727	3.53928277	1.4998E-16	9.4116E-15
ENSMUSG00000003348	*MOB3A*	0.66314712	5.49978095	1.5102E-16	9.4363E-15
ENSMUSG00000002546	*GOLGA2*	-0.39141522	6.19507083	1.687E-16	1.0496E-14
ENSMUSG00000017801	*MLX*	0.50178076	5.2368445	1.8604E-16	1.1478E-14
ENSMUSG00000081058	*H3C15*	5.39519878	0.10963222	1.8583E-16	1.1478E-14
ENSMUSG00000037343	*TAF2*	0.49291499	4.70943122	2.1655E-16	1.3249E-14
ENSMUSG00000040105	*PLPP6*	-0.62734894	3.92245975	2.2466E-16	1.3632E-14
ENSMUSG00000052934	*FBXO31*	-0.62283569	5.45653583	2.2816E-16	1.3787E-14
ENSMUSG00000025383	*IL23A*	2.70854412	0.67396256	2.3779E-16	1.431E-14
ENSMUSG00000029328	*HNRNPDL*	-0.39062047	6.41663364	2.6622E-16	1.5891E-14
ENSMUSG00000004815	*DGKQ*	-0.50954963	5.13960097	2.6911E-16	1.5998E-14
ENSMUSG00000055976	*CLDN23*	-1.71650613	1.25688124	2.8068E-16	1.6486E-14
ENSMUSG00000042289	*HSD3B7*	-0.48795651	7.09587854	2.7977E-16	1.6486E-14
ENSMUSG00000048799	*CEP120*	-0.5678304	5.13165262	2.8566E-16	1.6712E-14

ENSMUSG00000041084	*OSTC*	0.62727284	4.98577649	2.9214E-16	1.7023E-14
ENSMUSG00000038039	*GCC2*	-0.57187172	5.91528139	2.9523E-16	1.7135E-14
ENSMUSG00000024483	*ANKHD1*	-0.51866125	5.45136249	3.2E-16	1.85E-14
ENSMUSG00000051451	*CREBZF*	-0.46340722	5.18977418	3.2199E-16	1.8541E-14
ENSMUSG00000014859	*E2F4*	0.50261919	6.24829742	3.3301E-16	1.9101E-14
ENSMUSG00000039781	*CEP131*	-0.88605483	2.49464635	3.4641E-16	1.9793E-14
ENSMUSG00000032212	*SLTM*	0.65587993	6.16386169	3.6753E-16	2.0837E-14
ENSMUSG00000038481	*CDK19*	-0.91189158	3.26815122	4.0465E-16	2.2854E-14
ENSMUSG00000046722	*CDC42SE1*	0.44446657	5.88040619	4.0722E-16	2.291E-14
ENSMUSG00000000711	*RAB5B*	-0.39540978	6.78510656	4.2344E-16	2.3732E-14
ENSMUSG00000031734	*IRX3*	-1.41390188	2.09748356	4.7485E-16	2.6512E-14
ENSMUSG00000004642	*SLBP*	0.47126945	4.3244423	4.767E-16	2.6515E-14
ENSMUSG00000027597	*AHCY*	-0.46719502	10.0182972	4.8056E-16	2.6628E-14
ENSMUSG00000063659	*ZBTB18*	0.82203245	6.25179299	4.8491E-16	2.6768E-14
ENSMUSG00000041124	*MSANTD4*	0.46357617	5.30556983	4.9194E-16	2.7055E-14
ENSMUSG00000021112	*PALS1*	-0.49983723	4.96684833	5.9324E-16	3.2178E-14
ENSMUSG00000021417	*ECI2*	-0.49158388	5.94415913	5.9387E-16	3.2178E-14
ENSMUSG00000024777	*PPP2R5B*	-0.40485211	5.54451498	6.2251E-16	3.3483E-14
ENSMUSG00000028729	*EBNA1BP2*	0.43941064	6.59074425	6.2587E-16	3.3541E-14
ENSMUSG00000033099	*NOL12*	0.52701924	5.07662389	7.3796E-16	3.9404E-14
ENSMUSG00000022383	*PPARA*	-1.23494086	4.45763999	7.5823E-16	4.0198E-14
ENSMUSG00000021765	*FST*	-0.92186111	4.54560754	7.803E-16	4.1068E-14
ENSMUSG00000048310	*PSKH1*	-0.46805747	5.61411008	7.9161E-16	4.1514E-14
ENSMUSG00000062901	*KLHL24*	-1.3749394	5.30563512	8.1208E-16	4.2436E-14
ENSMUSG00000038872	*ZFHX3*	0.66058948	5.75926358	9.2543E-16	4.8188E-14
ENSMUSG00000031666	*RBL2*	-0.78123982	4.48714595	9.946E-16	5.1466E-14
ENSMUSG00000020088	*SAR1A*	0.42745931	6.47160489	9.9899E-16	5.1466E-14
ENSMUSG00000015837	*SQSTM1*	0.57811647	11.8853375	1.0005E-15	5.1466E-14
ENSMUSG00000022562	*OPLAH*	-0.62668905	6.00121801	1.0187E-15	5.2123E-14
ENSMUSG00000084128	*ESRP2*	-0.48680885	6.30608117	1.0511E-15	5.3591E-14

ENSMUSG00000031613	*HPGD*	-1.74500439	4.57392133	1.0916E-15	5.5463E-14
ENSMUSG00000020522	*MFAP3*	0.50463768	5.12146112	1.101E-15	5.5748E-14
ENSMUSG00000028857	*TMEM222*	0.49116792	4.73371433	1.1148E-15	5.6253E-14
ENSMUSG00000026425	*SRGAP2*	-0.49127243	5.49637678	1.123E-15	5.6471E-14
ENSMUSG00000034993	*VAT1*	-0.39648877	7.66488017	1.1383E-15	5.7046E-14
ENSMUSG00000031938	*4931406C07RIK*	0.6894663	5.71938297	1.2538E-15	6.2621E-14
ENSMUSG00000041959	*S100A10*	0.42800638	8.52560179	1.2694E-15	6.3185E-14
ENSMUSG00000053414	*HUNK*	-2.19239392	0.58727187	1.2881E-15	6.3898E-14
ENSMUSG00000038068	*RNF144B*	-0.61193309	7.11903828	1.3521E-15	6.6487E-14
ENSMUSG00000028330	*NCBP1*	0.40112886	5.98259986	1.3538E-15	6.6487E-14
ENSMUSG00000031393	*MECP2*	-0.57880955	4.30667121	1.3733E-15	6.722E-14
ENSMUSG00000026473	*GLUL*	-0.705595	6.08074567	1.4276E-15	6.9418E-14
ENSMUSG00000002052	*SUPT6*	0.46520251	6.70446226	1.4718E-15	7.1328E-14
ENSMUSG00000025935	*TRAM1*	0.41537949	7.21720985	1.4811E-15	7.1539E-14
ENSMUSG00000043091	*TUBA1C*	0.5402748	9.3157545	1.5196E-15	7.3159E-14
ENSMUSG00000001542	*ELL2*	-0.48367012	7.03675744	1.5311E-15	7.3473E-14
ENSMUSG00000024732	*CCDC86*	0.48382903	6.18318805	1.558E-15	7.4522E-14
ENSMUSG00000029407	*USO1*	0.39585686	7.0917365	1.6055E-15	7.6297E-14
ENSMUSG00000075232	*AMD1*	0.54895343	7.16744362	1.6289E-15	7.7157E-14
ENSMUSG00000026812	*TSC1*	-0.44572519	5.70456168	1.7357E-15	8.1951E-14
ENSMUSG00000039768	*DNAJC11*	0.42813765	6.66803855	1.7954E-15	8.4498E-14
ENSMUSG00000030779	*RBBP6*	0.4892097	6.47834695	1.9136E-15	8.9489E-14
ENSMUSG00000064373	*SELENOP*	-0.60657368	11.2891105	1.9692E-15	9.1518E-14
ENSMUSG00000002625	*AKAP8L*	-0.59525689	4.56024456	1.9858E-15	9.1988E-14
ENSMUSG00000014353	*TMEM87B*	-0.48259159	5.69824728	2.0182E-15	9.3193E-14
ENSMUSG00000006585	*CDT1*	-0.68949271	3.97348304	2.1435E-15	9.8362E-14
ENSMUSG00000005893	*NR2C2*	-0.44931744	5.14710929	2.1731E-15	9.9409E-14
ENSMUSG00000040964	*ARHGEF10L*	0.47315355	5.74508793	2.2119E-15	1.0087E-13
ENSMUSG00000026638	*IRF6*	-0.83556725	6.04288699	2.3168E-15	1.0533E-13
ENSMUSG00000013629	*CAD*	0.42757831	6.90958692	2.5229E-15	1.1399E-13

ENSMUSG00000015176	*NOLC1*	0.49873826	6.68347525	2.5393E-15	1.1438E-13
ENSMUSG00000028811	*YARS*	0.45630943	6.56904415	2.663E-15	1.1958E-13
ENSMUSG00000063954	*H2AC19*	1.08778757	3.94684076	2.7628E-15	1.2338E-13
ENSMUSG00000052593	*ADAM17*	0.40166434	5.12113417	2.8398E-15	1.2636E-13
ENSMUSG00000047777	*PHF13*	-0.62231644	4.37507116	2.9121E-15	1.2919E-13
ENSMUSG00000018750	*ZBTB4*	-0.46974438	5.1126022	3.15E-15	1.389E-13
ENSMUSG00000001366	*FBXO9*	0.64216213	4.6537147	3.1464E-15	1.389E-13
ENSMUSG00000056121	*FEZ2*	0.40032634	6.54329081	3.2777E-15	1.4367E-13
ENSMUSG00000026020	*NOP58*	0.46827555	6.88741887	3.2757E-15	1.4367E-13
ENSMUSG00000051007	*GATD1*	-0.60034435	3.95291783	3.4314E-15	1.4899E-13
ENSMUSG00000005442	*CIC*	-0.48596818	6.44329632	3.4396E-15	1.4899E-13
ENSMUSG00000038205	*PRKAB2*	-0.83437189	4.79233486	3.4767E-15	1.5015E-13
ENSMUSG00000024664	*FADS3*	1.08867569	6.65527492	3.7135E-15	1.5944E-13
ENSMUSG00000042246	*TMC7*	-0.929764	4.02741606	3.8032E-15	1.6282E-13
ENSMUSG00000017677	*WSB1*	0.69532297	4.5577923	3.8684E-15	1.6513E-13
ENSMUSG00000024331	*DSC2*	-0.68569262	6.65759813	4.1196E-15	1.7477E-13
ENSMUSG00000025241	*FYCO1*	-0.47157628	5.12077183	4.13E-15	1.7477E-13
ENSMUSG00000039804	*NCOA5*	0.6979598	5.24739044	4.1599E-15	1.7553E-13
ENSMUSG00000022360	*ATAD2*	-0.74681066	2.91966235	4.1995E-15	1.7669E-13
ENSMUSG00000024539	*PTPN2*	-0.58963199	4.56072577	4.2411E-15	1.7743E-13
ENSMUSG00000024165	*JPT2*	0.45197808	6.13227392	4.5223E-15	1.8865E-13
ENSMUSG00000026436	*ELK4*	-0.44285225	5.80474593	4.5423E-15	1.8895E-13
ENSMUSG00000029538	*SRSF9*	0.45648753	5.26263798	4.6463E-15	1.9273E-13
ENSMUSG00000061024	*RRS1*	0.3967903	6.21788524	4.7364E-15	1.9536E-13
ENSMUSG00000020027	*SOCS2*	1.57936123	4.22275396	5.093E-15	2.0831E-13
ENSMUSG00000036661	*DENND3*	-1.25887997	1.70261732	5.1446E-15	2.0948E-13
ENSMUSG00000036534	*SLC38A7*	0.44627194	6.39735071	5.157E-15	2.0948E-13
ENSMUSG00000033088	*TRIOBP*	-0.39593025	5.58599135	5.3033E-15	2.1335E-13
ENSMUSG00000030530	*FURIN*	-0.38256158	8.86887478	5.2768E-15	2.1335E-13
ENSMUSG00000048429	*TIMM29*	0.46197024	5.39350374	5.2945E-15	2.1335E-13

ENSMUSG00000020883	*FBXL20*	-0.60733018	5.66717661	5.5239E-15	2.2042E-13
ENSMUSG00000063480	*SNU13*	0.42544973	5.87539555	5.5236E-15	2.2042E-13
ENSMUSG00000039156	*STIM2*	0.44226729	5.44568995	5.9079E-15	2.351E-13
ENSMUSG00000049957	*CCDC137*	0.95930728	4.75445378	6.0581E-15	2.4043E-13
ENSMUSG00000026377	*NIFK*	0.48499852	5.91183383	6.1123E-15	2.4192E-13
ENSMUSG00000034203	*CHCHD4*	0.42911276	5.63370432	6.5111E-15	2.5632E-13
ENSMUSG00000055322	*TNS1*	-0.74442225	7.67033976	7.0584E-15	2.7522E-13
ENSMUSG00000026142	*RHBDD1*	0.42250276	6.31322631	7.0589E-15	2.7522E-13
ENSMUSG00000037296	*LSM1*	0.55438861	3.99179894	7.066E-15	2.7522E-13
ENSMUSG00000038393	*TXNIP*	-2.22160738	4.7626927	7.2076E-15	2.7999E-13
ENSMUSG00000038459	*ABHD17C*	0.49338207	6.04584772	7.7992E-15	3.0217E-13
ENSMUSG00000028238	*ATP6V0D2*	-3.19543494	-0.83195461	8.1537E-15	3.1376E-13
ENSMUSG00000042790	*RNF214*	-0.53941113	4.25230901	8.1624E-15	3.1376E-13
ENSMUSG00000024036	*SLC37A1*	-1.23774586	1.69648894	8.7075E-15	3.3211E-13
ENSMUSG00000034430	*ZXDC*	-0.51960783	4.54895211	8.7475E-15	3.3277E-13
ENSMUSG00000025577	*CBX2*	-0.83582505	3.75224617	9.0309E-15	3.4229E-13
ENSMUSG00000041268	*DMXL2*	-0.38048286	5.52553473	9.0445E-15	3.4229E-13
ENSMUSG00000031819	*EMC8*	0.45063662	4.99804414	9.186E-15	3.4586E-13
ENSMUSG00000027881	*PRPF38B*	0.53960779	5.91584922	9.1826E-15	3.4586E-13
ENSMUSG00000059208	*HNRNPM*	0.45434058	7.13457172	9.3515E-15	3.5119E-13
ENSMUSG00000018068	*INTS2*	0.60396731	4.09909527	9.851E-15	3.6901E-13
ENSMUSG00000044340	*PHLPP1*	-0.52320762	4.75957085	1.0572E-14	3.94E-13
ENSMUSG00000025176	*HOGA1*	-0.55649658	4.91890285	1.136E-14	4.2215E-13
ENSMUSG00000003868	*RUVBL2*	0.46462793	5.41919839	1.1385E-14	4.2215E-13
ENSMUSG00000033022	*CDO1*	-1.01192657	9.31842368	1.1697E-14	4.3263E-13
ENSMUSG00000040822	*1700123O20RIK*	0.50405008	4.48846482	1.1917E-14	4.3967E-13
ENSMUSG00000035673	*SBNO2*	0.65158316	6.66960635	1.2034E-14	4.4289E-13
ENSMUSG00000019210	*ATP6V1E1*	-0.40859462	6.98240603	1.2147E-14	4.4593E-13
ENSMUSG00000029657	*HSPH1*	0.49883772	6.99867137	1.2514E-14	4.571E-13
ENSMUSG00000028809	*SRRM1*	-0.39932007	6.65623855	1.267E-14	4.6072E-13

ENSMUSG00000030397	*MARK4*	-0.41945741	5.72351771	1.2794E-14	4.6387E-13
ENSMUSG00000035824	*TK2*	-0.50092651	4.04319999	1.3065E-14	4.7251E-13
ENSMUSG00000040028	*ELAVL1*	0.41272379	6.08602101	1.371E-14	4.9465E-13
ENSMUSG00000040327	*CUL9*	-0.61222771	4.19799668	1.3808E-14	4.9694E-13
ENSMUSG00000039568	*UBALD1*	-0.62089696	6.02037412	1.4436E-14	5.1827E-13
ENSMUSG00000036775	*DECR2*	-0.45601813	5.28377104	1.6653E-14	5.9208E-13
ENSMUSG00000024883	*RIN1*	1.0843028	6.06935777	1.695E-14	5.9976E-13
ENSMUSG00000035623	*RSF1*	-0.60728718	4.28268318	1.7207E-14	6.0737E-13
ENSMUSG00000029387	*GTF2H3*	0.47923262	4.42387783	1.7939E-14	6.3168E-13
ENSMUSG00000017950	*HNF4A*	-0.86448624	8.78592656	1.8363E-14	6.4509E-13
ENSMUSG00000001774	*CHORDC1*	0.64331065	5.48016313	1.9164E-14	6.716E-13
ENSMUSG00000032267	*USP28*	-0.63475864	3.60069202	2.0816E-14	7.2777E-13
ENSMUSG00000024137	*E4F1*	-0.70634896	3.70140155	2.0927E-14	7.2901E-13
ENSMUSG00000020123	*AVPR1A*	-1.70796561	2.59104756	2.1078E-14	7.3169E-13
ENSMUSG00000035041	*CREB3L3*	-0.77157735	7.11765604	2.1266E-14	7.3649E-13
ENSMUSG00000001280	*SP1*	-0.43972672	5.813933	2.1522E-14	7.4359E-13
ENSMUSG00000071350	*SETDB2*	-1.60911483	5.1572288	2.2222E-14	7.6418E-13
ENSMUSG00000035828	*PIM3*	-0.78431135	5.59018158	2.2913E-14	7.8612E-13
ENSMUSG00000031796	*CFAP20*	-0.56078646	4.00739021	2.39E-14	8.1615E-13
ENSMUSG00000020783	*NCBP3*	-0.40218767	4.64626141	2.4059E-14	8.1969E-13
ENSMUSG00000021981	*CAB39L*	-0.65828102	6.02035145	2.4561E-14	8.3364E-13
ENSMUSG00000021025	*NFKBIA*	0.91024611	5.09776929	2.5411E-14	8.5975E-13
ENSMUSG00000022721	*TRMT2A*	0.42637865	5.35125176	2.5495E-14	8.6062E-13
ENSMUSG00000000538	*TOM1L2*	-0.58287566	5.25555565	2.6177E-14	8.7959E-13
ENSMUSG00000003464	*PEX19*	-0.42732437	5.81285398	2.6781E-14	8.9784E-13
ENSMUSG00000035696	*RNF38*	-0.52814093	4.02229903	2.7484E-14	9.193E-13
ENSMUSG00000018666	*CBX1*	0.38398601	5.46687127	2.7992E-14	9.3416E-13
ENSMUSG00000050373	*SNX21*	-0.77223459	3.96354064	2.8257E-14	9.4087E-13
ENSMUSG00000022965	*IFNGR2*	0.46524634	6.32226428	2.8408E-14	9.4377E-13
ENSMUSG00000039713	*PLEKHG5*	-0.83219206	3.69645862	2.8476E-14	9.4389E-13

ENSMUSG00000040270	*BACH2*	-0.94989841	2.69485633	2.8742E-14	9.4987E-13
ENSMUSG00000038914	*DIDO1*	0.42703077	5.53694802	2.8786E-14	9.4987E-13
ENSMUSG00000024975	*PDCD4*	-0.74432606	4.77364849	3.046E-14	9.9953E-13
ENSMUSG00000074212	*DNAJB14*	0.38168463	5.01293642	3.0418E-14	9.9953E-13
ENSMUSG00000071379	*HPCAL1*	-0.58123853	3.30583034	3.1393E-14	1.0267E-12
ENSMUSG00000041598	*CDC42EP4*	-0.61858339	5.85564092	3.2054E-14	1.046E-12
ENSMUSG00000048503	*TLCD5*	-1.23554772	2.22971477	3.2358E-14	1.0535E-12
ENSMUSG00000027247	*ARHGAP1*	0.51310218	5.00349138	3.3233E-14	1.0796E-12
ENSMUSG00000020083	*FAM241B*	-1.04486098	2.18996375	3.372E-14	1.0911E-12
ENSMUSG00000023266	*FRS3*	-1.17031009	1.24029115	3.4115E-14	1.0961E-12
ENSMUSG00000040859	*BSDC1*	-0.46233427	5.87257539	3.4106E-14	1.0961E-12
ENSMUSG00000021701	*PLK2*	0.90142978	6.20950045	3.4048E-14	1.0961E-12
ENSMUSG00000042680	*GAREM1*	-0.58597841	4.82763193	3.4482E-14	1.1031E-12
ENSMUSG00000078671	*CHD2*	0.55863921	6.3844498	3.4796E-14	1.1108E-12
ENSMUSG00000040557	*METTL27*	-0.60686075	4.62934631	3.5507E-14	1.131E-12
ENSMUSG00000047649	*POLR1G*	0.62947032	5.34640464	3.7189E-14	1.182E-12
ENSMUSG00000056209	*NPM3*	0.413551	5.18214812	3.7291E-14	1.1827E-12
ENSMUSG00000038279	*NOP2*	0.41607401	6.77510432	3.8293E-14	1.2118E-12
ENSMUSG00000030231	*PLEKHA5*	-0.41954679	5.74952627	3.8755E-14	1.2238E-12
ENSMUSG00000041798	*GCK*	-0.83329094	4.47040335	3.9366E-14	1.2405E-12
ENSMUSG00000059866	*TNIP2*	-0.62536146	3.2327398	3.9676E-14	1.2445E-12
ENSMUSG00000059714	*FLOT1*	-0.41061149	6.41570475	3.9666E-14	1.2445E-12
ENSMUSG00000038214	*BEND3*	0.62442723	4.1115718	3.9918E-14	1.2471E-12
ENSMUSG00000041417	*PIK3R1*	0.88629641	5.46064489	4.0189E-14	1.2529E-12
ENSMUSG00000095990	*ZFP97*	-0.83693417	2.24754688	4.1632E-14	1.2952E-12
ENSMUSG00000031833	*MAST3*	-0.7804245	4.40930939	4.2028E-14	1.3028E-12
ENSMUSG00000026821	*RALGDS*	-1.16343479	2.00948569	4.2608E-14	1.3172E-12
ENSMUSG00000039270	*MEGF9*	-0.63735138	4.56344503	4.3738E-14	1.3464E-12
ENSMUSG00000026349	*CCNT2*	-0.54363338	4.74852712	4.4458E-14	1.3657E-12
ENSMUSG00000092203	*1110038B12RIK*	-0.60759328	5.51327448	4.5223E-14	1.3834E-12

ENSMUSG00000027284	*CDAN1*	-0.59828342	3.80693577	4.5862E-14	1.4001E-12
ENSMUSG00000024150	*MCFD2*	0.48334839	6.90437521	4.6378E-14	1.41E-12
ENSMUSG00000029817	*TRA2A*	0.57679464	4.43013139	4.7582E-14	1.4436E-12
ENSMUSG00000027533	*FABP5*	-0.70985387	4.271465	4.8731E-14	1.4747E-12
ENSMUSG00000038059	*SMIM3*	1.24957685	3.50434287	4.8809E-14	1.4747E-12
ENSMUSG00000026694	*METTL13*	0.43140399	5.14334124	5.001E-14	1.5079E-12
ENSMUSG00000033444	*SPECC1L*	-0.41182623	5.70439437	5.0875E-14	1.5296E-12
ENSMUSG00000032902	*SLC16A1*	0.38033244	6.5505043	5.0937E-14	1.5296E-12
ENSMUSG00000013646	*SH3BP5L*	0.48490319	5.2424797	5.2757E-14	1.581E-12
ENSMUSG00000020590	*SNX13*	-0.38823945	5.18164539	5.5617E-14	1.6633E-12
ENSMUSG00000071078	*NR2C2AP*	0.51218638	4.99151522	5.5801E-14	1.6654E-12
ENSMUSG00000027130	*SLC12A6*	-0.63171384	3.95958376	5.601E-14	1.6683E-12
ENSMUSG00000047230	*CLDN2*	-1.11662277	3.77865413	5.713E-14	1.6982E-12
ENSMUSG00000030315	*VGLL4*	0.57205285	4.61322128	5.8922E-14	1.7444E-12
ENSMUSG00000026019	*WDR12*	0.49271783	5.2586076	6.0539E-14	1.7886E-12
ENSMUSG00000020706	*FTSJ3*	0.42107649	7.27189829	6.517E-14	1.9216E-12
ENSMUSG00000059439	*BCAS3*	-0.5838082	4.22540712	6.5648E-14	1.9318E-12
ENSMUSG00000022453	*NAGA*	-0.44262377	4.90041021	6.7306E-14	1.9755E-12
ENSMUSG00000028849	*MAP7D1*	0.44231296	7.78042227	6.93E-14	2.0231E-12
ENSMUSG00000026977	*MARCHF7*	-0.46504408	5.39453678	6.9462E-14	2.0238E-12
ENSMUSG00000037851	*IARS*	0.39469737	7.87732716	6.9929E-14	2.0333E-12
ENSMUSG00000040010	*SLC7A5*	1.13373648	5.56984992	7.2129E-14	2.0932E-12
ENSMUSG00000052512	*NAV2*	-0.8877286	4.25738268	7.2606E-14	2.0987E-12
ENSMUSG00000051627	*H1F4*	2.68347912	0.44515196	7.2992E-14	2.1057E-12
ENSMUSG00000034610	*TUT4*	-0.532323	4.97366744	7.6611E-14	2.2058E-12
ENSMUSG00000092274	*NEAT1*	-1.16013401	8.48160976	7.936E-14	2.2672E-12
ENSMUSG00000060862	*ZBTB40*	0.73447501	4.38006825	8.119E-14	2.3149E-12
ENSMUSG00000037364	*SRRT*	0.44410383	6.37231818	8.3658E-14	2.3761E-12
ENSMUSG00000048249	*CREBRF*	-0.96031911	4.37809973	8.836E-14	2.5E-12
ENSMUSG00000009569	*MRTFB*	-0.52086789	6.01469869	9.1647E-14	2.588E-12

ENSMUSG00000066687	*ZBTB16*	-0.82233031	6.32631923	1.0028E-13	2.8154E-12
ENSMUSG00000035969	*RUSC2*	-0.65242673	6.96465365	1.012E-13	2.8359E-12
ENSMUSG00000047875	*GPR157*	-0.92306829	2.05542349	1.0586E-13	2.9571E-12
ENSMUSG00000062761	*ZFP512*	-0.40953778	4.70045465	1.0593E-13	2.9571E-12
ENSMUSG00000019082	*SLC25A22*	-0.4323877	6.2257622	1.0976E-13	3.0582E-12
ENSMUSG00000056749	*NFIL3*	1.06919455	6.28274537	1.1522E-13	3.1922E-12
ENSMUSG00000037112	*SIK2*	-0.51483472	4.64069337	1.1546E-13	3.193E-12
ENSMUSG00000031790	*MMP15*	-0.72473133	5.06303933	1.1675E-13	3.2226E-12
ENSMUSG00000042213	*ZFAND4*	-1.23205428	2.02063842	1.1751E-13	3.2375E-12
ENSMUSG00000022774	*NCBP2*	0.51789995	4.11844404	1.1851E-13	3.2589E-12
ENSMUSG00000028978	*NOS3*	1.83372136	1.13224444	1.1909E-13	3.2687E-12
ENSMUSG00000014599	*CSF1*	1.18994463	5.54328118	1.2483E-13	3.4199E-12
ENSMUSG00000020898	*CTC1*	-0.69079324	3.82672047	1.2567E-13	3.4363E-12
ENSMUSG00000053950	*ADNP2*	-0.47702642	4.79049983	1.2831E-13	3.5022E-12
ENSMUSG00000033272	*SLC35A4*	0.5572167	4.87405028	1.3115E-13	3.5729E-12
ENSMUSG00000026827	*GPD2*	-0.53533799	5.66810015	1.3778E-13	3.7327E-12
ENSMUSG00000034586	*HID1*	0.88747277	3.17073454	1.4512E-13	3.9243E-12
ENSMUSG00000075590	*NRBP2*	-0.45603461	6.63503638	1.5576E-13	4.2044E-12
ENSMUSG00000027999	*PLA2G12A*	0.44007634	4.67163142	1.6461E-13	4.4271E-12
ENSMUSG00000046179	*E2F8*	-2.06577366	0.26078288	1.7149E-13	4.5953E-12
ENSMUSG00000022858	*TRA2B*	0.48305305	5.91252708	1.7199E-13	4.6002E-12
ENSMUSG00000020122	*EGFR*	-0.47739382	8.1733786	1.7447E-13	4.6495E-12
ENSMUSG00000021109	*HIF1A*	-0.5280477	6.28434222	1.8568E-13	4.9393E-12
ENSMUSG00000039031	*ARHGAP18*	-0.81517183	3.37818378	1.8803E-13	4.9747E-12
ENSMUSG00000021468	*SPTLC1*	0.43451515	5.09790307	1.8803E-13	4.9747E-12
ENSMUSG00000018765	*FXR2*	-0.41266396	6.64511801	1.8919E-13	4.9966E-12
ENSMUSG00000021257	*ANGEL1*	-0.40675104	4.86387539	1.9871E-13	5.2291E-12
ENSMUSG00000024231	*CUL2*	0.45817738	5.83836765	2.0126E-13	5.2869E-12
ENSMUSG00000030556	*LRRC28*	-0.45641171	4.74848358	2.0221E-13	5.3021E-12
ENSMUSG00000000325		-0.7745738	3.82582945	2.0352E-13	5.3185E-12

ENSMUSG00000021794	*GLUD1*	-0.41337121	8.66650523	2.0356E-13	5.3185E-12
ENSMUSG00000018999	*SLC35B4*	0.44804516	4.90191262	2.0525E-13	5.3533E-12
ENSMUSG00000044252	*OSBPL1A*	-0.58944192	6.06809312	2.0608E-13	5.3655E-12
ENSMUSG00000015890	*AMDHD1*	-0.76143645	3.57214646	2.1717E-13	5.6242E-12
ENSMUSG00000000791	*IL12RB1*	-1.09893564	1.88072082	2.2137E-13	5.7229E-12
ENSMUSG00000030062	*RPN1*	0.38109827	7.51525414	2.2317E-13	5.7592E-12
ENSMUSG00000031090	*NADSYN1*	-0.48961689	4.13388871	2.2422E-13	5.7761E-12
ENSMUSG00000060477	*IRAK2*	-0.41518092	5.71250635	2.3476E-13	6.0266E-12
ENSMUSG00000002227	*MOV10*	-0.79639256	4.65373445	2.3554E-13	6.0361E-12
ENSMUSG00000004100	*PPAN*	0.41012868	6.01758861	2.3949E-13	6.1266E-12
ENSMUSG00000045817	*ZFP36L2*	-0.72386424	6.36687682	2.4763E-13	6.3185E-12
ENSMUSG00000002343	*ARMC6*	0.70966916	4.52671798	2.4785E-13	6.3185E-12
ENSMUSG00000020743	*MIF4GD*	0.45676153	5.14086124	2.5145E-13	6.3991E-12
ENSMUSG00000015597	*ZFP318*	-0.48442494	5.07267306	2.519E-13	6.3995E-12
ENSMUSG00000004085	*MAP3K20*	-0.58312579	5.23111521	2.5881E-13	6.5636E-12
ENSMUSG00000020074	*CCAR1*	0.64869298	5.55380114	2.6015E-13	6.5864E-12
ENSMUSG00000028970	*ABCB1B*	0.91688968	4.49661689	2.6401E-13	6.6726E-12
ENSMUSG00000038773	*KDM3B*	-0.3836224	5.37768655	2.6492E-13	6.6839E-12
ENSMUSG00000030091	*NUP210*	0.39742535	5.97987502	2.6929E-13	6.771E-12
ENSMUSG00000030159	*CLEC1B*	-1.59528802	0.52492334	2.7135E-13	6.7964E-12
ENSMUSG00000032735	*ABLIM3*	-0.84390437	3.5305929	2.7169E-13	6.7964E-12
ENSMUSG00000044337	*ACKR3*	2.69169335	1.43617334	2.759E-13	6.8782E-12
ENSMUSG00000023960	*ENPP5*	-0.50774561	3.87129718	2.9792E-13	7.3897E-12
ENSMUSG00000062753	*SMIM29*	-0.68718673	3.80205132	3.2544E-13	8.0451E-12
ENSMUSG00000018547	*PIP4K2B*	0.48176121	5.43641874	3.3102E-13	8.1693E-12
ENSMUSG00000031917	*NIP7*	0.41202794	5.33407044	3.3554E-13	8.2669E-12
ENSMUSG00000031529	*TNKS*	-0.40179894	5.39098244	3.6133E-13	8.8725E-12
ENSMUSG00000027122	*ARL14EP*	0.50843622	4.73201536	3.785E-13	9.2786E-12
ENSMUSG00000001305	*RRP15*	0.44668203	5.33441574	3.8649E-13	9.4431E-12
ENSMUSG00000052566	*HOOK2*	-0.40709132	5.16961658	3.8839E-13	9.4737E-12

ENSMUSG00000055435	*MAF*	-1.1140402	2.22843746	3.9776E-13	9.6541E-12
ENSMUSG00000020962	*GTF2A1*	-0.4088452	4.74551035	3.9852E-13	9.6565E-12
ENSMUSG00000033773	*RPAP2*	-0.5164394	3.68528618	4.0312E-13	9.7519E-12
ENSMUSG00000075585		-0.73588262	2.72693273	4.0409E-13	9.7593E-12
ENSMUSG00000020248	*NFYB*	-0.53481855	4.76760519	4.2141E-13	1.0131E-11
ENSMUSG00000028525	*PDE4B*	0.71282094	3.26253869	4.2153E-13	1.0131E-11
ENSMUSG00000025017	*PIK3AP1*	0.7874606	7.04333345	4.2141E-13	1.0131E-11
ENSMUSG00000067199	*FRAT1*	-1.44869298	1.3386745	4.2233E-13	1.0133E-11
ENSMUSG00000027612	*MMP24*	1.20360702	3.00086471	4.3932E-13	1.0524E-11
ENSMUSG00000037103	*DCAF15*	-0.55229555	3.27793309	4.4015E-13	1.0526E-11
ENSMUSG00000024254	*ABCG8*	-1.50377934	2.36846511	4.8959E-13	1.1671E-11
ENSMUSG00000024493	*LARS*	0.40044607	6.95255237	4.9928E-13	1.1882E-11
ENSMUSG00000047109	*CLDN14*	-0.73160787	3.14713417	5.0524E-13	1.2005E-11
ENSMUSG00000056394	*LIG1*	-0.53222586	4.07577654	5.0997E-13	1.2098E-11
ENSMUSG00000031955	*BCAR1*	0.89008691	6.74544547	5.1745E-13	1.2255E-11
ENSMUSG00000020546	*STXBP4*	0.8745876	2.34122782	5.3092E-13	1.2554E-11
ENSMUSG00000025264	*TSR2*	0.53727567	4.42565411	5.3986E-13	1.2724E-11
ENSMUSG00000061175	*FNIP2*	-0.40498836	5.57931026	5.5019E-13	1.2947E-11
ENSMUSG00000070871	*CCNYL1*	0.55189162	3.57785692	5.5651E-13	1.3075E-11
ENSMUSG00000032998	*FOXJ3*	-0.46195487	4.89045285	5.614E-13	1.3148E-11
ENSMUSG00000043065	*SPICE1*	-0.66213821	2.93493577	5.7447E-13	1.3411E-11
ENSMUSG00000070644	*ETNK2*	-0.43432441	4.65351126	5.7566E-13	1.3417E-11
ENSMUSG00000038594	*CEP85L*	-0.89268255	4.66731574	5.9229E-13	1.3783E-11
ENSMUSG00000038697	*TAF5L*	0.3791281	5.11025427	5.9525E-13	1.383E-11
ENSMUSG00000031976	*URB2*	0.45283236	5.3713169	6.1045E-13	1.4161E-11
ENSMUSG00000051518	*RPS19BP1*	0.41450789	4.5555741	6.3358E-13	1.4674E-11
ENSMUSG00000022377	*ASAP1*	-0.43718455	5.40097012	6.6764E-13	1.539E-11
ENSMUSG00000060716	*PLEKHH1*	-0.6611074	3.8883266	6.8701E-13	1.5788E-11
ENSMUSG00000042349	*IKBKE*	1.03798809	5.26933052	6.8842E-13	1.5795E-11
ENSMUSG00000051469	*ZFP24*	0.46121572	4.70266077	7.0703E-13	1.6171E-11

ENSMUSG00000040997	*ABHD4*	-0.40047313	6.98312918	7.1663E-13	1.6366E-11
ENSMUSG00000022040	*EPHX2*	-0.38511993	6.45546556	7.3091E-13	1.6643E-11
ENSMUSG00000042726	*TRAFD1*	-0.40895632	5.46533749	7.4271E-13	1.6856E-11
ENSMUSG00000072294	*KLF12*	-0.76953067	3.02642085	7.7345E-13	1.7527E-11
ENSMUSG00000020176	*GRB10*	0.90480796	4.17542588	7.887E-13	1.7845E-11
ENSMUSG00000042750	*BEX2*	-2.03480365	-0.30475965	8.02E-13	1.8118E-11
ENSMUSG00000087590	*EPB41L4AOS*	-0.58310143	3.92637471	8.2395E-13	1.8528E-11
ENSMUSG00000036473	*TBC1D24*	0.80076312	4.8600701	8.2297E-13	1.8528E-11
ENSMUSG00000026565	*POU2F1*	-0.56720508	3.64095309	8.5223E-13	1.9135E-11
ENSMUSG00000023055	*CALCOCO1*	-0.78931758	5.58530094	8.6178E-13	1.932E-11
ENSMUSG00000030909	*ANKS4B*	0.91618914	4.5158237	8.8613E-13	1.9835E-11
ENSMUSG00000041241	*MUL1*	0.39197293	6.10128129	8.9337E-13	1.9967E-11
ENSMUSG00000042302	*EHBP1*	-0.59599282	3.40752573	9.208E-13	2.0517E-11
ENSMUSG00000075701	*SELENOS*	0.47230573	5.95185068	9.1969E-13	2.0517E-11
ENSMUSG00000000126	*WNT9A*	-0.74993319	2.96471492	9.2955E-13	2.065E-11
ENSMUSG00000019518	*AP4M1*	-0.59733584	3.59073635	9.5571E-13	2.1155E-11
ENSMUSG00000018166	*ERBB3*	-1.18289879	6.98279285	1.0102E-12	2.2273E-11
ENSMUSG00000022358	*FBXO32*	-0.84867626	2.39795616	1.0353E-12	2.2786E-11
ENSMUSG00000024317	*RNF138*	-0.54406354	3.68672898	1.0403E-12	2.2833E-11
ENSMUSG00000069114	*ZBTB10*	-0.5575885	3.95653284	1.0418E-12	2.2833E-11
ENSMUSG00000044748	*DEFB1*	-1.50007091	4.50604955	1.0655E-12	2.3316E-11
ENSMUSG00000033624	*PDPR*	-0.56910892	3.75630704	1.1307E-12	2.4597E-11
ENSMUSG00000026113	*INPP4A*	-0.52685162	3.84571053	1.1392E-12	2.4745E-11
ENSMUSG00000030583	*SIPA1L3*	0.57719776	4.83399079	1.1888E-12	2.5746E-11
ENSMUSG00000031381	*PIGA*	-0.59794332	4.04223494	1.2141E-12	2.6256E-11
ENSMUSG00000034118	*TPST1*	0.43718506	4.76252678	1.2673E-12	2.7287E-11
ENSMUSG00000032598	*NCKIPSD*	-0.73436156	3.05855983	1.3097E-12	2.8157E-11
ENSMUSG00000020644	*ID2*	-0.83101432	3.44877393	1.3288E-12	2.8525E-11
ENSMUSG00000006850	*TMCO6*	-0.86849924	3.19295428	1.341E-12	2.8747E-11
ENSMUSG00000021133	*SUSD6*	-0.5178121	6.63520175	1.3971E-12	2.9818E-11

ENSMUSG00000010608	*RBM25*	0.445543	6.41082479	1.4398E-12	3.0686E-11
ENSMUSG00000029787	*AVL9*	-0.39402606	4.62911041	1.4448E-12	3.0747E-11
ENSMUSG00000030255	*SSPN*	-1.46639198	0.27245596	1.4637E-12	3.1104E-11
ENSMUSG00000031723	*TXNL4B*	0.50856705	4.15348973	1.506E-12	3.1957E-11
ENSMUSG00000039463	*SLC9A8*	0.38927115	6.15861582	1.5341E-12	3.2412E-11
ENSMUSG00000021597	*SLF1*	-0.82108614	1.69318523	1.5787E-12	3.3212E-11
ENSMUSG00000037526	*ATG14*	-0.56780092	4.3096413	1.6082E-12	3.3687E-11
ENSMUSG00000028820	*SFPQ*	0.6225012	7.45067378	1.7017E-12	3.5444E-11
ENSMUSG00000050310	*RICTOR*	-0.56799464	4.59365632	1.7181E-12	3.5734E-11
ENSMUSG00000073700	*KLHL21*	-0.53279449	6.48952504	1.7465E-12	3.6223E-11
ENSMUSG00000016921	*SRSF6*	0.44828711	6.72119799	1.8335E-12	3.7973E-11
ENSMUSG00000025993	*SLC40A1*	0.71141248	5.9596067	1.8966E-12	3.9224E-11
ENSMUSG00000010554	*METTL16*	0.43594119	5.22544507	1.9146E-12	3.9541E-11
ENSMUSG00000032599	*IP6K2*	-0.84445152	5.53864426	1.9901E-12	4.0928E-11
ENSMUSG00000026718	*STAM*	-0.38409439	5.30456915	1.9901E-12	4.0928E-11
ENSMUSG00000025971	*MAIP1*	0.39809287	4.57957972	2.0902E-12	4.2806E-11
ENSMUSG00000020077	*SRGN*	0.98871533	2.41435758	2.1446E-12	4.386E-11
ENSMUSG00000040022	*RAB11FIP2*	-0.52609674	3.36338246	2.1774E-12	4.4469E-11
ENSMUSG00000058392	*RRP1B*	0.5427295	4.98926323	2.2615E-12	4.6123E-11
ENSMUSG00000036894	*RAP2B*	0.92878528	4.92943739	2.2778E-12	4.639E-11
ENSMUSG00000021285	*PPP1R13B*	-0.5329852	4.57979923	2.3366E-12	4.7391E-11
ENSMUSG00000025245	*LZTFL1*	-0.47668266	3.84371728	2.3951E-12	4.8443E-11
ENSMUSG00000032382	*SNX1*	-0.42273465	5.82683545	2.4006E-12	4.8487E-11
ENSMUSG00000013275	*SLC41A1*	-0.54440802	4.35754531	2.429E-12	4.8927E-11
ENSMUSG00000072812		-0.78377404	5.41975623	2.5248E-12	5.0786E-11
ENSMUSG00000056515	*RAB31*	0.41955456	6.03157873	2.8574E-12	5.7397E-11
ENSMUSG00000059142	*ZFP945*	0.79930536	3.80684016	2.868E-12	5.7454E-11
ENSMUSG00000024654	*ASRGL1*	-0.66657607	4.68006891	2.8923E-12	5.7862E-11
ENSMUSG00000029321	*SLC10A6*	-1.52465835	2.1763959	3.0359E-12	6.057E-11
ENSMUSG00000019820	*UTRN*	-0.41731025	6.24456121	3.048E-12	6.0728E-11

ENSMUSG00000048285	*FRMD6*	0.90860755	4.56741241	3.2285E-12	6.415E-11
ENSMUSG00000068551	*ZFP467*	-0.96645278	2.111174	3.2898E-12	6.5191E-11
ENSMUSG00000030880	*POLR3E*	0.42966226	5.85785484	3.4238E-12	6.7664E-11
ENSMUSG00000117780		-1.28180394	1.2996458	3.5759E-12	7.0481E-11
ENSMUSG00000022637	*CBLB*	-0.49862305	5.65867108	3.6129E-12	7.1116E-11
ENSMUSG00000036155	*MGAT5*	-0.44853912	4.76781997	3.6859E-12	7.2455E-11
ENSMUSG00000033540	*IDUA*	-0.68607444	2.89998443	3.6951E-12	7.2539E-11
ENSMUSG00000038167	*PLEKHG6*	-0.82263667	3.04192623	3.959E-12	7.7101E-11
ENSMUSG00000018500	*ADORA2B*	-1.30620114	2.01270485	3.9977E-12	7.7751E-11
ENSMUSG00000044783	*A730008H23RIK*	-0.39381293	5.32117915	4.0918E-12	7.9058E-11
ENSMUSG00000044783	*HJURP*	-0.39381293	5.32117915	4.0918E-12	7.9058E-11
ENSMUSG00000031523	*DLC1*	0.59162431	4.73830981	4.2983E-12	8.2831E-11
ENSMUSG00000002222	*RMND5A*	-0.43993716	6.41137374	4.3828E-12	8.4347E-11
ENSMUSG00000042369	*RBM45*	0.41598347	5.14078379	4.4439E-12	8.5244E-11
ENSMUSG00000020346	*MGAT1*	0.40326377	6.7456689	4.5451E-12	8.6789E-11
ENSMUSG00000024843	*CHKA*	0.8321459	7.08324484	4.5448E-12	8.6789E-11
ENSMUSG00000059851	*KMT5C*	-0.68739929	3.42838716	4.8281E-12	9.1835E-11
ENSMUSG00000021136	*SMOC1*	-0.61784168	5.89842014	4.8482E-12	9.2096E-11
ENSMUSG00000031453	*RASA3*	-0.68733987	2.8434229	4.8695E-12	9.2383E-11
ENSMUSG00000029697	*FEZF1*	-2.42686596	-0.42926419	5.0427E-12	9.5421E-11
ENSMUSG00000039842	*MCPH1*	-0.5238015	3.55133199	5.1112E-12	9.6593E-11
ENSMUSG00000041272	*TOX*	-0.87790527	3.87933443	5.1204E-12	9.6643E-11
ENSMUSG00000033287	*KCTD17*	0.39330319	5.63859076	5.2421E-12	9.8685E-11
ENSMUSG00000052298	*CDC42SE2*	0.40124036	5.23516231	5.332E-12	1.0012E-10
ENSMUSG00000053730	*TMEM39B*	0.81009671	2.23325342	5.3281E-12	1.0012E-10
ENSMUSG00000032897	*NFYC*	0.41191757	4.79363173	5.4322E-12	1.0187E-10
ENSMUSG00000047022	*MIPOL1*	-0.72844759	3.04957381	5.5291E-12	1.0356E-10
ENSMUSG00000021750	*FAM107A*	-2.35311806	1.61494333	5.5502E-12	1.0382E-10
ENSMUSG00000117942		-0.54373243	3.87006057	5.5771E-12	1.0419E-10
ENSMUSG00000027940	*TPM3*	0.41045611	8.70075331	5.6956E-12	1.0613E-10

ENSMUSG00000033610	*PANK1*	-0.51843974	4.52333711	5.8912E-12	1.0964E-10
ENSMUSG00000020680	*TAF15*	0.41807118	6.75434734	5.9179E-12	1.1E-10
ENSMUSG00000037029	*ZFP146*	0.38998571	4.57690954	6.2595E-12	1.1605E-10
ENSMUSG00000007850	*HNRNPH1*	0.40699666	6.2252645	6.4285E-12	1.1889E-10
ENSMUSG00000031482	*SLC25A15*	0.6084699	5.81409637	6.5006E-12	1.2007E-10
ENSMUSG00000038256	*BCL9*	-0.74518732	2.65251922	6.5558E-12	1.2094E-10
ENSMUSG00000030256	*BHLHE41*	-1.09201306	2.18861419	6.5814E-12	1.2126E-10
ENSMUSG00000046733	*GPRC5A*	1.01933784	3.88651356	6.6376E-12	1.2214E-10
ENSMUSG00000038546	*RANBP9*	-0.38144828	5.92240452	6.6542E-12	1.2229E-10
ENSMUSG00000029192	*TBC1D14*	0.49320564	5.57019184	6.728E-12	1.2349E-10
ENSMUSG00000027429	*SEC23B*	0.42364834	5.95430521	7.046E-12	1.2901E-10
ENSMUSG00000019947	*ARID5B*	-0.67766028	5.69078759	7.069E-12	1.2927E-10
ENSMUSG00000042429	*ADORA1*	-1.1755024	3.10072919	7.4116E-12	1.3486E-10
ENSMUSG00000037475	*THOC2*	0.43948862	4.95618241	7.669E-12	1.3937E-10
ENSMUSG00000015882	*LCORL*	-0.61582738	3.38278704	7.7598E-12	1.4067E-10
ENSMUSG00000015882	*GM3414*	-0.61582738	3.38278704	7.7598E-12	1.4067E-10
ENSMUSG00000039782	*CPEB2*	-0.78036318	7.04852209	7.9743E-12	1.4438E-10
ENSMUSG00000040848	*SFT2D2*	0.37895535	6.56502761	8.0618E-12	1.4579E-10
ENSMUSG00000037369	*KDM6A*	-0.42098429	4.58308597	8.1551E-12	1.4711E-10
ENSMUSG00000068742	*CRY2*	-0.53824001	5.27741894	8.2357E-12	1.4838E-10
ENSMUSG00000021891	*METTL6*	0.49708571	4.53195031	8.4E-12	1.5097E-10
ENSMUSG00000040613	*APOBEC1*	0.46518096	4.73313406	8.4204E-12	1.5116E-10
ENSMUSG00000001248	*GRAMD1A*	-0.49489093	3.58752339	8.4682E-12	1.5183E-10
ENSMUSG00000079037	*PRNP*	0.43402137	4.73025672	8.5131E-12	1.5245E-10
ENSMUSG00000022887	*MASP1*	-0.44458756	6.74912187	8.8404E-12	1.5754E-10
ENSMUSG00000028328	*TMOD1*	-1.9588716	-0.72594955	8.9754E-12	1.5956E-10
ENSMUSG00000002996	*HBP1*	-0.68208256	6.69686057	9.2362E-12	1.638E-10
ENSMUSG00000032715	*TRIB3*	1.20400084	7.25688367	9.3074E-12	1.6486E-10
ENSMUSG00000089824	*RBM12*	0.74796732	4.11624074	9.408E-12	1.6644E-10
ENSMUSG00000044854	*1700056E22RIK*	-0.9881911	1.60778557	9.5128E-12	1.6809E-10

ENSMUSG00000021180	*RPS6KA5*	-1.3708454	-0.0192151	9.7647E-12	1.7192E-10
ENSMUSG00000034858	*ATOSA*	-0.88347152	4.68700718	9.9276E-12	1.7458E-10
ENSMUSG00000002249	*TEAD3*	-0.7349195	3.02827193	9.9935E-12	1.7532E-10
ENSMUSG00000029246	*PPAT*	0.38550877	5.26769364	9.9909E-12	1.7532E-10
ENSMUSG00000020258	*GLYCTK*	0.8717781	6.46726773	1.0085E-11	1.7661E-10
ENSMUSG00000004044	*CAVIN1*	1.08502537	3.65880739	1.0386E-11	1.8156E-10
ENSMUSG00000053801	*GRWD1*	0.4777332	5.65141932	1.0561E-11	1.844E-10
ENSMUSG00000024045	*AKAP8*	0.38072276	5.85731922	1.1053E-11	1.9231E-10
ENSMUSG00000029273	*SULT1D1*	-1.18283743	5.41051062	1.1818E-11	2.0465E-10
ENSMUSG00000037370	*ENPP1*	-0.65193323	4.77817677	1.1808E-11	2.0465E-10
ENSMUSG00000031565	*FGFR1*	-0.66019409	4.51369943	1.2159E-11	2.1005E-10
ENSMUSG00000035385	*CCL2*	1.14499828	6.86821851	1.2713E-11	2.1886E-10
ENSMUSG00000042557	*SIN3A*	-0.39859702	4.86598461	1.31E-11	2.2473E-10
ENSMUSG00000032369	*PLSCR1*	0.81259455	7.60537085	1.3099E-11	2.2473E-10
ENSMUSG00000022096	*HR*	-1.42798622	0.90686938	1.332E-11	2.2822E-10
ENSMUSG00000029185	*FAM114A1*	-0.53518652	4.57615417	1.3639E-11	2.3261E-10
ENSMUSG00000040274	*CDK6*	0.47307803	6.85781981	1.3663E-11	2.3275E-10
ENSMUSG00000030979	*UROS*	0.48480259	3.71198166	1.416E-11	2.4011E-10
ENSMUSG00000019832	*RAB32*	0.59814956	4.27392909	1.4671E-11	2.479E-10
ENSMUSG00000071856	*MCC*	-1.14886285	2.86842552	1.476E-11	2.4913E-10
ENSMUSG00000020889	*NR1D1*	-1.06478632	4.96346634	1.4983E-11	2.5213E-10
ENSMUSG00000060981	*H4C8*	1.47385156	1.44569583	1.5063E-11	2.5307E-10
ENSMUSG00000098234	*SNHG6*	-0.38681383	5.16330033	1.6141E-11	2.7056E-10
ENSMUSG00000028293	*SLC35A1*	0.41380132	4.33118477	1.6765E-11	2.8071E-10
ENSMUSG00000048109	*RBM15*	0.41670986	4.82926487	1.7292E-11	2.8919E-10
ENSMUSG00000029165	*AGBL5*	-0.69215966	3.06154721	1.752E-11	2.9234E-10
ENSMUSG00000037946	*FGD3*	1.27258864	4.26845992	1.7841E-11	2.9703E-10
ENSMUSG00000031880	*RRAD*	1.57301868	0.81012959	1.7862E-11	2.9703E-10
ENSMUSG00000050628	*UBALD2*	-0.81559769	5.4953436	1.7932E-11	2.9787E-10
ENSMUSG00000048537	*PHLDB1*	0.51997296	5.73608755	1.8101E-11	3.0033E-10

ENSMUSG00000024899	*PAPSS2*	-0.57248058	6.43417554	1.925E-11	3.1761E-10
ENSMUSG00000019866	*CRYBG1*	0.38741136	5.83361963	1.9508E-11	3.215E-10
ENSMUSG00000043998	*MGAT2*	0.4043328	6.00972059	1.9864E-11	3.2663E-10
ENSMUSG00000020994	*PNN*	0.5309853	5.33622994	2.0095E-11	3.3006E-10
ENSMUSG00000018841	*RAD51D*	-0.43220256	4.21507944	2.0959E-11	3.4388E-10
ENSMUSG00000030083	*ABTB1*	-0.5606111	4.46455162	2.1369E-11	3.4981E-10
ENSMUSG00000028609	*MAGOH*	0.40956556	4.39573878	2.1496E-11	3.515E-10
ENSMUSG00000025421	*HDHD2*	0.38518687	4.56792021	2.2323E-11	3.6381E-10
ENSMUSG00000017418	*ARL5B*	-0.4812087	5.02474398	2.2498E-11	3.6626E-10
ENSMUSG00000021238	*ALDH6A1*	-0.46261469	5.83971149	2.2951E-11	3.728E-10
ENSMUSG00000041977	*ARHGEF11*	0.37952872	6.37439854	2.3745E-11	3.8485E-10
ENSMUSG00000020107	*ANAPC16*	0.4102357	5.64555666	2.3832E-11	3.8545E-10
ENSMUSG00000026979	*PSD4*	0.61262371	4.22211315	2.3835E-11	3.8545E-10
ENSMUSG00000093769	*H3C14*	4.71222042	-0.6211725	2.3895E-11	3.86E-10
ENSMUSG00000020593	*LPIN1*	-1.3005189	5.76572672	2.3993E-11	3.8674E-10
ENSMUSG00000028713	*CYP4B1*	-0.7438251	3.0575979	2.4122E-11	3.8838E-10
ENSMUSG00000028680	*PLK3*	-0.6740161	6.85834763	2.4345E-11	3.9155E-10
ENSMUSG00000032440	*TGFBR2*	0.42775217	5.92403817	2.4753E-11	3.9724E-10
ENSMUSG00000029385	*CCNG2*	-0.99854305	5.04610086	2.557E-11	4.0913E-10
ENSMUSG00000058900	*RSL1*	1.08892452	3.41514744	2.5578E-11	4.0913E-10
ENSMUSG00000064220	*H2AC18*	0.86524826	4.00706031	2.5863E-11	4.1325E-10
ENSMUSG00000038859	*BAIAP2L1*	-0.39140243	6.39276334	2.5994E-11	4.1488E-10
ENSMUSG00000020642	*RNF144A*	-1.17503859	1.72611147	2.6921E-11	4.2921E-10
ENSMUSG00000079316	*RAB9*	-0.42750117	4.91912115	2.7028E-11	4.3046E-10
ENSMUSG00000057133	*CHD6*	-0.61738144	3.0995002	2.7932E-11	4.4436E-10
ENSMUSG00000021007	*SPATA7*	-0.71440587	1.77742738	2.8894E-11	4.5917E-10
ENSMUSG00000015942	*GTF2IRD2*	0.62994349	3.00239423	2.917E-11	4.6256E-10
ENSMUSG00000006307	*KMT2B*	-0.37876273	5.87383868	3.0229E-11	4.7781E-10
ENSMUSG00000048756	*FOXO3*	-0.61918306	5.88912314	3.0465E-11	4.8102E-10
ENSMUSG00000016933	*PLCG1*	-0.41223504	5.03026556	3.0555E-11	4.8192E-10

ENSMUSG00000017929	*B4GALT5*	0.45044093	4.84874935	3.284E-11	5.1631E-10
ENSMUSG00000024944	*ARL2*	-0.53842067	2.56262675	3.2969E-11	5.1777E-10
ENSMUSG00000022010	*TSC22D1*	1.00949792	5.31385051	3.3189E-11	5.2067E-10
ENSMUSG00000078427	*SARNP*	0.39733734	4.48763858	3.3493E-11	5.2488E-10
ENSMUSG00000023883	*PHF10*	0.45199148	5.92335171	3.4109E-11	5.3339E-10
ENSMUSG00000068134	*ZFP120*	-0.54962377	2.86532448	3.4213E-11	5.3445E-10
ENSMUSG00000038618	*RASSF7*	-0.64444008	3.28339069	3.4565E-11	5.388E-10
ENSMUSG00000031617	*TMEM184C*	0.45100288	4.55360103	3.5622E-11	5.5351E-10
ENSMUSG00000026737	*PIP4K2A*	-0.63786473	3.22818048	3.6293E-11	5.6275E-10
ENSMUSG00000038764	*PTPN3*	-0.39095685	5.16615078	3.7895E-11	5.8697E-10
ENSMUSG00000041779	*TRAM2*	-0.41230763	4.33042163	3.8325E-11	5.9238E-10
ENSMUSG00000032035	*ETS1*	0.96625909	5.27804412	3.9014E-11	6.0176E-10
ENSMUSG00000029551	*PSMG3*	0.49847347	4.26060279	3.9159E-11	6.0337E-10
ENSMUSG00000031781	*CIAPIN1*	0.4106618	5.17479206	3.9512E-11	6.0817E-10
ENSMUSG00000025347	*METTL7B*	0.53771519	6.97290359	3.9864E-11	6.1294E-10
ENSMUSG00000029073	*CPTP*	0.43383623	4.3978837	3.991E-11	6.1301E-10
ENSMUSG00000031714	*GAB1*	-0.39940866	4.91997805	4.0416E-11	6.1961E-10
ENSMUSG00000028163	*NFKB1*	0.38048533	5.79165422	4.0424E-11	6.1961E-10
ENSMUSG00000025408	*DDIT3*	1.5914887	5.29303887	4.3383E-11	6.6289E-10
ENSMUSG00000067889	*SPTBN2*	0.6791562	5.6300765	4.3727E-11	6.6676E-10
ENSMUSG00000003435	*SUPT5*	0.37904233	8.06819702	4.6732E-11	7.0744E-10
ENSMUSG00000057342	*SPHK2*	0.51999967	5.64841962	4.7747E-11	7.2058E-10
ENSMUSG00000028633	*CTPS*	0.46115205	5.98833989	4.8285E-11	7.2719E-10
ENSMUSG00000027016	*ZFP385B*	-0.70364671	3.66200485	4.9994E-11	7.5217E-10
ENSMUSG00000028862	*MAP3K6*	-0.72584498	4.39855354	5.2583E-11	7.887E-10
ENSMUSG00000028957	*PER3*	-1.16421648	1.38889904	5.313E-11	7.9609E-10
ENSMUSG00000034591	*SLC41A2*	0.38984364	5.76268984	5.3324E-11	7.9817E-10
ENSMUSG00000020873	*SLC35B1*	0.41812512	5.3768869	5.3915E-11	8.0506E-10
ENSMUSG00000031365	*ZFP275*	0.38532197	4.40273409	5.4783E-11	8.1668E-10
ENSMUSG00000023809	*RPS6KA2*	-1.14176183	1.14976102	5.5787E-11	8.2996E-10

ENSMUSG00000047604	*FRAT2*	-1.2395967	1.09981425	5.6018E-11	8.3255E-10
ENSMUSG00000036885	*ARHGEF26*	-0.50818777	4.49811482	5.8282E-11	8.6445E-10
ENSMUSG00000086841	*2410006H16RIK*	-0.40894725	5.98653737	5.9114E-11	8.7415E-10
ENSMUSG00000111375	*BTBD8*	-0.82287399	2.29989992	6.1855E-11	9.1192E-10
ENSMUSG00000016946	*KCTD5*	0.43683765	4.26646782	6.3093E-11	9.2831E-10
ENSMUSG00000039244	*INTS15*	0.39832099	5.79053361	6.4271E-11	9.4093E-10
ENSMUSG00000039384	*DUSP10*	-0.50503502	6.42531502	6.4408E-11	9.42E-10
ENSMUSG00000021009	*PTPN21*	0.3985705	6.86543404	6.467E-11	9.4395E-10
ENSMUSG00000029535	*TRIAP1*	0.4884708	4.21652573	6.5233E-11	9.5045E-10
ENSMUSG00000036873	*2410004B18RIK*	0.57161116	3.25483067	6.6039E-11	9.6107E-10
ENSMUSG00000037111	*SETD7*	-0.60219768	5.41539894	6.919E-11	1.0052E-09
ENSMUSG00000022766	*SERPIND1*	0.55770129	4.61754415	7.1284E-11	1.0343E-09
ENSMUSG00000039686	*ZER1*	-0.42732485	4.4328833	7.1449E-11	1.0357E-09
ENSMUSG00000074876		-0.50212856	3.84043669	7.233E-11	1.0474E-09
ENSMUSG00000068876	*CGN*	0.62320726	7.06916073	7.2432E-11	1.0479E-09
ENSMUSG00000068663	*CLEC16A*	0.39023242	4.92034161	7.2739E-11	1.0513E-09
ENSMUSG00000021903	*GALNT15*	-1.67549716	0.8764806	7.5185E-11	1.0856E-09
ENSMUSG00000040549	*CKAP5*	-0.41720994	4.63069116	7.6988E-11	1.1083E-09
ENSMUSG00000023150	*IVNS1ABP*	-0.42620687	6.74741519	7.7728E-11	1.1179E-09
ENSMUSG00000029009	*MTHFR*	0.39885604	5.51443809	8.0395E-11	1.1551E-09
ENSMUSG00000030498	*GAS2*	-0.58389763	4.52542787	8.2555E-11	1.1827E-09
ENSMUSG00000022325	*POP1*	0.67250359	3.39975966	8.3864E-11	1.1991E-09
ENSMUSG00000046861	*HECTD3*	-0.40968828	5.06710814	8.513E-11	1.2148E-09
ENSMUSG00000026692	*FMO4*	-0.80285141	2.15813809	8.5906E-11	1.2247E-09
ENSMUSG00000036880	*ACAA2*	-0.39235891	7.26365622	8.7261E-11	1.2404E-09
ENSMUSG00000026393	*NEK7*	-0.44610439	6.77564629	8.8587E-11	1.258E-09
ENSMUSG00000118168		-1.91799189	-0.25998569	9.0764E-11	1.2848E-09
ENSMUSG00000022594	*LYNX1*	-0.80951049	2.12692114	9.1589E-11	1.2919E-09
ENSMUSG00000026107	*NABP1*	0.44698356	6.95319389	9.1537E-11	1.2919E-09
ENSMUSG00000035356	*NFKBIZ*	-0.97824695	2.34630584	9.2048E-11	1.2972E-09

ENSMUSG00000030726	*POLD3*	-0.39608305	4.76926694	9.472E-11	1.331E-09
ENSMUSG00000049686	*ORAI1*	0.39683525	5.19001847	9.4994E-11	1.3335E-09
ENSMUSG00000049800	*SERTAD2*	0.38024957	5.01756736	9.6891E-11	1.3567E-09
ENSMUSG00000029217	*TEC*	-0.48326203	4.55814115	9.7112E-11	1.3581E-09
ENSMUSG00000020843	*TIMM22*	0.48265574	4.01463296	9.954E-11	1.3868E-09
ENSMUSG00000000085	*SCMH1*	-0.40365582	3.87960589	1.0004E-10	1.3924E-09
ENSMUSG00000043415	*OTUD1*	0.47158496	5.66906147	1.0422E-10	1.4478E-09
ENSMUSG00000029752	*ASNS*	0.44223684	6.37511621	1.0672E-10	1.4728E-09
ENSMUSG00000020017	*HAL*	-1.34841537	3.56178626	1.1036E-10	1.5216E-09
ENSMUSG00000019577	*PDK4*	-0.95694797	7.2993348	1.1167E-10	1.537E-09
ENSMUSG00000024258	*POLR2D*	0.56353594	3.23078325	1.1422E-10	1.5688E-09
ENSMUSG00000041132	*N4BP2L1*	-0.94806645	3.53898727	1.1525E-10	1.5801E-09
ENSMUSG00000054477	*KCNN2*	-1.04376839	2.05884991	1.1851E-10	1.6234E-09
ENSMUSG00000002897	*IL17RA*	0.47286478	6.83845183	1.1898E-10	1.6267E-09
ENSMUSG00000002910	*ARRDC2*	-1.07550066	1.58170114	1.1961E-10	1.6338E-09
ENSMUSG00000004562	*ARHGEF40*	-0.72588357	3.98959192	1.2347E-10	1.6803E-09
ENSMUSG00000071226	*CECR2*	-0.60983617	3.45337418	1.2347E-10	1.6803E-09
ENSMUSG00000024912	*FOSL1*	0.86611965	6.08552458	1.2336E-10	1.6803E-09
ENSMUSG00000059554	*CCDC28A*	-0.54797424	3.33513817	1.2754E-10	1.7308E-09
ENSMUSG00000031232	*MAGT1*	0.38284428	5.38238231	1.2769E-10	1.7313E-09
ENSMUSG00000033526	*PPIP5K1*	-0.57177501	3.12320593	1.2936E-10	1.7524E-09
ENSMUSG00000026784	*PDSS1*	0.63376082	2.81452399	1.3002E-10	1.7596E-09
ENSMUSG00000032068	*PLET1*	-1.02006405	4.14261569	1.3017E-10	1.76E-09
ENSMUSG00000004768	*RAB23*	0.50638838	3.538214	1.321E-10	1.7828E-09
ENSMUSG00000021712	*TRIM23*	-0.46399464	3.69858389	1.3386E-10	1.805E-09
ENSMUSG00000047888	*TNRC6B*	-0.47241113	4.85950835	1.3402E-10	1.8054E-09
ENSMUSG00000020034	*TCP11L2*	-1.17932527	3.22159524	1.3602E-10	1.8308E-09
ENSMUSG00000027327	*ADISSP*	-0.49491793	3.90439941	1.3856E-10	1.8615E-09
ENSMUSG00000026482	*RGL1*	-1.13460323	1.29914667	1.4766E-10	1.9765E-09
ENSMUSG00000025185	*LOXL4*	0.62660386	5.09457881	1.4982E-10	2.0018E-09

ENSMUSG00000041438	*UTP4*	0.40365702	6.2795374	1.5124E-10	2.0153E-09
ENSMUSG00000046982	*TSHZ1*	0.43304363	5.07585075	1.538E-10	2.0438E-09
ENSMUSG00000018733	*PEX12*	0.4067371	3.82644338	1.5519E-10	2.0604E-09
ENSMUSG00000055723	*RRAS2*	0.51110779	7.62848013	1.554E-10	2.0613E-09
ENSMUSG00000040282	*CDIN1*	-0.53678276	2.85702869	1.6157E-10	2.1393E-09
ENSMUSG00000002731	*PRKRA*	-0.59566811	2.84079458	1.6249E-10	2.1495E-09
ENSMUSG00000031843	*MPHOSPH6*	0.49332267	3.90297477	1.6485E-10	2.1788E-09
ENSMUSG00000073600	*PROB1*	-0.67141246	2.3147779	1.6508E-10	2.1799E-09
ENSMUSG00000054408	*SPCS3*	0.40683692	5.8549861	1.6694E-10	2.2005E-09
ENSMUSG00000031853	*MAP3K21*	-1.01265685	1.19717216	1.727E-10	2.2724E-09
ENSMUSG00000015776	*MED22*	0.46672535	4.92886747	1.7877E-10	2.3474E-09
ENSMUSG00000018541	*CWC25*	0.41708095	4.2812343	1.7951E-10	2.3535E-09
ENSMUSG00000028479	*GNE*	-0.53730809	6.15066781	1.841E-10	2.4051E-09
ENSMUSG00000041935	*RIMOC1*	-0.38704012	4.98712001	1.8564E-10	2.421E-09
ENSMUSG00000035049	*RRP12*	0.4075032	6.12280288	1.8778E-10	2.4444E-09
ENSMUSG00000027963	*EXTL2*	0.46339608	3.5145439	1.8838E-10	2.4501E-09
ENSMUSG00000010175	*PROX1*	-0.79146558	4.45414292	1.916E-10	2.4889E-09
ENSMUSG00000053091	*LINS1*	0.51761418	3.68083422	1.9285E-10	2.5006E-09
ENSMUSG00000022553	*MAF1*	-0.45090991	4.54751173	2.051E-10	2.6535E-09
ENSMUSG00000047767	*ATG16L2*	-0.46278457	3.95830081	2.1587E-10	2.7854E-09
ENSMUSG00000009293	*UBE2G2*	0.51956011	6.77090248	2.1769E-10	2.8064E-09
ENSMUSG00000036315	*POLR1H*	0.65518886	2.91734144	2.1813E-10	2.8087E-09
ENSMUSG00000025007	*ALDH18A1*	0.70820827	5.1108151	2.1825E-10	2.8087E-09
ENSMUSG00000035227	*SPCS2*	0.39196106	6.30441546	2.2165E-10	2.845E-09
ENSMUSG00000022893	*ADAMTS1*	-0.50710575	4.87718149	2.2242E-10	2.85E-09
ENSMUSG00000061028	*CLASRP*	-0.40464548	4.71981953	2.2342E-10	2.8577E-09
ENSMUSG00000071369	*MAP3K5*	-0.39881977	5.88679259	2.2596E-10	2.8877E-09
ENSMUSG00000041229	*PHF8*	-0.47671557	4.70406931	2.2626E-10	2.889E-09
ENSMUSG00000025008	*TCTN3*	-0.63507154	1.95439701	2.2938E-10	2.9212E-09
ENSMUSG00000045659	*PLEKHA7*	-0.58780765	3.96822657	2.3332E-10	2.9663E-09

ENSMUSG00000025225	*NFKB2*	0.51772257	6.89302075	2.3596E-10	2.9973E-09
ENSMUSG00000029312	*KLHL8*	-0.52304289	2.79909643	2.378E-10	3.018E-09
ENSMUSG00000028850	*GPATCH3*	0.41290562	4.31711212	2.3863E-10	3.0259E-09
ENSMUSG00000021240	*ABCD4*	-0.43173079	4.36951075	2.3989E-10	3.0393E-09
ENSMUSG00000039910	*CITED2*	-0.86304557	6.80598236	2.408E-10	3.0473E-09
ENSMUSG00000027163	*COMMD9*	0.49498371	4.19421196	2.4587E-10	3.1043E-09
ENSMUSG00000030208	*EMP1*	-1.05031042	2.54668812	2.5072E-10	3.1628E-09
ENSMUSG00000029287	*TGFBR3*	-0.95637693	1.3044661	2.5267E-10	3.1848E-09
ENSMUSG00000033107	*RNF125*	-0.73843287	5.67904386	2.5483E-10	3.2064E-09
ENSMUSG00000042675	*YPEL3*	-0.6411342	5.22035016	2.5869E-10	3.2457E-09
ENSMUSG00000072568	*LRATD2*	0.55838729	4.85231792	2.5884E-10	3.2457E-09
ENSMUSG00000032688	*MALT1*	-0.60633582	2.5298696	2.6476E-10	3.3087E-09
ENSMUSG00000030850	*ATE1*	-0.42258972	4.9510456	2.6563E-10	3.3168E-09
ENSMUSG00000028383	*HSDL2*	-0.39649215	5.38590958	2.7001E-10	3.3657E-09
ENSMUSG00000105703		-0.64888377	10.3051729	2.7529E-10	3.4286E-09
ENSMUSG00000074071	*FAM169B*	0.61631715	3.76535024	2.7579E-10	3.4319E-09
ENSMUSG00000029695	*AASS*	-0.67837011	5.77306667	2.8257E-10	3.5077E-09
ENSMUSG00000009575	*CBX5*	0.38145199	4.8792837	2.8776E-10	3.5688E-09
ENSMUSG00000015027	*GALNS*	-0.39961439	4.7403969	3.0756E-10	3.7888E-09
ENSMUSG00000038268	*OVCA2*	0.43840383	4.04393527	3.148E-10	3.8715E-09
ENSMUSG00000040123	*ZMYM5*	-0.38910371	4.6420422	3.2015E-10	3.934E-09
ENSMUSG00000028010	*GAR1*	0.39994847	5.27547331	3.2261E-10	3.9609E-09
ENSMUSG00000021431	*SNRNP48*	-0.4580119	4.10995037	3.4036E-10	4.1649E-09
ENSMUSG00000044350	*LACC1*	0.54709572	4.20664479	3.4033E-10	4.1649E-09
ENSMUSG00000053886	*SH2D4A*	0.86994823	3.354597	3.4451E-10	4.2087E-09
ENSMUSG00000009739	*POU6F1*	-0.63106501	2.61339162	3.4613E-10	4.2184E-09
ENSMUSG00000026981	*IL1RN*	0.80301303	4.5605786	3.4617E-10	4.2184E-09
ENSMUSG00000042608	*STK40*	0.68369958	6.25559262	3.529E-10	4.295E-09
ENSMUSG00000028469	*NPR2*	-0.45527233	4.02620374	3.5809E-10	4.3529E-09
ENSMUSG00000041836	*PTPRE*	-0.73686355	2.67922115	3.5909E-10	4.3613E-09

ENSMUSG00000037025	*FOXA2*	-0.47411061	5.39326914	3.7395E-10	4.5306E-09
ENSMUSG00000039253	*FN3KRP*	-0.66266015	2.76970364	3.8109E-10	4.6057E-09
ENSMUSG00000039911	*SPSB1*	-0.64025981	3.75812366	3.8089E-10	4.6057E-09
ENSMUSG00000031134	*RBMX*	0.49822373	3.67921762	3.8262E-10	4.6204E-09
ENSMUSG00000030200	*BCL2L14*	0.70673194	4.19893814	3.8388E-10	4.628E-09
ENSMUSG00000037669	*LDAH*	0.47723346	5.08491224	4.0796E-10	4.8901E-09
ENSMUSG00000034083	*CCDC174*	-0.45537927	3.97017323	4.312E-10	5.152E-09
ENSMUSG00000025880	*SMAD7*	0.88387719	3.43341078	4.3193E-10	5.1564E-09
ENSMUSG00000057315	*ARHGAP24*	-1.3174369	1.69589495	4.3232E-10	5.1569E-09
ENSMUSG00000037349	*NUDT22*	0.55288038	2.94750561	4.4709E-10	5.3159E-09
ENSMUSG00000029552	*TES*	-0.38693218	7.03506141	4.6152E-10	5.4706E-09
ENSMUSG00000022253	*NADK2*	-0.46158088	5.59727563	4.7201E-10	5.585E-09
ENSMUSG00000039062	*ANPEP*	-0.49342777	4.86204072	4.7941E-10	5.6634E-09
ENSMUSG00000006335	*TFPT*	0.477825	3.63451411	4.8365E-10	5.709E-09
ENSMUSG00000029556	*HNF1A*	0.38780967	5.64302057	4.8596E-10	5.727E-09
ENSMUSG00000027087	*ITGAV*	0.40413216	7.23997435	4.9903E-10	5.8716E-09
ENSMUSG00000074277	*PHLDB3*	-0.40984693	4.25426434	5.103E-10	5.9898E-09
ENSMUSG00000060376	*BCKDHA*	-0.42402757	5.66074691	5.1505E-10	6.0407E-09
ENSMUSG00000037447	*ARID5A*	-0.58481861	4.12408851	5.2074E-10	6.0929E-09
ENSMUSG00000044433	*CAMSAP3*	-0.38274453	5.28091618	5.2211E-10	6.0992E-09
ENSMUSG00000096696	*ZFP960*	-0.89898833	2.00006429	5.2396E-10	6.1159E-09
ENSMUSG00000025730	*RAB40C*	-0.53940016	4.22875219	5.2765E-10	6.1492E-09
ENSMUSG00000044167	*FOXO1*	-0.52045235	6.04054985	5.2938E-10	6.1645E-09
ENSMUSG00000032478	*NME6*	0.46330571	3.93658076	5.4292E-10	6.3121E-09
ENSMUSG00000020668	*KIF3C*	-0.93152766	1.85817226	5.4725E-10	6.3524E-09
ENSMUSG00000046079	*LRRC8D*	0.4051283	6.21044446	5.5157E-10	6.3976E-09
ENSMUSG00000047648	*FBXO30*	-0.43522024	5.35723953	5.5577E-10	6.4412E-09
ENSMUSG00000026027	*STRADB*	-0.4294289	4.87923249	5.5939E-10	6.478E-09
ENSMUSG00000030852	*TACC2*	-0.40712107	5.91426773	5.6188E-10	6.5017E-09
ENSMUSG00000043262	*UEVLD*	-0.40105764	4.17729663	5.7129E-10	6.595E-09

ENSMUSG00000078716	*TMEM8B*	-1.34999751	0.12400896	5.7945E-10	6.6839E-09
ENSMUSG00000046027	*STARD5*	0.48567297	6.11599194	5.9165E-10	6.814E-09
ENSMUSG00000031824	*6430548M08RIK*	0.94644732	3.76765789	5.9697E-10	6.8698E-09
ENSMUSG00000075229	*MIX23*	0.39211477	4.76582578	6.0251E-10	6.9281E-09
ENSMUSG00000002477	*SNRPD1*	0.39949283	4.91177444	6.218E-10	7.1332E-09
ENSMUSG00000003032	*KLF4*	0.77425826	3.73085948	6.2975E-10	7.2187E-09
ENSMUSG00000053646	*PLXNB1*	-0.83158108	2.73556883	6.4903E-10	7.4282E-09
ENSMUSG00000070643	*SOX13*	-0.54451293	4.0265468	6.543E-10	7.4827E-09
ENSMUSG00000033313	*FBXL8*	-0.57854656	2.84279371	6.6071E-10	7.5501E-09
ENSMUSG00000050777	*TMEM37*	-0.51031908	5.45374008	6.6418E-10	7.5839E-09
ENSMUSG00000078786	*ACTMAP*	0.41338219	4.02449447	6.9835E-10	7.9247E-09
ENSMUSG00000018425	*DHX40*	-0.42936203	5.09241976	7.0759E-10	8.0172E-09
ENSMUSG00000017119	*NBR1*	-0.46432212	7.26475276	7.1906E-10	8.1096E-09
ENSMUSG00000059895	*PTP4A3*	0.45058476	4.07665204	7.4492E-10	8.3883E-09
ENSMUSG00000031626	*SORBS2*	0.49885091	8.90781781	7.6958E-10	8.6483E-09
ENSMUSG00000034926	*DHCR24*	0.45619386	7.73296252	7.7639E-10	8.716E-09
ENSMUSG00000027806	*TSC22D2*	-0.4653081	7.62637978	7.8704E-10	8.8153E-09
ENSMUSG00000055320	*TEAD1*	0.40080733	6.46461546	7.9535E-10	8.9016E-09
ENSMUSG00000038213	*TAPBPL*	0.43583747	4.79803053	8.0515E-10	8.9976E-09
ENSMUSG00000030031	*KBTBD8*	0.92195782	1.57092264	8.1787E-10	9.112E-09
ENSMUSG00000038181	*CHPF2*	-0.3874553	5.8275991	8.2819E-10	9.2044E-09
ENSMUSG00000034247	*PLEKHM1*	-0.42290473	5.39470964	8.4384E-10	9.3375E-09
ENSMUSG00000025323	*SP4*	-0.95595987	1.22113945	8.5115E-10	9.4114E-09
ENSMUSG00000026791	*SLC2A8*	-0.53312021	3.05977692	8.6303E-10	9.5212E-09
ENSMUSG00000035310		-0.39843018	4.29773212	8.6491E-10	9.5348E-09
ENSMUSG00000025026	*ADD3*	-0.52093751	2.83045863	9.1089E-10	1.0012E-08
ENSMUSG00000042444	*MINDY2*	-0.52076062	3.63145644	9.3978E-10	1.0314E-08
ENSMUSG00000040616	*TMEM51*	0.48083123	4.43629168	9.3965E-10	1.0314E-08
ENSMUSG00000032557	*UBA5*	0.38954922	5.452828	9.6358E-10	1.0567E-08
ENSMUSG00000034793	*G6PC3*	-0.47305671	3.73249765	9.8223E-10	1.0755E-08

ENSMUSG00000031618	*NR3C2*	-0.65922801	2.73624944	9.8543E-10	1.0782E-08
ENSMUSG00000004791	*PGF*	-1.15615488	1.87307638	9.8635E-10	1.0785E-08
ENSMUSG00000036819	*JMJD4*	0.40410763	4.18557719	9.9198E-10	1.0838E-08
ENSMUSG00000024052	*LPIN2*	-0.52773503	9.10152214	1.0053E-09	1.0967E-08
ENSMUSG00000029597	*SDS*	-1.27110376	2.21657321	1.0328E-09	1.1231E-08
ENSMUSG00000042507	*MIDEAS*	0.44182354	5.17422993	1.0333E-09	1.1231E-08
ENSMUSG00000050910	*CDR2L*	0.81094854	1.68205758	1.0463E-09	1.1356E-08
ENSMUSG00000019312	*GRB7*	-1.02676563	5.22644119	1.0635E-09	1.1534E-08
ENSMUSG00000024851	*PITPNM1*	-0.40247051	4.5689964	1.0778E-09	1.1672E-08
ENSMUSG00000043895	*S1PR2*	0.58091435	4.76740588	1.0838E-09	1.1719E-08
ENSMUSG00000041775	*MAPK1IP1*	-0.56628947	2.60065659	1.0893E-09	1.1761E-08
ENSMUSG00000069833	*AHNAK*	0.58592648	7.4804749	1.0937E-09	1.1785E-08
ENSMUSG00000039956	*MRAP*	-0.5491279	5.57784197	1.1019E-09	1.1854E-08
ENSMUSG00000027550	*LRRCC1*	-0.66616128	2.83548313	1.1274E-09	1.211E-08
ENSMUSG00000025912	*MYBL1*	-0.86394163	1.99646932	1.1287E-09	1.2116E-08
ENSMUSG00000031706	*RFX1*	-0.47014082	4.55587304	1.1399E-09	1.2218E-08
ENSMUSG00000041040	*FAM117B*	-0.44927508	4.31918995	1.1537E-09	1.2357E-08
ENSMUSG00000039740	*ALG2*	0.42734652	4.17934399	1.1626E-09	1.2443E-08
ENSMUSG00000025608	*PODXL*	1.1556287	1.70445239	1.1807E-09	1.2618E-08
ENSMUSG00000022414	*TAB1*	-0.55483804	3.10856557	1.1824E-09	1.2628E-08
ENSMUSG00000110353	*GM33543*	-1.43828018	0.97740492	1.2169E-09	1.2958E-08
ENSMUSG00000028318	*POLR1E*	0.4365695	4.1110713	1.2208E-09	1.2981E-08
ENSMUSG00000021209	*PPP4R4*	-0.80346985	2.37570833	1.2466E-09	1.3245E-08
ENSMUSG00000023034	*NR4A1*	-0.81659588	2.34712854	1.2478E-09	1.3249E-08
ENSMUSG00000052565	*H1F3*	2.33093255	-0.73623617	1.2602E-09	1.336E-08
ENSMUSG00000041477	*DCP1B*	-0.46142491	3.08453108	1.2812E-09	1.3574E-08
ENSMUSG00000029771	*IRF5*	0.7105918	4.24185969	1.3081E-09	1.3829E-08
ENSMUSG00000028982	*SLC25A33*	0.4386431	5.60372655	1.3144E-09	1.3885E-08
ENSMUSG00000040584	*ABCB1A*	0.79649368	4.66752256	1.3209E-09	1.3923E-08
ENSMUSG00000040213	*KYAT3*	0.55558956	4.79543909	1.3302E-09	1.4002E-08

ENSMUSG00000002384	*BMP8B*	0.61387573	4.99691177	1.3482E-09	1.4172E-08
ENSMUSG00000020534	*SHMT1*	0.38437127	4.83502776	1.3675E-09	1.4354E-08
ENSMUSG00000032311	*NRG4*	-1.1085508	6.20450095	1.4226E-09	1.4868E-08
ENSMUSG00000027510	*RBM38*	0.55539362	4.25247729	1.4681E-09	1.5311E-08
ENSMUSG00000035877	*ZHX3*	-0.6806162	5.84405633	1.4756E-09	1.5378E-08
ENSMUSG00000040957	*CABLES1*	-0.87043888	1.50376419	1.536E-09	1.5973E-08
ENSMUSG00000033454	*ZBTB1*	0.48469646	3.95857698	1.5524E-09	1.611E-08
ENSMUSG00000001151	*PCNT*	-0.3870842	4.0810775	1.5903E-09	1.648E-08
ENSMUSG00000030347	*D6WSU163E*	0.43081246	3.69185431	1.6106E-09	1.662E-08
ENSMUSG00000034854	*MFSD12*	-0.4949061	3.12142477	1.6472E-09	1.6974E-08
ENSMUSG00000030088	*ALDH1L1*	-0.52738621	6.94327571	1.6715E-09	1.72E-08
ENSMUSG00000040928	*S100PBP*	-0.40521666	4.04810407	1.6769E-09	1.7243E-08
ENSMUSG00000029554	*MAD1L1*	0.41396209	4.08619307	1.7128E-09	1.7575E-08
ENSMUSG00000027624	*EPB41L1*	-0.62025248	3.89305553	1.7645E-09	1.8068E-08
ENSMUSG00000024222	*FKBP5*	-0.55543846	6.95488852	1.8261E-09	1.8647E-08
ENSMUSG00000048550	*THNSL1*	-0.74519042	2.43639836	1.87E-09	1.9082E-08
ENSMUSG00000062075	*LMNB2*	0.40582061	4.49318021	1.8747E-09	1.9103E-08
ENSMUSG00000037926	*SSH2*	0.47264059	4.4208406	1.8742E-09	1.9103E-08
ENSMUSG00000073758	*SH3D21*	-0.78328427	2.44298293	1.8903E-09	1.9236E-08
ENSMUSG00000031077	*FADD*	0.41935273	4.32455106	1.8969E-09	1.9289E-08
ENSMUSG00000024742	*FEN1*	0.45043451	3.9115445	1.9617E-09	1.9908E-08
ENSMUSG00000032109	*NLRX1*	-0.58408207	3.05321234	2.0101E-09	2.0342E-08
ENSMUSG00000041548	*HSPB8*	0.39935405	8.35391319	2.0377E-09	2.0593E-08
ENSMUSG00000026806	*DDX31*	0.44544864	3.99287723	2.0756E-09	2.0918E-08
ENSMUSG00000074141	*IL4I1*	-1.34470201	0.73006702	2.0823E-09	2.0957E-08
ENSMUSG00000074141	*IL4I1B*	-1.34470201	0.73006702	2.0823E-09	2.0957E-08
ENSMUSG00000086150	*BACH2OS*	1.36565426	0.46543698	2.1707E-09	2.1802E-08
ENSMUSG00000031295	*PHKA2*	-0.38192932	4.38728509	2.1924E-09	2.2004E-08
ENSMUSG00000026571	*DCAF6*	-0.52853076	5.61686074	2.2801E-09	2.2854E-08
ENSMUSG00000015745	*PLEKHO1*	-0.89609041	0.82306071	2.2988E-09	2.3026E-08

ENSMUSG00000024986	*HHEX*	-0.38847997	6.82560755	2.3177E-09	2.32E-08
ENSMUSG00000044813	*SHB*	0.6628897	6.17617799	2.4294E-09	2.4251E-08
ENSMUSG00000052609	*PLEKHG3*	-0.42783678	6.733969	2.5359E-09	2.5263E-08
ENSMUSG00000029209	*GNPDA2*	-0.54121258	2.86225288	2.5484E-09	2.537E-08
ENSMUSG00000048856	*SLC25A47*	-1.02457541	4.88647633	2.5526E-09	2.5395E-08
ENSMUSG00000020641	*RSAD2*	-1.22713761	6.23183641	2.635E-09	2.6144E-08
ENSMUSG00000056313	*TCIM*	-0.7727384	2.85365476	2.6952E-09	2.6692E-08
ENSMUSG00000021211	*AKR1C12*	-0.42187807	4.34241939	2.7376E-09	2.707E-08
ENSMUSG00000045005	*FZD5*	-0.42023403	4.03453351	2.8282E-09	2.791E-08
ENSMUSG00000022442	*TTLL1*	0.38621237	3.70397115	3.1105E-09	3.0531E-08
ENSMUSG00000026409	*PFKFB2*	-0.54204905	3.2106309	3.1526E-09	3.0924E-08
ENSMUSG00000063810	*ALMS1*	-0.724393	1.6337196	3.1801E-09	3.1152E-08
ENSMUSG00000079427	*MTHFSL*	-0.41506138	4.13834606	3.2337E-09	3.1614E-08
ENSMUSG00000022994	*ADCY6*	-0.46901996	3.69049086	3.3038E-09	3.2277E-08
ENSMUSG00000044197	*GPR146*	-0.64866553	3.43128764	3.3333E-09	3.2523E-08
ENSMUSG00000042510	*AA986860*	-1.12366269	1.38639116	3.5252E-09	3.4304E-08
ENSMUSG00000027244	*ATG13*	-0.45931709	6.42793543	3.5527E-09	3.4549E-08
ENSMUSG00000028755	*CDA*	-0.45460086	4.18743291	3.6363E-09	3.5267E-08
ENSMUSG00000031845	*BCO1*	-0.41881692	6.26978251	3.6357E-09	3.5267E-08
ENSMUSG00000018920	*CXCL16*	0.53693814	6.37571414	3.8808E-09	3.7489E-08
ENSMUSG00000026077	*NPAS2*	0.86817766	2.47852199	3.8832E-09	3.7489E-08
ENSMUSG00000026622	*NEK2*	-1.19851098	0.72515893	3.9064E-09	3.7688E-08
ENSMUSG00000028599	*TNFRSF1B*	0.55188766	5.09808941	3.9979E-09	3.8394E-08
ENSMUSG00000037686	*ASPG*	-0.66270628	4.52349977	4.2117E-09	4.0315E-08
ENSMUSG00000029101	*RGS12*	-1.00614721	1.51393147	4.3133E-09	4.1155E-08
ENSMUSG00000045838	*CCDC9B*	0.86911216	3.38504741	4.3659E-09	4.163E-08
ENSMUSG00000024579	*PCYOX1L*	0.93162651	1.45051102	4.3784E-09	4.1694E-08
ENSMUSG00000024589	*NEDD4L*	-0.43475188	6.78077439	4.4373E-09	4.2173E-08
ENSMUSG00000022359	*NTAQ1*	-0.6910479	1.7462404	4.6669E-09	4.416E-08
ENSMUSG00000031641	*CBR4*	-0.40372895	4.03035488	4.8331E-09	4.5581E-08

ENSMUSG00000026489	*COQ8A*	-0.75390781	4.30757667	4.8519E-09	4.5699E-08
ENSMUSG00000030739	*MYH14*	-0.41291353	3.87095553	4.862E-09	4.5765E-08
ENSMUSG00000100457	*D830032E09RIK*	-1.43003148	0.35194787	4.8864E-09	4.5957E-08
ENSMUSG00000048578	*MLEC*	0.38985305	6.80462573	4.9188E-09	4.6211E-08
ENSMUSG00000054057	*A930004D18RIK*	1.46299669	0.28893114	5.0053E-09	4.6993E-08
ENSMUSG00000040663	*CLCF1*	-0.50849294	3.88099259	5.0368E-09	4.7259E-08
ENSMUSG00000035109	*SHC4*	0.99677559	2.56208275	5.0942E-09	4.7736E-08
ENSMUSG00000024795	*KIF20B*	-0.64117278	1.80198901	5.1814E-09	4.8523E-08
ENSMUSG00000003581	*RNF215*	-0.51356165	3.33200125	5.262E-09	4.9184E-08
ENSMUSG00000042331	*SPECC1*	-0.40327637	5.80335075	5.2827E-09	4.9345E-08
ENSMUSG00000031889	*PHAF1*	-0.62155379	5.92656484	5.3615E-09	5.0018E-08
ENSMUSG00000018339	*GPX3*	-0.81104067	1.98489018	5.4059E-09	5.04E-08
ENSMUSG00000002825	*QTRT1*	0.46495264	4.14816322	5.4463E-09	5.0745E-08
ENSMUSG00000054072	*IIGP1*	-1.02082518	5.20374638	5.4576E-09	5.0817E-08
ENSMUSG00000018774	*CD68*	-0.45747842	7.60397905	5.5501E-09	5.1521E-08
ENSMUSG00000029650	*SLC46A3*	-0.91183384	1.48061527	5.6566E-09	5.2471E-08
ENSMUSG00000029053	*PRKCZ*	-0.39480871	3.97852638	5.7339E-09	5.312E-08
ENSMUSG00000026304	*RAB17*	0.45058251	3.96114606	6.0449E-09	5.5791E-08
ENSMUSG00000026380	*TFCP2L1*	-0.54066354	4.12525242	6.2042E-09	5.7154E-08
ENSMUSG00000118295		-1.4031317	-0.18142091	6.3006E-09	5.786E-08
ENSMUSG00000034255	*ARHGAP27*	-0.48674034	5.31392051	6.6243E-09	6.0719E-08
ENSMUSG00000025154	*ARHGAP19*	-1.09993941	0.14499273	6.6599E-09	6.0969E-08
ENSMUSG00000039232	*STX11*	1.52130249	1.7624418	6.7254E-09	6.1492E-08
ENSMUSG00000027699	*ECT2*	-0.81609365	0.86404291	6.7339E-09	6.1531E-08
ENSMUSG00000024232	*BAMBI*	-1.24852286	0.10106285	6.7627E-09	6.1756E-08
ENSMUSG00000038797	*ZSCAN2*	-0.63373449	1.958609	6.7731E-09	6.1813E-08
ENSMUSG00000021250	*FOS*	-1.53230006	1.59214009	6.8329E-09	6.2281E-08
ENSMUSG00000067931	*ZFP948*	0.52564696	4.06675562	6.9959E-09	6.3727E-08
ENSMUSG00000004561	*METTL17*	0.50815301	3.09414813	7.233E-09	6.5764E-08
ENSMUSG00000023147	*GET1*	0.38692464	4.108603	7.426E-09	6.7269E-08

ENSMUSG00000096740	*LBHD1*	0.79373043	2.70088244	7.4599E-09	6.7535E-08
ENSMUSG00000021147	*WDR37*	0.40699245	4.09462538	7.6111E-09	6.8819E-08
ENSMUSG00000037461	*INTS7*	0.38784984	4.75589264	7.6662E-09	6.9275E-08
ENSMUSG00000097101	*1810034E14RIK*	0.74326822	2.4163835	8.1213E-09	7.3117E-08
ENSMUSG00000026135	*ZFP142*	0.47205723	4.63498587	8.3835E-09	7.5431E-08
ENSMUSG00000071537	*KLRG2*	1.15514798	1.03337738	8.5216E-09	7.658E-08
ENSMUSG00000078317	*F8A*	-0.46448228	3.52717386	8.8282E-09	7.9045E-08
ENSMUSG00000078994	*ZFP429*	0.61927425	2.57497335	9.4489E-09	8.4089E-08
ENSMUSG00000051984	*SEC31B*	-0.62798234	2.59893285	9.6568E-09	8.5836E-08
ENSMUSG00000046603	*TCAIM*	-0.44319224	3.17429783	1.0173E-08	9.0044E-08
ENSMUSG00000020262	*ADARB1*	-0.42571909	5.50091339	1.0207E-08	9.0183E-08
ENSMUSG00000114025		-0.812269	1.085682	1.0346E-08	9.1349E-08
ENSMUSG00000000282	*MNT*	-0.41216714	6.03724382	1.0785E-08	9.5E-08
ENSMUSG00000028089	*CHD1L*	-0.3835478	4.41858686	1.0951E-08	9.6118E-08
ENSMUSG00000071669	*SNX29*	-0.88056505	1.58593254	1.1429E-08	9.9967E-08
ENSMUSG00000042549	*MAP2K3OS*	0.76373382	2.33918273	1.143E-08	9.9967E-08
ENSMUSG00000005836	*GATA6*	-0.43867492	4.83652555	1.1533E-08	1.0074E-07
ENSMUSG00000038533	*CBFA2T2*	-0.3957987	3.98666743	1.1734E-08	1.0214E-07
ENSMUSG00000028101	*PIAS3*	-0.47016098	3.54003186	1.1965E-08	1.0402E-07
ENSMUSG00000028766	*ALPL*	0.71275645	3.95059764	1.2588E-08	1.0924E-07
ENSMUSG00000028688	*TOE1*	0.39275579	3.85984177	1.264E-08	1.0963E-07
ENSMUSG00000026814	*ENG*	0.65039519	3.17997042	1.3066E-08	1.13E-07
ENSMUSG00000035914	*CD276*	0.58282666	3.52721402	1.3167E-08	1.1373E-07
ENSMUSG00000060639	*H4C9*	1.38756664	0.66579125	1.3701E-08	1.1814E-07
ENSMUSG00000052271	*BHLHA15*	2.22529467	0.20604523	1.5091E-08	1.2943E-07
ENSMUSG00000024037	*WDR4*	0.43106193	5.26045974	1.5273E-08	1.3092E-07
ENSMUSG00000044361	*BC024139*	-1.03744954	0.14307517	1.6695E-08	1.4262E-07
ENSMUSG00000041378	*CLDN5*	0.94669608	1.11779408	1.7E-08	1.4505E-07
ENSMUSG00000063889	*CREM*	0.45211074	3.79519473	1.7156E-08	1.4621E-07
ENSMUSG00000032643	*FHL3*	0.48468117	5.5230474	1.7289E-08	1.4717E-07

ENSMUSG00000042379	*ESM1*	-0.81912322	3.37896873	1.7766E-08	1.5097E-07
ENSMUSG00000042766	*TRIM46*	0.89208183	2.75031993	1.7848E-08	1.5145E-07
ENSMUSG00000033943	*MGA*	0.37868283	5.96533546	1.7904E-08	1.5179E-07
ENSMUSG00000027035	*CERS6*	-0.42860403	4.083191	1.8208E-08	1.5401E-07
ENSMUSG00000043587	*PXYLP1*	-0.46593939	3.44834734	1.8269E-08	1.5444E-07
ENSMUSG00000074578	*ZFAS1*	-0.47725656	5.23722518	1.8302E-08	1.5463E-07
ENSMUSG00000038332	*SESN1*	-0.88464214	4.01217162	1.8545E-08	1.5659E-07
ENSMUSG00000030279	*C2CD5*	0.60520753	3.0054788	1.8885E-08	1.5928E-07
ENSMUSG00000009687	*FXYD5*	1.15159271	0.66268557	1.9157E-08	1.612E-07
ENSMUSG00000010760	*PHLDA2*	1.40399241	1.14053421	1.9214E-08	1.6159E-07
ENSMUSG00000034684	*SEMA3F*	0.89247038	1.66647111	1.9501E-08	1.6382E-07
ENSMUSG00000002983	*RELB*	0.45474306	4.77500126	1.974E-08	1.6554E-07
ENSMUSG00000063160	*NUMBL*	-0.89062234	1.93462747	1.9836E-08	1.6616E-07
ENSMUSG00000094936	*RBM4*	0.50068101	3.62967458	1.9898E-08	1.6658E-07
ENSMUSG00000048307	*ANKRD46*	0.3835214	5.33138245	2.0057E-08	1.6781E-07
ENSMUSG00000054136	*ADM2*	1.10690259	2.69659037	2.0397E-08	1.7047E-07
ENSMUSG00000028373	*ASTN2*	-1.66587768	-0.86706621	2.0767E-08	1.7346E-07
ENSMUSG00000020604	*ARSG*	0.40279068	5.01216734	2.1335E-08	1.779E-07
ENSMUSG00000037405	*ICAM1*	1.07542132	4.91481498	2.1444E-08	1.7861E-07
ENSMUSG00000064090	*VRK2*	-0.55833611	2.14671492	2.2207E-08	1.8423E-07
ENSMUSG00000001630	*STK38L*	-0.38393421	6.25789669	2.2597E-08	1.8715E-07
ENSMUSG00000009378	*SLC16A12*	-0.45609805	3.97138502	2.3356E-08	1.9311E-07
ENSMUSG00000027690	*SLC2A2*	-0.61741196	3.9967401	2.3743E-08	1.9576E-07
ENSMUSG00000038175	*MYLIP*	-0.9127116	1.59228709	2.4814E-08	2.0356E-07
ENSMUSG00000037890	*WDR19*	-0.63546615	2.15863904	2.486E-08	2.0382E-07
ENSMUSG00000032038	*ST3GAL4*	-0.59555659	6.30355284	2.5091E-08	2.0551E-07
ENSMUSG00000115388		-0.67505866	7.08523554	2.6011E-08	2.1278E-07
ENSMUSG00000040505	*ABCG5*	-0.96938391	1.23896931	2.6113E-08	2.135E-07
ENSMUSG00000110151	*GM38416*	0.89299245	2.04500708	2.6892E-08	2.1926E-07
ENSMUSG00000001053	*N4BP3*	0.45894498	3.91678376	2.8432E-08	2.3104E-07

ENSMUSG00000116380		-1.16531573	0.11688213	2.8788E-08	2.3355E-07
ENSMUSG00000067219	*NIPAL1*	-0.50393634	4.76859169	2.9513E-08	2.3864E-07
ENSMUSG00000002265	*PEG3*	-1.2736181	0.57137956	2.9688E-08	2.3979E-07
ENSMUSG00000038622	*MED30*	-0.46115388	2.72398689	3.1015E-08	2.4941E-07
ENSMUSG00000030029	*LRIG1*	0.46772716	6.11351011	3.1242E-08	2.511E-07
ENSMUSG00000028028	*ALPK1*	-0.9439294	1.13593267	3.2176E-08	2.5832E-07
ENSMUSG00000028654	*MYCL*	1.00174851	2.54260195	3.2324E-08	2.5911E-07
ENSMUSG00000102101	*ZBTB11OS1*	-0.89696417	1.23465014	3.6644E-08	2.9196E-07
ENSMUSG00000109089	*4833411C07RIK*	-2.4524083	-0.86956435	3.8447E-08	3.0523E-07
ENSMUSG00000037552	*PLEKHG2*	-0.56763293	4.06312406	3.8685E-08	3.0672E-07
ENSMUSG00000043079	*SYNPO*	-0.82316879	4.39970336	3.9772E-08	3.15E-07
ENSMUSG00000023947	*NFKBIE*	0.71564551	2.82202358	3.9812E-08	3.1515E-07
ENSMUSG00000000957	*MMP14*	-0.40081982	6.20939839	4.0079E-08	3.1692E-07
ENSMUSG00000042118	*BHMT2*	-0.4918615	3.56333488	4.0128E-08	3.1714E-07
ENSMUSG00000035900	*GRAMD4*	0.42218694	4.34583223	4.0574E-08	3.1999E-07
ENSMUSG00000021611	*TERT*	-0.65230362	1.58066933	4.172E-08	3.283E-07
ENSMUSG00000030972	*ACSM5*	-0.60575002	2.73760411	4.3663E-08	3.4213E-07
ENSMUSG00000078429	*CTDSP2*	-0.49194155	6.15212609	4.4361E-08	3.4741E-07
ENSMUSG00000033055	*ANKRD54*	-0.41379487	3.85485494	4.548E-08	3.558E-07
ENSMUSG00000041220	*ELOVL6*	0.50553521	5.62237483	4.5649E-08	3.5679E-07
ENSMUSG00000031639	*TLR3*	-0.61759513	2.76269597	4.6E-08	3.5929E-07
ENSMUSG00000032754	*SLC8B1*	-0.38524815	5.08298513	4.607E-08	3.5964E-07
ENSMUSG00000040570	*RUNDC3B*	1.58275692	0.07650425	4.6722E-08	3.6415E-07
ENSMUSG00000044749	*ABCA6*	-0.41769071	6.31539029	4.7721E-08	3.7174E-07
ENSMUSG00000073437	*D330041H03RIK*	1.05981715	0.6196945	4.8704E-08	3.786E-07
ENSMUSG00000030161	*GABARAPL1*	-0.61140687	8.19006008	4.8772E-08	3.7892E-07
ENSMUSG00000034522	*ZFP395*	-0.74523538	3.24354269	4.9033E-08	3.8055E-07
ENSMUSG00000020492	*SKA2*	0.89984313	1.25481547	4.9181E-08	3.815E-07
ENSMUSG00000022833	*CCDC14*	-0.57077371	2.12482974	4.9627E-08	3.8414E-07
ENSMUSG00000028974	*DFFA*	0.44805289	3.79283819	5.159E-08	3.9745E-07

ENSMUSG00000033249	*HSF4*	-0.449372	3.0006784	5.2048E-08	4.0057E-07
ENSMUSG00000029687	*EZH2*	-0.47363597	2.89718244	5.3155E-08	4.0844E-07
ENSMUSG00000027478	*DNMT3B*	-0.6023759	1.80044387	5.3426E-08	4.1031E-07
ENSMUSG00000066892	*FBXL12*	-0.5479139	2.76640349	5.3891E-08	4.1323E-07
ENSMUSG00000031538	*PLAT*	-1.42570033	1.04140025	5.7133E-08	4.3627E-07
ENSMUSG00000079012	*SERPINA3M*	-0.40888251	7.83276213	5.7228E-08	4.3677E-07
ENSMUSG00000047180	*NEURL3*	1.22967739	2.50384375	5.8392E-08	4.4519E-07
ENSMUSG00000022122	*EDNRB*	-0.87999725	1.88065713	5.8627E-08	4.4675E-07
ENSMUSG00000044847	*LSM11*	-0.39856388	3.43318764	5.8901E-08	4.4813E-07
ENSMUSG00000038742	*ANGPTL6*	0.65436879	2.32856271	5.9498E-08	4.5221E-07
ENSMUSG00000042249	*GRK3*	-1.10049034	0.65643061	5.9891E-08	4.5473E-07
ENSMUSG00000047370		-0.85258472	3.70284852	6.0341E-08	4.5767E-07
ENSMUSG00000035234	*ABRAXAS1*	0.38871717	3.73066574	6.0855E-08	4.6102E-07
ENSMUSG00000041308	*SNTB2*	0.43981941	5.70591811	6.0876E-08	4.6102E-07
ENSMUSG00000026343	*GPR39*	0.39404392	5.32352787	6.1409E-08	4.6457E-07
ENSMUSG00000054717	*HMGB2*	-0.49550512	3.03197897	6.222E-08	4.6974E-07
ENSMUSG00000040717	*IL17RD*	-1.47828922	-0.35226507	6.2401E-08	4.7086E-07
ENSMUSG00000033233	*TRIM45*	-0.67346692	1.51353248	6.4069E-08	4.8295E-07
ENSMUSG00000019564	*ARID3A*	1.08490581	2.48022947	6.7232E-08	5.0472E-07
ENSMUSG00000087006	*GM13889*	1.06082512	5.95004935	6.7971E-08	5.0975E-07
ENSMUSG00000049866	*ARL4C*	-0.86560125	3.25087271	6.9822E-08	5.2176E-07
ENSMUSG00000015243	*ABCA1*	-0.53962365	5.44769966	7.0648E-08	5.274E-07
ENSMUSG00000000317	*BCL6B*	0.97470464	1.26734013	7.0615E-08	5.274E-07
ENSMUSG00000027589	*PCMTD2*	-0.4053872	4.35740564	7.269E-08	5.4182E-07
ENSMUSG00000028540	*DPH2*	0.42557157	4.57653525	7.4683E-08	5.5583E-07
ENSMUSG00000051224	*TCEANC*	-0.79304873	1.31772753	7.829E-08	5.8031E-07
ENSMUSG00000034245	*HDAC11*	-0.43672216	3.90643215	8.0955E-08	5.9886E-07
ENSMUSG00000038349	*PLCL1*	-1.31826561	0.31140088	8.2056E-08	6.0595E-07
ENSMUSG00000038507	*PARP12*	-0.39644423	5.7010874	8.3048E-08	6.1249E-07
ENSMUSG00000025220	*OGA*	0.47531628	7.07561133	8.459E-08	6.2356E-07

ENSMUSG00000042010	*ACACB*	-0.45770161	5.87821714	8.4648E-08	6.2367E-07
ENSMUSG00000073555	*GM4951*	-1.02398326	2.51850392	8.8563E-08	6.5023E-07
ENSMUSG00000024558	*MAPK4*	-0.89544356	2.09651057	8.8984E-08	6.5299E-07
ENSMUSG00000027635	*DSN1*	-0.78901092	0.98748116	8.9035E-08	6.5304E-07
ENSMUSG00000026930	*GPSM1*	-1.21259132	0.37773087	9.0205E-08	6.6096E-07
ENSMUSG00000036478	*BTG1*	0.40995938	7.4266336	9.2508E-08	6.7514E-07
ENSMUSG00000041757	*PLEKHA6*	0.78835671	3.65183905	9.3566E-08	6.8219E-07
ENSMUSG00000015702	*ANXA9*	0.45895028	2.77847448	9.384E-08	6.8351E-07
ENSMUSG00000114828	*AI463229*	-0.9156637	1.75704572	9.5217E-08	6.9285E-07
ENSMUSG00000036667	*TCAF1*	-0.4724261	2.9489004	9.8625E-08	7.1606E-07
ENSMUSG00000020770	*UNK*	-0.43955553	3.38631051	9.9331E-08	7.1993E-07
ENSMUSG00000034518	*HMGXB4*	-0.43096284	3.459179	1.0238E-07	7.4091E-07
ENSMUSG00000020415	*PTTG1*	-0.43633851	3.26008503	1.0589E-07	7.6517E-07
ENSMUSG00000058729	*LIN9*	-0.58204888	1.95008642	1.2332E-07	8.8507E-07
ENSMUSG00000042992	*BORCS5*	0.45301335	2.96472369	1.2347E-07	8.8524E-07
ENSMUSG00000027203	*DUT*	0.71711864	4.71499228	1.2343E-07	8.8524E-07
ENSMUSG00000043909	*TRP53BP1*	-0.39467191	3.4221626	1.2401E-07	8.8867E-07
ENSMUSG00000048087	*AHCYL*	-0.46828081	7.71836299	1.2439E-07	8.9099E-07
ENSMUSG00000023755	*RHEBL1*	-0.80147279	1.69909847	1.2595E-07	9.0131E-07
ENSMUSG00000032411	*TFDP2*	-0.54557437	3.23560863	1.2749E-07	9.1099E-07
ENSMUSG00000047394	*ODF3B*	-1.13493011	0.00774177	1.3164E-07	9.397E-07
ENSMUSG00000020099	*UNC5B*	0.54024842	5.74578466	1.3373E-07	9.5415E-07
ENSMUSG00000005672	*KIT*	-0.87473909	1.94485602	1.3429E-07	9.5769E-07
ENSMUSG00000038500	*PRR3*	0.38325449	3.26775554	1.3449E-07	9.5865E-07
ENSMUSG00000020381	*MRNIP*	-1.05120576	0.28348026	1.3459E-07	9.5888E-07
ENSMUSG00000034168	*IRF2BPL*	-0.44086193	5.73155218	1.3531E-07	9.6355E-07
ENSMUSG00000042659	*ARRDC4*	0.42387819	4.34240476	1.3714E-07	9.7376E-07
ENSMUSG00000063543		0.59149426	2.77437572	1.3842E-07	9.8147E-07
ENSMUSG00000078485	*PLEKHN1*	-0.40940868	3.10306846	1.3968E-07	9.8979E-07
ENSMUSG00000049580	*TSKU*	0.40248063	7.76987596	1.4166E-07	1.002E-06

ENSMUSG00000052563	*D930048N14RIK*	0.55984245	4.18235126	1.4245E-07	1.0071E-06
ENSMUSG00000024590	*LMNB1*	0.39281553	3.22341058	1.4387E-07	1.0152E-06
ENSMUSG00000032565	*NUDT16*	-0.45346515	2.79623435	1.469E-07	1.0346E-06
ENSMUSG00000029413	*NAAA*	0.77173833	1.93774203	1.5344E-07	1.077E-06
ENSMUSG00000020423	*BTG2*	-0.5192718	6.92103642	1.5645E-07	1.0971E-06
ENSMUSG00000031530	*DUSP4*	-0.90951812	4.32912075	1.5764E-07	1.1049E-06
ENSMUSG00000033530	*TTC7B*	-0.53383506	3.35886009	1.5788E-07	1.1061E-06
ENSMUSG00000054855	*RND1*	0.65882068	4.73631369	1.6092E-07	1.1252E-06
ENSMUSG00000014418	*HPS5*	0.39882241	3.56734477	1.6192E-07	1.1317E-06
ENSMUSG00000027864	*PTGFRN*	-0.52575531	3.55027171	1.6564E-07	1.1538E-06
ENSMUSG00000023206	*IL15RA*	0.56774902	3.13299599	1.656E-07	1.1538E-06
ENSMUSG00000039164	*NAIF1*	0.59394658	3.20742879	1.6592E-07	1.1547E-06
ENSMUSG00000022995	*ENAH*	1.00505221	1.87642927	1.684E-07	1.1703E-06
ENSMUSG00000038508	*GDF15*	-0.46387454	5.90969928	1.7254E-07	1.1979E-06
ENSMUSG00000044026	*SLC35G1*	0.4783733	4.21133175	1.7321E-07	1.202E-06
ENSMUSG00000034640	*TIPARP*	-0.67819391	5.18476418	1.7512E-07	1.2135E-06
ENSMUSG00000091337	*EID1*	0.46620855	3.59338753	1.885E-07	1.3013E-06
ENSMUSG00000028439	*FAM219A*	-0.39130745	3.36784066	1.9235E-07	1.3254E-06
ENSMUSG00000061393	*ACVR2B*	-0.55697074	2.14928417	1.9264E-07	1.3268E-06
ENSMUSG00000105692		0.69945568	1.84516013	1.9273E-07	1.3268E-06
ENSMUSG00000021594	*SRD5A1*	0.42259868	3.04589346	1.9284E-07	1.3269E-06
ENSMUSG00000030287	*ITPR2*	-0.39240916	4.45779325	1.9704E-07	1.3526E-06
ENSMUSG00000042810	*KRBA1*	-0.39188203	3.46496404	1.9864E-07	1.3628E-06
ENSMUSG00000020623	*MAP2K6*	-1.01918124	0.62038609	1.9906E-07	1.3646E-06
ENSMUSG00000063953	*AMD2*	0.70129792	2.69985299	2.0289E-07	1.3902E-06
ENSMUSG00000026177	*SLC11A1*	1.04813229	1.30750677	2.0471E-07	1.4021E-06
ENSMUSG00000044313	*MAB21L3*	1.92234738	0.67287618	2.0551E-07	1.4062E-06
ENSMUSG00000090622	*A930033H14RIK*	-1.05643538	0.92693825	2.0733E-07	1.4167E-06
ENSMUSG00000073490	*IFI207*	-0.9524623	1.2775649	2.0906E-07	1.4245E-06
ENSMUSG00000029798	*HERC6*	-0.38804558	3.93002791	2.1557E-07	1.4648E-06

ENSMUSG00000000876	*PXMP4*	-0.61472099	3.73147032	2.168E-07	1.4725E-06
ENSMUSG00000029910	*MAD2L1*	-0.57164869	2.41466265	2.1771E-07	1.478E-06
ENSMUSG00000027243	*HARBI1*	-0.40139617	3.93352615	2.1887E-07	1.4846E-06
ENSMUSG00000030470	*CSRP3*	-1.45466541	-0.50372358	2.2299E-07	1.509E-06
ENSMUSG00000025991	*CPS1*	-0.75233682	9.21317642	2.2806E-07	1.5411E-06
ENSMUSG00000049858	*SUOX*	-0.41045194	3.55788068	2.3547E-07	1.5883E-06
ENSMUSG00000018217	*PMP22*	1.11591696	0.68516002	2.3765E-07	1.6022E-06
ENSMUSG00000037572	*WDHD1*	0.5118638	2.26940216	2.4374E-07	1.6403E-06
ENSMUSG00000032066	*BCO2*	-0.89451502	1.5487529	2.4941E-07	1.6769E-06
ENSMUSG00000021185	*DGLUCY*	-0.43622459	5.43442633	2.514E-07	1.6895E-06
ENSMUSG00000022292	*RRM2B*	-0.50662105	2.8332103	2.5205E-07	1.6931E-06
ENSMUSG00000050471	*FAM118B*	0.40151195	3.76712173	2.5672E-07	1.7229E-06
ENSMUSG00000015143	*ACTN1*	0.49661384	7.76565912	2.6496E-07	1.7749E-06
ENSMUSG00000068335	*DOK1*	-1.08171569	-0.01957577	2.6642E-07	1.7839E-06
ENSMUSG00000106951	*5930430L01RIK*	-0.88874053	0.44593834	2.6681E-07	1.7857E-06
ENSMUSG00000075514	*GM13375*	-0.87364569	0.83237268	2.769E-07	1.8473E-06
ENSMUSG00000025902	*SOX17*	-1.2295943	0.38348609	2.7954E-07	1.8613E-06
ENSMUSG00000097164		-0.76516851	1.2031111	2.7963E-07	1.8613E-06
ENSMUSG00000062012	*ZFP13*	-0.83561445	1.16249564	2.8305E-07	1.8824E-06
ENSMUSG00000022512	*CLDN1*	-0.74750259	5.32918441	2.8817E-07	1.9147E-06
ENSMUSG00000034634	*LY6D*	-0.60635453	4.97956211	2.8964E-07	1.9236E-06
ENSMUSG00000020009	*IFNGR1*	0.46457073	6.44420353	2.9286E-07	1.9432E-06
ENSMUSG00000020528	*PRPSAP2*	0.43393396	2.96243797	3.0651E-07	2.0292E-06
ENSMUSG00000066800	*RNASEL*	-1.51333013	-0.9625171	3.1104E-07	2.0564E-06
ENSMUSG00000013483	*CARD14*	-1.22999227	-0.42206638	3.2074E-07	2.1158E-06
ENSMUSG00000035299	*MID1*	-0.75087734	3.17438308	3.2691E-07	2.1546E-06
ENSMUSG00000064141	*ZFP69*	-0.67244384	1.48414959	3.3026E-07	2.1737E-06
ENSMUSG00000045751	*MMS22L*	-0.53951142	2.24885042	3.3447E-07	2.1994E-06
ENSMUSG00000026779	*MASTL*	-0.84936768	0.44842189	3.3522E-07	2.2034E-06
ENSMUSG00000048106		0.42496695	2.88363904	3.413E-07	2.2403E-06

ENSMUSG00000115124		-1.73842331	-1.00998404	3.4622E-07	2.2686E-06
ENSMUSG00000039853	*TRIM14*	-0.4441552	3.61092834	3.4818E-07	2.2804E-06
ENSMUSG00000022945	*CHAF1B*	-0.46314778	2.58474073	3.4997E-07	2.2911E-06
ENSMUSG00000028803	*NIPAL3*	-0.41974614	3.96183887	3.5034E-07	2.2925E-06
ENSMUSG00000022105	*RB1*	-0.42926982	3.26487711	3.5119E-07	2.297E-06
ENSMUSG00000002603	*TGFB1*	0.58052135	2.05221259	3.5696E-07	2.3327E-06
ENSMUSG00000054115	*SKP2*	0.69640556	1.84719383	3.5988E-07	2.3507E-06
ENSMUSG00000041189	*CHRNB1*	0.38681403	4.02575337	3.6204E-07	2.3627E-06
ENSMUSG00000020032	*NUAK1*	0.68768228	2.26205274	3.668E-07	2.3917E-06
ENSMUSG00000036904	*FZD8*	-0.48066656	1.92264344	3.7568E-07	2.442E-06
ENSMUSG00000020812	*SNHG16*	-0.52426016	2.72514153	3.767E-07	2.4465E-06
ENSMUSG00000038425	*POLI*	-0.43850521	2.59123418	3.766E-07	2.4465E-06
ENSMUSG00000103243	*LCE1D*	-1.6532318	-1.38915702	3.7899E-07	2.4592E-06
ENSMUSG00000099966		-0.58375772	2.08983597	3.8762E-07	2.5119E-06
ENSMUSG00000030800	*PRSS8*	-0.57509483	3.19922179	3.881E-07	2.5138E-06
ENSMUSG00000024235	*MAP3K8*	-0.75750228	0.71351875	3.897E-07	2.522E-06
ENSMUSG00000059540	*TCEA2*	-0.89969709	0.54282642	4.0809E-07	2.6368E-06
ENSMUSG00000039670	*OXLD1*	-0.66699415	1.36209874	4.1888E-07	2.6989E-06
ENSMUSG00000021260	*HHIPL1*	0.9413379	3.32408595	4.3019E-07	2.7681E-06
ENSMUSG00000020063	*SIRT1*	-0.38426553	4.12756987	4.323E-07	2.7781E-06
ENSMUSG00000002944	*CD36*	-0.38106098	3.66546743	4.372E-07	2.8047E-06
ENSMUSG00000027459	*FAM110A*	0.48086013	3.17446252	4.4042E-07	2.8228E-06
ENSMUSG00000032661	*OAS3*	-1.09479214	0.65081595	4.4799E-07	2.8676E-06
ENSMUSG00000020893	*PER1*	-0.47080557	6.4224958	4.6813E-07	2.99E-06
ENSMUSG00000028088	*FMO5*	-0.56484712	6.47874142	4.6914E-07	2.9951E-06
ENSMUSG00000041777	*CIR1*	-0.38946192	4.38469533	4.7261E-07	3.0147E-06
ENSMUSG00000041642	*KIF21B*	-1.0607106	0.80152605	4.9061E-07	3.12E-06
ENSMUSG00000025464	*PAOX*	-0.54785822	3.07069899	4.9472E-07	3.1435E-06
ENSMUSG00000010067	*RASSF1*	0.46697254	5.72334006	5.2029E-07	3.3002E-06
ENSMUSG00000022528	*HES1*	0.55144877	3.0862259	5.213E-07	3.3051E-06

ENSMUSG00000042686	*JPH1*	-0.67031875	1.65893543	5.2916E-07	3.3492E-06
ENSMUSG00000026999	*NUP35*	0.43478803	3.27808867	5.3004E-07	3.3519E-06
ENSMUSG00000019850	*TNFAIP3*	0.55353984	4.69254116	5.324E-07	3.3639E-06
ENSMUSG00000037408	*CNNM4*	-0.4427039	3.47785377	5.3625E-07	3.3868E-06
ENSMUSG00000022464	*SLC38A4*	-0.38162333	8.76923697	5.4905E-07	3.4646E-06
ENSMUSG00000047443	*ERFE*	-0.80874808	1.29211083	5.4982E-07	3.4681E-06
ENSMUSG00000033065	*PFKM*	-0.51850263	2.90089115	5.5581E-07	3.5028E-06
ENSMUSG00000040669	*PHC1*	-0.42114569	2.92405056	5.5975E-07	3.5261E-06
ENSMUSG00000050069	*GREM2*	-1.90062022	-0.04475246	5.6056E-07	3.5297E-06
ENSMUSG00000062421	*ARF2*	0.44243434	3.44868689	5.6806E-07	3.5692E-06
ENSMUSG00000024151	*MSH2*	-0.37862893	3.73153267	6.1781E-07	3.8522E-06
ENSMUSG00000030878	*CDR2*	0.48221676	3.5190749	6.2117E-07	3.8715E-06
ENSMUSG00000031549	*IDO2*	-0.68942601	2.16896331	6.2567E-07	3.8962E-06
ENSMUSG00000047728	*LY6G2*	-0.71178211	3.24018686	6.3181E-07	3.9295E-06
ENSMUSG00000021360	*GCNT2*	-0.39304767	5.20240188	6.4714E-07	4.018E-06
ENSMUSG00000051335	*GFOD1*	0.76073313	1.43081006	6.5546E-07	4.0662E-06
ENSMUSG00000074384	*AI429214*	-1.03739294	-0.00618066	7.0317E-07	4.3275E-06
ENSMUSG00000018648	*DUSP14*	0.93784154	1.85418347	7.071E-07	4.348E-06
ENSMUSG00000054517	*TRIM65*	0.66268711	3.26581626	7.1761E-07	4.4052E-06
ENSMUSG00000032714	*SYDE1*	0.65682324	4.25691689	7.3119E-07	4.483E-06
ENSMUSG00000038065	*MTURN*	-0.86219029	2.12631069	7.4707E-07	4.5708E-06
ENSMUSG00000024378	*STARD4*	-0.42414088	4.06333982	7.6124E-07	4.644E-06
ENSMUSG00000072949	*ACOT1*	-0.65992808	3.37361979	7.6786E-07	4.6824E-06
ENSMUSG00000020263	*APPL2*	-0.41350842	4.38208111	8.1966E-07	4.9715E-06
ENSMUSG00000028864	*HGF*	-1.21254607	-0.14788596	8.2747E-07	5.0147E-06
ENSMUSG00000079179		0.46269419	3.36490472	8.4079E-07	5.0868E-06
ENSMUSG00000028834	*TRIM63*	-1.82054833	-1.80946581	8.5477E-07	5.1609E-06
ENSMUSG00000086868		-1.13351067	-0.92409873	8.673E-07	5.2302E-06
ENSMUSG00000021707	*DHFR*	-0.50404835	3.26956031	8.9899E-07	5.4035E-06
ENSMUSG00000072889	*NFXL1*	0.44050714	4.44938703	9.0004E-07	5.4065E-06

ENSMUSG00000032575	*MANF*	0.55242485	6.45532023	9.0023E-07	5.4065E-06
ENSMUSG00000029445	*HPD*	-0.57280523	5.96744033	9.1977E-07	5.5149E-06
ENSMUSG00000060429	*SNTB1*	-0.50608376	5.10293253	9.4369E-07	5.6422E-06
ENSMUSG00000031562	*DCTD*	0.66714772	2.15539565	9.4347E-07	5.6422E-06
ENSMUSG00000019256	*AHR*	-0.48018045	5.06824515	9.4488E-07	5.647E-06
ENSMUSG00000090698	*APOLD1*	1.65888813	-0.35759554	9.7802E-07	5.8355E-06
ENSMUSG00000040536	*NECAB1*	-0.60651774	2.69460716	9.804E-07	5.8474E-06
ENSMUSG00000024451	*ARAP3*	0.72103999	1.96378537	1.0127E-06	6.0227E-06
ENSMUSG00000040428	*PLEKHA4*	-1.34315773	-0.22196843	1.0401E-06	6.1732E-06
ENSMUSG00000030737	*SLCO2B1*	-0.50971522	3.39946117	1.0913E-06	6.4537E-06
ENSMUSG00000079442	*ST6GALNAC4*	-0.6787744	1.54424004	1.1254E-06	6.6315E-06
ENSMUSG00000026972	*ARRDC1*	-0.41321616	2.9450006	1.1329E-06	6.6623E-06
ENSMUSG00000041471	*SHLD2*	-0.54239293	4.01561873	1.1699E-06	6.8663E-06
ENSMUSG00000021884	*HACL1*	-0.55787057	3.3794636	1.177E-06	6.9022E-06
ENSMUSG00000020183	*CPM*	-0.8520916	0.37611944	1.1803E-06	6.9162E-06
ENSMUSG00000060090	*RP2*	-0.38216928	3.50717283	1.197E-06	6.9971E-06
ENSMUSG00000031310	*ZMYM3*	-0.45785145	3.42205673	1.2105E-06	7.0703E-06
ENSMUSG00000043822	*ADAMTSL5*	-0.42764317	2.52018881	1.2179E-06	7.1082E-06
ENSMUSG00000004655	*AQP1*	0.78936282	1.80794754	1.2624E-06	7.3531E-06
ENSMUSG00000056737	*CAPG*	-0.56184339	2.32405965	1.2753E-06	7.4196E-06
ENSMUSG00000020429	*IGFBP1*	-1.20050802	9.54407378	1.2921E-06	7.5085E-06
ENSMUSG00000024901	*PELI3*	-0.67829791	0.9349884	1.2931E-06	7.5108E-06
ENSMUSG00000019948	*ACTR6*	-0.40083389	2.78132462	1.3027E-06	7.561E-06
ENSMUSG00000036053	*FMNL2*	-0.40188968	5.53035829	1.3298E-06	7.7087E-06
ENSMUSG00000043467	*ZBTB37*	-0.3911148	2.79925792	1.3365E-06	7.7446E-06
ENSMUSG00000034163	*ZFC3H1*	0.47104595	5.45335263	1.3432E-06	7.7808E-06
ENSMUSG00000033356	*PUS7L*	0.48057105	2.64225506	1.3695E-06	7.9205E-06
ENSMUSG00000001120	*PCBP3*	-0.5424685	2.04778638	1.4043E-06	8.112E-06
ENSMUSG00000028438	*KIF24*	-0.94932268	-0.03533679	1.4298E-06	8.2529E-06
ENSMUSG00000028789	*AZIN2*	-0.59688205	2.322941	1.434E-06	8.2706E-06

ENSMUSG00000073096	*LRRC61*	-0.4203195	3.07850649	1.4337E-06	8.2706E-06
ENSMUSG00000024118	*TEDC2*	-0.46212762	2.98704186	1.4395E-06	8.2992E-06
ENSMUSG00000042523	*DNAL1*	-0.69177468	0.93146677	1.4818E-06	8.5279E-06
ENSMUSG00000029722	*AGFG2*	-0.3938196	5.71844157	1.5123E-06	8.6933E-06
ENSMUSG00000031520	*VEGFC*	0.55776009	1.89619693	1.528E-06	8.7725E-06
ENSMUSG00000042854	*TRP53RKB*	0.50335459	2.24683761	1.5349E-06	8.8073E-06
ENSMUSG00000029135	*FOSL2*	-0.44714986	5.63553396	1.5591E-06	8.9348E-06
ENSMUSG00000085492	*TRMT61B*	0.70656867	1.27174964	1.5595E-06	8.9348E-06
ENSMUSG00000056459	*ZBTB25*	0.52888856	2.24670213	1.5619E-06	8.9448E-06
ENSMUSG00000034936	*ARL4D*	-0.97507928	5.56346566	1.6335E-06	9.3334E-06
ENSMUSG00000087113	*GM11714*	0.53182618	2.55466665	1.6759E-06	9.5532E-06
ENSMUSG00000019817	*PLAGL1*	-0.87268785	0.60003572	1.6833E-06	9.5842E-06
ENSMUSG00000000385	*TMPRSS2*	-0.52787602	5.75070207	1.7132E-06	9.7256E-06
ENSMUSG00000019888	*MGAT4C*	-2.12469984	-1.58315217	1.7194E-06	9.7556E-06
ENSMUSG00000027381	*BCL2L11*	-0.64752928	3.08450398	1.7238E-06	9.773E-06
ENSMUSG00000028104	*POLR3GL*	-0.39113368	2.89595052	1.7627E-06	9.9782E-06
ENSMUSG00000042215	*BAG2*	0.76157916	4.36248998	1.776E-06	1.0042E-05
ENSMUSG00000090641	*ZFP712*	-0.94035357	-0.13405061	1.8207E-06	1.0279E-05
ENSMUSG00000037007	*ZFP113*	-0.40302742	2.99486843	1.8433E-06	1.0395E-05
ENSMUSG00000024666	*TMEM138*	0.44035376	2.57959894	1.8594E-06	1.0481E-05
ENSMUSG00000063488	*ZKSCAN7*	-0.40294495	3.1184377	1.8785E-06	1.0568E-05
ENSMUSG00000093930	*HMGCS1*	-0.39105985	7.10515366	1.8916E-06	1.0638E-05
ENSMUSG00000033831	*FGB*	-0.46476396	10.1531541	1.9024E-06	1.0691E-05
ENSMUSG00000020205	*PHLDA1*	0.69059899	5.94428044	1.9255E-06	1.0812E-05
ENSMUSG00000057969	*SEMA3B*	-0.75961093	1.07835828	1.9365E-06	1.0866E-05
ENSMUSG00000057722	*LEPR*	-1.00178984	-0.02339898	1.9476E-06	1.0913E-05
ENSMUSG00000026799	*MED27*	0.46253453	2.68605261	1.9512E-06	1.0927E-05
ENSMUSG00000033377	*PALMD*	-0.53052722	5.58999991	1.9562E-06	1.0943E-05
ENSMUSG00000037921	*DDX60*	-0.7407677	1.77086403	1.9681E-06	1.1001E-05
ENSMUSG00000115009		0.90839029	1.63559091	1.9686E-06	1.1001E-05

ENSMUSG00000110397		-2.39995248	-1.53452553	1.9707E-06	1.1006E-05
ENSMUSG00000055760	*GEMIN6*	0.40183895	3.05514593	2.0301E-06	1.1309E-05
ENSMUSG00000005413	*HMOX1*	0.97594576	7.61240491	2.0643E-06	1.1495E-05
ENSMUSG00000051146	*CAMK2N2*	-0.58921918	2.02261616	2.0669E-06	1.1505E-05
ENSMUSG00000032380	*DAPK2*	-0.52724562	4.82648124	2.0902E-06	1.1613E-05
ENSMUSG00000031378	*ABCD1*	-0.50239142	2.74588291	2.1068E-06	1.1701E-05
ENSMUSG00000060301	*2610008E11RIK*	-0.58224784	1.96447847	2.1545E-06	1.1938E-05
ENSMUSG00000035121	*NEIL2*	-0.81673437	0.85363303	2.1571E-06	1.1948E-05
ENSMUSG00000008384	*SERTAD1*	0.44650748	4.10822451	2.1608E-06	1.1964E-05
ENSMUSG00000079470	*UTP14B*	0.41960413	3.02777799	2.1628E-06	1.1971E-05
ENSMUSG00000026399	*CD55*	1.00881081	0.54014986	2.1896E-06	1.21E-05
ENSMUSG00000020102	*SLC16A7*	-0.42415636	3.7881167	2.2132E-06	1.2218E-05
ENSMUSG00000028977	*CASZ1*	-0.47940913	2.76926884	2.2346E-06	1.2322E-05
ENSMUSG00000028001	*FGA*	-0.62664087	9.5709497	2.2445E-06	1.2367E-05
ENSMUSG00000057156	*HOMEZ*	-0.41136243	2.43811402	2.3024E-06	1.2653E-05
ENSMUSG00000052302	*TBC1D30*	-0.68303231	2.28138355	2.3228E-06	1.2755E-05
ENSMUSG00000033618	*MAP3K13*	-0.42499199	4.85479662	2.3316E-06	1.2794E-05
ENSMUSG00000042647	*ACAD12*	-0.3833936	3.92034453	2.3315E-06	1.2794E-05
ENSMUSG00000094786		0.70110045	2.55062019	2.3623E-06	1.2953E-05
ENSMUSG00000020657	*DNAJC27*	-0.47202469	1.88475782	2.5295E-06	1.3767E-05
ENSMUSG00000022623	*SHANK3*	0.67427167	1.72746329	2.5288E-06	1.3767E-05
ENSMUSG00000043687	*1190005I06RIK*	-0.55801941	1.86995133	2.533E-06	1.3777E-05
ENSMUSG00000058254	*TSPAN7*	-0.45725067	3.44317038	2.6843E-06	1.4583E-05
ENSMUSG00000003721	*INSIG2*	-0.4636958	7.34866052	2.6889E-06	1.4602E-05
ENSMUSG00000052917	*SENP7*	-0.46050445	2.35915843	2.7104E-06	1.4708E-05
ENSMUSG00000055612	*CDCA7*	0.61868621	2.25118003	2.7124E-06	1.4709E-05
ENSMUSG00000111521		1.0180389	0.4800303	2.7303E-06	1.4789E-05
ENSMUSG00000039158	*AKNA*	1.15878403	0.22254357	2.8008E-06	1.5142E-05
ENSMUSG00000030747	*DGAT2*	0.39621456	7.42597267	2.8721E-06	1.5499E-05
ENSMUSG00000027397	*SLC20A1*	-0.58138805	7.30434079	2.8767E-06	1.5519E-05

ENSMUSG00000056267	*CEP70*	-0.55358915	1.42462753	2.8924E-06	1.5597E-05
ENSMUSG00000004748	*MTFP1*	0.40172508	2.77439752	2.9584E-06	1.5906E-05
ENSMUSG00000113581	*DIO3OS*	-2.19658872	-1.95938373	3.002E-06	1.6135E-05
ENSMUSG00000040152	*THBS1*	1.67318317	3.8703265	3.0823E-06	1.6548E-05
ENSMUSG00000046806	*CYREN*	0.55199999	2.38143962	3.1309E-06	1.6785E-05
ENSMUSG00000026088	*MITD1*	0.45927499	2.87815877	3.1464E-06	1.6862E-05
ENSMUSG00000006356	*CRIP2*	0.43906332	5.99218891	3.1811E-06	1.7029E-05
ENSMUSG00000054619	*METTL7A1*	-0.41408577	5.28482982	3.2203E-06	1.722E-05
ENSMUSG00000097772	*5430416N02RIK*	0.53093265	3.179632	3.3358E-06	1.7793E-05
ENSMUSG00000039166	*AKAP7*	-0.45292684	2.67154743	3.4394E-06	1.8312E-05
ENSMUSG00000027346	*GPCPD1*	-0.44108637	4.49528646	3.461E-06	1.842E-05
ENSMUSG00000037725	*CKAP2*	-0.69431248	1.66754129	3.5394E-06	1.8797E-05
ENSMUSG00000091144	*PHF11C*	-0.71576296	2.95832629	3.5796E-06	1.8996E-05
ENSMUSG00000054426	*A930005H10RIK*	0.42131314	2.36103937	3.6218E-06	1.92E-05
ENSMUSG00000047501	*CLDN4*	-0.7138105	6.67597247	3.6723E-06	1.9432E-05
ENSMUSG00000028011	*TDO2*	-0.9456227	6.08512716	3.6777E-06	1.9454E-05
ENSMUSG00000037525	*BCDIN3D*	-0.42507962	2.77397813	3.8177E-06	2.0151E-05
ENSMUSG00000080797	*GM15760*	-0.93578737	0.14446308	3.8709E-06	2.0417E-05
ENSMUSG00000030935	*ACSM3*	-0.6269773	1.61750391	3.9178E-06	2.0649E-05
ENSMUSG00000114255		0.38381769	2.51689569	3.9823E-06	2.0974E-05
ENSMUSG00000082361	*BTC*	-0.46033711	4.8085328	4.036E-06	2.1227E-05
ENSMUSG00000070031	*SP140*	-0.55860351	1.7391747	4.0915E-06	2.1472E-05
ENSMUSG00000025747	*TYMS*	0.40495143	2.53157876	4.1421E-06	2.1707E-05
ENSMUSG00000054836	*ELP6*	0.49522104	2.106753	4.5169E-06	2.3495E-05
ENSMUSG00000039621	*PREX1*	-0.92940518	0.18928436	4.5615E-06	2.3702E-05
ENSMUSG00000052688	*RAB7B*	0.8435027	1.96805595	4.5708E-06	2.3725E-05
ENSMUSG00000021943	*GDF10*	-1.54596015	-0.73683626	4.6398E-06	2.404E-05
ENSMUSG00000006154	*EPS8L1*	-0.93326926	1.74909509	4.6675E-06	2.4158E-05
ENSMUSG00000036022	*PABIR2*	-0.48282295	1.27091951	4.6949E-06	2.4292E-05
ENSMUSG00000079450	*CLDN34C1*	-1.33811231	-0.54525955	4.713E-06	2.4368E-05

ENSMUSG00000034731	*DGKH*	-0.47940245	2.3892953	4.7986E-06	2.4767E-05
ENSMUSG00000074643	*CPNE1*	-0.44096187	5.00485869	4.8209E-06	2.4856E-05
ENSMUSG00000052942	*GLIS3*	-0.69802953	2.535979	4.938E-06	2.5415E-05
ENSMUSG00000031665	*SALL1*	-0.41964107	6.882734	4.941E-06	2.5422E-05
ENSMUSG00000048764	*TMPRSS11F*	-0.87942853	1.14792659	4.9758E-06	2.5592E-05
ENSMUSG00000113450	*ZFP935*	-0.44291	2.43770496	5.0699E-06	2.6021E-05
ENSMUSG00000002486	*TCHP*	-0.45257922	2.7893774	5.0949E-06	2.614E-05
ENSMUSG00000033318	*GSTT2*	-0.42963326	3.55509887	5.2331E-06	2.6793E-05
ENSMUSG00000102752	*GM7694*	-0.63802097	2.02276171	5.3541E-06	2.7374E-05
ENSMUSG00000027160	*CCDC34*	-0.40409984	2.43462562	5.3673E-06	2.7432E-05
ENSMUSG00000038403	*HJV*	-0.7605897	3.20676399	5.4915E-06	2.8018E-05
ENSMUSG00000029156	*SGCB*	-0.41205567	2.77216904	5.5312E-06	2.8182E-05
ENSMUSG00000050786	*CCDC126*	0.53540071	1.30271852	5.548E-06	2.8258E-05
ENSMUSG00000087259	*2610035D17RIK*	-0.96216509	0.40117751	5.5807E-06	2.8379E-05
ENSMUSG00000087259	*LOC115487747*	-0.96216509	0.40117751	5.5807E-06	2.8379E-05
ENSMUSG00000017868	*SGK2*	0.53076148	4.41281736	5.5815E-06	2.8379E-05
ENSMUSG00000042677	*ZC3H12A*	-0.51597003	2.37469096	5.6339E-06	2.8625E-05
ENSMUSG00000041920	*SLC16A6*	0.65032542	4.68608029	5.7222E-06	2.9044E-05
ENSMUSG00000014361	*MERTK*	-0.47948185	2.33817387	5.7824E-06	2.9319E-05
ENSMUSG00000020863	*LUC7L3*	0.41914131	5.24056111	6.0202E-06	3.0368E-05
ENSMUSG00000048388	*FAM171B*	0.91929238	2.70935447	6.0746E-06	3.0632E-05
ENSMUSG00000066170		-0.95794229	0.61541964	6.1017E-06	3.0747E-05
ENSMUSG00000030761	*MYO7A*	0.40595232	3.78193157	6.1668E-06	3.1065E-05
ENSMUSG00000006403	*ADAMTS4*	0.91197822	1.349435	6.2324E-06	3.1363E-05
ENSMUSG00000035697	*ARHGAP45*	0.95475695	0.01701864	6.2469E-06	3.1425E-05
ENSMUSG00000001627	*IFRD1*	0.62694636	7.62858115	6.275E-06	3.1534E-05
ENSMUSG00000026259	*NGEF*	-0.57620268	2.61508564	6.3649E-06	3.1964E-05
ENSMUSG00000101655	*2310040G24RIK*	0.85127812	0.49224601	6.4752E-06	3.2473E-05
ENSMUSG00000034595	*PPP1R18*	0.75318049	1.90739692	6.5328E-06	3.274E-05
ENSMUSG00000109093	*GM39079*	-0.99210797	1.40869276	6.7344E-06	3.3658E-05

ENSMUSG00000022911	*ARL13B*	-0.3872474	3.22969496	6.9274E-06	3.4552E-05
ENSMUSG00000089712	*GM15889*	-1.13720391	1.68312903	7.0084E-06	3.4921E-05
ENSMUSG00000085241	*SNHG3*	-0.42125254	2.92270502	7.0409E-06	3.5059E-05
ENSMUSG00000031995	*ST14*	-0.75827974	1.41780402	7.0634E-06	3.5147E-05
ENSMUSG00000027276	*JAG1*	0.57023756	2.72660681	7.073E-06	3.5183E-05
ENSMUSG00000074802	*GAS2L3*	-0.58531823	2.17224289	7.1595E-06	3.5577E-05
ENSMUSG00000004661	*ARID3B*	-0.38506862	2.83266172	7.1783E-06	3.5647E-05
ENSMUSG00000026012	*CD28*	-1.64581968	-1.50769616	7.2679E-06	3.603E-05
ENSMUSG00000025815	*DHTKD1*	-0.55462737	3.16234268	7.2677E-06	3.603E-05
ENSMUSG00000000881	*DLG3*	-0.39246703	3.39298134	7.3255E-06	3.6267E-05
ENSMUSG00000006456	*RBM14*	0.53837808	4.98699322	7.3391E-06	3.6322E-05
ENSMUSG00000042500	*AGO4*	-0.53423339	1.63343759	7.4179E-06	3.6675E-05
ENSMUSG00000047638	*NR1H4*	-0.44497784	4.46367147	7.4631E-06	3.6874E-05
ENSMUSG00000038544	*INIP*	0.40117997	3.1892366	7.6044E-06	3.7509E-05
ENSMUSG00000042320	*PROX2*	0.65890702	1.08081681	7.6235E-06	3.759E-05
ENSMUSG00000003378	*GRIK5*	-0.69540043	1.07752647	7.6377E-06	3.7647E-05
ENSMUSG00000020707	*RNF135*	0.40266471	2.95215179	7.6888E-06	3.7874E-05
ENSMUSG00000022091	*SORBS3*	-0.90879505	3.52508788	7.7538E-06	3.8156E-05
ENSMUSG00000086881		-1.02835727	-0.31819176	7.7934E-06	3.8338E-05
ENSMUSG00000047496	*RNF152*	-0.71431882	1.87032577	7.8977E-06	3.8786E-05
ENSMUSG00000055733	*NAP1L3*	-1.34444363	-0.84449186	7.9087E-06	3.8801E-05
ENSMUSG00000038534	*OSBPL7*	-0.9173236	0.37208509	7.9063E-06	3.8801E-05
ENSMUSG00000027630	*TBL1XR1*	-0.41865881	5.04073812	7.9135E-06	3.8812E-05
ENSMUSG00000014542	*CLEC4F*	-1.3423102	-0.33003137	8.0038E-06	3.9228E-05
ENSMUSG00000032849	*ABCC4*	0.71082597	5.00908651	8.1561E-06	3.9935E-05
ENSMUSG00000043243	*NIBAN3*	0.69542698	1.27076341	8.3057E-06	4.0613E-05
ENSMUSG00000069631	*STRADA*	-0.39597121	2.53905434	8.3603E-06	4.0853E-05
ENSMUSG00000020105	*LRIG3*	-0.48623331	2.31828022	8.4298E-06	4.1165E-05
ENSMUSG00000043336	*FILIP1L*	-0.9571301	1.44455367	8.7117E-06	4.2457E-05
ENSMUSG00000097848	*GM807*	-1.62196631	-0.83002605	9.1971E-06	4.459E-05

ENSMUSG00000034121	*MKS1*	-0.44967297	2.3606484	9.198E-06	4.459E-05
ENSMUSG00000026072	*IL1R1*	-0.46119825	3.46618042	9.2414E-06	4.4756E-05
ENSMUSG00000041126	*H2AZ2*	-0.68332586	2.15202104	9.2699E-06	4.4865E-05
ENSMUSG00000027938	*CREB3L4*	1.09008328	-0.7663916	9.3166E-06	4.5061E-05
ENSMUSG00000054893	*ZFP667*	-0.86361423	0.84814173	9.3522E-06	4.5218E-05
ENSMUSG00000029094	*AFAP1*	-0.59452289	1.23860838	9.4208E-06	4.552E-05
ENSMUSG00000002504	*NHERF2*	0.5094618	2.4531144	9.5537E-06	4.6117E-05
ENSMUSG00000053062	*JAM2*	0.66306033	1.42375317	9.7631E-06	4.705E-05
ENSMUSG00000034795	*CCDC122*	0.38235908	3.41638755	9.8894E-06	4.7549E-05
ENSMUSG00000068854	*H2BC21*	0.93752289	0.79240929	1.0253E-05	4.9153E-05
ENSMUSG00000020019	*NTN4*	-0.67213638	1.44113726	1.0298E-05	4.9354E-05
ENSMUSG00000041075	*FZD7*	-0.46053767	2.95023782	1.0805E-05	5.1582E-05
ENSMUSG00000026317	*CLN8*	0.53712436	7.58969021	1.093E-05	5.2092E-05
ENSMUSG00000036273	*LRRK2*	-0.99447496	0.15201415	1.0969E-05	5.2195E-05
ENSMUSG00000022296	*BAALC*	1.32366238	-0.37662557	1.128E-05	5.3588E-05
ENSMUSG00000049086	*BMYC*	-0.61589017	1.86942846	1.1335E-05	5.3796E-05
ENSMUSG00000032431	*CRTAP*	0.38276043	4.39623152	1.1382E-05	5.4E-05
ENSMUSG00000008318	*RELT*	-1.04706488	0.02997248	1.1508E-05	5.4527E-05
ENSMUSG00000038894	*IRS2*	-0.45976668	4.92755283	1.1546E-05	5.4693E-05
ENSMUSG00000038943	*PRC1*	-1.01173852	0.09024721	1.1615E-05	5.4982E-05
ENSMUSG00000079112	*FAM90A1A*	-1.00355348	-0.59772132	1.1694E-05	5.5302E-05
ENSMUSG00000069601	*ANK3*	-0.52915162	3.66314824	1.1888E-05	5.6184E-05
ENSMUSG00000035064	*EEF2K*	0.38735644	3.80081422	1.1929E-05	5.6361E-05
ENSMUSG00000055003	*LRTM2*	-0.81169376	0.7390674	1.2184E-05	5.747E-05
ENSMUSG00000091405	*H4C14*	2.23173028	-1.04845843	1.2362E-05	5.8274E-05
ENSMUSG00000045664	*CDC42EP2*	0.94979551	0.69349882	1.2794E-05	6.0119E-05
ENSMUSG00000020387	*JADE2*	-0.39347946	3.67236128	1.307E-05	6.1354E-05
ENSMUSG00000029471	*CAMKK2*	0.45332066	2.36763446	1.3288E-05	6.2252E-05
ENSMUSG00000015312	*GADD45B*	-0.57666828	3.73008108	1.337E-05	6.2585E-05
ENSMUSG00000049536	*TCEAL1*	-0.76841738	-0.55526141	1.3768E-05	6.4324E-05

ENSMUSG00000028517	*PLPP3*	-0.43335576	4.73387552	1.3879E-05	6.478E-05
ENSMUSG00000029167	*PPARGC1A*	-0.61126184	1.74491884	1.3953E-05	6.5083E-05
ENSMUSG00000001029	*ICAM2*	1.86257516	-0.88366163	1.4178E-05	6.6043E-05
ENSMUSG00000037266	*RSRP1*	0.45069777	7.13401164	1.431E-05	6.658E-05
ENSMUSG00000032782	*CNTROB*	-0.45602111	1.91654888	1.4368E-05	6.6828E-05
ENSMUSG00000027200	*SEMA6D*	0.81021133	0.61533098	1.4441E-05	6.7091E-05
ENSMUSG00000039706	*LDB2*	-1.63741267	-1.19389793	1.4566E-05	6.7592E-05
ENSMUSG00000111128		-0.57507634	1.48302032	1.4728E-05	6.8266E-05
ENSMUSG00000078995	*ZFP456*	0.66447289	1.22431796	1.4919E-05	6.9007E-05
ENSMUSG00000022235	*CMBL*	-0.50903745	3.88323544	1.4941E-05	6.907E-05
ENSMUSG00000117879		-1.43026331	-1.45832974	1.5307E-05	7.0616E-05
ENSMUSG00000032350	*GCLC*	-0.61044939	10.1400793	1.5308E-05	7.0616E-05
ENSMUSG00000035351	*NUP37*	0.43550876	2.58331909	1.5606E-05	7.1805E-05
ENSMUSG00000056643	*CHST13*	0.59909635	1.5506255	1.5609E-05	7.1805E-05
ENSMUSG00000050440	*HAMP*	-0.94155905	2.69310494	1.5913E-05	7.3088E-05
ENSMUSG00000079083	*JRKL*	-0.4253066	1.9430392	1.5987E-05	7.3339E-05
ENSMUSG00000011148	*ADSSL1*	-0.50406025	2.24268331	1.6188E-05	7.415E-05
ENSMUSG00000079317	*TRAPPC2*	0.41240735	2.38946574	1.6189E-05	7.415E-05
ENSMUSG00000069270	*H2AC6*	1.83900637	-1.19605825	1.6418E-05	7.5101E-05
ENSMUSG00000034435	*TMEM30B*	-0.41673939	3.0093212	1.664E-05	7.6035E-05
ENSMUSG00000039167	*ADGRL4*	-0.59311412	1.70084185	1.674E-05	7.6432E-05
ENSMUSG00000084819	*GM11967*	-1.08741509	-0.96234824	1.6766E-05	7.6528E-05
ENSMUSG00000100235		-0.57814081	2.05681955	1.6909E-05	7.7132E-05
ENSMUSG00000027353	*MCM8*	-0.5343586	1.77592121	1.6998E-05	7.7516E-05
ENSMUSG00000038930	*RCCD1*	-0.4106563	2.66643269	1.7117E-05	7.7987E-05
ENSMUSG00000005233	*SPC25*	-0.68262223	1.28290659	1.7399E-05	7.9197E-05
ENSMUSG00000031982	*ARV1*	-0.49811282	1.66430666	1.8027E-05	8.1878E-05
ENSMUSG00000011267	*ZFP296*	0.73291562	0.59735853	1.8076E-05	8.2051E-05
ENSMUSG00000045312	*LHFPL2*	0.82104017	3.32156572	1.8116E-05	8.2205E-05
ENSMUSG00000100798	*GM19589*	-0.8339834	1.32595646	1.836E-05	8.3235E-05

ENSMUSG00000024855	*PACS1*	-0.45127953	2.66861137	1.8736E-05	8.4887E-05
ENSMUSG00000021831	*ERO1A*	0.50695032	6.84336467	1.8788E-05	8.5068E-05
ENSMUSG00000039747	*ORAI2*	1.45405101	-0.81318697	1.8802E-05	8.5107E-05
ENSMUSG00000040350	*TRIM7*	0.38389085	5.72190607	1.8854E-05	8.5288E-05
ENSMUSG00000030257	*SRGAP3*	-0.71812153	1.07069876	1.8992E-05	8.5808E-05
ENSMUSG00000043510	*HSCB*	-0.48731017	2.54411836	1.9714E-05	8.8879E-05
ENSMUSG00000023367	*TMEM176A*	0.40980294	7.79048648	2.0102E-05	9.0518E-05
ENSMUSG00000001156	*MXD1*	-0.57947585	2.84155782	2.0622E-05	9.2717E-05
ENSMUSG00000027358	*BMP2*	-0.62738619	1.22917674	2.0838E-05	9.3602E-05
ENSMUSG00000042724	*MAP3K9*	-1.46624436	-1.26421647	2.1026E-05	9.4333E-05
ENSMUSG00000042109	*CSDC2*	-0.70649811	0.82095119	2.1255E-05	9.5212E-05
ENSMUSG00000090942	*F830016B08RIK*	-1.05265987	-0.25863164	2.14E-05	9.5774E-05
ENSMUSG00000029392	*RILPL1*	-0.46569707	1.71043524	2.1426E-05	9.5863E-05
ENSMUSG00000075225	*CCDC162*	-0.61663694	1.83390154	2.186E-05	9.7595E-05
ENSMUSG00000078817	*NLRP12*	-1.1283166	2.28011685	2.2019E-05	9.8184E-05
ENSMUSG00000005148	*KLF5*	0.63190035	2.11561453	2.3237E-05	0.00010324
ENSMUSG00000041351	*RAP1GAP*	-0.46488227	2.74323457	2.3316E-05	0.00010353
ENSMUSG00000034429	*ZFP707*	-0.38395259	3.26580255	2.4123E-05	0.00010674
ENSMUSG00000037455	*SLC18B1*	0.5853524	1.5961252	2.4247E-05	0.00010724
ENSMUSG00000023176	*CPN2*	-0.47013272	4.39973916	2.4402E-05	0.00010786
ENSMUSG00000035686	*THRSP*	-0.55603923	3.09012877	2.4566E-05	0.00010849
ENSMUSG00000066442	*MTHFS*	-0.43369425	2.42962292	2.468E-05	0.00010896
ENSMUSG00000055707	*KLHL26*	-0.46946544	3.14784427	2.4748E-05	0.00010923
ENSMUSG00000022220	*ADCY4*	1.10150896	-0.3652906	2.4866E-05	0.00010972
ENSMUSG00000091387	*GCNT4*	-0.66075828	1.88923027	2.5238E-05	0.00011129
ENSMUSG00000015533	*ITGA2*	0.74858815	2.28494788	2.5329E-05	0.00011166
ENSMUSG00000021379	*ID4*	-1.35646195	-0.97028601	2.5647E-05	0.00011292
ENSMUSG00000016028	*CELSR1*	-0.89678035	-0.29756049	2.5739E-05	0.00011323
ENSMUSG00000048538		-0.51845924	3.58811652	2.5987E-05	0.00011425
ENSMUSG00000036995	*ASAP3*	-0.44258223	3.20043305	2.6633E-05	0.00011663

ENSMUSG00000026646	*SUV39H2*	0.60194362	1.36450025	2.6914E-05	0.00011772
ENSMUSG00000106019		-1.15014865	0.12400796	2.7063E-05	0.00011831
ENSMUSG00000106978		-0.80018396	0.44883642	2.7485E-05	0.00012008
ENSMUSG00000118366		-0.47246291	2.46873962	2.7743E-05	0.00012099
ENSMUSG00000098747	*GM27216*	-0.63254746	1.46856593	2.7859E-05	0.00012146
ENSMUSG00000085733		-0.77570182	0.25874691	2.7906E-05	0.00012162
ENSMUSG00000024778	*FAS*	0.40738142	2.85296738	2.8254E-05	0.00012297
ENSMUSG00000019989	*ENPP3*	-0.41691119	2.54272741	2.843E-05	0.00012359
ENSMUSG00000003929	*ZFP81*	-0.41028253	2.09085469	2.8783E-05	0.00012502
ENSMUSG00000030882	*DNHD1*	0.81575339	1.05156758	2.8848E-05	0.00012522
ENSMUSG00000033389	*ARHGAP44*	-0.44352411	2.02628128	3.0095E-05	0.0001299
ENSMUSG00000024065	*EHD3*	-0.90450351	0.01054569	3.0706E-05	0.00013234
ENSMUSG00000029322	*PLAC8*	-1.00513316	-0.61033185	3.1404E-05	0.00013511
ENSMUSG00000031987	*EGLN1*	0.44780588	6.67081824	3.1517E-05	0.00013548
ENSMUSG00000044005	*GLS2*	-0.47731337	5.32467287	3.172E-05	0.00013631
ENSMUSG00000055725	*PAQR3*	0.41623138	2.61771882	3.2227E-05	0.00013837
ENSMUSG00000035373	*CCL7*	0.7045681	1.53938834	3.2304E-05	0.00013866
ENSMUSG00000022218	*TGM1*	-0.67774813	2.9208027	3.5077E-05	0.00014986
ENSMUSG00000074491	*CLEC4G*	-1.69575544	-1.28230114	3.5225E-05	0.00015041
ENSMUSG00000023915	*TNFRSF21*	-0.54222732	2.4763948	3.5975E-05	0.00015326
ENSMUSG00000031827	*COTL1*	0.48460628	1.97308691	3.6561E-05	0.00015547
ENSMUSG00000004500	*ZFP324*	0.43136326	2.74791193	3.6844E-05	0.00015659
ENSMUSG00000027387	*ZC3H8*	0.45083651	2.07003742	3.6885E-05	0.00015672
ENSMUSG00000069662	*MARCKS*	-0.87959537	0.76573836	3.7599E-05	0.00015943
ENSMUSG00000036206	*SH3BP4*	0.42051205	3.3145243	3.7619E-05	0.00015947
ENSMUSG00000022978	*MIS18A*	-0.43703751	1.54432052	3.7861E-05	0.00016035
ENSMUSG00000005057	*SH2B2*	-0.47092628	2.32251189	3.7931E-05	0.00016056
ENSMUSG00000026782	*ABI2*	0.45198768	2.5157703	3.7973E-05	0.00016064
ENSMUSG00000052921	*ARHGEF15*	1.04206321	0.0607574	3.8989E-05	0.00016456
ENSMUSG00000026923	*NOTCH1*	-0.45265442	4.67330563	3.9154E-05	0.00016518

ENSMUSG00000039763	*DNAJC28*	-0.45261727	2.362127	3.9412E-05	0.0001662
ENSMUSG00000020709	*ADAP2*	-0.37918587	3.17767857	4.0487E-05	0.0001704
ENSMUSG00000113769	*5033406O09RIK*	-0.4196681	2.87003462	4.2051E-05	0.00017673
ENSMUSG00000050945	*ZFP438*	-0.41366711	1.68997149	4.2042E-05	0.00017673
ENSMUSG00000019916	*P4HA1*	0.45564421	4.18616504	4.2842E-05	0.00017974
ENSMUSG00000068744	*PSRC1*	-0.80904941	0.88055318	4.2991E-05	0.00018031
ENSMUSG00000046269	*USP27X*	-0.67123318	0.6289578	4.3234E-05	0.00018123
ENSMUSG00000040978	*GM11992*	-1.66707001	-1.7257294	4.3303E-05	0.00018141
ENSMUSG00000041842	*FHDC1*	-0.6419607	0.66689045	4.3494E-05	0.00018206
ENSMUSG00000030107	*USP18*	-0.55109996	4.53264072	4.3533E-05	0.00018217
ENSMUSG00000028671	*GALE*	0.38403486	4.71636757	4.3688E-05	0.00018261
ENSMUSG00000108181		-0.77389247	-0.09544909	4.5218E-05	0.00018858
ENSMUSG00000041096	*TSPYL2*	-0.37939184	2.91016045	4.5655E-05	0.00019018
ENSMUSG00000026875	*TRAF1*	2.04901091	-1.35441452	4.5934E-05	0.00019118
ENSMUSG00000022999	*LMBR1L*	-0.38048676	3.9889361	4.597E-05	0.00019128
ENSMUSG00000044927	*H1F10*	-1.21809021	-1.22755824	4.6321E-05	0.00019264
ENSMUSG00000041660	*BBOX1*	-0.52595936	1.85357157	4.6575E-05	0.00019358
ENSMUSG00000090264	*EIF4EBP3*	-0.8544039	5.07217711	4.6973E-05	0.00019501
ENSMUSG00000063445	*NMRAL1*	0.56264462	1.4439375	4.8522E-05	0.00020099
ENSMUSG00000028716	*PDZK1IP1*	-0.92884568	-0.01312479	4.935E-05	0.00020419
ENSMUSG00000037833	*SH2D4B*	1.14434447	-0.61934366	4.945E-05	0.00020454
ENSMUSG00000092232		-0.62802456	2.21746346	4.9511E-05	0.00020468
ENSMUSG00000073062	*ZXDB*	-0.54029265	2.0350537	5.0978E-05	0.00021045
ENSMUSG00000092051		-0.95964477	-0.17702697	5.107E-05	0.00021071
ENSMUSG00000115100		-0.58827599	1.70159703	5.1756E-05	0.0002133
ENSMUSG00000041313	*SLC7A1*	0.50935041	4.58987424	5.3356E-05	0.00021958
ENSMUSG00000048924	*CCDC125*	0.39258783	2.69262544	5.4414E-05	0.00022359
ENSMUSG00000041491	*CEP78*	-0.38081102	2.36729711	5.4463E-05	0.00022364
ENSMUSG00000021579	*LRRC14B*	-0.56420797	4.00714825	5.4829E-05	0.00022502
ENSMUSG00000004105	*ANGPTL2*	-0.97027753	0.17003498	5.5229E-05	0.00022634

ENSMUSG00000022837	*IQCB1*	-0.45671864	1.74814	5.641E-05	0.00023073
ENSMUSG00000036136	*FAM110C*	-1.20231347	-0.50535774	5.6477E-05	0.00023094
ENSMUSG00000028453	*FANCG*	-0.59518218	1.52163181	5.6704E-05	0.00023181
ENSMUSG00000090210	*ITGA10*	0.63919591	0.92139071	5.6743E-05	0.0002319
ENSMUSG00000044903	*PSG22*	-0.68053536	1.41633452	5.7046E-05	0.00023285
ENSMUSG00000033082	*CLEC1A*	-2.1241512	-1.41220814	5.7369E-05	0.00023407
ENSMUSG00000027947	*IL6RA*	0.41643361	5.37367947	5.8548E-05	0.00023855
ENSMUSG00000066829	*ZFP810*	-0.55898469	1.18357771	5.9321E-05	0.00024149
ENSMUSG00000045382	*CXCR4*	-0.8360127	0.52787067	5.972E-05	0.00024298
ENSMUSG00000043155	*HPDL*	0.38729043	2.65580057	5.9957E-05	0.00024381
ENSMUSG00000030554	*SYNM*	-1.05564529	-0.29826278	6.0238E-05	0.00024482
ENSMUSG00000027322	*SIGLEC1*	-1.92636364	-1.51513852	6.0397E-05	0.00024526
ENSMUSG00000045251	*ZFP688*	-0.38534993	2.39949913	6.2164E-05	0.0002516
ENSMUSG00000099681		0.44581622	2.04883362	6.2452E-05	0.00025263
ENSMUSG00000038042	*PTPDC1*	-0.49031911	1.94015791	6.2678E-05	0.00025331
ENSMUSG00000074971	*FIBIN*	1.42564891	0.12081298	6.3555E-05	0.0002566
ENSMUSG00000003545	*FOSB*	-1.26457695	0.97052592	6.5317E-05	0.0002632
ENSMUSG00000071176	*ARHGEF10*	0.57129404	3.06556356	6.5623E-05	0.00026437
ENSMUSG00000001622	*CSN3*	0.6490237	4.22027402	6.7382E-05	0.000271
ENSMUSG00000029380	*CXCL1*	0.70051227	4.26771091	6.7971E-05	0.000273
ENSMUSG00000024376	*EPB41L4A*	-0.79163383	0.15375323	6.8111E-05	0.00027349
ENSMUSG00000037979	*CCDC92*	0.94810761	0.28951425	6.8849E-05	0.00027607
ENSMUSG00000103715		-0.86591541	-0.0213282	6.9044E-05	0.0002767
ENSMUSG00000030244	*GYS2*	-0.43496502	2.5479704	7.1007E-05	0.0002838
ENSMUSG00000095315	*GM10130*	0.44937466	1.60326653	7.2617E-05	0.00028968
ENSMUSG00000104814		-0.76324994	0.67787945	7.2907E-05	0.00029076
ENSMUSG00000034110	*KCTD7*	0.61828096	0.96899963	7.2927E-05	0.00029076
ENSMUSG00000028037	*IFI44*	-0.40425822	4.25936355	7.314E-05	0.00029145
ENSMUSG00000039005	*TLR4*	0.72941062	0.5225387	7.3433E-05	0.00029238
ENSMUSG00000040841	*SIX5*	-0.82472313	0.43421773	7.3882E-05	0.00029385

ENSMUSG00000049044	*RAPGEF4*	-0.6541089	5.89238084	7.4272E-05	0.00029524
ENSMUSG00000031382	*ASB11*	-0.70097698	0.33259267	7.433E-05	0.00029539
ENSMUSG00000052658	*5830454E08RIK*	-1.51196249	-1.2866085	7.4608E-05	0.0002962
ENSMUSG00000042842	*SERPINB6B*	-0.76052164	2.01120467	7.4687E-05	0.0002964
ENSMUSG00000035713	*USP35*	-0.42092704	2.23660891	7.6609E-05	0.00030338
ENSMUSG00000021336	*SLC17A4*	-0.51836899	2.23945087	7.6951E-05	0.00030457
ENSMUSG00000043542	*ZC2HC1A*	-0.54976488	1.40567271	7.7963E-05	0.00030808
ENSMUSG00000029468	*P2RX7*	-0.6193974	0.65211363	7.8368E-05	0.00030943
ENSMUSG00000115431		-0.86470483	0.96571102	7.9216E-05	0.00031244
ENSMUSG00000048677	*TPCN2*	-0.38459315	2.63386135	8.2051E-05	0.00032284
ENSMUSG00000032135	*MCAM*	1.18606319	-0.16185147	8.2731E-05	0.00032525
ENSMUSG00000034906	*NCAPH*	-0.55396145	1.03601348	8.2889E-05	0.0003257
ENSMUSG00000083396	*GM15542*	0.49422999	3.77752557	8.3102E-05	0.00032645
ENSMUSG00000020905	*USP43*	0.53894212	3.92725318	8.3222E-05	0.00032684
ENSMUSG00000085445		-0.95784786	-0.47796876	8.4602E-05	0.00033199
ENSMUSG00000057835	*ZFP119A*	-0.47348326	1.32037941	8.4811E-05	0.00033263
ENSMUSG00000097577	*6230400D17RIK*	0.69663697	0.76429585	8.5437E-05	0.00033483
ENSMUSG00000073434	*WDR90*	-0.46482966	2.20456604	8.5668E-05	0.00033554
ENSMUSG00000031480	*THSD1*	-0.40455129	2.53683206	8.7492E-05	0.00034205
ENSMUSG00000014776	*NOL3*	-0.41658189	1.95087304	8.8589E-05	0.00034615
ENSMUSG00000052117	*D630039A03RIK*	1.02868986	-0.67654808	9.0426E-05	0.00035267
ENSMUSG00000067653	*ANKRD23*	0.55763478	3.90397467	9.054E-05	0.00035303
ENSMUSG00000057596	*TRIM30D*	-0.51565101	3.17090846	9.097E-05	0.00035451
ENSMUSG00000028933	*XRCC2*	-0.4284055	1.88499689	9.3667E-05	0.00036435
ENSMUSG00000038115	*ANO2*	-1.5200818	-0.79123989	9.3804E-05	0.00036478
ENSMUSG00000022494	*SHISA9*	-1.11190921	-0.87826028	9.4014E-05	0.0003655
ENSMUSG00000039629	*STRIP2*	-1.98145578	-2.04884414	9.4306E-05	0.00036644
ENSMUSG00000052595	*A1CF*	-0.40867788	3.64105132	9.5288E-05	0.00036967
ENSMUSG00000021835	*BMP4*	-0.69189912	0.82453142	9.5348E-05	0.00036981
ENSMUSG00000024818	*SLC25A45*	0.42827153	3.36647084	9.5816E-05	0.00037133

ENSMUSG00000031434	*MORC4*	-0.52129761	3.68303293	9.6223E-05	0.00037281
ENSMUSG00000029153	*OCIAD2*	-0.42267801	1.52085893	9.7223E-05	0.00037619
ENSMUSG00000067872	*CCDC87*	-1.08391938	-0.88921669	9.8936E-05	0.00038221
ENSMUSG00000038178	*SLC43A2*	-0.60339236	3.63019518	0.00010001	0.00038616
ENSMUSG00000026712	*MRC1*	-0.80046142	0.21232083	0.00010242	0.00039452
ENSMUSG00000003534	*DDR1*	-0.40468112	4.98690841	0.00010254	0.00039488
ENSMUSG00000022836	*MYLK*	-0.42614606	3.3736613	0.00010275	0.0003955
ENSMUSG00000041679	*FBXL9*	-0.51511562	1.07240091	0.00010316	0.00039687
ENSMUSG00000041782	*LAD1*	-0.55448009	2.21800174	0.00010333	0.0003974
ENSMUSG00000025001	*HELLS*	-0.4475375	1.992103	0.0001045	0.00040168
ENSMUSG00000001768	*RIN2*	-0.45483552	2.34307751	0.000105	0.00040321
ENSMUSG00000001768	*BC039771*	-0.45483552	2.34307751	0.000105	0.00040321
ENSMUSG00000101397	*MUG-PS1*	-0.41594047	3.60723171	0.00010787	0.00041345
ENSMUSG00000078922	*TGTP1*	-0.83496524	0.8188847	0.0001082	0.0004145
ENSMUSG00000029174	*TBC1D1*	0.41907569	3.83556464	0.00010823	0.00041453
ENSMUSG00000043789	*VWCE*	-0.4613663	2.08341339	0.00010827	0.00041455
ENSMUSG00000046186	*CD109*	-1.38972924	-0.29066796	0.00011021	0.00042142
ENSMUSG00000056124	*B4GALT6*	-0.55712534	3.02271378	0.00011237	0.00042903
ENSMUSG00000070461		0.78582516	0.48240006	0.00011527	0.00043963
ENSMUSG00000096971	*4930556M19RIK*	-0.4568577	1.56835548	0.00011701	0.00044572
ENSMUSG00000069678	*PCGF1*	-0.41490527	1.82490861	0.00011735	0.00044666
ENSMUSG00000117771	*GM25432*	1.02950003	0.25820923	0.00011767	0.00044776
ENSMUSG00000095193	*GM20939*	-0.4385119	1.12672748	0.000118	0.00044865
ENSMUSG00000006784	*ODAD4*	-1.53731548	-1.65446441	0.0001181	0.00044891
ENSMUSG00000020424	*CASTOR1*	-0.47475366	1.61948516	0.00011942	0.00045323
ENSMUSG00000090877	*HSPA1B*	0.52923671	4.31264257	0.00011976	0.00045438
ENSMUSG00000044595	*DND1*	1.19405784	-0.75384783	0.00012219	0.00046269
ENSMUSG00000022899	*SLC15A2*	-0.61410925	0.66385807	0.00012227	0.00046275
ENSMUSG00000028041	*ADAM15*	0.80182096	0.97843199	0.00012341	0.00046681
ENSMUSG00000034949	*ZFR2*	-0.8145135	0.01759325	0.0001259	0.00047548

ENSMUSG00000072915	*GM12258*	0.58649259	0.61348351	0.00012593	0.00047548
ENSMUSG00000000958	*SLC7A7*	0.45867762	2.45364282	0.00012878	0.00048539
ENSMUSG00000045328	*CENPE*	-1.27974749	-1.28383725	0.00013012	0.00048978
ENSMUSG00000031145	*PRICKLE3*	-0.43185583	2.18757971	0.00013208	0.00049666
ENSMUSG00000053604	*RPIA*	0.38550913	3.2190175	0.0001323	0.00049725
ENSMUSG00000046546	*FAM43A*	0.49583464	2.16941788	0.00013294	0.00049938
ENSMUSG00000087693		-1.00087839	-0.70325989	0.0001353	0.00050758
ENSMUSG00000031024	*DENND2B*	0.440701	4.68898604	0.00013802	0.00051728
ENSMUSG00000037617	*SPAG1*	0.84050572	0.60701745	0.00013948	0.00052209
ENSMUSG00000050192	*EIF5A2*	-0.67498542	0.23362328	0.00014375	0.00053697
ENSMUSG00000085995	*GM2788*	-1.62720037	-1.62488156	0.0001451	0.00054145
ENSMUSG00000026483	*NIBAN1*	0.73287153	4.4131751	0.00014935	0.0005559
ENSMUSG00000033762	*RECQL4*	0.56024907	1.67169446	0.0001506	0.00056001
ENSMUSG00000028532	*CACHD1*	0.4467859	6.03215859	0.00015221	0.00056556
ENSMUSG00000035459	*STAB2*	-0.38041656	3.22771857	0.00015238	0.00056603
ENSMUSG00000107516		1.39450838	-0.51888806	0.00015311	0.00056845
ENSMUSG00000033900	*MAP9*	-0.46332486	2.18885325	0.00015449	0.00057271
ENSMUSG00000019189	*RNF145*	0.40365217	3.78670275	0.00015482	0.00057366
ENSMUSG00000020728	*CEP112*	-1.22612491	-1.06392277	0.00015936	0.00058899
ENSMUSG00000006542	*PRKAG3*	0.84142654	0.22234806	0.00016353	0.00060348
ENSMUSG00000048965	*MRGPRE*	-0.44071832	1.89664205	0.00016664	0.00061372
ENSMUSG00000027737	*SLC7A11*	0.50375156	7.17285655	0.00016944	0.00062278
ENSMUSG00000031255	*SYTL4*	-0.78971709	0.35147408	0.00017011	0.00062493
ENSMUSG00000036687	*TMEM184A*	-0.38093422	2.08846203	0.00017737	0.00064919
ENSMUSG00000029314	*GPAT3*	0.41685316	5.93214442	0.00017861	0.00065357
ENSMUSG00000116673	*A630089N07RIK*	0.55123345	0.96375467	0.00018011	0.00065884
ENSMUSG00000089809	*A930011G23RIK*	-0.74470852	3.81528823	0.00018423	0.00067246
ENSMUSG00000089809	*RASGEF1B*	-0.74470852	3.81528823	0.00018423	0.00067246
ENSMUSG00000020407	*UPP1*	0.54128418	3.65090511	0.00018435	0.0006726
ENSMUSG00000033685	*UCP2*	-0.66656952	1.35817033	0.00018572	0.00067703

ENSMUSG00000028527	*AK4*	0.53465436	6.570687	0.00018775	0.00068324
ENSMUSG00000075256	*CERKL*	-1.35594477	-1.30449258	0.00018786	0.00068349
ENSMUSG00000056602	*FRY*	1.09123409	-0.22589796	0.00019082	0.00069322
ENSMUSG00000022797	*TFRC*	-0.53085392	5.37642776	0.0001915	0.00069536
ENSMUSG00000029512	*ULK1*	-0.44715651	5.98966891	0.00019191	0.00069633
ENSMUSG00000038550	*CIART*	1.00569044	1.95233861	0.0001919	0.00069633
ENSMUSG00000068699	*FLNC*	-0.83984213	1.79047096	0.00019502	0.00070559
ENSMUSG00000098004		1.16223901	-0.27223379	0.00019504	0.00070559
ENSMUSG00000057219	*ARMC7*	0.42364839	2.13115758	0.00019825	0.00071631
ENSMUSG00000041445	*MMRN2*	0.54704295	1.42115289	0.00019993	0.00072219
ENSMUSG00000036882	*ARHGAP33*	1.47552703	-0.97416357	0.00020158	0.00072747
ENSMUSG00000034463	*SCARA3*	-1.72437049	-2.08258851	0.00020176	0.00072792
ENSMUSG00000000184	*CCND2*	0.73770873	1.10244371	0.00020287	0.00073138
ENSMUSG00000086727	*4931428L18RIK*	-1.18019721	-1.05817287	0.00020354	0.00073327
ENSMUSG00000086727	*GM29669*	-1.18019721	-1.05817287	0.00020354	0.00073327
ENSMUSG00000109408	*A930037H05RIK*	-1.31087958	-1.52817923	0.0002058	0.00074048
ENSMUSG00000116657		-0.40780164	3.51566713	0.00020711	0.00074483
ENSMUSG00000028698	*PIK3R3*	1.02442857	-0.27640244	0.00021189	0.00076075
ENSMUSG00000097222	*GATA6OS*	-0.72856324	0.37130448	0.00021306	0.00076446
ENSMUSG00000026017	*CARF*	-0.48816154	1.6110085	0.00021416	0.00076795
ENSMUSG00000102353		-0.38406633	1.72475456	0.00021453	0.00076908
ENSMUSG00000061482	*H4C4*	1.77877066	-1.38814574	0.00021541	0.00077075
ENSMUSG00000042404	*DENND4B*	-0.37988772	1.67492719	0.00021677	0.00077541
ENSMUSG00000067430	*ZFP763*	-0.57228421	0.58142739	0.00021779	0.00077887
ENSMUSG00000053461	*HHIPL2*	-0.55862544	1.59977974	0.00022746	0.0008095
ENSMUSG00000039236	*ISG20*	0.40926689	2.49461392	0.00022738	0.0008095
ENSMUSG00000059824	*DBP*	-0.81517889	1.413695	0.00023652	0.00083949
ENSMUSG00000068877	*SELENBP2*	-0.49097627	6.63267777	0.00023804	0.00084449
ENSMUSG00000023908	*PKMYT1*	-0.38595013	2.40405587	0.00024022	0.00085078
ENSMUSG00000015854	*CD5L*	-1.36054714	0.9058553	0.00024657	0.00087099

ENSMUSG00000051124	*GIMAP9*	0.43318791	2.35019253	0.00025147	0.00088658
ENSMUSG00000022340	*SYBU*	1.20745658	-0.30715105	0.00025229	0.00088904
ENSMUSG00000117485	*GM19696*	-1.01057713	0.10072506	0.00025349	0.00089275
ENSMUSG00000029228	*LNX1*	-0.92438948	-0.04855259	0.00025367	0.00089281
ENSMUSG00000006517	*MVD*	-0.38448209	3.09594616	0.00025821	0.00090685
ENSMUSG00000026447	*PIK3C2B*	-0.59981696	0.96398484	0.00025933	0.00091034
ENSMUSG00000117725		-0.67891838	0.17783562	0.00026334	0.00092353
ENSMUSG00000086390	*1810019D21RIK*	-0.53129513	2.13335283	0.00026504	0.00092885
ENSMUSG00000110781		-0.91384001	-0.24126685	0.00026567	0.00093061
ENSMUSG00000010651	*ACAA1B*	-0.44516782	6.53862833	0.00026616	0.0009321
ENSMUSG00000099146	*0610031O16RIK*	-0.4278148	1.59485749	0.0002663	0.00093236
ENSMUSG00000071633	*GM4952*	-1.1472723	-0.54903311	0.00026921	0.00094167
ENSMUSG00000100277	*1810053B23RIK*	-1.88662713	-0.47520581	0.00026996	0.00094404
ENSMUSG00000024987	*CYP26A1*	0.64264904	3.99628426	0.00027247	0.00095238
ENSMUSG00000030309	*CAPRIN2*	-0.42404352	1.6594532	0.00027256	0.00095246
ENSMUSG00000068758	*IL3RA*	-0.43746245	3.24302026	0.00027277	0.00095296
ENSMUSG00000103039		-0.99627603	-1.0362599	0.00027364	0.00095553
ENSMUSG00000021120	*PIGH*	0.41051305	2.67910605	0.00027518	0.00096004
ENSMUSG00000059060	*RAD51B*	-0.77949298	0.06832124	0.00027541	0.00096058
ENSMUSG00000034303	*CCDC15*	-0.91374995	0.06268438	0.00028155	0.00098061
ENSMUSG00000047821	*TRIM16*	1.68724388	-1.46013973	0.00028246	0.00098356
ENSMUSG00000031425	*PLP1*	-0.7253962	-0.18066117	0.00028693	0.00099768
ENSMUSG00000051113	*GARIN5A*	-0.83869023	-0.60148042	0.00029704	0.00102921
ENSMUSG00000074243		-1.08867146	-0.49557175	0.00029879	0.00103479
ENSMUSG00000019831	*WASF1*	0.39249481	2.19320368	0.00029948	0.00103642
ENSMUSG00000047492	*INHBE*	0.46872443	3.41344303	0.00030187	0.00104419
ENSMUSG00000040093	*BMF*	-0.83520029	0.7955893	0.00030542	0.00105401
ENSMUSG00000026121	*SEMA4C*	0.70701313	1.05498082	0.00030642	0.00105719
ENSMUSG00000046607	*HRK*	-0.95931895	-1.06051515	0.00030775	0.0010613
ENSMUSG00000051177	*PLCB1*	-0.39505224	2.22815188	0.00030801	0.00106194

ENSMUSG00000037544	*DLGAP5*	-1.57676209	-1.44295759	0.00031815	0.0010951
ENSMUSG00000044949	*UBTD2*	0.51805409	1.97226052	0.00032123	0.00110492
ENSMUSG00000081490		1.92448865	-1.59772633	0.00032212	0.00110774
ENSMUSG00000064356	*ATP8*	-0.42778427	9.1108349	0.0003228	0.00110981
ENSMUSG00000043518	*RAI2*	-0.62458674	2.07052869	0.00032337	0.00111124
ENSMUSG00000072964	*BHLHB9*	-1.46748867	-1.69738683	0.00033426	0.00114601
ENSMUSG00000070436	*SERPINH1*	0.73261017	1.24601754	0.00033757	0.00115619
ENSMUSG00000016356	*COL20A1*	0.94109384	0.48512674	0.00033771	0.00115619
ENSMUSG00000053113	*SOCS3*	-0.6970966	0.8555483	0.0003385	0.00115837
ENSMUSG00000029032	*ARHGEF16*	-0.43515593	1.73043694	0.00034027	0.00116388
ENSMUSG00000083863		-0.40401747	3.33898163	0.00034362	0.00117371
ENSMUSG00000000093	*TBX2*	-1.03607858	-0.79182714	0.00034659	0.0011822
ENSMUSG00000080316	*SPACA6*	-0.50718319	1.91534751	0.00034692	0.00118304
ENSMUSG00000086782	*E130102H24RIK*	-0.78322363	0.14758497	0.00034748	0.00118469
ENSMUSG00000073787	*GM10575*	-0.79743841	-0.07015939	0.00035238	0.00119943
ENSMUSG00000039903	*EVA1C*	-0.9383272	0.4759645	0.00035533	0.00120865
ENSMUSG00000028838	*EXTL1*	0.40589486	3.13376257	0.00035606	0.00121085
ENSMUSG00000002459	*RGS20*	-0.60172058	0.99363156	0.00035822	0.00121762
ENSMUSG00000117231	*GM41609*	-1.30643485	-1.56548391	0.00036248	0.00123125
ENSMUSG00000050493	*FAM167B*	-1.19193748	-0.93807223	0.0003648	0.00123711
ENSMUSG00000108218		-0.48454657	1.14691843	0.00036492	0.00123722
ENSMUSG00000038260	*TRPM4*	-1.0521824	-0.57782244	0.00036805	0.00124729
ENSMUSG00000118087	*4833438C02RIK*	0.65437742	0.67511508	0.00037367	0.00126515
ENSMUSG00000042515	*PWWP3B*	-0.71321068	0.7745135	0.000378	0.00127803
ENSMUSG00000020453	*PATZ1*	-0.50290712	2.05491227	0.00037936	0.00128165
ENSMUSG00000115867		0.62571592	1.03083971	0.00037942	0.00128165
ENSMUSG00000027778	*IFT80*	0.41050378	1.78117081	0.00038423	0.00129641
ENSMUSG00000053007	*CREB5*	-0.46188847	4.7656824	0.00038586	0.00130133
ENSMUSG00000026833	*OLFM1*	-1.26583316	-0.72489938	0.0003865	0.00130287
ENSMUSG00000118107		-0.79644545	0.12043372	0.00038918	0.00131041

ENSMUSG00000021265	*SLC25A29*	0.84252209	0.09212406	0.000391	0.00131595
ENSMUSG00000002058	*UNC119*	-0.4958727	1.50331214	0.00039188	0.00131795
ENSMUSG00000085355	*3010003L21RIK*	0.40852873	1.70682247	0.00040106	0.00134579
ENSMUSG00000035105	*EGLN3*	1.05118035	5.76943074	0.00040622	0.00136154
ENSMUSG00000051855	*MEST*	0.86569233	0.6854124	0.00040647	0.00136207
ENSMUSG00000045672	*COL27A1*	-0.60093436	2.75827127	0.00040726	0.00136411
ENSMUSG00000041594	*TMTC4*	-0.50810775	1.0223349	0.00041365	0.00138423
ENSMUSG00000042797	*AQP11*	-0.38587091	2.47398733	0.00041422	0.00138582
ENSMUSG00000063415	*CYP26B1*	-0.51881905	2.22643291	0.00042078	0.00140651
ENSMUSG00000061046	*HAGHL*	-0.40313412	2.25488523	0.00042169	0.00140921
ENSMUSG00000030046	*BMP10*	-1.86535528	-1.83085187	0.0004264	0.00142301
ENSMUSG00000028480	*GLIPR2*	0.93200375	1.25945055	0.00042834	0.00142885
ENSMUSG00000092035	*PEG10*	0.56071948	2.51027776	0.00042874	0.00142985
ENSMUSG00000016552	*FOXRED2*	0.55833451	1.17296915	0.00043214	0.0014402
ENSMUSG00000031755	*BBS2*	-0.54997226	1.12212482	0.00043456	0.00144729
ENSMUSG00000059588	*CALCRL*	-0.50066697	1.26264861	0.00043589	0.00145072
ENSMUSG00000042333	*TNFRSF14*	-0.47264439	1.40112363	0.00044005	0.00146325
ENSMUSG00000100680	*1810044D09RIK*	-0.58261033	0.723723	0.00044153	0.00146717
ENSMUSG00000022221	*RIPK3*	1.39903489	-1.1625145	0.00044605	0.00148018
ENSMUSG00000041219	*ARHGAP11A*	-0.5322809	1.80410077	0.00046374	0.00153367
ENSMUSG00000111977		-1.2947166	-1.59607157	0.00046782	0.00154583
ENSMUSG00000034771	*TLE2*	-0.6332738	1.2628496	0.00046909	0.0015493
ENSMUSG00000052364	*B630019K06RIK*	-1.16198841	-1.11733212	0.00048033	0.00158109
ENSMUSG00000023066	*RTTN*	-0.49373366	0.54832323	0.00048062	0.00158132
ENSMUSG00000033594	*SPATA2L*	-0.62376117	0.46604047	0.00048455	0.00159318
ENSMUSG00000053198	*PRX*	1.39530538	-1.19384872	0.00048571	0.00159629
ENSMUSG00000038807	*RAP1GAP2*	0.65588242	0.7132759	0.00050381	0.00164913
ENSMUSG00000001520	*NRIP2*	0.69056178	1.82208817	0.00050376	0.00164913
ENSMUSG00000097636		1.29914437	-0.37049608	0.00050372	0.00164913
ENSMUSG00000039813	*TBC1D2*	-0.49872419	1.03064045	0.00050493	0.00165241

ENSMUSG00000111528	*GM39460*	-0.79616477	-0.44416237	0.00051059	0.0016687
ENSMUSG00000040389	*WDR47*	-0.39569976	1.66755809	0.00051701	0.00168707
ENSMUSG00000103041		-0.59614568	0.46589352	0.00052452	0.00170853
ENSMUSG00000020080	*HKDC1*	-0.62107273	1.15466635	0.00052702	0.00171591
ENSMUSG00000004038	*GSTM3*	0.51012202	7.91926492	0.00052813	0.00171914
ENSMUSG00000000686	*ABHD15*	-0.49381236	1.03391395	0.00053103	0.00172745
ENSMUSG00000073424	*CYP4F15*	-0.52173902	1.48595876	0.0005422	0.00176142
ENSMUSG00000041695	*KCNJ2*	-0.95772727	0.24424739	0.00054702	0.00177631
ENSMUSG00000078700	*D030028A08RIK*	0.43314037	1.43625665	0.00057314	0.00185455
ENSMUSG00000057234	*METTL15*	-0.49087889	1.46910092	0.00057506	0.00185996
ENSMUSG00000109841	*E330011O21RIK*	-0.48889981	3.55393946	0.00058102	0.00187758
ENSMUSG00000078435	*AU041133*	0.50710951	1.05453608	0.00058782	0.00189687
ENSMUSG00000002885	*ADGRE5*	0.84274906	0.77651805	0.00058839	0.00189764
ENSMUSG00000097743	*GM16973*	-0.49702941	0.82477618	0.00059989	0.00193131
ENSMUSG00000118038	*GM9895*	-1.44355266	-1.49105459	0.00060719	0.00195279
ENSMUSG00000071192	*WFIKKN1*	-0.95666504	-0.58751982	0.0006145	0.00197274
ENSMUSG00000113262	*GM48551*	0.61019468	1.3386486	0.00062623	0.00200558
ENSMUSG00000052353	*CEMIP*	1.34173964	-1.28726713	0.00063936	0.0020454
ENSMUSG00000059674	*CDH24*	0.64151703	0.45126048	0.0006492	0.00207372
ENSMUSG00000039239	*TGFB2*	0.46661527	3.62638481	0.00065058	0.00207677
ENSMUSG00000109291	*GM2814*	-1.15465853	-1.42685817	0.00065317	0.00208322
ENSMUSG00000039131	*GIPC2*	-0.61193782	1.04323032	0.00066264	0.00211204
ENSMUSG00000027474	*CCM2L*	1.35168297	-1.07088014	0.00066341	0.00211403
ENSMUSG00000078866	*ZFP970*	0.44550835	3.30709241	0.00067709	0.00215437
ENSMUSG00000031539	*AP3M2*	-0.40332642	1.76694198	0.00068467	0.00217423
ENSMUSG00000045106	*CCDC73*	-0.92426374	-1.34260586	0.00068691	0.00218088
ENSMUSG00000087026	*A230103J11RIK*	1.05021559	-0.4269265	0.00068976	0.0021885
ENSMUSG00000069743	*ZFP820*	-0.4390091	1.03131842	0.00069914	0.00221492
ENSMUSG00000027577	*CHRNA4*	-0.41617073	1.60920278	0.00069955	0.00221575
ENSMUSG00000030849	*FGFR2*	-0.470656	2.53191712	0.00070028	0.00221759

ENSMUSG00000026479	*LAMC2*	0.40910542	3.13895369	0.00070331	0.00222621
ENSMUSG00000108738		0.8799386	-0.77391671	0.00071827	0.00227062
ENSMUSG00000074500	*ZFP558*	-0.63292938	-0.18228679	0.00073834	0.00232987
ENSMUSG00000087249	*GM16062*	0.38007173	1.62382566	0.00074059	0.00233617
ENSMUSG00000051817	*SOX12*	-0.51577518	1.26858197	0.00074581	0.00235211
ENSMUSG00000020937	*PLCD3*	-0.56740856	0.08394015	0.00078509	0.00246645
ENSMUSG00000032064	*DIXDC1*	-0.59801531	1.01424634	0.00078654	0.00246997
ENSMUSG00000021234	*FAM161B*	0.87181745	-0.54022711	0.0007864	0.00246997
ENSMUSG00000057037	*CFHR1*	-0.44380167	2.51305919	0.00079413	0.00249272
ENSMUSG00000086938	*4930481A15RIK*	-0.93611762	-0.50247804	0.0008142	0.00254756
ENSMUSG00000038058	*NOD1*	-0.42303747	1.92087609	0.00081687	0.00255484
ENSMUSG00000089810		-0.4229862	1.13755399	0.00081754	0.00255638
ENSMUSG00000048489	*DEPP1*	-0.73718303	6.44423896	0.00082094	0.00256645
ENSMUSG00000039958	*ETFBKMT*	-0.37862164	5.45552918	0.00084716	0.00264619
ENSMUSG00000022579	*GPIHBP1*	-1.15718711	-0.80625785	0.00085646	0.00267239
ENSMUSG00000041180	*HECTD2*	-0.39833299	2.493063	0.00086298	0.0026893
ENSMUSG00000032192	*GNB5*	-0.69319885	0.01220461	0.00087216	0.00271445
ENSMUSG00000027496	*AURKA*	-0.53821489	0.69487802	0.00087302	0.00271657
ENSMUSG00000087222		-0.62745623	0.01490132	0.00088222	0.00274111
ENSMUSG00000021335	*SLC17A1*	-0.58431808	0.33486763	0.00089308	0.00276959
ENSMUSG00000031907	*ZFP90*	-0.47689396	1.42068218	0.00089545	0.00277635
ENSMUSG00000014164	*KLHL3*	0.44364731	1.78198036	0.00089695	0.0027804
ENSMUSG00000034394	*LIF*	-0.65200914	0.77892967	0.00089966	0.00278705
ENSMUSG00000071551	*AKR1C19*	-0.58545463	2.91492149	0.00089957	0.00278705
ENSMUSG00000024421	*LAMA3*	-0.56856742	1.59151128	0.00090418	0.00280045
ENSMUSG00000062861	*ZFP28*	-0.58463769	0.85174326	0.00091081	0.0028198
ENSMUSG00000112276		-0.82622381	-1.04834926	0.00091349	0.00282691
ENSMUSG00000054757	*AKR1C20*	-0.46125779	1.52753431	0.00091569	0.00283311
ENSMUSG00000054659	*PM20D2*	0.43496446	1.58567899	0.00092095	0.0028476
ENSMUSG00000032114	*SLC37A4*	-0.43185267	4.76948204	0.00092175	0.00284947

ENSMUSG00000085095		1.11240141	-0.32090986	0.00092568	0.0028604
ENSMUSG00000039611	*PGAP4*	-0.96126496	-0.80377354	0.00093838	0.00289538
ENSMUSG00000038637	*LRRC56*	-0.49113099	0.92946563	0.00095275	0.0029354
ENSMUSG00000059327	*EDA*	-0.49196836	1.2960273	0.00095959	0.00295588
ENSMUSG00000031214	*OPHN1*	-0.50076799	1.03891677	0.00096098	0.00295951
ENSMUSG00000049555	*TMIE*	-0.96508319	-0.40243652	0.00097596	0.00300063
ENSMUSG00000028003	*LRAT*	-1.30458482	-1.34564296	0.00097663	0.00300172
ENSMUSG00000018727	*CPSF4L*	-1.32375474	-1.05849214	0.00098075	0.00301219
ENSMUSG00000072763	*5430403G16RIK*	-0.71039171	-0.34159766	0.00098154	0.00301399
ENSMUSG00000021281	*TNFAIP2*	0.49890618	2.39605894	0.00100234	0.00307275
ENSMUSG00000091119	*CCDC152*	-0.85949851	-0.1599311	0.00100415	0.00307698
ENSMUSG00000015222	*MAP2*	-1.57676969	-1.38156752	0.00102827	0.00314434
ENSMUSG00000084807		1.31824373	-0.78450144	0.00103012	0.00314934
ENSMUSG00000060923	*ACYP2*	-0.54385538	0.66616728	0.00103754	0.00317071
ENSMUSG00000113749	*MRTO4-PS1*	1.20279489	-1.40192389	0.00105115	0.00321031
ENSMUSG00000056753		-1.0350423	-1.30124212	0.00105418	0.00321787
ENSMUSG00000097006	*9530082P21RIK*	-0.85552643	-0.46495238	0.00105883	0.00322904
ENSMUSG00000026429	*UBE2T*	-0.66667004	-0.03593514	0.00107622	0.00327596
ENSMUSG00000086141	*9030622O22RIK*	-1.02343778	-1.45320206	0.0010823	0.00329298
ENSMUSG00000022148	*FYB*	0.40219969	3.23725602	0.0011168	0.00339036
ENSMUSG00000018238	*GDF9*	0.65010794	0.54349691	0.00113904	0.00344644
ENSMUSG00000040084	*BUB1B*	-0.57855654	0.244604	0.00115123	0.00347762
ENSMUSG00000097048	*1600020E01RIK*	0.52004073	2.30328222	0.00115197	0.00347913
ENSMUSG00000020303	*STC2*	1.96849369	-1.66571244	0.0011569	0.0034933
ENSMUSG00000113831		-0.99468639	-0.94592684	0.00116922	0.00352686
ENSMUSG00000096472	*CDKN2D*	-0.74229774	-0.29316781	0.00117296	0.0035367
ENSMUSG00000056904	*GM5620*	0.41166966	2.99286171	0.00117395	0.00353897
ENSMUSG00000109372	*GM19410*	1.12505905	-0.04423164	0.00117839	0.00355015
ENSMUSG00000087132	*A930001C03RIK*	-0.48060967	1.13265559	0.00119261	0.00358712
ENSMUSG00000062101	*ZFP119B*	-0.479303	1.02427512	0.00119782	0.00359982

ENSMUSG00000103906	*TIGD5*	-0.51626817	1.29243467	0.00120526	0.0036207
ENSMUSG00000117123		0.4472206	1.24165809	0.00121621	0.00364987
ENSMUSG00000020212	*MDM1*	-0.6806714	0.55484397	0.0012221	0.00366291
ENSMUSG00000085091		-0.74724089	-0.07171132	0.00122368	0.0036663
ENSMUSG00000044349	*SNHG11*	-0.62541441	1.36515343	0.00122572	0.0036709
ENSMUSG00000086606		0.56907783	0.4918793	0.00123754	0.0037048
ENSMUSG00000038463	*OLFML2B*	-0.60580977	0.78939031	0.00123852	0.00370699
ENSMUSG00000043644	*0610009L18RIK*	-0.80324369	-0.4801937	0.00125173	0.00374422
ENSMUSG00000017740	*SLC12A5*	-0.87346278	-0.83655129	0.00126861	0.00379239
ENSMUSG00000041540	*SOX5*	-0.5620475	0.99073286	0.00130534	0.00389478
ENSMUSG00000010154	*SPIRE2*	-1.10215373	-0.86128122	0.00130743	0.0038997
ENSMUSG00000014633	*CMC2*	0.45392045	1.72428731	0.00135646	0.00403529
ENSMUSG00000052125	*F730043M19RIK*	2.11403286	-1.11838625	0.0013587	0.00403869
ENSMUSG00000106825		-0.78692272	-0.64272038	0.00136573	0.00405796
ENSMUSG00000047298	*KCNV2*	-1.20920415	-1.26692291	0.00141989	0.00420272
ENSMUSG00000025473	*ADAM8*	0.62467411	2.27965682	0.00142562	0.00421798
ENSMUSG00000035270	*IMPG2*	-0.48683041	1.06947075	0.0014313	0.00423136
ENSMUSG00000074213		-1.12033441	-1.07205364	0.00144321	0.00426316
ENSMUSG00000105368		-0.99685473	-1.21750956	0.0014496	0.00428117
ENSMUSG00000062410	*HSD3B3*	-0.54675932	2.07905223	0.00146781	0.00433059
ENSMUSG00000054863	*TAFA5*	-0.43560903	1.17897489	0.00149143	0.00439409
ENSMUSG00000038541	*SRD5A2*	-0.60324972	0.38220172	0.0015008	0.0044208
ENSMUSG00000030087	*KLF15*	-0.39488296	5.35181973	0.00152033	0.00447205
ENSMUSG00000032060	*CRYAB*	0.52339018	0.95983694	0.00153286	0.0045053
ENSMUSG00000112796		-0.92320371	-0.92178408	0.00154202	0.0045286
ENSMUSG00000090659	*ZFP493*	0.38573379	1.34898986	0.0015513	0.00455131
ENSMUSG00000110156	*GM42067*	0.75342584	0.14648762	0.00155288	0.00455503
ENSMUSG00000054453	*SYTL5*	-0.44220578	1.78959566	0.00156453	0.00458501
ENSMUSG00000039981	*ZC3H12D*	-0.53839208	1.46230757	0.00157078	0.00459834
ENSMUSG00000075031	*H2BC3*	1.64726199	-1.59664614	0.0016093	0.0047008

ENSMUSG00000045466	*ZFP956*	-0.39791182	1.01312792	0.00163035	0.00475851
ENSMUSG00000062309	*RPP25*	1.01424357	-1.23775157	0.00164579	0.0047988
ENSMUSG00000025790	*SLCO3A1*	0.68899463	0.51908752	0.00166949	0.00486211
ENSMUSG00000036964	*TRIM17*	0.98418884	-0.83060008	0.00167362	0.00487221
ENSMUSG00000060244	*ALYREF2*	0.42237014	1.4477121	0.00168101	0.00489178
ENSMUSG00000108378		-0.87382743	-0.91500163	0.00168235	0.00489473
ENSMUSG00000019214	*CHTF18*	-0.70083856	-0.04313851	0.00171203	0.00497022
ENSMUSG00000049804	*ARMCX4*	-1.29926505	-1.30316428	0.0017406	0.0050472
ENSMUSG00000036216	*LEAP2*	-0.58603558	1.05199725	0.00174609	0.00505911
ENSMUSG00000006930	*HAP1*	-1.04189467	-1.11505833	0.00175316	0.00507677
ENSMUSG00000051246	*MSANTD1*	0.96354983	-1.12602046	0.0017622	0.00510075
ENSMUSG00000016496	*CD274*	-0.46439342	1.52560388	0.00176827	0.00511529
ENSMUSG00000033705	*STARD9*	-0.53351817	0.4673264	0.00177276	0.00512527
ENSMUSG00000107005		-0.82402842	-0.84690482	0.00177426	0.0051286
ENSMUSG00000008398	*ELK3*	0.71173293	1.08968337	0.00177797	0.00513495
ENSMUSG00000031478	*NEK3*	-0.40774671	1.65522859	0.00180765	0.00521689
ENSMUSG00000057132	*RPGRIP1*	1.60499675	-1.40580898	0.00181119	0.00522607
ENSMUSG00000116097	*GM36738*	-0.47037145	0.69861874	0.00182662	0.00526646
ENSMUSG00000061451	*TMEM151A*	-1.00788368	-1.09960196	0.00183057	0.00527579
ENSMUSG00000107092		0.7458406	-0.03608062	0.0018328	0.00528116
ENSMUSG00000062310	*GLRP1*	1.64080486	-1.74216321	0.00185196	0.00533324
ENSMUSG00000061601	*PCLO*	-0.65945228	-0.336579	0.0018592	0.00535199
ENSMUSG00000001521	*TULP3*	-0.5835866	0.16428344	0.00186332	0.00536173
ENSMUSG00000074280		-1.23395498	-1.28555942	0.00187306	0.0053866
ENSMUSG00000110251		-1.12833947	-1.74694139	0.00187651	0.0053923
ENSMUSG00000040710	*ST8SIA4*	-1.26078751	-1.02212286	0.00187912	0.00539876
ENSMUSG00000100975		0.62443785	0.6132194	0.00188159	0.00540373
ENSMUSG00000041000	*TRIM62*	-0.44186829	1.14673458	0.00189039	0.00542264
ENSMUSG00000015605	*SRF*	-0.38719202	6.04663309	0.00189032	0.00542264
ENSMUSG00000109147		-0.70134017	-0.45088061	0.00189083	0.00542284

ENSMUSG00000090369	*4933411K16RIK*	-0.97053769	-1.12023543	0.00192064	0.00549546
ENSMUSG00000086296		0.70529651	0.10449187	0.00192827	0.00551513
ENSMUSG00000071722	*SPIN4*	0.73949473	-0.23544142	0.00193463	0.00553011
ENSMUSG00000055866	*PER2*	0.46850845	3.72690883	0.00195283	0.00557886
ENSMUSG00000025202	*SCD3*	-0.57471223	0.55082324	0.00195529	0.0055848
ENSMUSG00000112532	*GM36283*	0.49140033	1.79484184	0.00198053	0.00565251
ENSMUSG00000024030	*ABCG1*	-0.76319676	0.21860237	0.00198169	0.0056547
ENSMUSG00000019942	*CDK1*	-0.69188491	0.15528853	0.00198321	0.00565795
ENSMUSG00000090564	*A430057M04RIK*	0.60086989	0.28218654	0.00199456	0.00568811
ENSMUSG00000030641	*DDIAS*	-0.38144597	3.06748929	0.00199564	0.00569009
ENSMUSG00000039004	*BMP6*	-0.51125083	1.61066486	0.00200283	0.0057095
ENSMUSG00000089889	*0610040B10RIK*	-0.71906285	-0.06011581	0.00203222	0.00578431
ENSMUSG00000087008		0.65875768	0.04051316	0.00204352	0.00581306
ENSMUSG00000085642		-0.7320774	0.03758674	0.00206073	0.0058609
ENSMUSG00000090077	*LIME1*	0.51065794	1.75221907	0.00208601	0.00592873
ENSMUSG00000039217	*IL18*	-0.51644155	0.69804377	0.0020935	0.00594489
ENSMUSG00000035239	*NEU3*	0.52230497	1.23647526	0.00210779	0.00598083
ENSMUSG00000034205	*LOXL2*	0.46627756	4.66919355	0.0021169	0.00600204
ENSMUSG00000040829	*ZMYND15*	0.41213836	1.94428879	0.00213031	0.00603539
ENSMUSG00000117819		-0.39886695	2.22509364	0.00213609	0.00604827
ENSMUSG00000060131	*ATP8B4*	1.14185192	-0.98801177	0.00215492	0.00609336
ENSMUSG00000078773	*RAD54B*	-0.89656349	-0.59493183	0.00216636	0.00612099
ENSMUSG00000027777	*SCHIP1*	0.52587804	0.68268865	0.00221624	0.00624871
ENSMUSG00000087213	*2810408I11RIK*	0.79996356	-0.88755963	0.0022218	0.00626319
ENSMUSG00000108402		-0.73280111	-0.72688431	0.00222965	0.00628291
ENSMUSG00000082938	*GM2810*	-0.42230063	1.96577844	0.00223383	0.00629346
ENSMUSG00000074006	*OMP*	0.95130544	-0.87220664	0.00223602	0.00629722
ENSMUSG00000008496	*POU2F2*	2.45569693	-1.55904753	0.0022472	0.00632507
ENSMUSG00000093507		1.42610388	-2.01534405	0.00225131	0.00633543
ENSMUSG00000023032	*SLC4A8*	-0.8310286	-1.07899373	0.00225738	0.00635006

ENSMUSG00000056771		-0.78579511	-0.89762111	0.00229126	0.00643922
ENSMUSG00000068270	*SHROOM4*	0.68404712	0.6581256	0.00229758	0.00645327
ENSMUSG00000001986	*GRIA3*	-0.62465829	0.43231256	0.00230495	0.00647149
ENSMUSG00000035891	*CERK*	0.44448391	2.42357727	0.00231816	0.00650361
ENSMUSG00000038608	*DOCK10*	-0.88857381	-0.55429096	0.00236526	0.00661805
ENSMUSG00000022099	*DMTN*	-0.9197754	-1.05864002	0.00237037	0.00662854
ENSMUSG00000087365		-1.01443579	-1.33338564	0.00242088	0.0067582
ENSMUSG00000019647	*SEMA6A*	-1.04720127	-0.62811314	0.00244797	0.00682346
ENSMUSG00000082962		-1.45033369	-1.43672996	0.00246304	0.00685766
ENSMUSG00000085379		0.83286404	-0.02081418	0.00246265	0.00685766
ENSMUSG00000020914	*TOP2A*	-0.87993797	-1.23944973	0.00247891	0.0068966
ENSMUSG00000045284	*DCAF12L1*	-0.44744908	1.3743384	0.00248342	0.00690655
ENSMUSG00000043505	*GIMAP5*	1.05917902	-1.11549899	0.00249265	0.00692828
ENSMUSG00000079737	*PPHLN1-PS1*	0.53693139	0.83152062	0.00250014	0.00694647
ENSMUSG00000079737	*BFAR*	0.53693139	0.83152062	0.00250014	0.00694647
ENSMUSG00000041372	*B4GALNT3*	1.57051533	-0.34255517	0.00250384	0.00695281
ENSMUSG00000114117		-0.75505474	-0.7288143	0.00251163	0.00697047
ENSMUSG00000023341	*MX2*	-0.4066116	2.82707209	0.0025247	0.0070041
ENSMUSG00000021365	*NEDD9*	0.58286712	2.7533065	0.00259197	0.00716636
ENSMUSG00000024544	*LDLRAD4*	-0.45821388	1.46555736	0.00259719	0.00717809
ENSMUSG00000038702	*DSEL*	-0.48300303	1.20243801	0.00260774	0.00720453
ENSMUSG00000064294	*AOX3*	-0.41497299	3.37910328	0.00262972	0.00725842
ENSMUSG00000097715		-0.89978846	0.20040049	0.00264307	0.00729117
ENSMUSG00000038092	*HSD3B5*	-0.72426672	1.4998806	0.00268644	0.00740385
ENSMUSG00000011884	*GLTP*	-0.40248382	1.48155049	0.00270987	0.00745443
ENSMUSG00000036480	*PRSS56*	0.59314808	-0.18801882	0.00270952	0.00745443
ENSMUSG00000019987	*ARG1*	-0.5401236	7.0805794	0.00271393	0.00746421
ENSMUSG00000101609	*KCNQ1OT1*	0.59440825	1.02091644	0.00271756	0.00746719
ENSMUSG00000051396	*HSPA14*	0.59380751	0.56738363	0.00275219	0.0075518
ENSMUSG00000051396	*GM45902*	0.59380751	0.56738363	0.00275219	0.0075518

ENSMUSG00000021747	*CFAP20DC*	-0.40737447	1.23905287	0.00281051	0.00770123
ENSMUSG00000078202	*NRARP*	1.02752962	-0.88941824	0.00282465	0.00773828
ENSMUSG00000074794	*ARRDC3*	-0.54226692	4.52124949	0.00288305	0.00787475
ENSMUSG00000109783		-0.61071742	0.37584506	0.00288771	0.00788454
ENSMUSG00000063851	*RNF183*	1.04517991	0.10123852	0.00292703	0.00798301
ENSMUSG00000059277	*R74862*	-0.67092095	0.08341461	0.00301867	0.00821311
ENSMUSG00000086075		-1.04250338	-1.19634286	0.00302811	0.00823573
ENSMUSG00000038415	*FOXQ1*	-0.44290001	3.22563827	0.00309469	0.00839504
ENSMUSG00000000982	*CCL3*	1.7901994	-1.86797384	0.00310193	0.00841315
ENSMUSG00000026358	*RGS1*	-0.8213235	-0.68654892	0.00312295	0.00846078
ENSMUSG00000081232	*GM14373*	-0.81140862	-0.93574287	0.00312264	0.00846078
ENSMUSG00000074024	*4632427E13RIK*	-0.49683681	0.28109008	0.00313293	0.0084831
ENSMUSG00000043004	*GNG2*	-1.35107908	-1.65510183	0.00315017	0.00852037
ENSMUSG00000074903		-0.6252917	0.47309544	0.00317875	0.00858977
ENSMUSG00000039099	*WDR93*	-0.97845271	-1.08312639	0.00325995	0.00879104
ENSMUSG00000107997		-0.81570622	-0.78599318	0.0032762	0.00882711
ENSMUSG00000038151	*PRDM1*	1.19066734	-1.22569596	0.00329776	0.00887867
ENSMUSG00000029762	*AKR1B8*	0.45974662	1.35917681	0.00331702	0.00891745
ENSMUSG00000118559	*A930007A09RIK*	-0.5700141	-0.17763869	0.00339825	0.00911748
ENSMUSG00000026205	*SLC23A3*	1.18283026	-1.26540443	0.00341885	0.00916772
ENSMUSG00000097643		-1.02453402	-1.01019827	0.00342572	0.00918447
ENSMUSG00000031304	*IL2RG*	1.13986655	-0.80442868	0.00345304	0.00924756
ENSMUSG00000033715	*AKR1C14*	-0.3801885	3.51407009	0.00345574	0.00925312
ENSMUSG00000108391		-0.83861467	-0.96275368	0.00347905	0.00930586
ENSMUSG00000047139	*CD24A*	-0.73237207	-0.92193289	0.00353837	0.00945368
ENSMUSG00000107195		-1.07100013	-1.58990668	0.00354364	0.00946603
ENSMUSG00000048731	*GGNBP1*	-0.47246264	1.04454333	0.00354688	0.00947297
ENSMUSG00000026196	*BARD1*	-0.402775	0.90103646	0.0035774	0.00954407
ENSMUSG00000044042	*FMN1*	-0.5625484	0.95368604	0.00358245	0.0095558
ENSMUSG00000085287	*4833418N02RIK*	-0.74545433	-0.49312914	0.00358467	0.00955964

ENSMUSG00000087165	*2010001A14RIK*	-0.53653462	0.12757957	0.00359041	0.00956834
ENSMUSG00000035919	*BBS9*	-0.44648718	1.31522807	0.00360367	0.00960021
ENSMUSG00000019235	*RPS6KL1*	-0.44802476	1.44923018	0.00360571	0.00960279
ENSMUSG00000028687	*MUTYH*	-0.49911253	0.59847126	0.00363571	0.00966803
ENSMUSG00000024905	*TESMIN*	-0.55774637	-0.22735734	0.00366364	0.00973347
ENSMUSG00000079505		-0.38329924	1.27082254	0.00367481	0.00975781
ENSMUSG00000023571	*C1QTNF12*	0.81000329	-0.35745604	0.00370658	0.00983011
ENSMUSG00000074874	*CTLA2B*	-1.01905716	-0.52628459	0.00372152	0.00986404
ENSMUSG00000048498	*CD300E*	2.25221594	-1.84677251	0.00374218	0.00991163
ENSMUSG00000092212	*SLC22A13B*	1.42535388	-1.51149687	0.00374661	0.00991981
ENSMUSG00000029163	*EMILIN1*	-0.50200059	1.38245324	0.00375315	0.00993353
ENSMUSG00000042564	*FAM227A*	-0.83876898	-0.98657615	0.00379106	0.01001762
ENSMUSG00000097673		-1.23692695	-1.15630677	0.00380209	0.01004497
ENSMUSG00000022987	*ZFP641*	-1.12293634	-1.2150136	0.00380689	0.01005222
ENSMUSG00000069793	*SLFN9*	-0.85798711	-0.14418852	0.00381146	0.01005885
ENSMUSG00000071042	*RASGRP3*	0.4245404	0.88978708	0.00382773	0.01009455
ENSMUSG00000087593	*GM16174*	-0.91764729	-1.27276877	0.00387882	0.01021164
ENSMUSG00000086754		0.45813439	0.79450421	0.00388858	0.01023478
ENSMUSG00000069892	*9930111J21RIK2*	-0.79738905	-0.11831736	0.003892	0.01024197
ENSMUSG00000051159	*CITED1*	-0.73732965	-0.78099819	0.00391016	0.01028421
ENSMUSG00000034855	*CXCL10*	-0.99871605	2.49611846	0.0039432	0.01036183
ENSMUSG00000096956	*SNHG18*	-0.73470432	-0.00743765	0.00397674	0.01043876
ENSMUSG00000105353		-0.420936	2.01551744	0.00399635	0.01048836
ENSMUSG00000020701	*TMEM132E*	0.69020203	-0.16749857	0.00401204	0.01052201
ENSMUSG00000097817		-0.85555932	-0.98940885	0.00401975	0.01053659
ENSMUSG00000027715	*CCNA2*	0.95559131	-1.26760244	0.00403652	0.01056923
ENSMUSG00000066361	*SERPINA3C*	0.93268622	-1.00193253	0.0040589	0.01062215
ENSMUSG00000027959	*SASS6*	-0.42292162	1.01321756	0.00406672	0.01063314
ENSMUSG00000028307	*ALDOB*	-0.37949955	7.80442628	0.00406619	0.01063314
ENSMUSG00000025766	*D3ERTD751E*	-0.60258997	0.43842036	0.00409473	0.01069877

ENSMUSG00000081476		0.89105052	-0.60397466	0.00411711	0.01075149
ENSMUSG00000053038		0.41045045	0.83565882	0.00413555	0.01079774
ENSMUSG00000097842	*9330104G04RIK*	-0.6634684	-0.64159598	0.00420265	0.01096124
ENSMUSG00000038473	*NOS1AP*	-0.46341507	1.5525787	0.00420729	0.01096942
ENSMUSG00000091900		0.74099222	0.67891473	0.00422028	0.01099939
ENSMUSG00000097080	*1700086O06RIK*	0.4755728	0.76529271	0.00423793	0.01103857
ENSMUSG00000030222	*RERG*	-1.29053181	-1.75728256	0.00424564	0.01105177
ENSMUSG00000049985	*ANKRD55*	-0.47414138	1.81823171	0.00428379	0.01114315
ENSMUSG00000042787	*EXOG*	0.43692934	1.38662022	0.0043292	0.01124535
ENSMUSG00000017146	*BRCA1*	-0.51226787	0.86167045	0.00433159	0.01124956
ENSMUSG00000082820		-0.9774679	-0.70805624	0.00433769	0.01126342
ENSMUSG00000097313		-0.4674885	0.63672503	0.004346	0.01128301
ENSMUSG00000063388		-1.04855468	-1.31971314	0.00436957	0.01133018
ENSMUSG00000079553	*KIFC1*	-0.5181102	1.06509129	0.00440563	0.01141359
ENSMUSG00000032294	*PKM*	0.63072034	6.86876041	0.00442622	0.01146492
ENSMUSG00000006720	*ZFP184*	-0.77056866	-0.90858726	0.00448006	0.01159414
ENSMUSG00000081111	*GM5913*	0.43159725	1.20491965	0.00449185	0.01161647
ENSMUSG00000037157	*IL22RA1*	0.44389113	4.13364266	0.00455259	0.01175906
ENSMUSG00000022440	*C1QTNF6*	-0.45891539	2.21433962	0.00460895	0.01190044
ENSMUSG00000009108	*GNAT2*	0.42505622	1.03897022	0.00461695	0.01191481
ENSMUSG00000030004	*NAT8*	-0.59262059	0.50774904	0.00462555	0.01193281
ENSMUSG00000074340	*OVGP1*	-0.62571374	0.33098173	0.00466801	0.012036
ENSMUSG00000109032	*GM7972*	1.07375023	-1.43411036	0.00469345	0.01208675
ENSMUSG00000045875	*ADRA1A*	-0.8229438	-0.60106352	0.00479255	0.01233331
ENSMUSG00000055692	*TMEM191*	-0.95598309	-1.04195865	0.00480473	0.01235815
ENSMUSG00000049904	*TMEM17*	-0.38320937	0.45433204	0.00484384	0.01244783
ENSMUSG00000049119	*FAM110B*	-0.61443257	-0.20628151	0.00484942	0.01246001
ENSMUSG00000117226	*GM31235*	-0.44214523	1.66298654	0.00487296	0.01251172
ENSMUSG00000050914	*ANKRD37*	0.90352226	1.29667155	0.00491018	0.01259394
ENSMUSG00000024352	*SPATA24*	-0.49863568	0.6240209	0.00492398	0.01262507

ENSMUSG00000039496	*CDNF*	-0.54717583	-0.4450133	0.00496889	0.01273353
ENSMUSG00000103046		-0.8050988	-0.93571557	0.0049928	0.01279259
ENSMUSG00000106831		0.53103954	1.47968503	0.0050046	0.01281611
ENSMUSG00000090952		-0.44438157	1.43568704	0.00504276	0.01290258
ENSMUSG00000029095	*ABLIM2*	-0.62448764	0.02305766	0.00507912	0.01298657
ENSMUSG00000020674	*PXDN*	-0.40075936	1.66125592	0.00510972	0.01305344
ENSMUSG00000041577	*PRELP*	-0.76881785	-1.02734101	0.00514069	0.01312799
ENSMUSG00000030247	*KCNJ8*	-0.55611082	0.79488726	0.00516224	0.01317156
ENSMUSG00000097885		-0.87439764	-1.22872332	0.00521397	0.01329663
ENSMUSG00000034845	*PLVAP*	0.60798731	0.79056526	0.00522137	0.01331086
ENSMUSG00000025500	*LMNTD2*	-0.74178358	-0.48514914	0.00523563	0.01334258
ENSMUSG00000047810	*CCDC88B*	0.56182473	0.27688895	0.00529088	0.01346702
ENSMUSG00000034037	*FGD5*	0.45490466	0.81751536	0.0052939	0.01347238
ENSMUSG00000032666	*1700025G04RIK*	-0.63650155	0.43008309	0.00535568	0.01361545
ENSMUSG00000000628	*HK2*	0.77565151	0.63225375	0.00536415	0.0136346
ENSMUSG00000075266	*CENPW*	-0.47348503	0.48882981	0.00539883	0.01371326
ENSMUSG00000074580	*4931440P22RIK*	-0.79095409	-0.79559409	0.00552267	0.01399152
ENSMUSG00000042439	*ZFP532*	-0.45365434	0.92766556	0.0055616	0.014078
ENSMUSG00000112639		-0.76634426	-0.80105524	0.00557918	0.01411764
ENSMUSG00000110630		-0.63454173	-0.34471267	0.00557839	0.01411764
ENSMUSG00000037466	*TEDC1*	-0.57051122	0.12339471	0.00559229	0.0141435
ENSMUSG00000070425	*XNTRPC*	0.67022681	0.41271308	0.0056303	0.01423472
ENSMUSG00000046841	*CKAP4*	-0.46489279	0.51465698	0.00563656	0.0142481
ENSMUSG00000050737	*PTGES*	0.83506386	1.74271631	0.00568309	0.0143509
ENSMUSG00000026785	*PKN3*	-0.43113186	0.50750705	0.00577009	0.0145356
ENSMUSG00000091154		-0.52088885	-0.07487677	0.00581037	0.01462703
ENSMUSG00000092241	*GM20522*	-0.71664027	-0.93667757	0.00582606	0.01466151
ENSMUSG00000055632	*HMCN2*	-0.41594508	1.13986438	0.00585902	0.01473434
ENSMUSG00000048455	*SPRR1B*	-1.36141312	-1.83849704	0.00593583	0.01491219
ENSMUSG00000071719	*NALF2*	-0.41429519	1.54337906	0.00595406	0.01494775

ENSMUSG00000028159	*DAPP1*	0.54300375	0.15694465	0.00596603	0.01497012
ENSMUSG00000032375	*APH1B*	-0.48723212	0.52820016	0.00600979	0.01506448
ENSMUSG00000055633	*ZFP580*	-0.92030024	-1.3046024	0.00604727	0.01515584
ENSMUSG00000058625	*GM17383*	0.44952333	3.20541208	0.0060618	0.01518966
ENSMUSG00000054931	*ZKSCAN4*	-0.753558	-0.95404757	0.00606383	0.01519217
ENSMUSG00000017724	*ETV4*	-0.62830696	-0.15136048	0.00608032	0.01523087
ENSMUSG00000053137	*MAPK11*	0.65744153	0.47918377	0.00608657	0.01524134
ENSMUSG00000060093	*H4C1*	1.37596412	-1.63847536	0.00609531	0.01525802
ENSMUSG00000106870		-0.65870148	-0.6837206	0.00614534	0.01537277
ENSMUSG00000029822	*OSBPL3*	0.39851525	3.89113642	0.00619704	0.01549153
ENSMUSG00000081600	*GM12286*	0.42419883	1.13208858	0.00627509	0.01566267
ENSMUSG00000025743	*SDC3*	-0.47137949	1.62140753	0.00630432	0.01573294
ENSMUSG00000116358		0.4430061	5.08893736	0.0063628	0.01585194
ENSMUSG00000074738	*FNDC10*	-0.95358299	-1.17757105	0.00638862	0.01590278
ENSMUSG00000031737	*IRX5*	-0.74062402	-0.8776799	0.00638994	0.01590336
ENSMUSG00000093916		-0.80155346	-1.17994729	0.00639411	0.01591104
ENSMUSG00000053964	*LGALS4*	-0.42334714	1.44978008	0.00639644	0.01591416
ENSMUSG00000029231	*PDGFRA*	-1.10094943	-1.4815436	0.00640743	0.0159388
ENSMUSG00000046916	*MYCT1*	-0.54080712	-0.16735726	0.00643013	0.01598984
ENSMUSG00000035498	*CDCP1*	-0.52310158	1.69316357	0.00648905	0.01611452
ENSMUSG00000074652	*MYH7B*	1.78741588	-1.84027176	0.00656063	0.01626203
ENSMUSG00000082896		0.43883421	1.1243485	0.00658278	0.01630592
ENSMUSG00000054967	*ZFP647*	-0.42788543	0.01870228	0.00673607	0.01665754
ENSMUSG00000018983	*E2F2*	0.53781149	0.55632912	0.006738	0.01665951
ENSMUSG00000048960	*PREX2*	-0.94577429	-0.77332144	0.00694707	0.01711589
ENSMUSG00000027796	*SMAD9*	0.48281094	0.69977663	0.00695711	0.01713776
ENSMUSG00000026463	*ATP2B4*	0.46055282	0.94046599	0.00696182	0.01714648
ENSMUSG00000021118	*PLEK2*	-0.53936789	0.63978933	0.00706381	0.01738894
ENSMUSG00000022639		0.40552882	1.43476335	0.00716475	0.01761084
ENSMUSG00000031842	*PDE4C*	-0.39863959	1.44903175	0.00717676	0.01763152

ENSMUSG00000060470	*ADGRG3*	-0.62861093	-0.00176758	0.00720414	0.01768693
ENSMUSG00000090389		0.49904915	0.97068719	0.007208	0.01769347
ENSMUSG00000089756	*ZFP966*	0.40377904	1.45447195	0.00721557	0.01769726
ENSMUSG00000089756	*ZFP969*	0.40377904	1.45447195	0.00721557	0.01769726
ENSMUSG00000026228	*HTR2B*	-0.59218803	0.25945648	0.00723219	0.01772617
ENSMUSG00000107331		-0.72046401	-0.83001802	0.00724258	0.01774868
ENSMUSG00000035983	*GM7008*	0.82882634	-1.10694096	0.0072492	0.01776193
ENSMUSG00000041774	*YDJC*	0.58057641	0.39037373	0.00733293	0.01794611
ENSMUSG00000030827	*FGF21*	-0.89221737	-0.58657652	0.00735527	0.0179978
ENSMUSG00000054065	*PKP3*	-0.65132927	-0.29793746	0.00739312	0.01807836
ENSMUSG00000053965	*PDE5A*	0.45539913	2.10713179	0.00745004	0.01820845
ENSMUSG00000068452	*DUOX2*	-1.27436069	-2.13801815	0.00746064	0.01823132
ENSMUSG00000031803	*B3GNT3*	0.4866584	0.75659816	0.0074984	0.01829922
ENSMUSG00000100347	*GM7895*	-0.56339555	0.32333815	0.00757542	0.01846572
ENSMUSG00000113640	*ADAT3*	0.79471557	-0.00808524	0.00758689	0.01848447
ENSMUSG00000028039	*EFNA3*	1.38286151	-1.42739073	0.00763022	0.01857772
ENSMUSG00000029151	*SLC30A3*	-0.73253496	-1.26757759	0.00766087	0.01863688
ENSMUSG00000074171		-0.58361584	-0.20048039	0.00766529	0.01864455
ENSMUSG00000029283	*CDC7*	0.54698501	-0.21561483	0.00767512	0.01866536
ENSMUSG00000001300	*EFNB2*	-0.54128975	-0.1303878	0.00776438	0.01886057
ENSMUSG00000109674		-1.06488983	-1.51152758	0.00782862	0.01898835
ENSMUSG00000043015	*NEMP2*	-0.59331457	-0.47380174	0.00786047	0.01904986
ENSMUSG00000107201		-0.96317298	-1.4150843	0.00786425	0.01905588
ENSMUSG00000085133		-0.39360798	1.16264828	0.00787361	0.01907226
ENSMUSG00000062960	*KDR*	0.57281172	1.13043888	0.00788327	0.01908937
ENSMUSG00000051220	*ERCC6L*	-0.49565421	0.17877811	0.00788837	0.01909857
ENSMUSG00000110980		-0.94511374	-1.79300662	0.00796424	0.01926637
ENSMUSG00000086742	*GM16201*	0.83457039	1.2135347	0.00796361	0.01926637
ENSMUSG00000025271	*PFKFB1*	-0.38883854	2.66784155	0.00805092	0.01944083
ENSMUSG00000014837	*MATCAP1*	0.65930259	2.13946696	0.00806375	0.01946862

ENSMUSG00000020681	*ACE*	-0.44567648	1.52540475	0.00810123	0.01954303
ENSMUSG00000047473	*ZFP30*	-0.53309064	-0.01896668	0.00810529	0.01954962
ENSMUSG00000038022	*MINDY4*	-0.44785641	0.47979547	0.00812345	0.01958056
ENSMUSG00000097375		0.42213078	0.80419777	0.00812566	0.01958266
ENSMUSG00000040552	*C3AR1*	1.29555025	-1.16722772	0.00814936	0.01963334
ENSMUSG00000097493		0.59567103	0.14547445	0.0081807	0.01969592
ENSMUSG00000038777	*SEMA6C*	-1.07437944	-1.73763948	0.00821705	0.01977171
ENSMUSG00000023505	*CDCA3*	-0.67805954	-0.7467491	0.008263	0.01985842
ENSMUSG00000113440		-0.49880004	-0.39634271	0.00828173	0.01989348
ENSMUSG00000024524	*GNAL*	-0.55923132	0.42307089	0.00833976	0.02002307
ENSMUSG00000031785	*ADGRG1*	0.43441558	1.45700898	0.00835911	0.0200564
ENSMUSG00000086765	*GM11827*	-0.63278731	0.98904534	0.00836979	0.02007547
ENSMUSG00000118506	*CFAP141*	0.46564704	1.14659488	0.0084005	0.02014254
ENSMUSG00000037411	*SERPINE1*	1.17840062	7.98773346	0.00847872	0.02032346
ENSMUSG00000073427	*GM4924*	-0.39322285	0.72752305	0.00855679	0.02048719
ENSMUSG00000032671	*A930018P22RIK*	-0.83937074	-1.40547852	0.00856058	0.02049293
ENSMUSG00000023979	*GUCA1B*	0.41118739	0.94195575	0.00862518	0.0206274
ENSMUSG00000032657	*ENTREP3*	0.55094349	0.46178414	0.0087131	0.02080714
ENSMUSG00000041607	*MBP*	-0.44521477	0.74303083	0.00876013	0.02090247
ENSMUSG00000097413		-0.5513592	-0.28134769	0.00876776	0.02091387
ENSMUSG00000029177	*CENPA*	-0.54656857	0.24032674	0.00876741	0.02091387
ENSMUSG00000005124	*CCN4*	0.39845128	1.84845502	0.00891212	0.02121686
ENSMUSG00000028358	*ZFP618*	-0.67489879	-0.47011644	0.00892161	0.02123601
ENSMUSG00000028807	*ZBTB8A*	0.453347	0.83543915	0.00894609	0.02128047
ENSMUSG00000052131	*AKR1B7*	0.73084192	-0.1652657	0.00897835	0.02134907
ENSMUSG00000073599	*ECSCR*	0.93318095	-0.54906295	0.00898188	0.02134907
ENSMUSG00000098985	*GM27219*	0.99083057	-1.16016501	0.00899885	0.0213852
ENSMUSG00000085192		0.69018421	-0.63640511	0.0090178	0.02142328
ENSMUSG00000060044	*TMEM26*	-1.27372958	-1.84307556	0.00902824	0.02144462
ENSMUSG00000019768	*ESR1*	-0.39681058	1.50547895	0.00907291	0.02152635

ENSMUSG00000052949	*RNF157*	-0.86023896	-1.09162421	0.00916631	0.02171639
ENSMUSG00000034573	*PTPN13*	-0.46731707	1.10273541	0.00917123	0.02172453
ENSMUSG00000112527	*GM35696*	0.58457994	0.42382872	0.00925457	0.0219078
ENSMUSG00000042745	*ID1*	0.69069074	-0.31084314	0.00932539	0.02205768
ENSMUSG00000036777	*ANLN*	-1.22903631	-1.59745325	0.00935132	0.02211545
ENSMUSG00000025265	*FGD1*	-0.77974093	-1.16763792	0.00941032	0.02224423
ENSMUSG00000114004	*GM48552*	1.63097904	-1.83018407	0.00951891	0.02247558
ENSMUSG00000039693	*MSANTD3*	0.55560586	1.69175019	0.00952487	0.02248604
ENSMUSG00000049414		0.43942073	1.13553228	0.00952823	0.02249035
ENSMUSG00000022362		0.53196182	1.1641755	0.00953659	0.02250648
ENSMUSG00000048967	*YJEFN3*	0.67002995	-0.64110182	0.00967773	0.02280659
ENSMUSG00000118423	*LRRC70*	-1.03920644	-1.41451413	0.00973621	0.02293336
ENSMUSG00000047712	*UST*	0.55643528	1.13301776	0.00985357	0.02317633
ENSMUSG00000038156	*SPON1*	-0.53207764	0.61137205	0.00991124	0.02330079
ENSMUSG00000091952		0.87220353	-0.92881672	0.00993686	0.02335354
ENSMUSG00000025964	*ADAM23*	-0.52374897	0.31718605	0.00993946	0.02335591
ENSMUSG00000035799	*TWIST1*	-0.65447676	-0.22324275	0.00996517	0.02341257
ENSMUSG00000028634	*HIVEP3*	0.6219329	-0.19015361	0.01010732	0.02370481
ENSMUSG00000043013	*ONECUT1*	-0.82408629	0.81783985	0.01014201	0.023771
ENSMUSG00000117628		-0.40845521	0.87647634	0.01018519	0.02385697
ENSMUSG00000097675	*1700101I11RIK*	-0.47584416	-0.34687886	0.01021876	0.02392798
ENSMUSG00000116305		1.07877236	-1.26994636	0.01022726	0.02394405
ENSMUSG00000036109	*MBNL3*	-0.67376809	-0.17736006	0.01027988	0.02405192
ENSMUSG00000097616	*1110019D14RIK*	0.43514369	0.52173703	0.01028363	0.02405686
ENSMUSG00000026271	*GPR35*	0.64116619	0.0140501	0.01035374	0.02419772
ENSMUSG00000073374		-0.65697006	0.01887403	0.01036256	0.02421449
ENSMUSG00000027314	*DLL4*	0.46978225	1.06228298	0.01041709	0.02432643
ENSMUSG00000030945	*ACSM2*	-0.68786607	-0.59328614	0.01047872	0.02445867
ENSMUSG00000087177	*E130307A14RIK*	-0.76683469	-1.27028271	0.01049987	0.02449636
ENSMUSG00000110684		0.63839857	-0.74801091	0.01051268	0.02452043

ENSMUSG00000014813	*STC1*	1.04362709	-1.11363225	0.01051353	0.02452043
ENSMUSG00000022610	*MAPK12*	-0.78167734	-0.90508899	0.01051905	0.02452942
ENSMUSG00000034786	*GPSM3*	0.72884489	-0.88933296	0.01055996	0.02461309
ENSMUSG00000034783	*CD207*	-1.03753871	-1.62667654	0.01058747	0.02466937
ENSMUSG00000028874	*FGR*	-0.99000868	-1.32435772	0.01064716	0.02480059
ENSMUSG00000109829		-0.73764095	-1.03889999	0.01071642	0.02492725
ENSMUSG00000095528	*GM10375*	0.43216336	1.9886729	0.01083643	0.02518155
ENSMUSG00000112249	*GM30262*	0.5961897	-0.39379694	0.01098603	0.02549288
ENSMUSG00000043993		-0.61119407	0.07903655	0.01110714	0.02572511
ENSMUSG00000035211	*XRRA1*	-0.91412094	-1.59331862	0.01124512	0.02600777
ENSMUSG00000024501	*DPYSL3*	0.82842673	0.18329915	0.01125191	0.02601937
ENSMUSG00000019960	*DUSP6*	0.41792624	4.00308608	0.01125946	0.02602864
ENSMUSG00000022756	*SLC7A4*	-0.65009314	-0.31813987	0.01129446	0.026089
ENSMUSG00000054435	*GIMAP4*	0.40652021	1.04570326	0.01130397	0.02610277
ENSMUSG00000033350	*CHST2*	-0.72942672	-0.59125183	0.01152249	0.02654474
ENSMUSG00000007033	*HSPA1L*	-1.08433583	-1.42473683	0.0115349	0.026565
ENSMUSG00000035095	*FAM167A*	0.93420943	-0.96818598	0.01157167	0.02664133
ENSMUSG00000097657	*GM7389*	0.43650918	0.13916093	0.01157854	0.02665247
ENSMUSG00000027994	*MCUB*	-0.87924513	-0.85295616	0.01159047	0.02667207
ENSMUSG00000032420	*NT5E*	-0.62261444	3.10904374	0.01168739	0.02686983
ENSMUSG00000085615		-0.40629151	0.91147614	0.01180585	0.02711246
ENSMUSG00000103901		-0.69834466	-1.05892038	0.01184874	0.02720246
ENSMUSG00000020120	*PLEK*	1.11109456	-0.95725554	0.01190685	0.02732305
ENSMUSG00000021210	*AKR1C6*	-0.41039838	2.59183219	0.0120128	0.02751457
ENSMUSG00000097415	*AU020206*	-0.41685083	0.71341188	0.01216479	0.02784532
ENSMUSG00000092471	*CYP21A2-PS*	-0.62080281	0.16469982	0.01217631	0.02786735
ENSMUSG00000111417		-0.71529874	-1.03222814	0.01226344	0.02804054
ENSMUSG00000070729		0.48446831	0.08875304	0.0125335	0.02857794
ENSMUSG00000041605	*INAVA*	0.38116521	1.7119692	0.0125738	0.02865648
ENSMUSG00000011263	*EXOC3L2*	1.13512249	-1.22302838	0.01258032	0.02866689

ENSMUSG00000097994		0.50706606	0.0809557	0.01262828	0.02874612
ENSMUSG00000046589	*LRRC8E*	-0.38521112	0.56247038	0.01265488	0.02880102
ENSMUSG00000026039	*SGO2A*	-0.64235441	-0.42753548	0.01274289	0.02897888
ENSMUSG00000071347	*C1QTNF9*	0.67453523	-0.45271783	0.01277789	0.02904948
ENSMUSG00000047534	*MIS18BP1*	-0.76593024	-1.12050976	0.01289813	0.02926982
ENSMUSG00000091994	*E130317F20RIK*	-0.58687587	-0.35090384	0.01296046	0.02938718
ENSMUSG00000074215		-0.56397809	-0.88914466	0.01305887	0.02960118
ENSMUSG00000078134	*SRSF3-PS*	0.72271634	-0.19569072	0.01321074	0.02991312
ENSMUSG00000045551	*FPR1*	2.25153356	-1.82984346	0.01343648	0.03035403
ENSMUSG00000022070	*BORA*	0.49439923	0.21074053	0.01346359	0.03041061
ENSMUSG00000023959	*CLIC5*	-0.55251263	0.08900665	0.01352039	0.03051909
ENSMUSG00000039137	*WHRN*	-0.49056391	0.26523534	0.01352201	0.03051909
ENSMUSG00000001506	*COL1A1*	-1.19131456	-1.45607777	0.01357143	0.03061182
ENSMUSG00000085274		0.91269645	-1.40302986	0.01375523	0.03100259
ENSMUSG00000038754	*ELOVL3*	-0.4885561	0.78966623	0.01387069	0.03123404
ENSMUSG00000019906	*LIN7A*	-0.60055949	0.00635834	0.01396614	0.03142969
ENSMUSG00000022180	*SLC7A8*	1.08854162	-1.53845855	0.01407048	0.0316209
ENSMUSG00000001741	*IL16*	0.90500466	-0.36653756	0.01414531	0.03176476
ENSMUSG00000050234	*GJA4*	-0.93146941	-1.48235482	0.01423165	0.03194399
ENSMUSG00000086213	*A330040F15RIK*	-0.55662895	-0.03428997	0.01423754	0.03195232
ENSMUSG00000015980	*LRRC27*	0.53371357	-0.3520996	0.01424833	0.03196677
ENSMUSG00000019990	*PDE7B*	-0.88773673	-1.35450578	0.01434688	0.0321633
ENSMUSG00000029561	*OASL2*	-0.41207253	1.73067222	0.01437229	0.03221249
ENSMUSG00000040990	*SH3KBP1*	-0.45645239	0.56349591	0.01453581	0.03254214
ENSMUSG00000045930	*CLEC14A*	-1.13550137	-1.13593286	0.01456645	0.03260078
ENSMUSG00000050370	*CH25H*	-0.78405386	-0.73300523	0.01464805	0.03276343
ENSMUSG00000113328		-0.4512549	0.4730345	0.0147652	0.03301542
ENSMUSG00000107167	*B3GALT9*	0.48730824	0.10905447	0.01480273	0.0330881
ENSMUSG00000085468		0.73951753	-0.1731428	0.01488091	0.03323364
ENSMUSG00000087357		1.42770737	-1.77679076	0.01498872	0.033449

ENSMUSG00000005950	*P2RX5*	0.41409596	2.31074855	0.01514977	0.03378785
ENSMUSG00000030970	*CTBP2*	0.76571385	-0.55586818	0.01528353	0.03406548
ENSMUSG00000072605	*GM10376*	-2.11524474	-0.89998541	0.01543249	0.03438183
ENSMUSG00000114500	*GM8983*	-0.55625945	-0.49545874	0.01557003	0.0346567
ENSMUSG00000074896	*IFIT3*	-0.55850324	3.40056546	0.01560249	0.03471843
ENSMUSG00000030968	*PDILT*	-0.62105641	-0.82901565	0.01560508	0.03471894
ENSMUSG00000087445		-0.81377411	-1.53495232	0.01565962	0.03481392
ENSMUSG00000040732	*ERG*	-0.4171312	0.86532376	0.01568914	0.0348637
ENSMUSG00000001672	*MARVELD3*	0.54951307	0.26367518	0.01575137	0.03499139
ENSMUSG00000049001	*NDNF*	-1.20517979	-2.42709646	0.01583537	0.03515142
ENSMUSG00000034379	*WDR5B*	-0.39684905	0.47850375	0.01584451	0.03516638
ENSMUSG00000029001	*FBXO44*	-1.65112348	-2.07202124	0.01635741	0.03618987
ENSMUSG00000021456	*FBP2*	0.49380784	0.33791045	0.01641294	0.03630179
ENSMUSG00000112300		0.67695414	-0.59984545	0.01649697	0.0364547
ENSMUSG00000000290	*ITGB2*	0.64274556	0.48992088	0.01650794	0.03647345
ENSMUSG00000036086	*ZRANB3*	-0.45882809	0.05663762	0.01658199	0.03659301
ENSMUSG00000097174		0.41338758	0.36887653	0.01675617	0.03694964
ENSMUSG00000027684	*MECOM*	-1.15955732	-1.39888427	0.01676009	0.03695274
ENSMUSG00000021061	*SPTB*	-1.03269268	-1.55933212	0.01677833	0.03698563
ENSMUSG00000042857	*GM9776*	-0.53965649	-0.58445195	0.01678976	0.03699036
ENSMUSG00000100037		-1.43814108	-1.06341486	0.01682288	0.03705777
ENSMUSG00000066456	*HMGN3*	-0.7059698	-0.43949104	0.01683937	0.03707741
ENSMUSG00000073680	*TMEM88B*	-0.72870431	-0.97313154	0.01686559	0.03712959
ENSMUSG00000054871	*TMEM158*	-0.59442686	-0.42592585	0.0169075	0.0372051
ENSMUSG00000101856	*1700096K18RIK*	-0.4984117	-0.2185139	0.01692543	0.03722977
ENSMUSG00000032528	*VIPR1*	-0.86536416	-1.34845753	0.01705385	0.03745194
ENSMUSG00000068130	*ZFP442*	-0.58636249	-0.54445492	0.01708614	0.03750267
ENSMUSG00000008999	*BMP7*	-0.6615421	-1.1070962	0.01711453	0.03755376
ENSMUSG00000032766	*GNG11*	-0.3960956	0.67069615	0.01713305	0.03758013
ENSMUSG00000037341	*SLC9A7*	0.55777533	0.13063762	0.01715334	0.03761644

ENSMUSG00000021367	*EDN1*	-0.45527208	1.32004052	0.01715771	0.0376204
ENSMUSG00000038074	*FKBP14*	0.3803306	0.7441046	0.01720307	0.03770297
ENSMUSG00000042678	*MYO15A*	-0.8576281	-0.95984348	0.01727371	0.03783521
ENSMUSG00000021822	*PLAU*	-0.73846769	-0.87675207	0.01733585	0.03794928
ENSMUSG00000102555		0.70509864	-0.68229283	0.0173554	0.03798014
ENSMUSG00000032839	*TRPC1*	-0.81089707	-1.02802079	0.01742553	0.03812223
ENSMUSG00000010307	*TMEM86A*	-0.49412027	0.54352252	0.01746729	0.03820791
ENSMUSG00000027329	*SPEF1*	0.50703165	0.48789696	0.01759388	0.03845044
ENSMUSG00000033405	*NUDT15*	0.40071092	0.87231047	0.01792622	0.03909529
ENSMUSG00000110010		0.89497665	-1.4502026	0.01798389	0.03920941
ENSMUSG00000044469	*TNFAIP8L1*	0.50965193	0.89692107	0.01825233	0.03972979
ENSMUSG00000085912	*TRP53COR1*	-0.5155477	0.38373137	0.01826903	0.03976026
ENSMUSG00000006205	*HTRA1*	-1.3516581	-1.69989859	0.01850904	0.04021703
ENSMUSG00000097113	*GM19705*	-0.38534042	0.89266526	0.0185879	0.04035853
ENSMUSG00000025161	*SLC16A3*	0.77672223	4.44604394	0.01865965	0.04049036
ENSMUSG00000026395	*PTPRC*	1.09704901	-1.34819316	0.01866719	0.04050074
ENSMUSG00000074824	*RSLCAN18*	-0.62831754	-0.15643579	0.01870259	0.04055957
ENSMUSG00000086714	*0610009E02RIK*	0.55368569	-0.46432978	0.01872732	0.04058523
ENSMUSG00000044024	*RELL2*	-0.55688365	-0.15647789	0.01874328	0.04061183
ENSMUSG00000106025		0.60617633	-0.24560551	0.01878787	0.04069643
ENSMUSG00000092192	*DNAAF4*	-0.9942831	-1.3419287	0.01890131	0.04090633
ENSMUSG00000105741		0.59226657	-0.74091383	0.0189339	0.04095836
ENSMUSG00000023015	*RACGAP1*	-0.96759107	-1.50861745	0.01903161	0.04113338
ENSMUSG00000091985		0.61056938	-0.59500271	0.0191796	0.0413984
ENSMUSG00000097321	*1700028E10RIK*	-0.45557628	-0.42108494	0.01930286	0.04163384
ENSMUSG00000097321	*GM3704*	-0.45557628	-0.42108494	0.01930286	0.04163384
ENSMUSG00000021451	*SEMA4D*	-0.80012229	-0.90099518	0.01933417	0.04168913
ENSMUSG00000086443		0.38171372	0.66885575	0.01935653	0.04172508
ENSMUSG00000074682	*ZCCHC3*	-0.62017087	-0.90665171	0.01961205	0.04223248
ENSMUSG00000100782	*GM28231*	-0.99469743	-1.65879986	0.0196785	0.04235579

ENSMUSG00000019853	*HEBP2*	0.3936715	1.17375766	0.01967268	0.04235579
ENSMUSG00000057948	*UNC13D*	0.70998223	-0.60451164	0.01970679	0.04239298
ENSMUSG00000037280	*GALNT6*	-0.96539437	-1.55281183	0.01974328	0.04245905
ENSMUSG00000115919	*GM31583*	-0.59651043	-0.47259319	0.01980305	0.04255305
ENSMUSG00000104876		-0.45617666	1.66142079	0.01980082	0.04255305
ENSMUSG00000020814	*MXRA7*	0.47943194	-0.22717923	0.02001716	0.04297252
ENSMUSG00000046056	*SBSN*	-0.71254815	-1.28779735	0.02007819	0.04306164
ENSMUSG00000008153	*CLSTN3*	-0.77722056	-1.10775159	0.02011179	0.04311263
ENSMUSG00000023050	*MAP3K12*	-0.49387477	0.21989088	0.0201437	0.04316843
ENSMUSG00000048915	*EFNA5*	-0.4927402	-0.26640317	0.02015972	0.04319358
ENSMUSG00000043999	*GPR75*	-0.47038103	-0.48255891	0.02033592	0.04352954
ENSMUSG00000074254	*CYP2A4*	-0.3884331	1.86191948	0.02039994	0.04364749
ENSMUSG00000112926		0.64860369	-0.79833018	0.0206063	0.04406334
ENSMUSG00000035948	*ACSS3*	-0.55297555	-0.47596223	0.02070358	0.04422627
ENSMUSG00000055494		-0.62368333	-1.05070396	0.02071637	0.04424714
ENSMUSG00000050989	*SELENON*	-0.39319947	1.77517575	0.02082887	0.04444218
ENSMUSG00000083261	*GM7816*	0.61293641	0.85465159	0.02084578	0.04445889
ENSMUSG00000104682		0.86158199	-1.31597347	0.02115792	0.04505262
ENSMUSG00000100594	*2810414N06RIK*	-0.56253477	-0.39964945	0.02120927	0.04514887
ENSMUSG00000105617		0.72102559	-0.78222741	0.02140396	0.04554351
ENSMUSG00000056592	*ZFP658*	0.52312679	-0.1443539	0.0214211	0.04557337
ENSMUSG00000090381		0.51344213	1.4036286	0.02149158	0.04570345
ENSMUSG00000054690	*EMCN*	-0.6337789	-0.11675855	0.02150642	0.04571514
ENSMUSG00000031262	*CENPI*	-0.65342861	-1.20717731	0.02160044	0.04589508
ENSMUSG00000087377		-0.51466798	-0.46872709	0.02170305	0.04606644
ENSMUSG00000084983		-0.6302935	-0.30057268	0.02181992	0.04629443
ENSMUSG00000030022	*ADAMTS9*	-0.37929165	0.08175607	0.02184219	0.04633498
ENSMUSG00000050821	*FAM131A*	-0.73687753	-1.02520819	0.02208155	0.04678867
ENSMUSG00000117525		1.1810831	-1.69177028	0.02211908	0.04684791
ENSMUSG00000097047	*1110020A21RIK*	-0.62102615	-0.70650494	0.02219154	0.04699461

ENSMUSG00000031398	*PLXNA3*	-0.58543408	-0.39584454	0.02235289	0.04725452
ENSMUSG00000020672	*SNTG2*	-0.78625998	-1.47543915	0.02240957	0.0473607
ENSMUSG00000024462	*GABBR1*	-0.40611972	0.09176226	0.02242349	0.04737664
ENSMUSG00000031227	*MAGEE1*	0.6692309	-0.69932213	0.0224252	0.04737664
ENSMUSG00000035232	*PDK3*	0.77157361	-0.77949028	0.02242722	0.04737664
ENSMUSG00000116861	*GM8253*	-0.73903027	-1.26555228	0.02246618	0.04742791
ENSMUSG00000059810	*RGS3*	-0.45701091	0.43631093	0.02246163	0.04742791
ENSMUSG00000091269	*GM6682*	0.43916544	1.2226791	0.0224836	0.04745567
ENSMUSG00000040434	*LARGE2*	-0.85335225	-1.33336842	0.02267239	0.04778546
ENSMUSG00000086328		-0.91802622	-1.52430211	0.02274618	0.04792723
ENSMUSG00000059461		0.42863112	0.41424324	0.02278359	0.04797164
ENSMUSG00000024053	*EMILIN2*	0.9802929	-0.66455358	0.02280921	0.0480187
ENSMUSG00000054181		-0.7021661	-1.0125012	0.0229316	0.04824869
ENSMUSG00000105176		-0.50091659	-0.51007139	0.02302939	0.04841976
ENSMUSG00000099998	*GM6501*	0.65494361	-0.15045712	0.02330042	0.04893355
ENSMUSG00000117975	*ITPRIP*	0.43672078	0.3300171	0.02344209	0.04920996
ENSMUSG00000080896		-1.78283753	-2.10073472	0.02345805	0.04922937
ENSMUSG00000042962	*GM5436*	-0.74689488	-1.19995197	0.02359211	0.04946125
ENSMUSG00000030559	*RAB38*	-0.60228655	-0.45207107	0.02368679	0.04961017
ENSMUSG00000035578	*IQCG*	-0.46881984	-0.07978126	0.0236826	0.04961017
ENSMUSG00000001128	*CFP*	0.41370099	0.72083488	0.0237933	0.04981903
ENSMUSG00000097099	*GM9917*	0.51516401	-0.36776172	0.02385105	0.04991147

Table S3. Significant differentially expressed genes by BAY-3827 in the presence of MK-8722 in control mouse primary hepatocytes. Differentially expressed genes were defined as significant (abs (FC ≥ 1.3), FDR < 0.05) in BAY-3827 + MK-8722 treated AMPKα1α2$^{+/+}$ (control) primary hepatocytes compared to MK-8722 treatment alone.

REFERENCES AND NOTES

1. D. G. Hardie, F. A. Ross, S. A. Hawley, AMPK: A nutrient and energy sensor that maintains energy homeostasis. *Nat. Rev. Mol. Cell Biol.* **13**, 251–262 (2012).

2. G. R. Steinberg, D. G. Hardie, New insights into activation and function of the AMPK. *Nat. Rev. Mol. Cell Biol.* **24**, 255–272 (2023).

3. D. G. Hardie, K. Sakamoto, AMPK: A key sensor of fuel and energy status in skeletal muscle. *Physiology* **21**, 48–60 (2006).

4. F. A. Ross, C. MacKintosh, D. G. Hardie, AMP-activated protein kinase: A cellular energy sensor that comes in 12 flavours. *FEBS J.* **283**, 2987–3001 (2016).

5. W. J. Smiles, A. J. Ovens, J. S. Oakhil, B. Kofler, The metabolic sensor AMPK: Twelve enzymes in one. *Molecular*. *Metabolism* **90**, 102042 (2024).

6. B. Xiao, M. J. Sanders, E. Underwood, R. Heath, F. V. Mayer, D. Carmena, C. Jing, P. A. Walker, J. F. Eccleston, L. F. Haire, P. Saiu, S. Howell, R. Aasland, S. Martin, D. Carling, S. Gamblin, Structure of mammalian AMPK and its regulation by ADP. *Nature* **472**, 230–233 (2011).

7. C. G. Langendorf, B. E. Kemp, Choreography of AMPK activation. *Cell Res.* **25**, 5–6 (2015).

8. S. A. Hawley, F. M. Russell, D. G. Hardie, AMP-activated protein kinase can be allosterically activated by ADP but AMP remains the key activating ligand. *Biochem. J.* **8**, 587–599 (2024).

9. G. R. Steinberg, D. Carling, AMP-activated protein kinase: The current landscape for drug development. *Nat. Rev. Drug Discov.* **18**, 527–551 (2019).

10. D. Carling, V. A. Zammit, D. G. Hardie, A common bicyclic protein kinase cascade inactivates the regulatory enzymes of fatty acid and cholesterol biosynthesis. *FEBS Lett.* **223**, 217–222 (1987).

11. D. G. Hardie, AMP-activated protein kinase — A journey from 1 to 100 downstream targets. *Biochem. J.* **479**, 2327–2343 (2022).

12. K. Sakamoto, G. D. Holman, Emerging role for AS160/TBC1D4 and TBC1D1 in the regulation of GLUT4 traffic. *Am. J. Physiol. Endocrinol. Metab.* **295**, E29–E37 (2008).

13. Q. Chen, B. Xie, S. Zhu, P. Rong, Y. Sheng, S. Ducommun, L. Chen, C. Quan, M. Li, K. Sakamoto, C. MacKintosh, S. Chen, H. Wang, A Tbc1d1 Ser231Ala-knockin mutation partially impairs AICAR- but not exercise-induced muscle glucose uptake in mice. *Diabetologia* **60**, 336–345 (2017).

14. R. Kjøbsted, J. L. W. Roll, N. O. Jørgensen, J. B. Birk, M. Foretz, B. Viollet, A. Chadt, H. Al-Hasani, J. F. P. Wojtaszewski, AMPK and TBC1D1 regulate muscle glucose uptake after, but not during, exercise and contraction. *Diabetes* **68**, 1427–1440 (2019).

15. B. Cool, B. Zinker, W. Chiou, L. Kifle, N. Cao, M. Perham, R. Dickinson, A. Adler, G. Gagne, R. Iyengar, G. Zhao, K. Marsh, P. Kym, P. Jung, H. Camp, E. Frevert, Identification and characterization of a small molecule AMPK activator that treats key components of type 2 diabetes and the metabolic syndrome. *Cell Metab.* **3**, 403–416 (2006).

16. O. Göransson, A. McBride, S. A. Hawley, F. A. Ross, N. Shpiro, M. Foretz, B. Viollet, D. G. Hardie, K. Sakamoto, Mechanism of action of A-769662, a valuable tool for activation of AMP-activated protein kinase. *J. Biol. Chem.* **282**, 32549–32560 (2007).

17. B. Xiao, M. J. Sanders, D. Carmena, N. J. Bright, L. F. Haire, E. Underwood, B. R. Patel, R. B. Heath, P. A. Walker, S. Hallen, F. Giordanetto, S. Martin, D. Carling, S. Gamblin, Structural basis of AMPK regulation by small molecule activators. *Nat. Commun.* **4**, 3017 (2013).

18. R. W. Myers, H. P. Guan, J. Ehrhart, A. Petrov, S. Prahalada, E. Tozzo, X. Yang, M. M. Kurtz, M. Trujillo, D. G. Trotter, D. Feng, S. Xu, G. Eiermann, M. A. Holahan, D. Rubins, S. Conarello, X. Niu, S. C. Souza, C. Miller, J. Liu, K. Lu, W. Feng, Y. Li, R. Painter, J. Milligan, H. He, F. Liu, A. Ogawa, D. Wisniewski, R. Rohm, L. Wang, M. Bunzel, Y. Qian, W. Zhu, H. Wang, B. Bennet, L. Scheuch, G. Fernandez, C. Li, M. Klimas, G. Zhou, M. Van Heek, T. Biftu, A. Weber, D. Kelley, N. Thornberry, M. Erion, D. Kemp, I. Sebhat, Systemic

pan-AMPK activator MK-8722 improves glucose homeostasis but induces cardiac hypertrophy. *Science* **357**, 507–511 (2017).

19. E. C. Cokorinos, J. Delmore, A. R. Reyes, B. Albuquerque, R. Kjøbsted, N. O. Jørgensen, J. L. Tran, A. Jatkar, K. Cialdea, R. M. Esquejo, J. Meissen, M. F. Calabrese, J. Cordes, R. Moccia, D. Tess, C. T. Salatto, T. M. Coskran, A. C. Opsahl, D. Flynn, M. Blatnik, W. Li, E. Kindt, M. Foretz, B. Viollet, J. Ward, R. Kurumbail, A. Kalgutkar, J. Wojtaszewski, K. Cameron, R. Miller, Activation of skeletal muscle AMPK promotes glucose disposal and glucose lowering in non-human primates and mice. *Cell Metab.* **25**, 1147–1159.e10 (2017).

20. J. E. Strang, D. D. Astridge, V. T. Nguyen, P. Reigan, Small molecule modulators of AMP-activated protein kinase (AMPK) activity and their potential in cancer therapy. *J. Med. Chem.* **68**, 2238–2254 (2025).

21. D. Vara-Ciruelos, F. M. Russell, D. Grahame Hardie, The strange case of AMPK and cancer: Dr Jekyll or Mr Hyde? *Open Biol.* **9**, 190099 (2019).

22. D. R. Alessi, K. Sakamoto, J. R. Bayascas, LKB1-dependent signaling pathways. *Annu. Rev. Biochem.* **75**, 137–163 (2006).

23. R. J. Shaw, N. Bardeesy, B. D. Manning, L. Lopez, M. Kosmatka, R. A. DePinho, L. C. Cantley, The LKB1 tumor suppressor negatively regulates mTOR signaling. *Cancer Cell* **6**, 91–99 (2004).

24. X. Huang, S. Wullschleger, M. Shpiro, V. A. McGuire, K. Sakamoto, Y. L. Woods, W. McBurnie, S. Fleming, D. R. Alessi, Important role of the LKB1-AMPK pathway in suppressing tumorigenesis in PTEN-deficient mice. *Biochem. J.* **412**, 211–221 (2008).

25. F. M. Russell, D. G. Hardie, AMP-activated protein kinase: Do we need activators or inhibitors to treat or prevent cancer? *Int. J. Mol. Sci.* **22**, 186 (2020).

26. T. Monteverde, N. Muthalagu, J. Port, D. J. Murphy, Evidence of cancer-promoting roles for AMPK and related kinases. *FEBS J.* **282**, 4658–4671 (2015).

27. G. Zhou, R. Myers, Y. Li, Y. Chen, X. Shen, J. Fenyk-Melody, M. Wu, J. Ventre, T. Doebber, N. Fujii, N. Musi, M. F. Hirshman, L. J. Goodyear, D. E. Moller, Role of AMP-activated protein kinase in mechanism of metformin action. *J. Clin. Investig.* **108**, 1167–1174 (2001).

28. P. B. Yu, C. C. Hong, C. Sachidanandan, J. L. Babitt, D. Y. Deng, S. A. Hoyng, H. Y. Lin, K. D. Bloch, R. T. Peterson, Dorsomorphin inhibits BMP signals required for embryogenesis and iron metabolism. *Nat. Chem. Biol.* **4**, 33–41 (2008).

29. N. Handa, T. Takagi, S. Saijo, S. Kishishita, D. Takaya, M. Toyama, T. Terada, M. Shirouzu, A. Suzuki, S. Lee, T. Yamauchi, M. Okada-Iwabu, M. Iwabu, T. Kadowaki, Y. Minokoshi, S. Yokoyama, Structural basis for compound C inhibition of the human AMP-activated protein kinase α2 subunit kinase domain. *Acta Crystallogr. D Biol. Crystallogr.* **67**, 480–487 (2011).

30. A. Chaikuad, I. Alfano, G. Kerr, C. E. Sanvitale, J. H. Boergermann, J. T. Triffitt, F. Von Delft, S. Knapp, P. Knaus, A. N. Bullock, Structure of the bone morphogenetic protein receptor ALK2 and implications for fibrodysplasia ossificans progressiva. *J. Biol. Chem.* **287**, 36990–36998 (2012).

31. J. Bain, L. Plater, M. Elliott, N. Shpiro, C. J. Hastie, H. Mclauchlan, I. Klevernic, J. S. C. Arthur, D. R. Alessi, P. Cohen, The selectivity of protein kinase inhibitors: A further update. *Biochem. J.* **408**, 297–315 (2007).

32. J. Vogt, R. Traynor, G. P. Sapkota, The specificities of small molecule inhibitors of the TGFß and BMP pathways. *Cell. Signal.* **23**, 1831–1842 (2011).

33. T. A. Dite, C. G. Langendorf, A. Hoque, S. Galic, R. J. Rebello, A. J. Ovens, L. M. Lindqvist, K. R. W. Ngoei, N. X. Y. Ling, L. Furic, B. E. Kemp, J. W. Scott, J. S. Oakhill, AMP-activated protein kinase selectively inhibited by the type II inhibitor SBI-0206965. *J. Biol. Chem.* **293**, 8874–8885 (2018).

34. B. Dasgupta, W. Seibel, Compound C/Dorsomorphin: Its use and misuse as an AMPK inhibitor, in *Methods in Molecular Biology*, (Humana Press Inc., 2018), pp. 195–202.

35. D. F. Egan, M. G. H. Chun, M. Vamos, H. Zou, J. Rong, C. J. Miller, H. J. Lou, D. Raveendra-Panickar, C. C. Yang, D. J. Sheffler, P. Teriete, J. Asara, B. Turk, N. Cosford, R. Shaw, Small molecule inhibition of the autophagy kinase ULK1 and identification of ULK1 substrates. *Mol. Cell* **59**, 285–297 (2015).

36. D. Ahwazi, K. Neopane, G. R. Markby, F. Kopietz, A. J. Ovens, M. Dall, A. S. Hassing, P. Gräsle, Y. Alshuweishi, J. T. Treebak, I. Salt, O. Göransson, E. Zeqiraj, J. Scott, K. Sakamoto, Investigation of the specificity and mechanism of action of the ULK1/AMPK inhibitor SBI-0206965. *Biochem. J.* **478**, 2977–2997 (2021).

37. C. Lemos, V. K. Schulze, S. J. Baumgart, E. Nevedomskaya, T. Heinrich, J. Lefranc, B. Bader, C. D. Christ, H. Briem, L. P. Kuhnke, S. Holton, U. Bömer, P. Lienau, F. von Nussbaum, C. Nising, M. Bauser, A. Hägebarth, D. Mumberg, B. Haendler, The potent AMPK inhibitor BAY-3827 shows strong efficacy in androgen-dependent prostate cancer models. *Cell. Oncol.* **44**, 581–594 (2021).

38. S. A. Hawley, F. M. Russell, F. A. Ross, D. G. Hardie, BAY-3827 and SBI-0206965: Potent ampk inhibitors that paradoxically increase Thr172 phosphorylation. *Int. J. Mol. Sci.* **25**, 453 (2024).

39. G. Manning, D. B. Whyte, R. Martinez, T. Hunter, S. Sudarsanam, The protein kinase complement of the human genome. *Science* **298**, 1912–1934 (2002).

40. H. Ashkenazy, S. Abadi, E. Martz, O. Chay, I. Mayrose, T. Pupko, N. Ben-Tal, ConSurf 2016: An improved methodology to estimate and visualize evolutionary conservation in macromolecules. *Nucleic Acids Res.* **44**, W344–W350 (2016).

41. B. Yariv, E. Yariv, A. Kessel, G. Masrati, A. B. Chorin, E. Martz, I. Mayrose, T. Pupko, N. Ben-Tal, Using evolutionary data to make sense of macromolecules with a “face-lifted” ConSurf. *Protein Sci.* **32**, e4582 (2023).

42. C. G. Langendorf, K. R. W. Ngoei, J. W. Scott, N. X. Y. Ling, S. M. A. Issa, M. A. Gorman, M. W. Parker, K. Sakamoto, J. S. Oakhill, B. E. Kemp, Structural basis of allosteric and

synergistic activation of AMPK by furan-2-phosphonic derivative C2 binding. *Nat. Commun.* **7**, 10912 (2016).

43. V. Modi, R. L. Dunbrack, Defining a new nomenclature for the structures of active and inactive kinases. *Proc. Natl. Acad. Sci. U.S.A.* **116**, 6818–6827 (2019).

44. S. S. Taylor, M. M. Keshwani, J. M. Steichen, A. P. Kornev, Evolution of the eukaryotic protein kinases as dynamic molecular switches. *Philos. Trans. R. Soc. Lond. B. Biol. Sci.* **367**, 2517–2528 (2012).

45. M. J. Sanders, Y. Ratinaud, K. Neopane, N. Bonhoure, E. A. Day, O. Ciclet, S. Lassueur, M. N. Pinta, M. Deak, B. Brinon, S. Christen, G. Steinberg, D. Barron, K. Sakamoto, Natural (dihydro)phenanthrene plant compounds are direct activators of AMPK through its allosteric drug and metabolite-binding site. *J. Biol. Chem.* **298**, 101852 (2022).

46. N. Boudaba, A. Marion, C. Huet, R. Pierre, B. Viollet, M. Foretz, Ampk re-activation suppresses hepatic steatosis but its downregulation does not promote fatty liver development. *EBioMedicine* **28**, 194–209 (2018).

47. R. W. Hunter, M. Foretz, L. Bultot, M. D. Fullerton, M. Deak, F. A. Ross, S. A. Hawley, N. Shpiro, B. Viollet, D. Barron, B. Kemp, G. Steinberg, D. Hardie, K. Sakamoto, Mechanism of action of compound-13: An α1-selective small molecule activator of AMPK. *Chem. Biol.* **21**, 866–879 (2014).

48. T. Hashimoto, Y. Urushihara, Y. Murata, Y. Fujishima, Y. Hosoi, AMPK increases expression of ATM through transcriptional factor Sp1 and induces radioresistance under severe hypoxia in glioblastoma cell lines. *Biochem. Biophys. Res. Commun.* **590**, 82–88 (2022).

49. X. S. Rao, X. X. Cong, X. K. Gao, Y. P. Shi, L. J. Shi, J. F. Wang, C. Y. Ni, M. J. He, Y. Xu, C. Yi, Z. Meng, J. Liu, P. Lin, L. Zheng, Y. Zhou, AMPK-mediated phosphorylation enhances the auto-inhibition of TBC1D17 to promote Rab5-dependent glucose uptake. *Cell Death Differ.* **28**, 3214–3234 (2021).

50. K. Yamano, A. I. Fogel, C. Wang, A. M. van der Bliek, R. J. Youle, Mitochondrial Rab GAPs govern autophagosome biogenesis during mitophagy. *eLife* **3**, e01612 (2014).

51. W. S. Yang, M. J. Caliva, V. S. Khadka, M. Tiirikainen, M. L. Matter, Y. Deng, J. W. Ramos, RSK1 and RSK2 serine/threonine kinases regulate different transcription programs in cancer. *Front. Cell Dev. Biol.* **10**, 1015665 (2023).

52. G. P. Sapkota, L. Cummings, F. S. Newell, C. Armstrong, J. Bain, M. Frodin, M. Grauert, M. Hoffmann, G. Schnapp, M. Steegmaier, P. Cohen, D. Alessi, BI-D1870 is a specific inhibitor of the p90 RSK (ribosomal S6 kinase) isoforms in vitro and in vivo. *Biochem. J.* **401**, 29–38 (2007).

53. D. Utepbergenov, U. Derewenda, N. Olekhnovich, G. Szukalska, B. Banerjee, M. K. Hilinski, D. A. Lannigan, P. T. Stukenberg, Z. S. Derewenda, Insights into the inhibition of the p90 ribosomal S6 kinase (RSK) by the flavonol glycoside SL0101 from the 1.5 Å crystal structure of the N-terminal domain of RSK2 with bound inhibitor. *Biochemistry* **51**, 6499–6510 (2012).

54. R. Jain, M. Mathur, J. Lan, A. Costales, G. Atallah, S. Ramurthy, S. Subramanian, L. Setti, P. Feucht, B. Warne, L. Doyle, S. Basham, A. Jefferson, M. Lindvall, B. Appleton, C. Shafer, Discovery of potent and selective RSK inhibitors as biological probes. *J. Med. Chem.* **58**, 6766–6783 (2015).

55. B. W. Murray, C. Guo, J. Piraino, J. K. Westwick, C. Zhang, J. Lamerdin, E. Dagostino, D. Knighton, C. M. Loi, M. Zager, E. Kraynov, I. Popoff, J. Christensen, R. Martinez, S. Kephart, J. Marakovits, S. Karlicek, S. Bergqvist, T. Smeal, Small-molecule p21-activated kinase inhibitor PF-3758309 is a potent inhibitor of oncogenic signaling and tumor growth. *Proc. Natl. Acad. Sci. U.S.A.* **107**, 9446–9451 (2010).

56. C. Schneider, J. Hilbert, F. Genevaux, S. Höfer, L. Krauß, F. Schicktanz, C. T. Contreras, S. Jansari, A. Papargyriou, T. Richter, A. M. Alfayomy, C. Falcomatà, C. Schneeweis, F. Orben, R. Öllinger, F. Wegwitz, A. Boshnakovska, P. Rehling, D. Müller, P. Ströbel, V. Ellenrieder, L. Conradi, E. Hessmann, M. Ghadimi, M. Grade, M. Wirth, K. Steiger, R.

Rad, B. Kuster, W. Sippl, M. Reichert, D. Saur, G. Schneider, A novel AMPK inhibitor sensitizes pancreatic cancer cells to ferroptosis induction. *Adv. Sci.* **11**, 2307695 (2024).

57. H. Möbitz, The ABC of protein kinase conformations. *Biochim. Biophys. Acta* **1854**, 1555–1566 (2015).

58. D. P. Byrne, S. Shrestha, M. Galler, M. Cao, L. A. Daly, A. E. Campbell, C. E. Eyers, E. A. Veal, N. Kannan, P. A. Eyers, Aurora A regulation by reversible cysteine oxidation reveals evolutionarily conserved redox control of Ser/Thr protein kinase activity. *Sci. Signal.* **639**, 2713 (2020).

59. X. Huang, M. Begley, K. A. Morgenstern, Y. Gu, P. Rose, H. Zhao, X. Zhu, Crystal structure of an inactive Akt2 kinase domain. *Structure* **1**, 21–30 (2003).

60. R. Anjum, J. Blenis, The RSK family of kinases: Emerging roles in cellular signalling. *Nat. Rev. Mol. Cell Biol.* **9**, 747–758 (2008).

61. F. Koutsougianni, D. Alexopoulou, A. Uvez, A. Lamprianidou, E. Sereti, C. Tsimplouli, E. Ilkay Armutak, K. Dimas, P90 ribosomal S6 kinases: A bona fide target for novel targeted anticancer therapies? *Biochem. Pharmacol.* **210**, 115488 (2023).

62. B. Viollet, M. Foretz, Animal models to study AMPK. *EXS* **107**, 441–469 (2016).

63. N. Ashraf, J. L. van Nostrand, Fine-tuning AMPK in physiology and disease using point-mutant mouse models. *Dis. Model Mech.* **17**, dmm050798 (2024).

64. K. Sakamoto, A. McCarthy, D. Smith, K. A. Green, D. G. Hardie, A. Ashworth, D. R. Alessi, Deficiency of LKB1 in skeletal muscle prevents AMPK activation and glucose uptake during contraction. *EMBO J.* **24**, 1810–1820 (2005).

65. C. J. Hastie, H. J. McLauchlan, P. Cohen, Assay of protein kinases using radiolabeled ATP: A protocol. *Nat. Protoc.* **1**, 968–971 (2006).

66. T. A. Dite, N. X. Y. Ling, J. W. Scott, A. Hoque, S. Galic, B. L. Parker, K. R. W. Ngoei, C. G. Langendorf, M. T. O'Brien, M. Kundu, G. R. Steinberg, K. Sakamoto, B. E. Kemp, J. S.

Oakhill, The autophagy initiator ULK1 sensitizes AMPK to allosteric drugs. *Nat. Commun.* **8**, 571 (2017).

67. J. W. Scott, E. Park, R. M. Rodriguiz, J. S. Oakhill, S. M. A. Issa, M. T. Obrien, T. A. Dite, C. G. Langendorf, W. C. Wetsel, A. R. Means, B. E. Kemp, Autophosphorylation of CaMKK2 generates autonomous activity that is disrupted by a T85S mutation linked to anxiety and bipolar disorder. *Sci. Rep.* **5**, 14436 (2015).

68. A. M. Waterhouse, J. B. Procter, D. M. A. Martin, M. Clamp, G. J. Barton, Jalview Version 2–A multiple sequence alignment editor and analysis workbench. *Bioinformatics* **25**, 1189–1191 (2009).

69. P. A. Ewels, A. Peltzer, S. Fillinger, H. Patel, J. Alneberg, A. Wilm, M. U. Garcia, P. Di Tommaso, S. Nahnsen, The nf-core framework for community-curated bioinformatics pipelines. *Nat. Biotechnol.* **38**, 276–278 (2020).

70. M. D. Robinson, D. J. McCarthy, G. K. Smyth, edgeR: A bioconductor package for differential expression analysis of digital gene expression data. *Bioinformatics* **26**, 139–140 (2009).

71. M. E. Ritchie, B. Phipson, D. Wu, Y. Hu, C. W. Law, W. Shi, G. K. Smyth, Limma powers differential expression analyses for RNA-sequencing and microarray studies. *Nucleic Acids Res.* **43**, e47 (2015).

72. L. Yan, ggvenn: Draw Venn Diagram by ‘ggplot2’. R package version 0.1.10 (2025); https://github.com/yanlinlin82/ggvennhttps://github.com/yanlinlin82/ggvenn.

73. R. Kolde, pheatmap: Pretty Heatmaps. R package version 1.0.12 (2019); https://CRAN.R-project.org/package=pheatmaphttps://CRAN.R-project.org/package=pheatmap.

74. S. Xu, E. Hu, Y. Cai, Z. Xie, X. Luo, L. Zhan, W. Tang, Q. Wang, B. Liu, R. Wang, W. Xie, T. Wu, L. Xie, G. Yu, Using clusterProfiler to characterize multiomics data. *Nat. Protoc.* **19**, 3292–3320 (2024).

75. R Core Team, R: A language and environment for statistical computing. R Foundation for Statistical Computing, Vienna, Austria (2021); https://R-project.org.

76. C. Vonrhein, C. Flensburg, P. Keller, A. Sharff, O. Smart, W. Paciorek, T. Womack, G. Bricogne, Data processing and analysis with the autoPROC toolbox. *Acta Crystallogr. D Biol. Crystallogr.* **67**, 293–302 (2011).

77. A. J. McCoy, R. W. Grosse-Kunstleve, P. D. Adams, M. D. Winn, L. C. Storoni, R. J. Read, Phaser crystallographic software. *J. Appl. Cryst.* **40**, 658–674 (2007).

78. P. Emsley, B. Lohkamp, W. G. Scott, K. Cowtan, Features and development of coot. *Acta Crystallogr. D Biol. Crystallogr.* **66**, 486–501 (2010).

79. P. V. Afonine, R. W. Grosse-Kunstleve, N. Echols, J. J. Headd, N. W. Moriarty, M. Mustyakimov, T. C. Terwilliger, A. Urzhumtsev, P. H. Zwart, P. D. Adams, Towards automated crystallographic structure refinement with phenix.refine. *Acta Crystallogr. D Biol. Crystallogr.* **68**, 352–367 (2012).

80. The PyMOL Molecular Graphics System, Version 3.0 Schrödinger, LLC.

81. E. F. Pettersen, T. D. Goddard, C. C. Huang, G. S. Couch, D. M. Greenblatt, E. C. Meng, T. E. Ferrin, UCSF chimera - A visualization system for exploratory research and analysis. *J. Comput. Chem.* **25**, 1605–1612 (2004).
